# Biological Activity Evaluation of Phenolic Isatin-3-Hydrazones Containing a Quaternary Ammonium Center of Various Structures

**DOI:** 10.3390/ijms252011130

**Published:** 2024-10-17

**Authors:** Margarita Neganova, Yulia Aleksandrova, Alexandra Voloshina, Anna Lyubina, Nurbol Appazov, Sholpan Yespenbetova, Zulfiia Valiullina, Aleksandr Samorodov, Sergey Bukharov, Elmira Gibadullina, Anipa Tapalova, Andrei Bogdanov

**Affiliations:** 1 Arbuzov Institute of Organic and Physical Chemistry, FRC Kazan Scientific Center, Russian Academy of Sciences, Akad. Arbuzov st. 8, 420088 Kazan, Russia; neganova83@mail.ru (M.N.); elmirak_1978@mail.ru (E.G.); 2 Nesmeyanov Institute of Organoelement Compounds, Russian Academy of Sciences, 119991 Moscow, Russia; 3 Laboratory of Engineering Profile “Physical and Chemical Methods of Analysis”, Korkyt Ata Kyzylorda University, Ayteke bi, Str., 29A, Kyzylorda 120014, Kazakhstan; 4 “CNEC” LLP, Dariger Ali Lane, 2, Kyzylorda 120001, Kazakhstan; 5 Department of Pharmacology, Bashkir State Medical University, Lenin st. 8, 450008 Ufa, Russia; 6 Department of Technology of Basic Organic and Petrochemical Synthesis, Kazan National Research Technological University, K. Marx Str. 68, 420015 Kazan, Russia

**Keywords:** isatin, quaternary ammonium compounds, hydrazones, antioxidants, hemostasis, cytotoxicity

## Abstract

A series of new isatin-3-hydrazones bearing different ammonium fragments was synthesized by a simple and easy work-up reaction of Girard’s reagents analogs with 1-(3,5-di-*tert*-butyl-4-hydroxybenzyl)isatin. All derivatives have been shown to have antioxidant properties. In terms of bactericidal activity against gram-positive bacteria, including methicillin-resistant strains of *Staphylococcus aureus*, the best compounds are **3a**, **3e**, and **3m**, bearing octyl, acetal, and brucine ammonium centers, respectively. In addition, brucine and quinine derivatives **3l**, and **3j** exhibit platelet antiaggregation activity at the level of acetylsalicylic acid, and this series of isatin derivatives does not adversely affect the hemostasis system as a whole. Thus, all the obtained results can lay the groundwork for future pharmaceutical developments for the creation of effective antibacterial drugs with reduced systemic toxicity due to the presence of antioxidant properties.

## 1. Introduction

**Figure 1:**
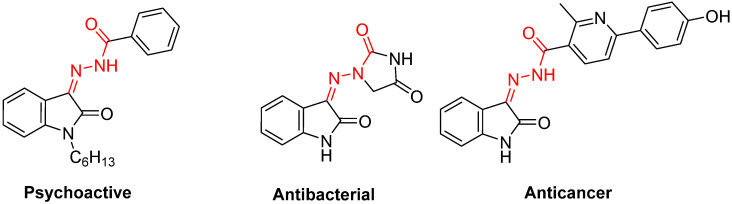
Representatives of isatin acylhydrazones with different bioactivities.

One of the most popular approaches to the creation of innovative drugs is the construction of hybrid molecules by decorating the basic chemical scaffold with various pharmacophoric fragments carrying a certain functional load. Isatin (indoline-2,3-dione) is a convenient platform for creating a large series of compounds with practically useful properties. Isatin is mainly used in medicinal chemistry since its derivatives have a wide spectrum of biological activity [1,2,3,4,5,6,7]. The convenience of using isatin in the synthesis of new compounds is due to the high reactivity of the carbonyl group at position 3 of the heterocycle. In this regard, the interest of researchers is focused on the production of spirocycles [8,9,10,11,12], idene derivatives [13,14], Schiff bases [15,16,17], etc. Thus, isatin-3-hydrazones exhibit antimicrobial [18], neuroprotective [19,20], psychoactive [21], anticancer [22], and other activities (Figure 1).

**Figure 2:**
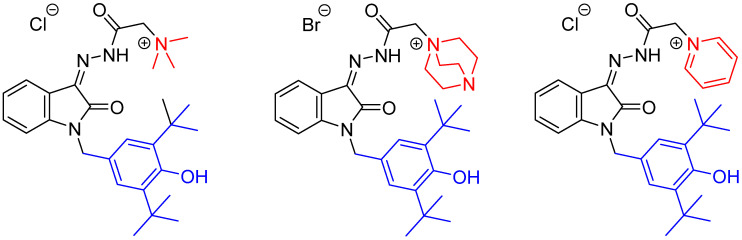
Previously published phenolic isatin hydrazones with antimicrobial activity [25,26,27].

On the other hand, sterically hindered phenols, which represent a class of known phenolic antioxidants, slow down the processes of lipid peroxidation and reduce oxidative stress in the organism [23,24]. These are promising components of drugs with various spectrums of action due to the increased efficiency of biologically active compounds. All of the above indicates the relevance of creating phenolic isatin-3-hydrazones hybrid molecular structures and conducting a comprehensive study of the biological activity of potential drug candidates. Using a structural hybridization approach, we previously obtained a series of hybrid isatin derivatives containing an antioxidant phenolic moiety, an isatin core, and an ammonium cation of varying hardness [25,26,27] (Figure 2).

In this paper, we describe the synthesis of a series of new water-soluble hydrazones based on sterically hindered phenolic isatin containing an ammonium fragment of various structures, with the aim of finding agents with a broad spectrum of physiological action.

## 2. Results and Discussion

### 2.1. Chemistry

#### Synthesis of Ammonium Isatin-3-acylhydrazones

**Scheme 1:**
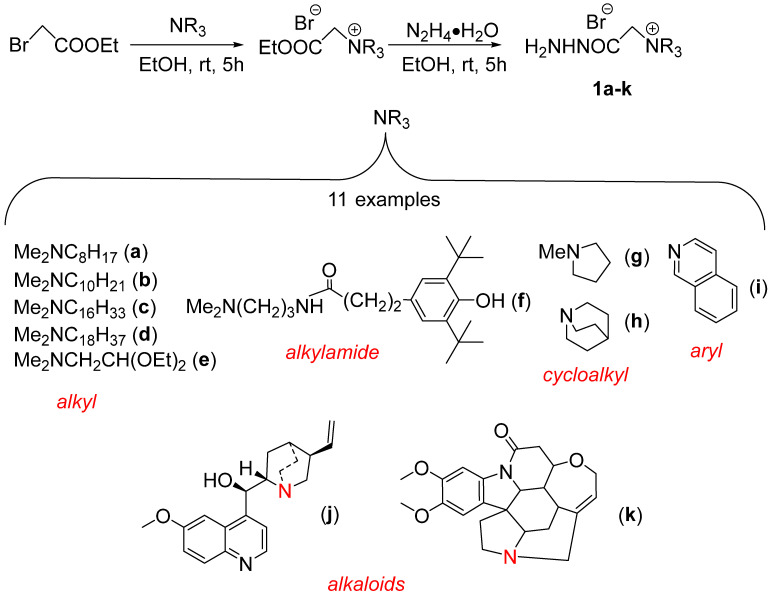
Two-step synthesis of ammonium acetohydrazides.

Ammonium acetohydrazides **1a–k** were synthesized by the quaternization reaction of certain tertiary amines with ethyl bromoacetate followed by hydrazinolysis of the corresponding esters (Scheme 1). In order to establish the influence of the structure of the ammonium fragment on the biological activity, the preparation of hydrazides **1j**, and **k**, based on the natural alkaloids quinine and brucine, was of particular interest.

Analogs of Girard’s reagents **1a–k** were isolated in moderate to high yields in pure form as air-stable white powders. Their structure and purity have been unequivocally proven by IR and NMR spectroscopy and elemental analysis data (Figures S1–S111, Supplementary Materials). The condensation reaction of ammonium hydrazides **1a–k** with 1-(3,5-di-*tert*-butyl-4-hydroxybenzyl)isatin **2** made it possible to obtain the target acylhydrazones in high yields (Scheme 2).

Compounds **3a–k** were isolated in high yields (72–90%) after an easy work-up of the reaction mixture. They are yellow powders, soluble in water, DMSO, and DMF to varying degrees. Trialkylammonium derivatives are also soluble in chloroform.

**Scheme 3:**
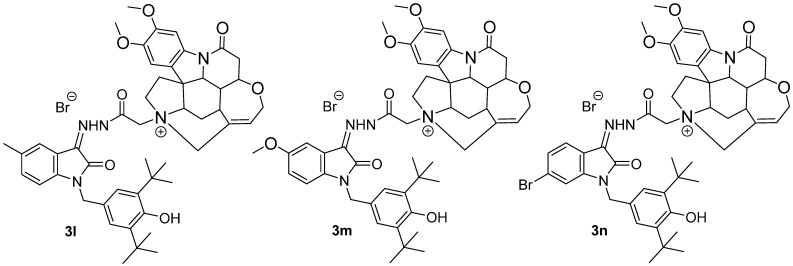
New isatin-3-acylhydrazones based on brucine alkaloid.

Currently, there is growing interest in ammonium salts based on natural compounds [28]. In this regard, under the same conditions, we obtained compounds **3l–n**, based on brucine hydrazide **1k**, containing substituents of various natures in the aromatic part of the oxindole (Scheme 3).

Thus, using simple and convenient synthetic procedures, we obtained various ammonium-charged isatin derivatives to establish their biological activity.

### 2.2. Biological Studies

A wide range of biological activity was investigated for the newly synthesized phenolic isatin-3-hydrazones to understand the further therapeutic vector of these substances. The studies included experiments to determine antioxidant status; influence on the blood clotting system; and cytotoxic, hemotoxic, and antimicrobial activities. Next, we will dwell in detail on each type of property, and at the end, we will summarize which modification leads to the best bioeffect.

#### 2.2.1. Antimicrobial Activity

Due to the fact that the widespread incidence of infections caused by resistant bacteria is a global health problem throughout the world [29,30], an urgent area of modern medical chemistry is the search for molecules with antimicrobial activity. It has been shown that ammonium isatin-3-hydrazones can exhibit high antimicrobial activity against gram-positive bacteria (*Staphylococcus aureus*, *Bacillus cereus*, and *Enterococcus faecalis*) [18], which cause dangerous infectious diseases in humans and animals [31,32,33,34]. The most outstanding results were demonstrated by compounds **3a**, **3b**, **3e**, **3f**, **3l**, and **3m**, whose MIC against *S. aureus* was 2–4 times higher than that of the reference drug norfloxacin (Table 1). It is important to note that these lead compounds were also effective against methicillin-resistant *S. aureus* strains. In addition, ammonium salt **3e**, containing an acetal fragment, showed activity against *B. cereus* and *E. faecalis* 4 and 2 times higher than the reference drug, respectively. The results obtained are of significant interest since the widespread incidence of infections caused by resistant bacteria is a global health problem throughout the world [29,30].

#### 2.2.2. Hemolytic and Cytotoxic Activity

**Figure 3:**
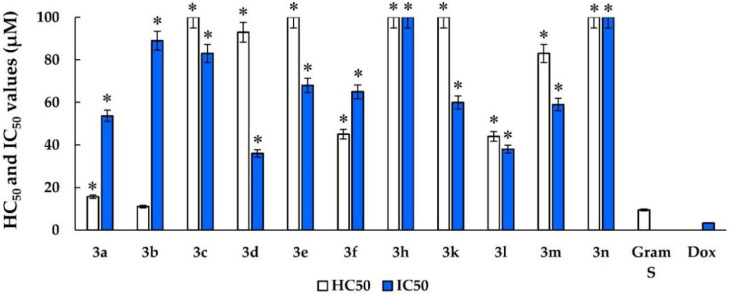
Hemotoxic and cytotoxic activity of **3a–3n**, expressed in terms of HC_50_ и IC_50_; * Values indicate *p* < 0.01.

An important parameter when studying the biological activity of new chemical compounds is their cytotoxic effect on mammalian cells [35]. The ability of the test compound to cause the destruction of human red blood cells illustrates its toxic effect on the internal environment of the body [36,37]. In this regard, test compounds were tested for cytotoxicity against red blood cells and the human hepatocyte-like cell line Chang liver (Figure 3).

Data on hemolytic and cytotoxic activity are presented as HC_50_ and IC_50_ values. Gramicidin S (Gram S) and doxorubicin (Dox) were used as comparison drugs to assess hemolytic properties and cytotoxicity, respectively. It can be seen that the most hemolytically active compound is **3b**, which exhibited an HC_50_ value close to gramicidin S. The hemolytic properties of the remaining compounds **3a**, and **3c–3n**, as well as the cytotoxic activity of the entire series of compounds **3a–3n**, are significantly different from the reference drugs. The selectivity index of the leading compounds against *Staphylococcus aureus* (IC_50_/MIC) ranged from 4.8 to 28. The *n*-octyl analog **3a** showed the greatest selectivity.

#### 2.2.3. Antioxidant Activity

It is well known that reactive oxygen species play one of the key roles in the genesis and progression of malignant neoplasms [38,39,40]. Thus, even a slight shift in the balance of highly active oxygen-containing molecules towards oxidative stress leads to the activation of a number of signaling pathways [38], leading to DNA damage [41] and, as a result, the induction of mutagenesis [42], a pathological change in the metabolic profile of tumor cells [43], namely, an offset towards the Warburg effect [44] as well as increased cell proliferation and migration [45]. In other words, all the above-mentioned processes contribute to the malignant transformation of cells and intensify oncogenesis. In this regard, the regulation of the level of reactive oxygen species by therapeutic agents capable of targeting and modulating the function of signaling molecules seems to be a promising therapeutic approach in the creation of antitumor drugs [46].

In our work, the study of the antioxidant potential of compounds was carried out by the ability to inhibit the process of Fe(II)-induced lipid peroxidation of rat brain homogenate. Due to the fact that all synthesized phenolic isatin-3-hydrazones containing a quaternary ammonium center showed pronounced antioxidant activity, we determined the IC_50_ values of the lipid peroxidation inhibiting effects (Table 2).

It should be noted that the ability to reduce the level of malondialdehyde, a marker of lipid peroxidation, of most compounds was higher than for the comparison drug Trolox, which may be due to the inclusion in the structure of molecules of more effective functional groups responsible for the manifestation of antioxidant properties.

As shown in Table 2, for 12 derivatives, these values ranged from 3 to 9 μM, which suggests a pronounced ability of these compounds to modulate processes associated with oxidative stress.

#### 2.2.4. Anticoagulant and Antiaggregation Activities

Cancer is characterized by a violation of the regulation of various biological systems physiologically involved in hemostasis [47,48].

For example, the results of current epidemiological studies demonstrate a 9-fold increase in the risk of venous thromboembolism compared with people without cancer [49,50,51,52]. That is why the presence of antiplatelet properties as a mechanism of action of potential antitumor agents is considered a promising approach to the development of therapeutic agents to combat oncopathologies.

In this work, anticoagulant and antiaggregation properties were studied (Table 3).

The findings show that brucine and quinine derivatives **3l**, and **3j** exhibit antiaggregational activity exceeding the values of acetylsalicylic acid (13.7 vs. 20.5 for **3l** and 20.7 for **3j** at *p* < 0.05). Compounds **3b**, **3c**, **3e**, **3g**, **3f**, and **3l** have an antiplatelet effect at the level of acetylsalicylic acid. However, one should note that compounds **3b**, **3c**, **3e,** and **3f**, in addition to antiaggregational activity, lengthen the lag period, which characterizes the process of release of endogenous agonists of aggregation from platelets. This effect is absent in acetylsalicylic acid, which indicates a potentially wide antithrombotic potential of the studied compounds. With respect to the coagulation link of hemostasis, these compounds showed an effect exclusively on the APTT index. It should be noted that the results of APTT elongation different from the control were recorded in compounds **3c**, **3e**, **3g**, **3i**, and **3k**. Compound **3l** extended the APTT value similarly to sodium heparin (19.2 vs. 20.3 at *p* < 0.05). Therefore, the resulting compounds have a high potential as a scaffold for the development of effective anticoagulant and antiaggregation agents.

Thus, the obtained phenolic isatin-3-hydrazones have a high potential as a basis for the development of potential drugs due to the presence of a set of positive biological properties. So, most compounds have antimicrobial effects and have also shown anticoagulant, antiplatelet, and antioxidant properties that can help to avoid systemic toxicity to the body as a whole.

**Figure 4:**
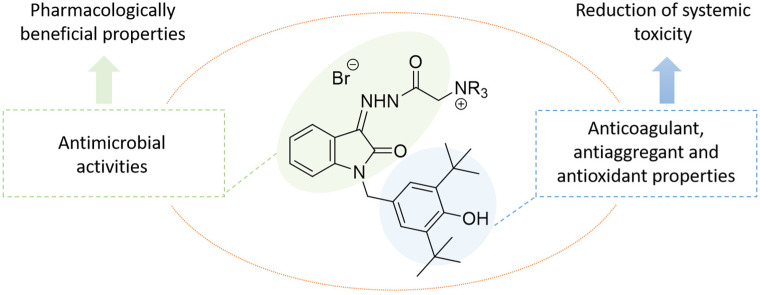
Summary of biological activity data of phenolic isatin-3-hydrazones.

Thus, all the obtained results on the biological activity of the synthesized compounds can lay the groundwork for future pharmaceutical developments for the creation of effective antibacterial drugs on their basis with reduced systemic toxicity due to the presence of antioxidant properties (Figure 4).

## 3. Materials and Methods

### 3.1. Chemistry

IR spectra were recorded on an IR Fourier spectrometer Tensor 37 (Bruker Optik GmbH, Ettlingen, Germany) in the 400–3600 cm^−1^ range in KBr. The ^1^H- and ^13^C-NMR spectra were recorded on a Bruker AVANCE 400 spectrometer (Bruker BioSpin, Rheinstetten, Germany) operating at 400 MHz (for ^1^H NMR) and 101 MHz (for ^13^C NMR), a Brucker spectrometer AVANCE*III*-500 (Bruker BioSpin, Rheinstetten, Germany) operating at 500 MHz (for ^1^H NMR) and 126 MHz (for ^13^C MMR), and a Bruker AVANCE 600 spectrometer (Bruker BioSpin, Rheinstetten, Germany) operating at 600 MHz (for ^1^H NMR) and 151 MHz (for ^13^C NMR). Chemical shifts were measured in δ (ppm) with reference to the solvent (δ = 7.26 ppm and 77.00 ppm for CDCl_3_, δ = 2.50 ppm and 39.50 ppm for DMSO-*d*_6_, for ^1^H and ^13^C NMR, respectively). Mass spectra ESI and MALDI were obtained on AmazonX (Bremen, Bruker, Germany) and UltraFlex III TOF/TOF (Bremen, Bruker, Germany) spectrometers, respectively. Elemental analysis was performed on a CHNS-O Elemental Analyser EuroEA3028-HT-OM (EuroVector S.p.A., Milan, Italy). The melting points were determined on the Stuart SMP10 apparatus (Birmingham, UK).

**Synthesis of ammonium hydrazides 1a–k (general method)**. In total, 0.53 g (3.2 mmol) of ethyl bromoacetate was added to a solution of 2.8 mmol of corresponding tertiary amine in 10 mL of ethanol. The solution was stirred at room temperature for 5 h and left overnight. After rotary removal of the solvent, the viscous residue was washed with diethyl ether (5 × 10 mL) and dried in a vacuum. The resulting intermediate product was dissolved in 10 mL of ethanol, and 0.6 g (12 mmol) of hydrazine hydrate (80% aqueous solution) was added. The reaction mass was stirred at room temperature for 5 h and left overnight. Then, volatile substances were removed in a vacuum. The residue was washed with diethyl ether and dried to form white powders.

***N*-(2-Hydrazinyl-2-oxoethyl)-*N,N*-dimethyloctan-1-ammonium bromide (1a)**. White powder. Yield 85%, m.p. = 120–122 °C. IR spectrum, ν, cm^−1^: 1694 (C=O), 2955 (CH), 3135 (NH), 3438 (NH). ^1^H NMR (400 MHz, CDCl_3_) δ 4.39 (s, 2H, CH_2_), 3.55–3.49 (m, 2H, CH_2_), 3.30 (s, 6H, CH_3_), 1.71–1.62 (m, 2H, CH_2_), 1.21–1.13 (m, 10H, CH_2_), 0.72–0.76 (m, 3H, CH_3_). ^13^C NMR (101 MHz, CDCl_3_) δ 162.2, 65.8 (CH_2_), 62.3 (CH_2_), 51.8 (CH_3_), 31.3 (CH_2_), 28.8 (CH_2_), 28.7 (CH_2_), 26.0 (CH_2_), 22.2 (CH_2_), 13.7 (CH_3_). MS (MALDI): *m*/*z* = 230.2 [M-Br]^+^; Found: C, 46.35; H, 9.01; N, 13.37. Anal. calcd (%) for C_12_H_28_BrN_3_O: C, 46.45; H, 9.10; N, 13.54.

***N*-(2-Hydrazinyl-2-oxoethyl)-*N,N*-dimethyldecan-1-ammonium bromide (1b)**. White powder. Yield 70%, m.p. = 150–152 °C. IR spectrum, ν, cm^−1^: 1693 (C=O), 2927 (CH), 3151 (NH), 3393 (NH). ^1^H NMR (600 MHz, CDCl_3_) δ 4.48 (s, 2H, CH_2_), 3.57–3.54 (m, 2H, CH_2_), 3.35 (s, 6H, CH_3_), 1.72–1.68 (m, 2H, CH_2_), 1.27–1.23 (m, 4H, CH_2_), 1.19–1.16 (m, 10H, CH_2_), 0.77 (t, *J* = 7.1 Hz, 3H, CH_3_). ^13^C NMR (151 MHz, CDCl_3_) δ 162.2, 65.9 (CH_2_), 62.3 (CH_2_), 51.8 (CH_3_), 31.6 (CH_2_), 29.20 (CH_2_), 29.19 (CH_2_), 28.99 (CH_2_), 28.96 (CH_2_), 26.0 (CH_2_), 22.7 (CH_2_), 22.4 (CH_2_), 13.8 (CH_3_). MS (MALDI): *m*/*z* = 258.1 [M-Br]^+^; Found: C, 49.56; H, 9.42; N, 12.30. Anal. calcd (%) for C_14_H_32_BrN_3_O: C, 49.70; H, 9.53; N, 12.42.

***N*-(2-Hydrazinyl-2-oxoethyl)-*N,N*-dimethylhexadecan-1-ammonium bromide (1c)**. White powder. Yield 89%, m.p. = 167–168 °C. IR spectrum, ν, cm^−1^: 1679 (C=O), 2920 (CH), 3197 (NH), 3323 (NH). ^1^H NMR (400 MHz, DMSO-*d*_6_) δ 4.55 (br. s, 1H, NH), 4.03 (s, 2H, CH_2_), 3.45–3.42 (m, 2H, CH_2_), 3.17 (s, 6H, CH_3_), 1.72–1.66 (m, 2H, CH_2_), 1.26–1.23 (m, 26H, CH_2_), 0.85 (t, *J* = 7.1 Hz, 3H, CH_3_). ^13^C NMR (101 MHz, DMSO-*d*_6_) δ 161.8, 64.7 (CH_2_), 60.9 (CH_2_), 51.1 (CH_3_), 31.2 (CH_2_), 28.98 (CH_2_), 28.96 (CH_2_), 28.94 (CH_2_), 28.9 (CH_2_), 28.7 (CH_2_), 28.6 (CH_2_), 28.4 (CH_2_), 25.7 (CH_2_), 22.0 (CH_2_), 21.8 (CH_2_), 13.9 (CH_3_). MS (MALDI): *m*/*z* = 342.4 [M-Br]^+^; Found: C, 56.72; H, 10.38; N, 9.86. Anal. calcd (%) for C_20_H_44_BrN_3_O: C, 56.86; H, 10.50; N, 9.95.

***N*-(2-Hydrazinyl-2-oxoethyl)-*N,N*-dimethyloctadecan-1-ammonium bromide (1d)**. White powder. Yield 92%, m.p. = 171–172 °C. IR spectrum, ν, cm^−1^: 1699 (C=O), 2960 (CH), 3136 (NH), 3433 (NH). ^1^H NMR (400 MHz, DMSO-*d*_6_) δ 9.72 (s, 1H, NH), 3.99 (s, 2H, CH_2_), 3.43–3.40 (m, 2H, CH_2_), 3.16 (s, 6H, CH_3_), 1.70–1.64 (m, 2H, CH_2_), 1.19–1.16 (m, 30H, CH_2_), 0.85 (t, *J* = 7.0 Hz, 3H, CH_3_). ^13^C NMR (101 MHz, DMSO-*d*_6_) δ 161.8, 64.7 (CH_2_), 60.9 (CH_2_), 51.1 (CH_3_), 31.2 (CH_2_), 28.96 (CH_2_), 28.92 (CH_2_), 28.88 (CH_2_), 28.7 (CH_2_), 28.6 (CH_2_), 28.4 (CH_2_), 25.7 (CH_2_), 22.0 (CH_2_), 21.8 (CH_2_), 13.9 (CH_3_). MS (MALDI): *m*/*z* = 370.4 [M-Br]^+^; Found: C, 58.40; H, 10.67; N, 9.24. Anal. calcd (%) for C_22_H_48_BrN_3_O: C, 58.65; H, 10.74; N, 9.33.

***N*-(2,2-Diethoxyethyl)-2-hydrazinyl-*N,N*-dimethyl-2-oxoethan-1-ammonium bromide (1e)**. White powder. Yield 96%, m.p. = 160–161 °C. IR spectrum, ν, cm^−1^: 1684 (C=O), 2979 (CH), 3230 (NH), 3445 (NH). ^1^H NMR (400 MHz, CDCl_3_) δ 5.05–5.02 (m, 1H, CH), 4.64 (s, 2H, CH_2_), 3.81–3.79 (m, 2H, CH_2_), 3.72–3.61 (m, 4H, CH_2_), 3.48 (s, 6H, CH_3_), 1.16 (t, *J* = 6.9 Hz, 6H, CH_3_). ^13^C NMR (101 MHz, CDCl_3_) δ 162.3, 97.5 (CH), 65.2 (CH_2_), 63.7 (CH_2_), 53.7 (CH_3_), 15.1 (CH_3_). MS (MALDI): *m*/*z* = 234.0 [M-Br]^+^; Found: C, 38.15; H, 7.61; N, 13.29. Anal. calcd (%) for C_10_H_24_BrN_3_O_3_: C, 38.22; H, 7.70; N, 13.37.

**3-(3-(3,5-Di-*tert*-butyl-4-hydroxyphenyl)propanamido)-*N*-(2-hydrazinyl-2-oxoethyl)-*N,N*-dimethylpropan-1-ammonium bromide (1f)**. White powder. Yield 59%, m.p. = 173–175 °C. IR spectrum, ν, cm^−1^: 1680 (C=O), 2943 (CH), 3200 (NH), 3313 (NH). ^1^H NMR (400 MHz, DMSO-*d*_6_) δ 8.06 (s, 1H, NH), 6.91 (s, 2H, ArH), 4.05 (s, 2H, CH_2_), 3.50–3.46 (m, 2H, CH_2_), 3.18 (s, 6H, CH_3_), 3.14–3.09 (m, 4H, CH_2_), 2.70 (t, *J* = 7.8 Hz, 2H, CH_2_), 2.33 (t, *J* = 7.8 Hz, 2H, CH_2_), 1.91–1.83 (m, 2H, CH_2_), 1.35 (s, 18H, CH_3_). ^13^C NMR (101 MHz, DMSO-*d*_6_) δ 172.0, 161.7, 151.9, 139.1, 132.1, 124.1 (CH), 64.8 (CH_2_), 63.0 (CH_2_), 61.1 (CH_2_), 51.2 (CH_3_), 37.7 (CH_2_), 35.4 (CH_2_), 34.4, 30.4 (CH_3_), 22.7 (CH_2_). MS (MALDI): *m*/*z* = 435.2 [M-Br]^+^; Found: C, 55.80; H, 8.37; N, 10.79. Anal. calcd (%) for C_24_H_43_BrN_4_O_3_: C, 55.92; H, 8.41; N, 10.87.

**1-(2-Hydrazinyl-2-oxoethyl)-1-methylpyrrolidin-1-ium bromide (1g)**. White powder. Yield 99%, m.p. = 140–142 °C. IR spectrum, ν, cm^−1^: 1668 (C=O), 2933 (CH), 3185 (NH), 3320 (NH). ^1^H NMR (400 MHz, DMSO-*d*_6_) δ 4.21 (s, 2H, CH_2_), 3.66–3.62 (m, 4H, CH_2_), 3.19 (s, 6H, CH_3_), 2.14–2.07 (m, 4H, CH_2_). ^13^C NMR (101 MHz, DMSO-*d*_6_) δ 162.3, 64.6 (CH_2_), 61.0 (CH_2_), 49.1 (CH_3_), 21.0 (CH_2_). MS (MALDI): *m*/*z* = 157.8 [M-Br]^+^; Found: C, 35.19; H, 6.70; N, 17.53. Anal. calcd (%) for C_7_H_16_BrN_3_O: C, 35.31; H, 6.77; N, 17.65.

**1-(2-Hydrazinyl-2-oxoethyl)quinuclidin-1-ium bromide (1h)**. White powder. Yield 85%, m.p. = 199–201 °C. IR spectrum, ν, cm^−1^: 1686 (C=O), 2947 (CH), 3144 (NH), 3355 (NH). ^1^H NMR (600 MHz, DMSO-*d*_6_) δ 3.98 (s, 2H, CH_2_), 3.63–3.60 (m, 6H, CH_2_), 1.90–1.84 (m, 6H, CH_2_), 1.78–1.75 (m, 1H, CH). ^13^C NMR (151 MHz, DMSO-*d*_6_) δ 161.4, 61.5 (CH_2_), 54.9 (CH_2_), 23.2 (CH_2_), 18.8 (CH). MS (MALDI): *m*/*z* = 183.8 [M-Br]^+^; Found: C, 40.80; H, 6.76; N, 15.82. Anal. calcd (%) for C_9_H_18_BrN_3_O: C, 40.92; H, 6.87; N, 15.91.

**1-(2-Hydrazinyl-2-oxoethyl)isoquinolin-1-ium bromide (1i)**. White powder. Yield 71%, m.p. = 203–205 °C. IR spectrum, ν, cm^−1^: 1650 (C=C), 1678 (C=O), 2925 (CH), 3037 (NH), 3197 (OH), 3442 (NH). ^1^H NMR (400 MHz, DMSO-*d*_6_) δ 10.08 (s, 1H, ArH), 9.20 (br. s, 1H, NH), 8.72–8.70 (m, 1H, ArH), 8.63–8.61 (m, 1H, ArH), 8.55–8.53 (m, 1H, ArH), 8.39–8.37 (m, 1H, ArH), 8.31–8.27 (m, 1H, ArH), 8.11–8.07 (m, 1H, ArH), 5.55 (s, 2H, CH_2_). ^13^C NMR (101 MHz, DMSO-*d*_6_) δ 163.7, 151.4 (CH), 137.3 (CH), 137.1 (CH), 136.0, 131.3 (CH), 130.5 (CH), 127.3, 126.8 (CH), 125.3 (CH), 60.3 (CH_2_). MS (MALDI): *m*/*z* = 201.8 [M-Br]^+^; Found: C, 46.76; H, 4.19; N, 14.71. Anal. calcd (%) for C_11_H_12_BrN_3_O: C, 46.83; H, 4.29; N, 14.89.

**(1*S*,2*S*,4*S*,5*R*)-1-(2-Hydrazinyl-2-oxoethyl)-2-((*R*)-hydroxy(6-methoxyquinolin-4-yl)methyl)-5-vinylquinuclidin-1-ium bromide (1j)**. White powder. Yield 90%, m.p. = 217–219 °C. IR spectrum, ν, cm^−1^: 1510 (C=N), 1621 (C=C), 1684 (C=O), 2929 (CH), 3251 (NH), 3418 (br. s., NH, OH). ^1^H NMR (400 MHz, DMSO-*d*_6_) δ 8.82 (d, *J* = 4.6 Hz, 1H, ArH), 7.98 (d, *J* = 9.9 Hz, 1H, ArH), 7.78 (d, *J* = 4.6 Hz, 1H, ArH), 7.43 (dd, *J* = 9.9 Hz, *J* = 2.4 Hz, 2ArH), 6.75 (d, *J* = 3.5 Hz, 1H, OH), 6.05 (br s, *J* = 3.5 Hz, 1H, CH), 5.75 (ddd, *J* = 5.3 Hz, *J* = 10.5 Hz, *J* = 17.5 Hz, 1H, CH=), 5.23 (d, *J* = 17.5 Hz, 1H, *trans*-CH_2_=), 5.03 (d, *J* = 10.5 Hz, 1H, *cis*-CH_2_=), 4.46–4.84 (m, 3H, 2CH_2_, CH), 4.31 (br. s, 2H, CH_2_), 4.13 (s, 3H, CH_3_), 3.65 (m, 1H, CH), 2.90 (m, 1H, CH), 1.91–2.03 (m, 2H, CH_2_), 1.06 (m, 2H, CH_2_). ^13^C NMR (101 MHz, DMSO-*d*_6_) δ 163.0, 158.0, 147.2 (CH), 143.7, 143.5, 138.1 (CH), 131.2 (CH), 125.5, 122.1 (CH), 120.2 (CH), 115.6 (CH_2_), 101.5 (CH), 65.5 (CH), 63.6 (CH), 60.3 (CH_2_), 57.9 (CH_2_), 56.6 (CH), 56.5 (CH_2_), 36.7 (CH_3_), 25.3 (CH_2_), 24.8 (CH), 21.3 (CH). MS (ESI): *m*/*z* = 397.3 [M-Br]^+^; Found: C, 55.21; H, 6.00; N, 11.60. Anal. calcd (%) for C_22_H_29_BrN_4_O_3_: C, 55.35; H, 6.12; N, 11.74.

**6-(2-Hydrazinyl-2-oxoethyl)-10,11-dimethoxy-14-oxo-4a,4a^1^,5,5a,6,7,8,8a^1^,15,15a-decahydro-2*H*,14*H*-4,6-methanoindolo[3,2,1-*ij*]oxepino[2,3,4-*de*]pyrrolo[2,3-*h*]quinolin-6-ium bromide (1k)**. White powder. Yield 76%, m.p. = 257–259 °C. IR spectrum, ν, cm^−1^: 1462 (C-N), 1504 (C-O), 1651 (C=O), 1691 (C=O), 2890 (CH), 2939 (CH), 3241 (NH), 3375 (NH). ^1^H NMR (400 MHz, DMSO-*d*_6_) δ 10.04 (br. s, 1H, NH), 7.64 (s, 1H, ArH); 7.28 (s, 1H, ArH); 6.40 (m, 1H, =CH); 4.70 (br. s, 1H, CH); 4.30–4.44 (m, 4H, CH_2_, OCH_2_); 4.13–4.22 (m, 4H, 2CH_2_); 4.07–4.09 (m, 1H, OCH); 3.80 (s, 3H, OCH_3_); 3.74 (s, 3H, OCH_3_); 2.61–2.69 (m, 2H, CH_2_); 2.13–2.16 (m, 1H, CH); 1.66–1.68 (m, 1H, CH), 1.47–1.46 (m, 1H, CH). ^13^C NMR (101 MHz, DMSO-*d*_6_) δ 168.5, 161.7; 149.7; 146.0; 136.1 (CH); 135.5; 132.5; 120.1; 107.6 (CH); 100.4 (CH); 75.9 (CH); 73.6 (CH); 63.3 (CH_2_); 63.2 (CH_2_); 62.4 (CH_2_); 59.6 (CH_2_); 58.6 (CH); 56.4 (CH_3_); 55.7 (CH_3_); 51.9; 46.2 (CH); 38.6 (CH_2_); 28.9 (CH); 24.4 (CH_2_). Found: C, 54.70; H, 5.60; N, 10.11. Anal. calcd (%) for C_25_H_31_BrN_4_O_5_: C, 54.85; H, 5.71; N, 10.23.

**Synthesis of ammonium isatin-3-acylhydrazones 3a–k (general method)**. To the mixture of 1-(3,5-di-*tert*-butyl-4-hydroxybenzyl)isatin **2** (10 mmol) and 15 mL of absolute ethanol, corresponding hydrazide **1a–k** (10 mmol) and three drops of trifluoroacetic acid were successively added. The reaction solution was heated under reflux for 3 h. After spontaneously cooling to room temperature, the precipitate formed was filtered, washed with absolute ether, and dried in a vacuum.

***N*-(2-(2-(1-(3,5-Di-*tert*-butyl-4-hydroxybenzyl)-2-oxoindolin-3-ylidene)hydrazinyl)-2-oxoethyl)-*N,N*-dimethyloctan-1-ammonium bromide (3a)**. Yellow powder. Yield 80%, m.p. = 203–204 °C. IR spectrum, ν, cm^−1^: 1617 (C=C), 1685 (C=O), 1699 (C=O), 2955 (CH), 3401 (NH), 3614 (OH). ^1^H NMR (600 MHz, CDCl_3_) δ 12.78 (s, 1H, NH), 7.91 (d, *J* = 7.3 Hz, 1H, Ar), 7.30 (dd, *J* = 7.8 Hz, *J* = 7.8 Hz, 1H, Ar), 7.10–7.07 (m, 3H, Ar), 6.84 (d, *J* = 7.8 Hz, 1H, Ar), 5.30 (s, 2H, CH_2_), 5.18 (s, 1H, OH), 4.75 (s, 2H, CH_2_), 3.90–3.85 (m, 2H, CH_2_), 3.73 (s, 6H, CH_3_), 1.77–1.70 (m, 2H, CH_2_), 1.37 (s, 18H, CH_3_), 1.22 (br. s, 10H, CH_2_), 0.83 (t, 3H, *J* = 7.1 Hz, CH_3_). ^13^C NMR (150 MHz, DMSO-*d*_6_) δ 166.1, 160.4, 153.3, 149.0, 143.4, 139.4, 132.2 (CH), 126.5, 124.1 (CH), 123.3 (CH), 121.0 (CH), 118.5, 110.8 (CH), 64.7 (CH_2_), 59.5 (CH_2_), 51.5 (CH_3_), 43.0 (CH_2_), 34.4, 31.1 (CH_2_), 30.2 (CH_3_), 28.33 (CH_2_), 28.29 (CH_2_), 25.6 (CH_2_), 22.0 (CH_2_), 21.8 (CH_2_), 13.9 (CH_3_). MS (MALDI): *m*/*z* = 577.4 [M-Br]^+^; Found: C, 63.80; H, 8.09; Br, 12.10; N, 8.48. Anal. calcd. (%) for C_35_H_53_BrN_4_O_3_: C, 63.91; H, 8.12; Br, 12.15; N, 8.52.

***N*-(2-(2-(1-(3,5-Di-*tert*-butyl-4-hydroxybenzyl)-2-oxoindolin-3-ylidene)hydrazinyl)-2-oxoethyl)-*N,N*-dimethyldecan-1-ammonium bromide (3b)**. Yellow powder. Yield 73%, m.p. = 233–235 °C. IR spectrum, ν, cm^−1^: 1613 (C=C), 1677 (C=O), 2926 (CH), 3400 (NH), 3642 (OH). ^1^H NMR (600 MHz, CDCl_3_) δ 12.84 (s, 1H, NH), 7.92 (d, *J* = 7.0 Hz, 1H, Ar), 7.33 (dd, *J* = 7.4 Hz, *J* = 7.7 Hz, 1H, Ar), 7.15–7.11 (m, 3H, Ar), 6.87 (d, *J* = 7.9 Hz, 1H, Ar), 5.31 (s, 2H, CH_2_), 5.20 (s, 1H, OH), 4.78 (s, 2H, CH_2_), 3.89–3.86 (m, 2H, CH_2_), 3.72 (s, 6H, CH_3_), 1.80–1.83 (m, 2H, CH_2_), 1.40 (s, 18H, CH_3_), 1.24 (br. s, 14H, CH_2_), 0.86 (t, 3H, *J* = 7.0 Hz, CH_3_). ^13^C NMR (150 MHz, CDCl_3_) δ 167.6, 161.0, 153.6, 143.7, 132.2 (CH), 125.4, 124.6 (CH), 124.5 (CH), 123.8 (CH), 123.0 (CH), 118.9, 110.0 (CH), 65.2 (CH_2_), 60.4 (CH_2_), 52.9 (CH_3_), 44.0 (CH_2_), 34.3, 31.8 (CH_2_), 30.2 (CH_3_), 29.34 (CH_2_), 29.32 (CH_2_), 29.2 (CH_2_), 29.1 (CH_2_), 26.2 (CH_2_), 23.1 (CH_2_), 22.6 (CH_2_), 14.0 (CH_3_). MS (MALDI): *m*/*z* = 605.7 [M-Br]^+^; Found: C, 64.73; H, 8.29; Br, 11.60; N, 8.12. Anal. calcd. (%) for C_37_H_57_BrN_4_O_3_: C, 64.80; H, 8.38; Br, 11.65; N, 8.17.

***N*-(2-(2-(1-(3,5-Di-*tert*-butyl-4-hydroxybenzyl)-2-oxoindolin-3-ylidene)hydrazinyl)-2-oxoethyl)-*N,N*-dimethylhexadecan-1-ammonium bromide (3c)**. Yellow powder. Yield 91%, m.p. = 257–259 °C. IR spectrum, ν, cm^−1^: 1617 (C=C), 1685 (C=O), 2924 (CH), 3211 (NH), 3392 (NH), 3640 (OH). ^1^H NMR (400 MHz, CDCl_3_) δ 12.78 (s, 1H, NH), 7.92 (d, *J* = 7.4 Hz, 1H, Ar), 7.30 (dd, *J* = 7.9 Hz, *J* = 7.7 Hz, 1H, Ar), 7.10–7.04 (m, 3H, Ar), 6.83 (d, *J* = 7.9 Hz, 1H, Ar), 5.29 (s, 2H, CH_2_), 5.19 (s, 1H, OH), 4.74 (s, 2H, CH_2_), 3.88–3.84 (m, 2H, CH_2_), 3.73 (s, 6H, CH_3_), 1.81–1.71 (m, 2H, CH_2_), 1.37 (s, 18H, CH_3_), 1.23–1.21 (m, 22H, CH_2_), 0.85 (t, 3H, *J* = 7.0 Hz, CH_3_). ^13^C NMR (150 MHz, CDCl_3_) δ 165.7, 160.9, 153.5, 143.5, 136.4, 136.2, 132.0 (CH), 125.3, 124.5 (CH), 123.7 (CH), 122.8 (CH), 118.9, 109.8 (CH), 64.9 (CH_2_), 60.2 (CH_2_), 52.8 (CH_3_), 43.8 (CH_2_), 34.2, 31.8 (CH_2_), 30.1 (CH_3_), 29.6 (CH_2_), 29.53 (CH_2_), 29.46 (CH_2_), 29.32 (CH_2_), 29.2 (CH_2_), 26.1 (CH_2_), 23.0 (CH_2_), 22.6 (CH_2_), 14.0 (CH_3_). MS (MALDI): *m*/*z* = 690.0 [M-Br]^+^; Found: C, 67.00; H, 8.95; Br, 10.25; N, 7.22. Anal. calcd. (%) for C_43_H_69_BrN_4_O_3_: C, 67.08; H, 9.03; Br, 10.38; N, 7.28.

***N*-(2-(2-(1-(3,5-Di-*tert*-butyl-4-hydroxybenzyl)-2-oxoindolin-3-ylidene)hydrazinyl)-2-oxoethyl)-*N,N*-dimethyloctadecan-1-ammonium bromide (3d)**. Yellow powder. Yield 95%, m.p. = 264–265 °C. IR spectrum, ν, cm^−1^: 1617 (C=C), 1685 (C=O), 2924 (CH), 3211 (NH), 3368 (NH), 3640 (OH). ^1^H NMR (400 MHz, CDCl_3_) δ 12.82 (s, 1H, NH), 7.93 (d, *J* = 7.7 Hz, 1H, Ar), 7.32 (dd, *J* = 7.9 Hz, *J* = 7.7 Hz, 1H, Ar), 7.12–7.07 (m, 3H, Ar), 6.86 (d, *J* = 7.9 Hz, 1H, Ar), 5.32 (s, 2H, CH_2_), 5.21 (s, 1H, OH), 4.77 (s, 2H, CH_2_), 3.89–3.86 (m, 2H, CH_2_), 3.73 (s, 6H, CH_3_), 1.81–1.71 (m, 2H, CH_2_), 1.39 (s, 18H, CH_3_), 1.24 (br. s, 28H, CH_2_), 0.87 (t, 3H, *J* = 6.9 Hz, CH_3_). ^13^C NMR (100 MHz, DMSO-*d*_6_) δ 169.4, 160.1, 153.3, 143.4, 139.4, 135.0, 132.2 (CH), 126.4, 124.1 (CH), 123.2 (CH), 121.0 (CH), 118.7, 110.7 (CH), 64.4 (CH_2_), 59.4 (CH_2_), 51.4 (CH_3_), 43.0 (CH_2_), 34.4, 31.2 (CH_2_), 30.2 (CH_3_), 29.0 (CH_2_), 28.93 (CH_2_), 28.86 (CH_2_), 28.7 (CH_2_), 28.6 (CH_2_), 28.3 (CH_2_), 25.6 (CH_2_), 22.0 (CH_2_), 21.8 (CH_2_), 13.8 (CH_3_). MS (MALDI): *m*/*z* = 717.9 [M-Br]^+^; Found: C, 67.70; H, 9.15; Br, 9.82; N, 6.93. Anal. calcd. (%) for C_45_H_73_BrN_4_O_3_: C, 67.73; H, 9.22; Br, 10.01; N, 7.02.

**2-(2-(1-(3,5-Di-*tert*-butyl-4-hydroxybenzyl)-2-oxoindolin-3-ylidene)hydrazinyl)-*N*-(2,2-diethoxyethyl)-*N,N*-dimethyl-2-oxoethan-1-ammonium bromide (3e)**. Yellow powder. Yield 87%, m.p. = 183–184 °C. IR spectrum, ν, cm^−1^: 1615 (C=C), 1685 (C=O), 1718 (C=O), 2970 (CH), 3401 (NH), 3577 (OH). ^1^H NMR (400 MHz, CDCl_3_) δ 12.76 (s, 1H, NH), 7.78 (d, *J* = 7.4 Hz, 1H, Ar), 7.32 (dd, *J* = 7.9 Hz, *J* = 7.4 Hz, 1H, Ar), 7.10–7.06 (m, 3H, Ar), 6.87 (d, *J* = 7.6 Hz, 1H, Ar), 5.29 (s, 2H, CH_2_), 5.19 (s, 1H, OH), 4.96 (s, 1H, CH), 4.78 (s, 2H, CH_2_), 4.12 (m, 2H, CH_2_), 3.89 (s, 6H, CH_3_), 3.78–3.63 (m, 4H, CH_2_), 1.38 (s, 18H, CH_3_), 1.17 (t, 6H, *J* = 6.9 Hz, CH_3_). ^13^C NMR (100 MHz, DMSO-*d*_6_) δ 166.3, 160.4, 153.3, 143.4, 139.4, 134.8, 132.2 (CH), 126.5, 124.1 (CH), 123.3 (CH), 120.8 (CH), 118.4, 110.9 (CH), 96.5 (CH), 63.8 (CH_2_), 62.4 (CH_2_), 60.6 (CH_2_), 53.5 (CH_3_), 42.9 (CH_2_), 34.4, 30.2 (CH_3_), 14.9 (CH_3_). MS (MALDI): *m*/*z* = 581.5 [M-Br]^+^; Found: C, 59.80; H, 7.39; Br, 12.00; N, 8.39. Anal. calcd. (%) for C_33_H_49_BrN_4_O_5_: C, 59.90; H, 7.46; Br, 12.08; N, 8.47.

***N*-(2-(2-(1-(3,5-Di-*tert*-butyl-4-hydroxybenzyl)-2-oxoindolin-3-ylidene)hydrazinyl)-2-oxoethyl)-3-(3-(3,5-di-*tert*-butyl-4-hydroxyphenyl)propanamido)-*N,N*-dimethylpropan-1-ammonium bromide (3f)**. Yellow powder. Yield 92%, m.p. = 234–236 °C. IR spectrum, ν, cm^−1^: 1617 (C=C), 1688 (C=O), 2958 (CH), 3383 (NH), 3638 (OH). ^1^H NMR (400 MHz, CDCl_3_) δ 12.87 (s, 1H, NH), 7.80 (d, *J* = 7.3 Hz, 1H, Ar), 7.32 (dd, *J* = 8.1 Hz, *J* = 7.1 Hz, 1H, Ar), 7.13–7.04 (m, 3H, Ar), 7.00 (s, 2H, Ar), 6.87 (d, *J* = 7.8 Hz, 1H, Ar), 5.20 (s, 1H, OH), 4.99 (s, 2H, CH_2_), 4.77 (s, 2H, CH_2_), 4.10–4.06 (m, 2H, CH_2_), 3.53 (s, 6H, CH_3_), 3.40–3.33 (m, 4H, CH_2_), 2.87–2.83 (m, 2H, CH_2_), 2.58–2.54 (m, 2H, CH_2_), 1.39 (s, 36H, CH_3_). ^13^C NMR (100 MHz, CDCl_3_) δ 174.0, 165.1, 160.9, 153.6, 152.0, 143.7, 136.6, 136.5, 135.8, 131.5 (CH), 125.3, 124.9 (CH), 124.8, 124.6 (CH), 123.8 (CH), 122.5 (CH), 118.7, 110.1 (CH), 64.8 (CH_2_), 60.6 (CH_2_), 52.1 (CH_3_), 44.0 (CH_2_), 38.6 (CH_2_), 36.2 (CH_2_), 34.6 (CH_2_), 34.3, 30.3 (CH_3_), 30.1 (CH_3_), 29.4 (CH_2_). MS (MALDI): *m*/*z* = 783.0 [M-Br]^+^; Found: C, 65.30; H, 7.81; Br, 9.13; N, 8.01. Anal. calcd. (%) for C_47_H_68_BrN_5_O_5_: C, 65.41; H, 7.94; Br, 9.26; N, 8.12.

**1-(2-(2-(1-(3,5-Di-*tert*-butyl-4-hydroxybenzyl)-2-oxoindolin-3-ylidene)hydrazinyl)-2-oxoethyl)-1-methylpyrrolidin-1-ium bromide (3g)**. Yellow powder. Yield 79%, m.p. = 226–227 °C. IR spectrum, ν, cm^−1^: 1617 (C=C), 1681 (C=O), 2958 (CH), 3201 (NH), 3375 (NH), 3622 (OH). ^1^H NMR (400 MHz, CDCl_3_) δ 12.90 (s, 1H, NH), 8.03 (d, *J* = 7.3 Hz, 1H, Ar), 7.45 (dd, *J* = 7.6 Hz, *J* = 7.1 Hz, 1H, Ar), 7.26–7.21 (m, 3H, Ar), 7.00 (d, *J* = 7.8 Hz, 1H, Ar), 5.64 (s, 2H, CH_2_), 5.37 (s, 1H, OH), 4.91 (s, 2H, CH_2_), 4.40–4.30 (m, 4H, CH_2_), 3.67 (s, 3H, CH_3_), 2.50–2.38 (m, 4H, CH_2_), 1.54 (s, 18H, CH_3_). ^13^C NMR (100 MHz, CDCl_3_) δ 166.2, 160.7, 153.4, 143.3, 136.3, 135.8, 131.8 (CH), 125.2, 124.4 (CH), 123.4 (CH), 122.5 (CH), 118.8, 109.8 (CH), 65.7 (CH_2_), 62.0 (CH_2_), 49.5 (CH_3_), 43.7 (CH_2_), 34.1, 30.0 (CH_3_), 21.3 (CH_3_). MS (ESI): *m*/*z* = 505.5 [M-Br]^+^; Found: C, 61.43; H, 7.00; Br, 13.50; N, 9.48. Anal. calcd. (%) for C_30_H_41_BrN_4_O_3_: C, 61.53; H, 7.06; Br, 13.65; N, 9.57.

**1-(2-(2-(1-(3,5-Di-*tert*-butyl-4-hydroxybenzyl)-2-oxoindolin-3-ylidene)hydrazinyl)-2-oxoethyl)quinuclidin-1-ium bromide (3h)**. Yellow powder. Yield 88%, m.p. = 195–196 °C. IR spectrum, ν, cm^−1^: 1612 (C=C), 1686 (C=O), 2953 (CH), 3216 (NH), 3350 (NH), 3637 (OH). ^1^H NMR (400 MHz, CDCl_3_) δ 12.74 (s, 1H, NH), 7.91 (d, *J* = 7.6 Hz, 1H, Ar), 7.30 (dd, *J* = 8.0 Hz, *J* = 7.7 Hz, 1H, Ar), 7.11–7.06 (m, 3H, Ar), 6.82 (d, *J* = 7.9 Hz, 1H, Ar), 5.21–5.17 (m, 3H, CH_2_, OH), 4.74 (s, 2H, CH_2_), 4.21–4.18 (m, 6H, CH_2_), 2.25–2.22 (m, 1H, CH), 2.09–2.05 (m, 6H, CH_2_), 1.38 (s, 18H, CH_3_). ^13^C NMR (100 MHz, CDCl_3_) δ 165.8, 160.8, 153.5, 143.4, 138.1, 136.4, 131.9 (CH), 125.2 (CH), 124.5 (CH), 123.5 (CH), 122.6, 118.9, 110.8 (CH), 65.7 (CH_2_), 55.6 (CH_2_), 46.3 (CH_2_), 34.1, 30.4 (CH_3_), 22.8 (CH_2_), 19.4 (CH_3_). MS (MALDI): *m*/*z* = 531.4 [M-Br]^+^; Found: C, 66.30; H, 8.79; Br, 10.68; N, 7.43. Anal. calcd. (%) for C_41_H_65_BrN_4_O_3_: C, 66.38; H, 8.83; Br, 10.77; N, 7.55.

**2-(2-(2-(1-(3,5-Di-*tert*-butyl-4-hydroxybenzyl)-2-oxoindolin-3-ylidene)hydrazinyl)-2-oxoethyl)isoquinolin-2-ium bromide (3i)**. Yellow powder. Yield 91%, m.p. = 267–268 °C. IR spectrum, ν, cm^−1^: 1605 (C=C), 1679 (C=O), 2947 (CH), 3185 (NH), 3407 (NH), 3625 (OH). ^1^H NMR (400 MHz, DMSO-*d*_6_) δ 12.83 (s, 1H, NH), 10.14 (s, 1H, Ar), 8.82 (d, *J* = 6.8 Hz, 1H, Ar), 8.69 (d, *J* = 6.7 Hz, 1H, Ar), 8.56 (d, *J* = 8.3 Hz, 1H, Ar), 8.42 (d, *J* = 8.3 Hz, 1H, Ar), 8.33 (dd, *J* = 8.1 Hz, *J* = 7.1 Hz, 1H, Ar), 8.12 (dd, *J* = 8.0 Hz, *J* = 7.3 Hz, 1H, Ar), 7.68 (d, *J* = 7.1 Hz, 1H, Ar), 7.50 (dd, *J* = 8.0 Hz, *J* = 6.9 Hz, 1H, Ar), 7.28 (d, *J* = 7.5 Hz, 1H, Ar), 7.23–7.20 (m, 1H, Ar), 6.97 (s, 1H, OH), 6.36 (s, 2H, CH_2_), 4.91 (s, 2H, CH_2_), 1.34 (s, 18H, CH_3_). ^13^C NMR (100 MHz, DMSO-*d*_6_) δ 167.7, 160.5, 153.4, 152.0 (CH), 143.4, 139.5, 137.6, 137.3 (CH), 136.4 (CH), 135.1, 132.2 (CH), 131.4 (CH), 130.6 (CH), 127.4 (CH), 126.7, 126.5, 125.3 (CH), 124.1 (CH), 123.4 (CH), 120.7 (CH), 118.6, 110.9 (CH), 60.9 (CH_2_), 43.0 (CH_2_), 34.4, 30.2 (CH_3_). MS (ESI): *m*/*z* = 549.5 [M-Br]^+^; Found: C, 64.71; H, 8.30; Br, 11.51; N, 8.01. Anal. calcd. (%) for C_34_H_37_BrN_4_O_3_: C, 64.80; H, 8.38; Br, 11.65; N, 8.17.

**(1*S*,2*S*,4*S*,5*R*)-1-(2-(2-(1-(3,5-Di-*tert*-butyl-4-hydroxybenzyl)-2-oxoindolin-3-ylidene)hydrazinyl)-2-oxoethyl)-2-((*R*)-hydroxy-(6-methoxyquinolin-4-yl)methyl)-5-vinylquinuclidin-1-ium bromide (3j)**. Yellow powder. Yield 87%, m.p. = 203–205 °C. IR spectrum, ν, cm^−1^: 1618 (C=C), 1685 (C=O), 2956 (CH), 3197 (NH), 3398 (NH), 3630 (OH). ^1^H NMR (400 MHz, DMSO-*d*_6_) δ 12.91 (s, 1H, NH), 8.87 (d, *J* = 4.7 Hz, 1H, ArH), 8.02 (d, *J* = 9.2 Hz, 1H, ArH), 7.84 (d, *J* = 4.7 Hz, 1H, ArH), 7.52–7.48 (m, 3ArH), 7.34–7.26 (m, 2ArH), 7.21 (dd, *J* = 7.6 Hz, *J* = 7.6 Hz, 1H, ArH), 7.15–7.13 (m, 3H, 3ArH), 6.85–6.93 (m, 2H, ArH, CH=), 6.05 (br s, 1H, CH), 5.80–5.72 (m, 1H, CH=), 5.26 (s, 1H, OH), 5.22 (d, *J* = 17.1 Hz, 1H, *trans*-CH_2_=), 5.03 (d, *J* = 10.5 Hz, 1H, *cis*-CH_2_=), 4.89–4.84 (m, 3H, CH_2_, CH), 4.60 (br. s, 2H, CH_2_), 4.08 (s, 3H, CH_3_), 2.15 (br. s, 2H, CH_2_), 1.97–2.05 (m, 2H, CH_2_), 1.31 (br. s, 20H, 9CH_3_, CH_2_). ^13^C NMR (101 MHz, DMSO-*d*_6_) δ 167.2, 162.3, 158.5, 153.4, 147.5 (CH), 146.3, 143.5, 142.0, 139.5, 138.2 (CH), 132.4 (CH), 130.1 (CH), 127.0, 126.5 (CH), 125.6 (CH), 124.2 (CH), 123.3, 120.8 (CH), 120.5 (CH), 115.7 (CH_2_), 114.3, 110.8 (CH), 101.3 (CH), 65.4 (CH), 63.0 (CH), 60.5 (CH_2_), 57.4 (CH), 57.2 (CH_2_), 56.1 (CH), 43.1 (CH_2_), 36.7 (CH_3_), 35.7 (CH_3_), 34.4, 30.2 (CH_3_), 25.3 (CH_2_), 24.8 (CH), 21.2 (CH). MS (ESI): *m*/*z* = 745.2 [M-Br]^+^; Found: C, 65.40; H, 6.48; Br, 9.54; N, 8.38. Anal. calcd. (%) for C_45_H_54_BrN_5_O_5_: C, 65.53; H, 6.60; Br, 9.69; N, 8.49.

**6-(2-(2-(1-(3,5-Di-*tert*-butyl-4-hydroxybenzyl)-2-oxoindolin-3-ylidene)hydrazinyl)-2-oxoethyl)-10,11-dimethoxy-14-oxo-4a,4a^1^,5,5a,6,7,8,8a^1^,15,15a-decahydro-2*H*,14*H*-4,6-methanoindolo[3,2,1-*ij*]oxepino[2,3,4-*de*]pyrrolo[2,3-*h*]quinolin-6-ium bromide (3k)**. Light orange powder. Yield 64%, m.p. = 230–232 °C. IR spectrum, ν, cm^−1^: 1615 (C=C), 1685 (C=O), 2955 (CH), 3401 (NH), 3637 (OH). ^1^H NMR (400 MHz, CDCl_3_) δ 12.92 (s, 1H, NH), 8.30 (s, 1H, Ar), 7.75 (s, 1H, Ar), 7.53–7.49 (m, 1H, Ar), 7.30–7.26 (m, 1H, Ar), 7.14 (s, 1H, Ar), 7.09 (s, 2H, Ar), 6.99 (m, 1H, =CH), 6.80 (d, *J* = 7.8 Hz, 1H, Ar), 5.44 (s, 1H, OH), 4.79 (s, 2H, CH_2_), 4.71 (s, 2H, CH_2_), 4.66–4.62 (m, 2H, CH_2_), 4.33–4.32 (m, 2H, CH_2_), 4.26–4.22 (m, 1H, CH), 4.07–4.00 (m, 2H, CH_2_), 3.88 (s, 6H, CH_3_), 3.40–3.38 (m, 2H, CH_2_), 3.14–3.07 (m, 2H, CH_2_), 2.68–2.63 (m, 2H, CH_2_), 2.17–2.14 (m, 1H, CH), 1.81–1.77 (m, 1H, CH), 1.38 (s, 18H, CH_3_). ^13^C NMR (100 MHz, CDCl_3_) δ 168.5, 166.4, 160.9, 153.6, 150.4, 147.1, 143.5, 138.1, 137.2 (CH), 136.5, 135.4, 132.8, 132.1 (CH), 125.3, 124.6 (CH), 124.0 (CH), 118.6, 110.9 (CH), 109.7 (CH), 107.0 (CH), 100.7 (CH), 75.3 (CH), 65.0 (CH_2_), 64.1 (CH_2_), 61.0 (CH_2_), 59.4 (CH), 57.5 (CH), 56.2 (CH_3_), 52.9, 46.8 (CH), 44.3 (CH_2_), 43.9 (CH_2_), 41.8 (CH_2_), 39.9 (CH_2_), 34.3, 30.2 (CH_3_), 26.1 (CH_2_). MS (MALDI): *m*/*z* = 814.5 [M-Br]^+^; Found: C, 64.30; H, 6.20; Br, 8.74; N, 7.67. Anal. calcd. (%) for C_48_H_56_BrN_5_O_7_: C, 64.42; H, 6.31; Br, 8.93; N, 7.83.

**6-(2-(2-(1-(3,5-Di-*tert*-butyl-4-hydroxybenzyl)-5-methyl-2-oxoindolin-3-ylidene)hydrazinyl)-2-oxoethyl)-10,11-dimethoxy-14-oxo-4a,4a^1^,5,5a,6,7,8,8a^1^,15,15a-decahydro-2*H*,14*H*-4,6-methanoindolo[3,2,1-*ij*]oxepino[2,3,4-*de*]pyrrolo[2,3-*h*]quinolin-6-ium bromide (3l)**. Light orange powder. Yield 96%, m.p. = 252–254 °C. IR spectrum, ν, cm^−1^: 1626 (C=C), 1681 (C=O), 2956 (CH), 3402 (NH), 3613 (OH). ^1^H NMR (600 MHz, DMSO-*d*_6_) δ 12.74 (s, 1H, NH), 7.68–7.66 (m, 2H, Ar), 7.48 (s, 1H, Ar), 7.30 (d, *J* = 7.7 Hz, 1H, Ar), 7.14–7.13 (m, 3H, Ar), 6.96 (s, 1H, =CH), 6.43 (s, 1H, OH), 5.43 (s, 2H, CH_2_), 4.86 (s, 2H, CH_2_), 4.50–4.49 (m, 1H, CH), 4.41–4.37 (m, 2H, CH_2_), 4.27–4.21 (m, 3H, CH, CH_2_), 4.18–4.12 (m, 3H, CH, CH_2_), 3.81 (s, 3H, CH_3_), 3.75 (s, 3H, CH_3_), 2.97–2.88 (m, 2H, CH_2_), 2.69–2.66 (m, 1H, CH), 2.41–2.46 (m, 1H, CH), 2.33 (s, 3H, CH_3_), 2.20–2.18 (m, 1H, CH), 1.72–1.70 (m, 1H, CH), 1.50–1.48 (m, 1H, CH), 1.33 (s, 18H, CH_3_). ^13^C NMR (150 MHz, DMSO-*d*_6_) δ 168.5, 166.7, 160.5, 153.3, 149.7, 146.0, 141.2, 139.5, 136.2 (CH), 135.6, 134.9, 132.5 (CH), 126.6, 124.1 (CH), 121.7 (CH), 120.1, 118.6, 110.6 (CH), 108.5 (CH), 107.8, 100.3 (CH), 75.7 (CH), 74.7 (CH), 63.2 (CH_2_), 62.6 (CH_2_), 60.6 (CH_2_), 58.8 (CH), 56.8 (CH), 55.7 (CH_3_), 52.1 (CH), 46.2 (CH), 43.0 (CH_2_), 34.4, 30.2 (CH_3_), 30.2 (CH), 24.9 (CH_2_), 20.4 (CH_3_). MS (ESI): *m*/*z* = 828.5 [M-Br]^+^; Found: C, 64.58; H, 6.30; Br, 8.61; N, 7.70. Anal. calcd. (%) for C_49_H_58_BrN_5_O_7_: C, 64.75; H, 6.43; Br, 8.79; N, 7.71.

**6-(2-(2-(1-(3,5-Di-*tert*-butyl-4-hydroxybenzyl)-5-methoxy-2-oxoindolin-3-ylidene)hydrazinyl)-2-oxoethyl)-10,11-dimethoxy-14-oxo-4a,4a^1^,5,5a,6,7,8,8a^1^,15,15a-decahydro-2*H*,14*H*-4,6-methanoindolo[3,2,1-*ij*]oxepino[2,3,4-*de*]pyrrolo[2,3-*h*]quinolin-6-ium bromide (3m)**. Light orange powder. Yield 97%, m.p. = 275–277 °C. IR spectrum, ν, cm^−1^: 1616 (C=C), 1681 (C=O), 2957 (CH), 3403 (NH), 3615 (OH). ^1^H NMR (600 MHz, DMSO-*d*_6_) δ 12.76 (s, 1H, NH), 7.66 (s, 1H, Ar), 7.47–7.45 (m, 1H, Ar), 7.16–7.14 (m, 3H, Ar), 7.07 (dd, *J* = 8.9 Hz, *J* = 2.2 Hz, 1H, Ar), 6.97 (s, 1H, =CH), 6.43 (s, 1H, OH), 5.39 (s, 2H, CH_2_), 4.85 (s, 2H, CH_2_), 4.48–4.45 (m, 1H, CH), 4.40–4.37 (m, 2H, CH_2_), 4.25–4.22 (m, 3H, CH, CH_2_), 4.16–4.13 (m, 3H, CH, CH_2_), 3.80 (s, 6H, CH_3_), 3.75 (s, 3H, CH_3_), 2.95–2.92 (m, 2H, CH_2_), 2.67–2.64 (m, 1H, CH), 2.39–2.34 (m, 1H, CH), 2.20–2.17 (m, 1H, CH), 1.73–1.70 (m, 1H, CH), 1.51–1.49 (m, 1H, CH), 1.33 (s, 18H, CH_3_). ^13^C NMR (150 MHz, DMSO-*d*_6_) δ 168.5, 166.7, 160.4, 155.9, 153.3, 149.7, 146.0, 139.5, 137.0, 135.6 (CH), 132.7, 126.5, 124.0 (CH), 120.1, 119.5, 117.3 (CH), 111.7 (CH), 108.4 (CH), 107.5 (CH), 100.3 (CH), 75.8 (CH), 74.7 (CH), 63.5 (CH_2_), 63.2 (CH_2_), 62.6 (CH_2_), 62.2 (CH), 62.0 (CH), 60.6 (CH_2_), 59.6 (CH_2_), 56.7 (CH_3_), 56.0 (CH_3_), 55.7 (CH_3_), 52.1 (CH), 51.8 (CH), 46.1 (CH), 43.0 (CH_2_), 34.4, 30.3 (CH_3_), 29.0 (CH_2_), 24.8 (CH_2_). MS (ESI): *m*/*z* = 844.5 [M-Br]^+^; Found: C, 63.56; H, 6.27; Br, 8.52; N, 7.50. Anal. calcd. (%) for C_49_H_58_BrN_5_O_8_: C, 63.63; H, 6.32; Br, 8.64; N, 7.57.

**6-(2-(2-(1-(3,5-Di-*tert*-butyl-4-hydroxybenzyl)-6-bromo-2-oxoindolin-3-ylidene)hydrazinyl)-2-oxoethyl)-10,11-dimethoxy-14-oxo-4a,4a^1^,5,5a,6,7,8,8a^1^,15,15a-decahydro-2*H*,14*H*-4,6-methanoindolo[3,2,1-*ij*]oxepino[2,3,4-*de*]pyrrolo[2,3-*h*]quinolin-6-ium bromide (3n)**. Yellow powder. Yield 95%, m.p. = 283–285 °C. IR spectrum, ν, cm^−1^: 1653 (C=C), 1698 (C=O), 2925 (CH), 3426 (NH). Due to the impossibility of obtaining a solution of high concentration, ^1^H and ^13^C NMR spectra were not recorded. MS (MALDI): *m*/*z* = 894.5 [M-Br]^+^; Found: C, 59.08; H, 5.47; Br, 16.30; N, 7.04. Anal. calcd. (%) for C_48_H_55_Br_2_N_5_O_7_: C, 59.20; H, 5.69; Br, 16.41; N, 7.19.

### 3.2. Biological Studies

**Antimicrobial activity**

Gram-positive bacteria (*Staphylococcus aureus* ATCC 6538P FDA 209P, *Bacillus cereus* ATCC 10702 NCTC 8035, *Enterococcus faecalis* ATCC 29212, and methicillin-resistant *Staphylococcus aureus* MRSA-1 and MRSA-2), were used as test objects. As a reference drug for studying antibacterial activity, norfloxacin was used. Bacteriostatic properties were studied by the method of serial dilutions in liquid nutrient media by procedures described in [53], determining MIC, which inhibits the growth and production of the test microorganism. The MBC, causing complete death of the pathogen was determined according to the previously described procedure [54].

Bacterial strains were purchased from the State Collection of Pathogenic Microorganisms and Cell Cultures “GKPM-Obolensk” and methicillin-resistant strains MRSA-1 and MRSA-2 were obtained from hospital patients in the Republican Clinical Hospital (Kazan, Russia). To perform the tests, 96-well plates were prepared with Mueller–Hinton broth. The plates were then inoculated with a standardized suspension of the test microorganism (*S. aureus*, *B. cereus,* and *E. faecalis*). The concentration of bacteria was 3.0 × 10^5^ CFU/mL (colony-forming units per milliliter). Bacterial cultures were incubated at 37 °C. Data were collected every 24 h for 5–7 days, and the experiment was replicated three times. Compounds were diluted directly in nutrient media. The stock solution was supplemented with compounds at a concentration of 500 μM, as well as 5% DMSO for better solubility. The range of tested concentrations was 0.5–250 μM. Control wells contained 2.5% DMSO. It was shown that DMSO at this concentration does not inhibit bacterial growth.

**Hemolytic activity**

Hemolytic activity of **3a–3n** was estimated by comparing the optical density of a solution containing the test compound with that of blood at 100% hemolysis. Substances of gramicidin S (Sigma) were used as a reference drug. The experiments were carried out as described earlier [55].

**Cytotoxicity assay**

Substances of doxorubicin (Merck Life Science LLC, Moscow, Russia) were used as a reference drug. Hepatocyte-like cells (Chang liver) from the collection at the Research Institute of Virology of the Russian Academy of Medical Sciences (Moscow, Russia) were used in experiments. The cells were cultured on the standard nutrient medium Eagle from the Chumakov Research Institute of Poliomyelitis and Viral Encephalitis (PanEco, Moscow, Russia), supplemented with 10% fetal calf serum and 1% non-essential amino acids. The cytotoxic effect on cells was determined by the colorimetric method of cell proliferation, that is, the MTT test. Cells were seeded on a 96-well Nunc plate at a concentration of 5 × 10^3^ cells per well in a volume of 100 μL of medium and cultured in a CO_2_ incubator at 37 °C until a monolayer was formed. The nutrient medium was then removed, and 100 μL of the test drug solutions at the specified dilutions, prepared directly in the nutrient medium with the addition of 1% DMSO to improve solubility, were added to the wells. Cytotoxicity analysis was performed in the concentration range (1–100 μM). After 24 h of cell incubation with the test compounds, the nutrient medium was removed from the plates, and 100 μL of serum-free nutrient medium containing MTT at a concentration of 0.5 mg mL^−1^ was added and incubated for 4 h at 37 °C. Then, 100 μL of DMSO was added to the formazan crystals in each well. Optical density was recorded at 540 nm on an Invitrologic plate reader (Novosibirsk, Russia). IC_50_ (half maximum inhibitory concentration) was calculated using an online tool: MLA–“Quest Graph™ IC_50_ Calculator” (AAT Bioquest, Inc., Pleasanton, CA, USA, 6 March 2024, https://www.aatbio.com/tools/ic50-calculator). The selectivity index (SI) was calculated as the ratio between the IC_50_ value for normal cells and the IC_50_ value for cancer cells. Experiments were repeated three times. Intact cells cultured together with experimental cells were used as a control [56].

**Antioxidant Potential Study**

The phenolic isatin-3-hydrazones containing a quaternary ammonium center in the concentration range from 0.01 to 100 μM and rat brain homogenate (2 mg/mL) were introduced into the wells of the deep-hole plate. Each concentration of the test substance was measured in triplicate.

FeSO_4_ decahydrate was added as an initiator, participating in the cyclic Fenton reaction and, as a result, leading to the formation of reactive hydroxyl radicals. After a 30-min incubation at 37 °C, a reagent for TBARs-reactive products was added to each sample, incubated for 90 min at 90 °C.

After 90 min, the samples were centrifuged at 6000 rpm for 20 min and the optical density of the selected supernatant was measured on an Invitrologic plate reader (Novosibirsk, Russia) at λ = 540 nm.

Additionally, the values of semi-maximal inhibition (IC_50_) of lipid peroxidation were calculated, which represent concentrations at which the level of malondialdehyde was reduced by 50%.

In this work, the standard antioxidant Trolox was used as a positive control.

**Anticoagulant and Antiaggregation Activities Study**

The in vitro experiments were performed using the blood of healthy male donors aged 18–24 years (total 48 donors). The study was approved by the Ethics Committee of the Federal State Budgetary Educational Institution of Higher Education at the Bashkir State Medical University of the Ministry of Health of the Russian Federation (No. 1 dated 30 January 2024). Informed consent was obtained from all participants before blood sampling. The blood was collected from the cubital vein using a system of vacuum blood collection, the BD Vacutainer^®^ (Becton, Dickinson and Company, Franklin Lakes, NJ, USA). A 3.8% sodium citrate solution in a 9:1 ratio was used as a venous blood stabilizer. The study of the effect on platelet aggregation was performed using the Born method [57] using the aggregometer «AT-02» (SPC Medtech, Moscow, Russia). The assessment of antiplatelet activity of the studied compounds and reference preparations was started with the final concentration of 2 × 10^−3^ mol/L. Adenosine diphosphate (ADP; 20 μg/mL) and collagen (5 mg/mL) manufactured by Tehnologia-Standart Company, Russia, were used as inducers of aggregation. The study on the anticoagulant activity was performed by standard recognized clotting tests using an optical two-channel automatic analyzer of blood coagulation, the Solar CGL 2110 (CJSC SOLAR, Minsk, Belarus). The following parameters were studied: activated partial thromboplastin time (APTT), prothrombin time (PT), and fibrinogen concentrations according to the Clauss method. The determination of anticoagulant activity of the studied compounds and reference preparation was performed in a concentration of 5 × 10^−4^ g/mL using the reagents manufactured by Tehnologia-Standart Company (Barnaul, Russia).

### 3.3. Statistical Analysis

The data were expressed as mean ± SEM. Statistical comparisons were made using a one-way analysis of variance (ANOVA) followed by Dunnett’s Multiple Comparison tests. The two-way repeated measures (mixed model) ANOVA followed by Bonferroni posttests were also used to compare the recognition of two objects. A difference with a *p*-value ≤ 0.05 was considered statistically significant. The statistical analysis was performed using GraphPad Prism 5 (GraphPad Software, San Diego, CA, USA). The IC_50_ values were calculated using the online calculator MLA-Quest Graph™ IC_50_ Calculator (AAT Bioquest, Inc., 14 February 2021). Statistical analysis was performed using the Mann–Whitney test (*p* < 0.05). Tabular and graphical data contain mean values and standard deviation. The results of the study of the anticoagulant and antiaggregation activities were processed using the statistical package Statistica 10.0 (StatSoft Inc., Tulsa, OK, USA). The Shapiro–Wilk test was used to check the normality of actual data distribution. The form of distribution of the data obtained differed from the normal one; therefore, non-parametric methods were used for further analysis. The data were presented as medians and 25 and 75 percentiles. Analysis of variance was conducted using the Kruskal–Wallis test. A *p*-value of 0.05 was considered statistically significant.

## 4. Conclusions

In order to obtain potential drug candidates, a series of novel hybrid phenolic isatin-3-hydrazones containing a quaternary ammonium center of various lipophilicity and rigidity was synthesized with high yields by the simple and easy work-up reaction of Girard’s reagents analogs with 1-(3,5-di-*tert*-butyl-4-hydroxybenzyl)isatin. The purpose of introducing a fragment of sterically hindered phenol into the structure of the hybrid molecule was to add antioxidant action to the main properties of isatins, mediating an additional spectrum of positive biological effects. All the studied compounds highly suppressed lipid peroxidation of rat brain homogenates, thereby demonstrating antioxidant activity. Moreover, the synthesized hybrids exhibited anticoagulant and antiaggregation properties, which may, in the future, reduce the overall systemic toxicity to the human body and correct blood toxic effects. In turn, due to the use of isatin-3-hydrazones as a basis, the tested compounds exhibited a pronounced antimicrobial effect.

## Figures and Tables

**Scheme 2:**
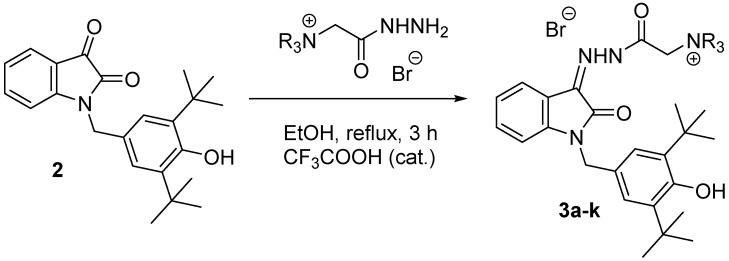
Synthesis of isatin hydrazones containing an ammonium center of various structures.

**Table 1:** Antimicrobial activity against some gram-positive bacterial strains of compounds under study *.

Cmpd.	MIC/MBC, μM				
	*Sa*	*Bc*	*Ef*	*MRSA-1*	*MRSA-2*
**3a**	3.0 ± 0.2/11.9 ± 0.9	11.9 ± 1.1/n.a.	47.5 ± 4.2/190 ± 18	3.0 ± 0.2/23.8 ± 1.9	5.9 ± 0.6/95 ± 7.8
**3b**	5.7 ± 0.5/91.1 ± 8.7	11.4 ± 0.8/n.a.	45.6 ± 4.3/91.1 ± 8.2	5.7 ± 0.4/11.4 ± 0.9	2.8 ± 0.2/91.1 ± 7.5
**3c**	41.0 ± 3.8/325 ± 28	325 ± 26/n.a.	20.3 ± 1.7/325 ± 31	n.d.	n.d.
**3d**	78.3 ± 6.7/n.a.	n.a.	39.2 ± 2.8/157 ± 14	n.d.	n.d.
**3e**	5.9 ± 0.5/5.9 ± 0.5	5.9 ± 0.6/n.a.	11.8 ± 0.7/47.3 ± 3.8	5.9 ± 0.5/11.8 ± 1.0	3.0 ± 0.2/23.7 ± 1.9
**3f**	4.5 ± 0.4/4.5 ± 0.4	36.3 ± 2.8/n.a.	9.0 ± 0.8/18.1 ± 1.6	4.5 ± 0.3/36.3 ± 2.9	36.3 ± 2.7/36.3 ± 2.7
**3g**	n.a.	n.a.	n.a.	n.d.	n.d.
**3h**	51.2 ± 4.4/51.2 ± 4.4	n.a.	51.2 ± 4.8/51.2 ± 4.8	n.d.	n.d.
**3i**	n.a.	n.a.	n.a.	n.d.	n.d.
**3j**	n.a.	n.a.	n.a.	n.d.	n.d.
**3k**	35.0 ± 2.9/35.0 ± 2.9	17.5 ± 1.5/n.a.	n.a.	n.d.	n.d.
**3l**	8.6 ± 0.7/138 ± 12	8.6 ± 0.8/68.8 ± 6.2	n.a.	34.4 ± 2.8/68.8 ± 5.7	34.4 ± 2.8/68.8 ± 6.3
**3m**	4.2 ± 0.3/8.4 ± 0.7	16.9 ± 1.4/67.6 ± 5.5	n.a.	16.9 ± 1.5/270 ± 22	16.9 ± 1.3/67.6 ± 6.4
**3n**	128 ± 11/n.a.	n.a.	n.a.	n.d.	n.d.
Norfloxacin	12.2 ± 0.9/12.2 ± 0.9	24.5 ± 1.7/24.5 ± 1.7	24.5 ± 1.7/48.9 ± 3.2	>500/>500	12.2 ± 0.8/48.9 ± 3.3

* *Staphylococcus aureus* (*Sa*), *Bacillus cereus* (*Bc*), *Enterococcus faecalis* (*Ef*), Methicillin-resistant *Staphylococcus aureus* (*MRSA-1* and *MRSA-2*); MIC-minimum inhibitory concentration; MBC-minimum bactericidal concentration; n.d.-not determined; n.a.-no activity: MIC, MBC > 500 μM; experiments were carried out in triplicate.

**Table 2:** IC_50_ values of the lipid peroxidation inhibiting effects of synthesized phenolic isatin-3-hydrazones.

Cmpd.	IC_50_, μM	Cmpd.	IC_50_, μM
**3a**	4.53 ± 0.22	**3h**	4.53 ± 0.22
**3b**	8.98 ± 0.12	**3i**	5.79 ± 0.00
**3c**	27.77 ± 0.25	**3j**	6.85 ± 0.28
**3d**	15.24 ± 0.11	**3k**	5.47 ± 0.13
**3e**	3.41 ± 0.16	**3l**	8.18 ± 0.31
**3f**	6.38 ± 0.01	**3m**	6.79 ± 0.18
**3g**	6.68 ± 0.02	**3n**	7.32 ± 0.11
**Trolox**		30.90 ± 1.54	

**Table 3:** Anticoagulant and antiaggregating activity of compounds.

Cmpd.	Latent Period, % of Control	Maximum Amplitude (MA), % of Control	Aggregation Rate, % of Control	Time to MA, % of Control	APTT ^$^, % of Control
**3a**	+3.7 (3.1–4.5) ^#^	−4.3 (3.2–5.7) *^,#^	+4.2 (3.1–5.8) ^#^	+14.6 (13.2–17.5) *^,#^	+1.2 (0.7–2.4)
**3b**	+4.6 (3.1–6.2) ^#^	−14.4 (11.3–16.7) *	−10.4 (8.3–12.1) *	+18.6 (14.9–21.3) *^,#^	+3.7 (3.2–5.6)
**3c**	+6.1 (4.7–7.2) *^,#^	−13.1 (10.7–14.5) *	−20.7 (18.3–24.1) *^,#^	−14.1 (11.2–15.7) *^,#^	+6.2 (5.7–9.4) *^,†^
**3d**	+2.3 (2.1–3.7) ^#^	−1.6 (1.2–3.5) ^#^	−4.1 (3.7–5.2) ^#^	−10.5 (9.3–13.6) *^,#^	+1.9 (1.4–3.3)
**3e**	+7.4 (5.3–8.2) *^,#^	−18.1 (15.3–19.7) *	−8.9 (6.1–11.7) *	+15.9 (12.4–17.5) *	+6.5 (4.8–7.3) *^,†^
**3f**	+10.2 (8.9–13.5) *	−11.6 (9.4–12.3) *	−11.5 (8.5–13.4) *	−17.5 (16.4–20.3) *^,#^	+3.4 (2.7–5.9)
**3g**	−3.0 (1.5–4.3)	−14.9 (13.3–15.9) *	−12.1 (10.9–14.3) *	−26.7 (24.4–28.7) *^,#^	+6.3 (5.6–7.4) *^,†^
**3h**	+4.1 (3.8–5.3) *^,#^	−1.2 (1.0–2.8) ^#^	−2.3 (1.8–3.5) ^#^	−15.6 (14.8–17.2) *^,#^	+2.4 (1.7–3.6)
**3i**	+7.1 (6.4–7.9) *^,#^	−2.6 (1.6–3.7) ^#^	+24.9 (21.8–27.4) *^,#^	−15.6 (14.5–16.7) *^,#^	+7.3 (6.2–10.1) *^,†^
**3j**	−12.1 (9.4–13.9) *^,#^	−20.7 (18.6–23.8) *^,#^	−31.2 (30.4–33.5) *^,#^	+12.7 (10.4–14.5) *	+3.2 (2.5–4.7)
**3k**	−3.1 (2.5–4.6) ^#^	+5.2 (3.4–8.2) ^#^	+2.5 (1.5–4.3) ^#^	−12.6 (10.2–14.7) *^,#^	+7.1 (6.3–8.2) *^,†^
**3l**	−25.0 (21.5–28.5) *^,#^	−20.5 (17.8–22.4) *^,#^	−16.9 (14.8–18.3) *^,#^	+19.3 (17.6–20.5) *^,#^	+19.2 (16.3–20.7) *
**3m**	−3.1 (2.7–5.1)	−17.8 (15.9–16.6) *	−18.7 (16.5–19.2) *^,#^	+17.2 (16.4–19.3) *^,#^	+15.4 (13.2–17.1) *^,†^
**3n**	+2.8 (2.1–3.6) ^#^	+1.1 (0.9–2.4) ^#^	−1.1 (0.5–1.6) ^#^	−11.7 (10.5–14.5) *^,#^	+2.1 (1.3–3.7)
**Acetylsalicylic acid**	−2.1 (1.1–2.6)	−13.7 (10.8–16.4) *	−10.5 (7.6–12.3) *	+10.5 (8.7–13.4) *	-
**Heparin sodium**	-	-	-	-	+20.3 (19.7–21.4) *

* *p* < 0.05-compared to control; ^#^ *p* < 0.05-compared to acetylsalicylic acid; ^†^ *p* < 0.05-compared to Heparin sodium; ^$^ APTT-activated partial thromboplastin time.

## Data Availability

Samples of all described compounds are available from the author A. Bogdanov.

## Supplementary Materials

The supporting information can be downloaded at: https://www.mdpi.com/article/10.3390/ijms252011130/s1, Figures S1–S111—Copies of NMR, IR, and mass-spectra of all synthesized compounds.
