# Supplementary material for: Biological Activity Evaluation of Phenolic Isatin-3-Hydrazones Containing a Quaternary Ammonium Center of Various Structures
Source: Int J Mol Sci. 2024 Oct 17;25(20):11130. doi: 10.3390/ijms252011130 (PMC11507835; doi:10.3390/ijms252011130)
Supplement: Supplementary file 1 [file ijms-25-11130-s001.zip › ijms-3215704-supplementary.pdf]

SUPPLEMENTARY MATERIALS

Biological activity evaluation of phenolic isatin-3-hydrazones containing a quaternary ammonium center of various structures

Margarita Neganova[1,2,3], Yulia Aleksandrova[1,2,3], Alexandra Voloshina[1], Anna Lyubina[1], Nurbol Appazov[3,4], Sholpan Yespenbetova[3], Zulfiia Valiullina[5], Aleksandr Samorodov[5], Sergey Bukharov[6], Elmira Gibadullina[1,3], Anipa Tapalova[3,]* and Andrei Bogdanov[1,]*

[1] Arbuzov Institute of Organic and Physical Chemistry, FRC Kazan Scientific Center, Russian Academy of Sciences, Akad. Arbuzov st. 8, Kazan, 420088, Russia; abogdanov@inbox.ru (AB); microbi@iopc.ru (AV); aplyubina@gmail.com (AL); elmirak_1978@mail.ru (EG)
[2] Nesmeyanov Institute of Organoelement Compounds, Russian Academy of Sciences, 119991 Moscow, Russia; neganova83@mail.ru (MN); yulia.aleks.97@mail.ru (YuA)
[3] Korkyt Ata Kyzylorda University, Ayteke bi, Str., 29A, Kyzylorda, 120014, Kazakhstan; nurasar.82@korkyt.kz (NA); sholpan-sultan@mail.ru (SYe); anipa52@mail.ru (AT)
[4] ''CNEC'' LLP, Dariger Ali Lane, 2, Kyzylorda 120001, Kazakhstan
[5] Bashkir State Medical University, Lenin st. 8, Ufa, 450008, Russia; z_suleimanova@mail.ru (ZV); avsamorodov@gmail.com (AS)
[6] Kazan National Research Technological University, K. Marx str. 68, Kazan 420015 Russia; svbukharov@mail.ru (SB)

*Correspondence: abogdanov@inbox.ru (AB), anipa52@mail.ru (AT)

Contents

Copies of NMR spectra 2-73

Copies of IR spectra 74-86

Copies of mass-spectra 87-112

Copies of NMR spectra

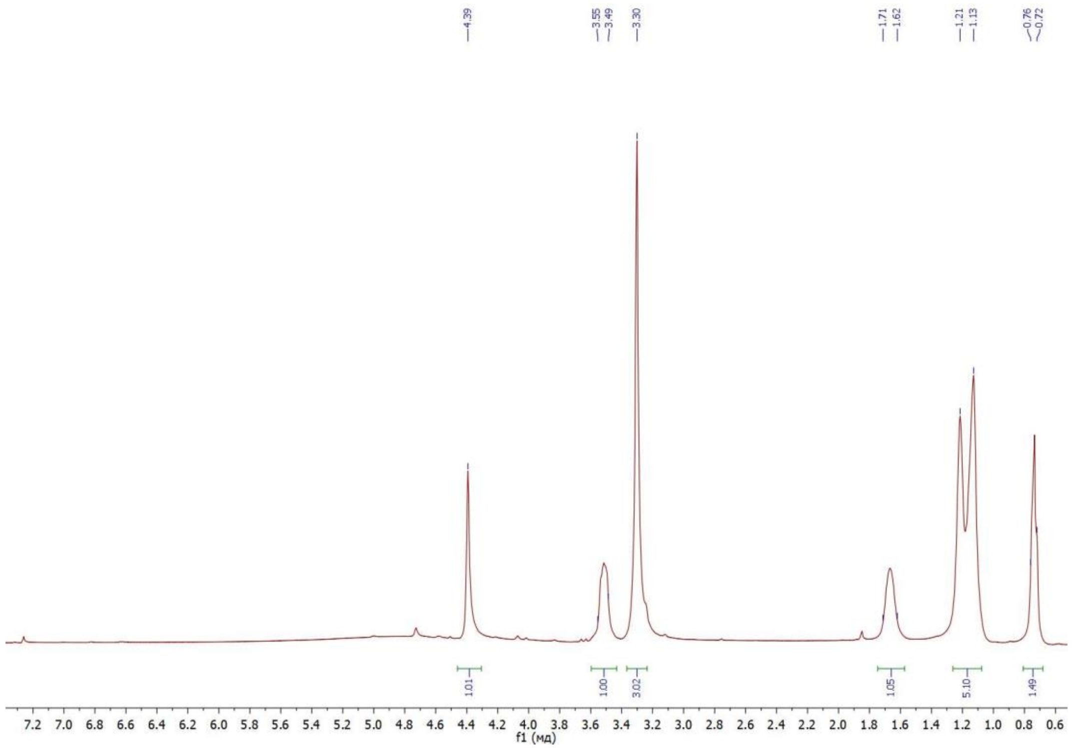

Figure S1. ^{1}H NMR spectrum of compound **1a** (400 MHz, $CDCl_3$)

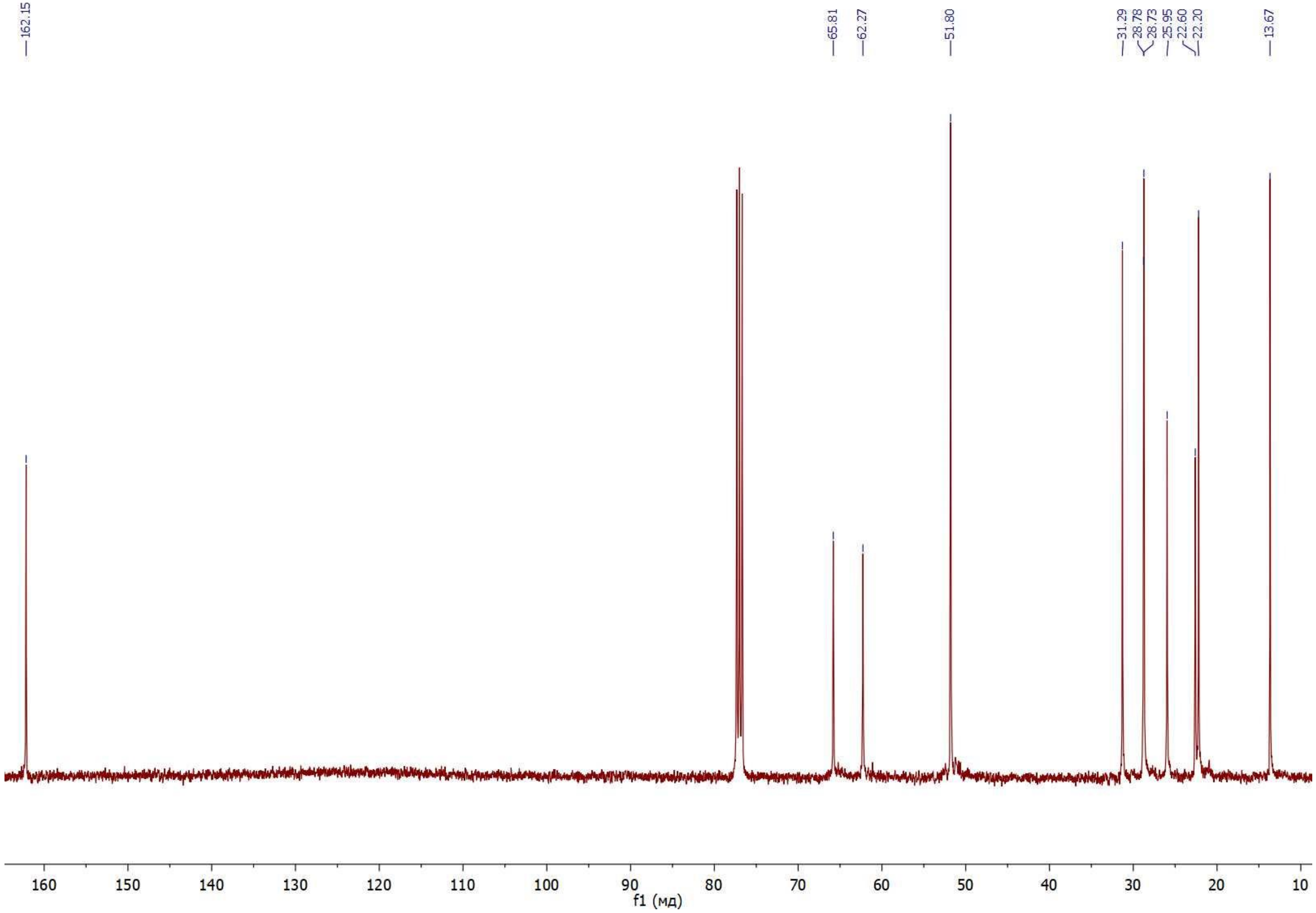

Figure S2. ^{13}C-{^{1}H} NMR spectrum of compound **1a** (101 MHz, $CDCl_3$)

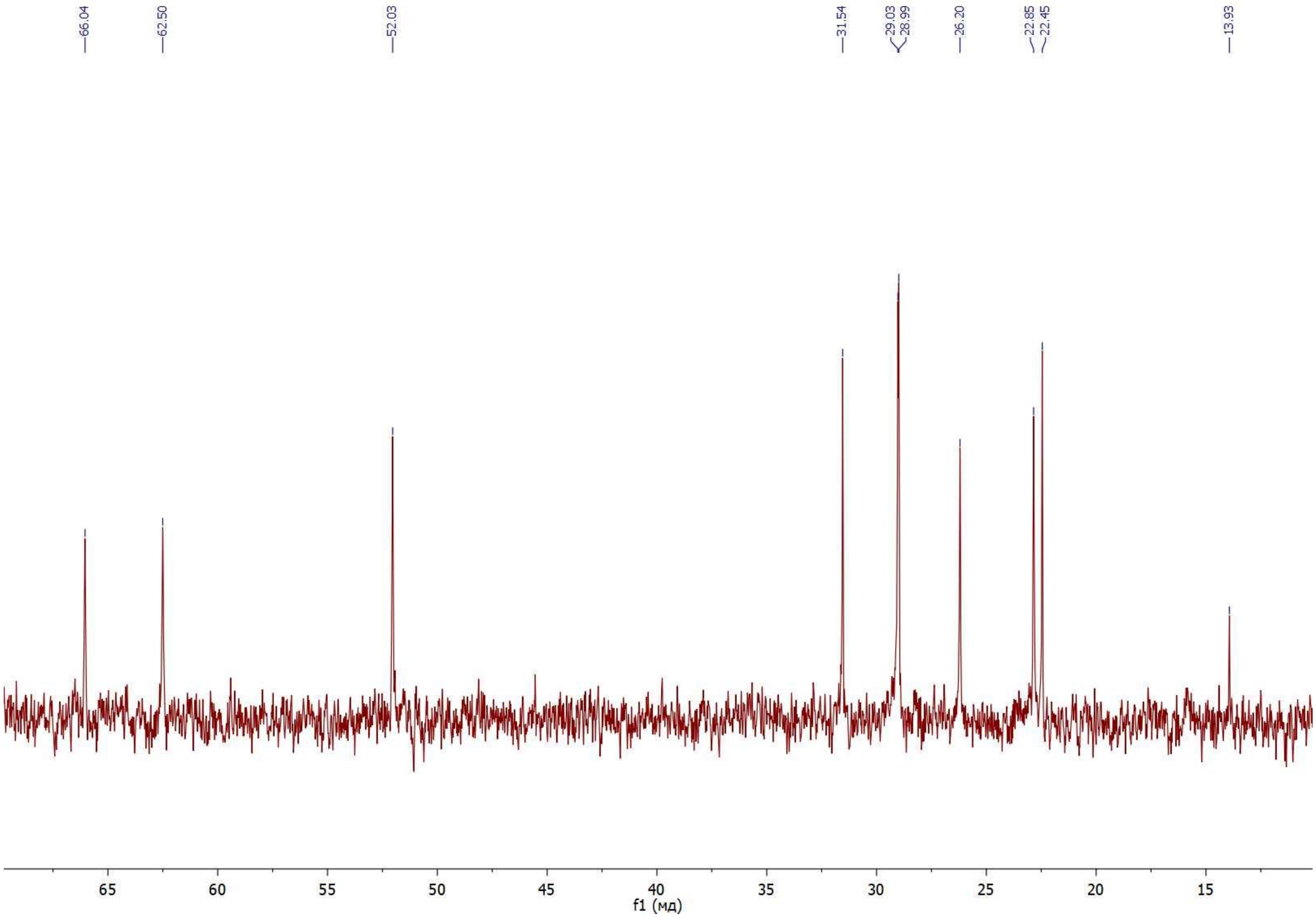

Figure S3. ^{13}C (dept) NMR spectrum of compound **1a** (101 MHz, $CDCl_3$)

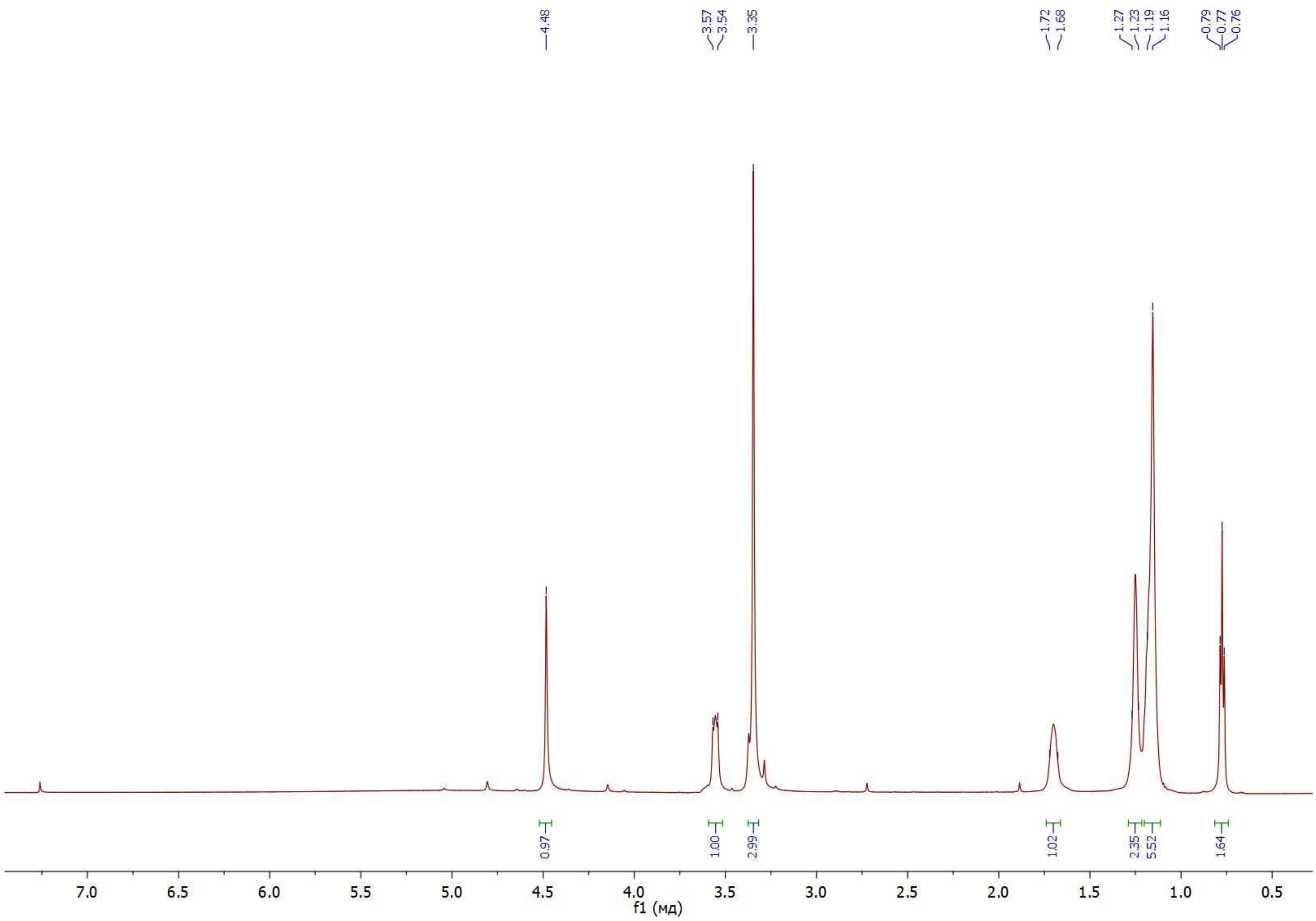

Figure S4. ^{1}H NMR spectrum of compound **1b** (600 MHz, $CDCl_3$)

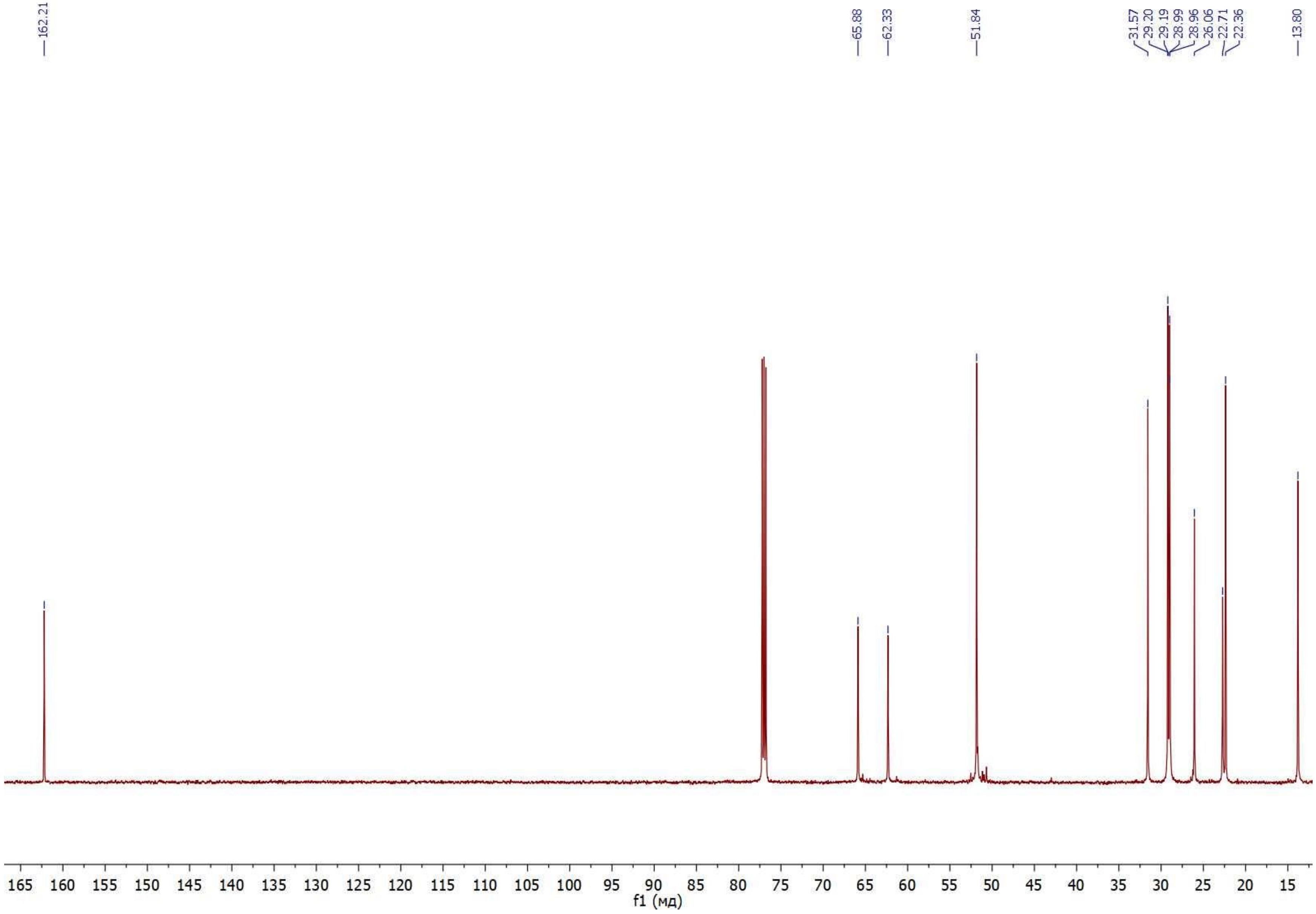

Figure S5. ^{13}C-{^{1}H} NMR spectrum of compound **1b** (151 MHz, $CDCl_3$)

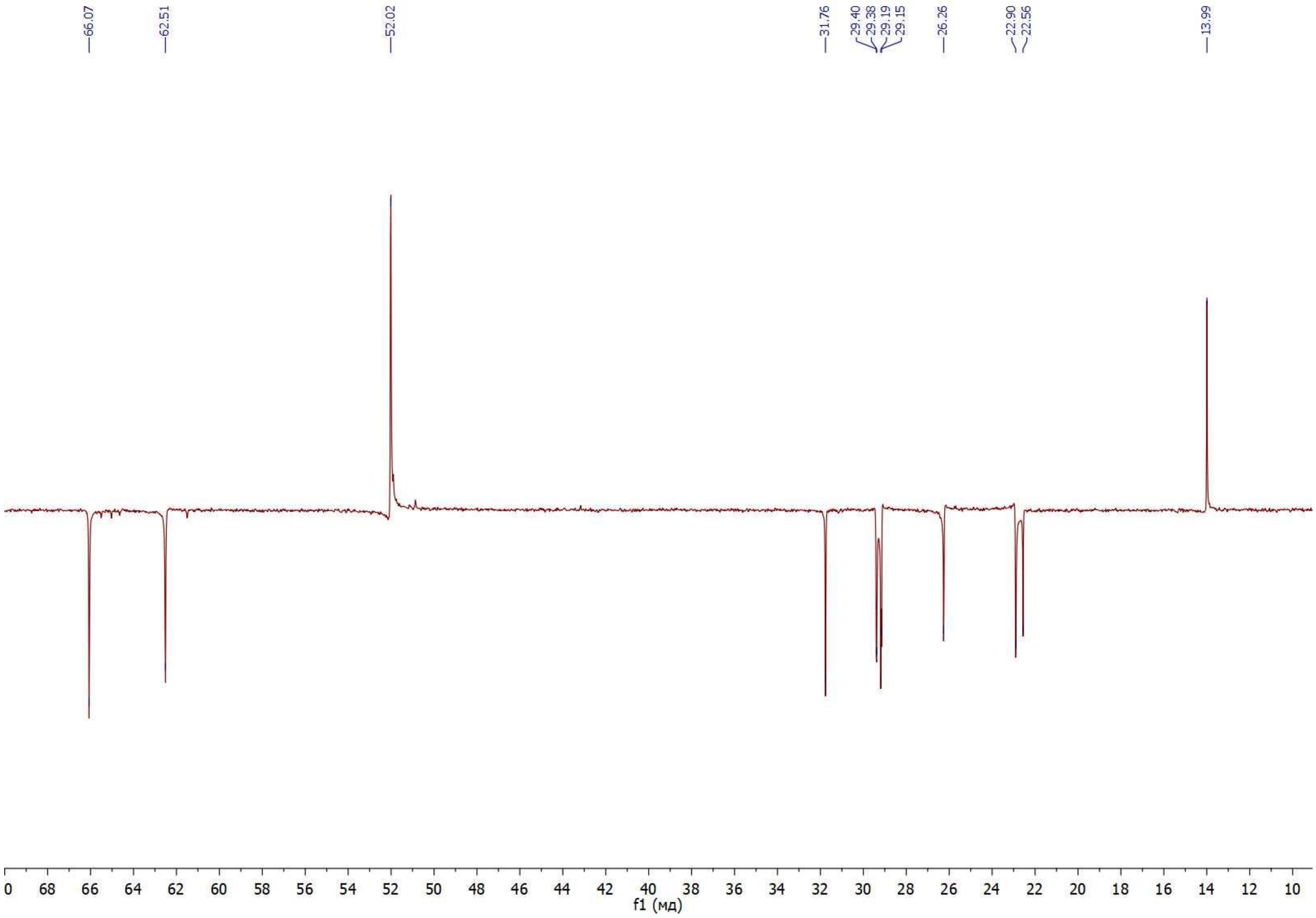

Figure S6. ^{13}C (dept) NMR spectrum of compound **1b** (151 MHz, $CDCl_3$)

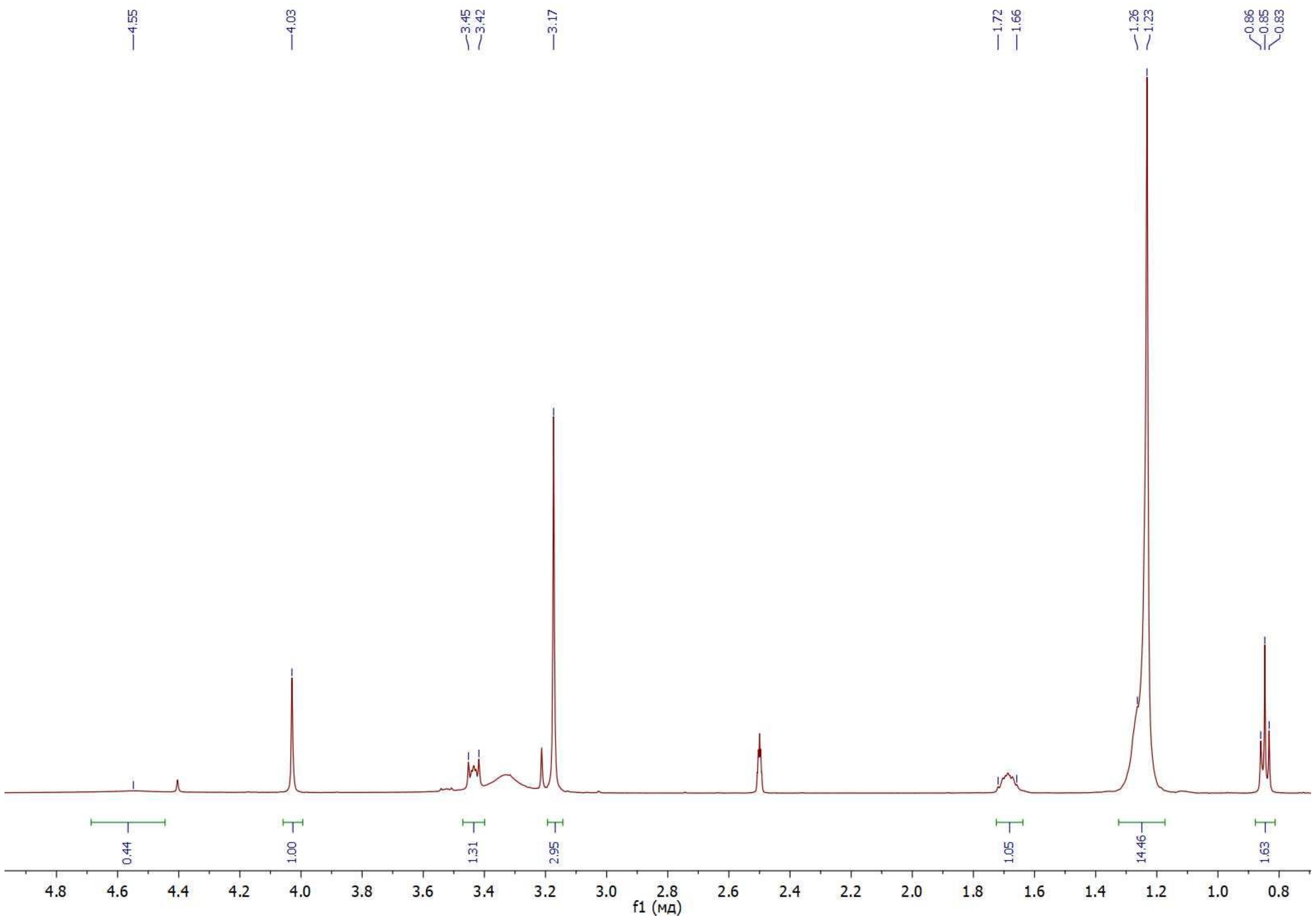

Figure S7. ^{1}H NMR spectrum of compound **1c** (400 MHz, DMSO-d_6)

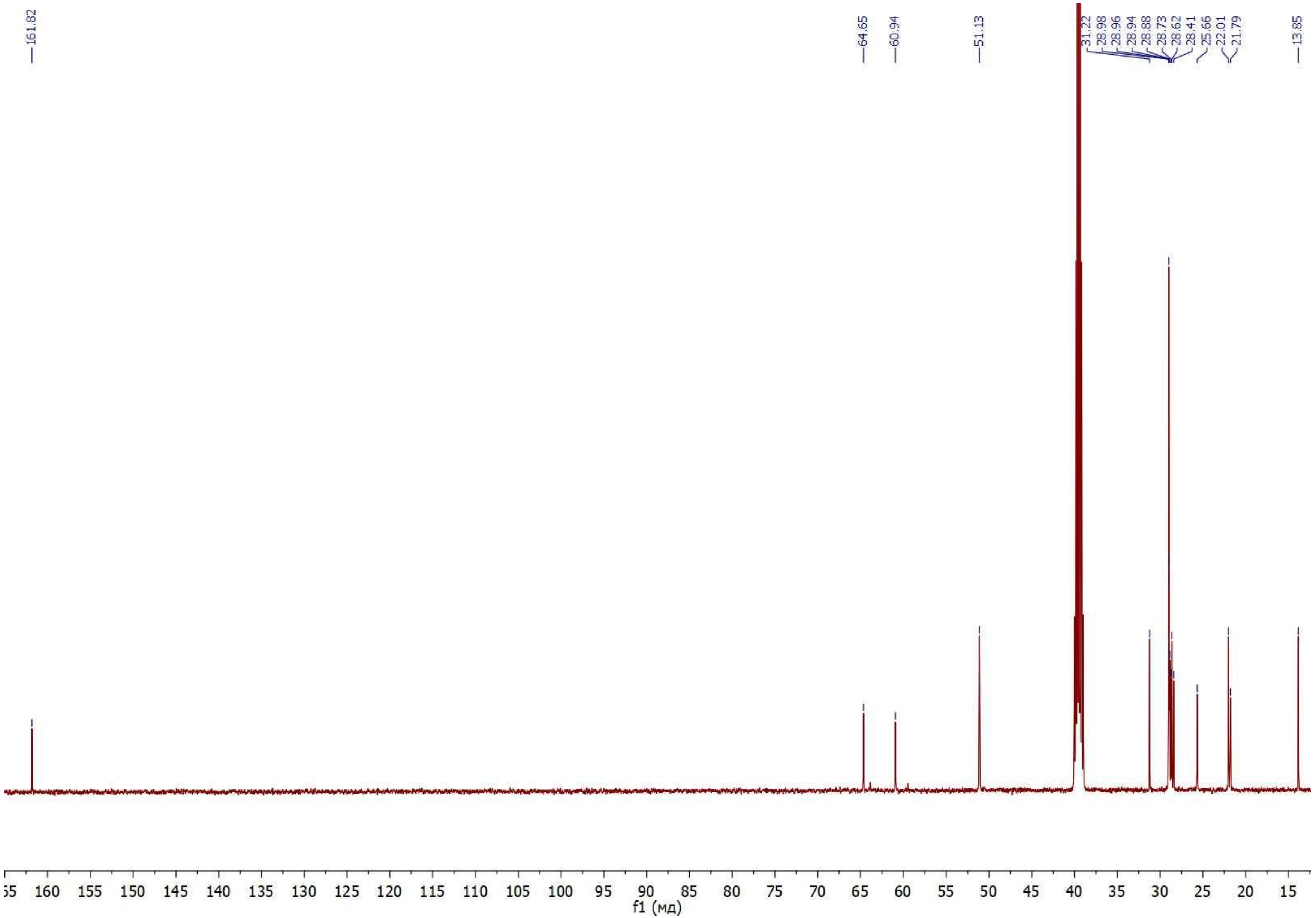

Figure S8. $^{13}C-\{^{1}H\}$ NMR spectrum of compound **1c** (101 MHz, DMSO-d_6)

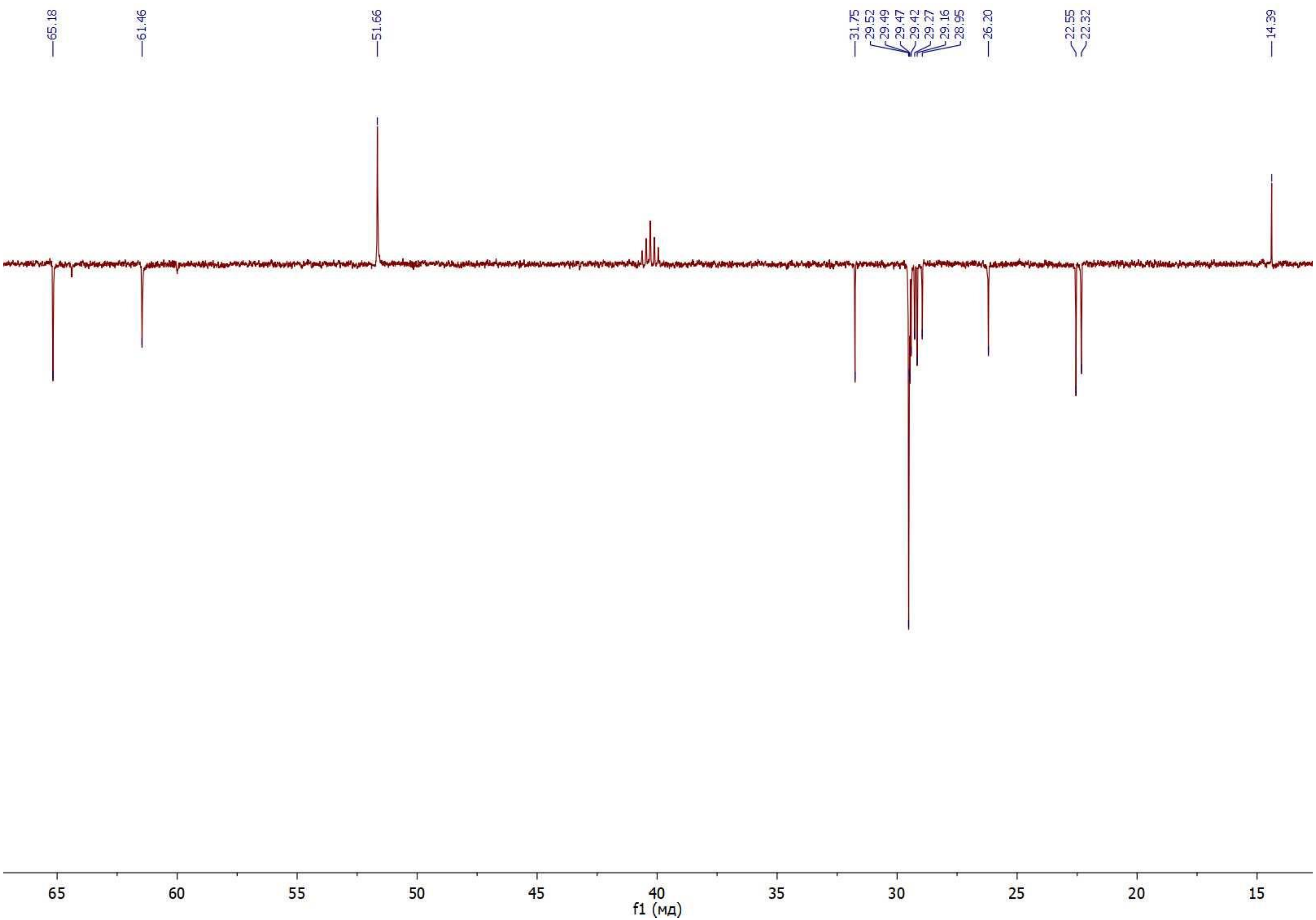

Figure S9. ^{13}C (dept) NMR spectrum of compound **1c** (101 MHz, DMSO-d_6)

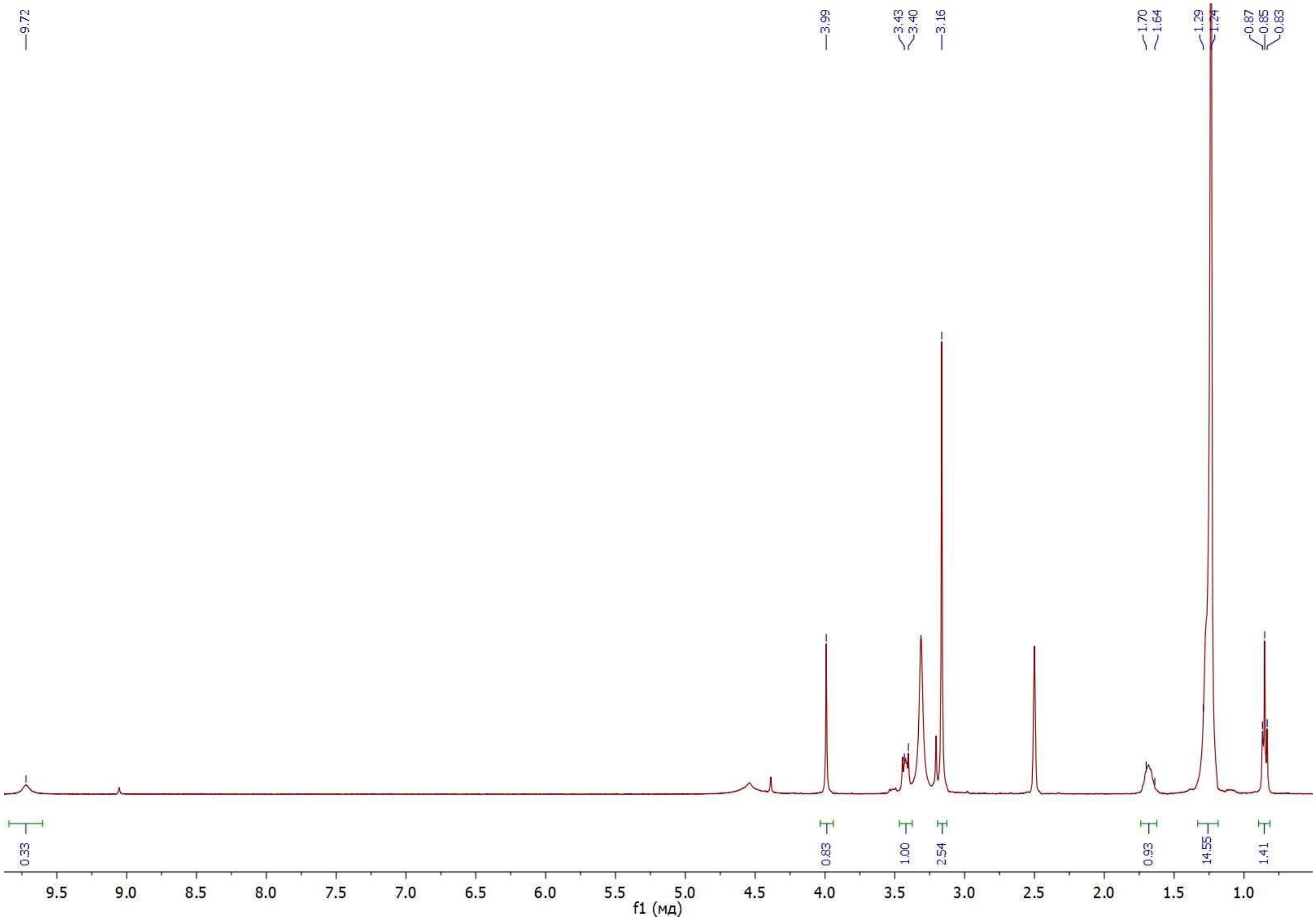

Figure S10. ¹H NMR spectrum of compound **1d** (400 MHz, DMSO-d_6)

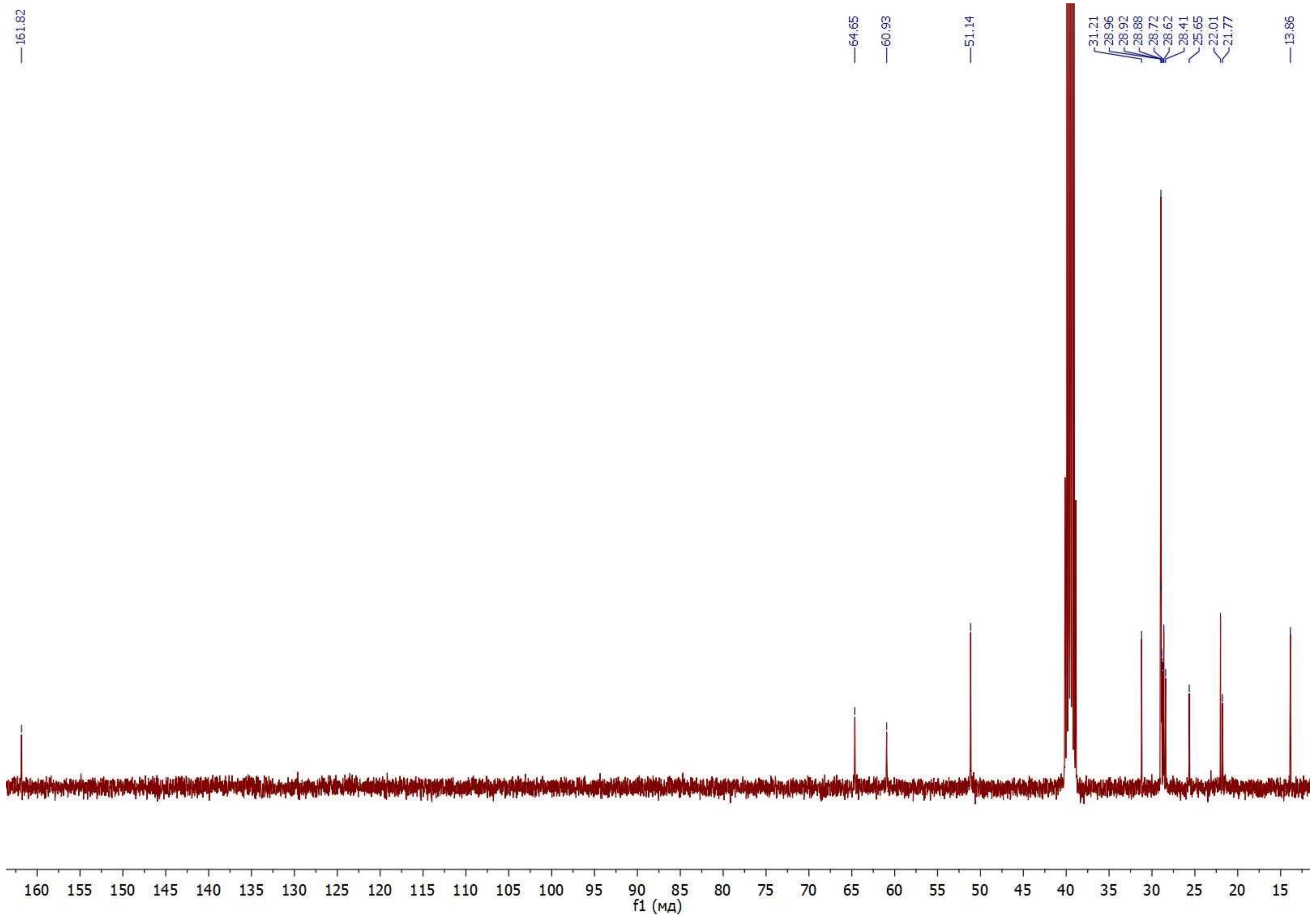

Figure S11. ^{13}C-{^{1}H} NMR spectrum of compound **1d** (101 MHz, DMSO-d_6)

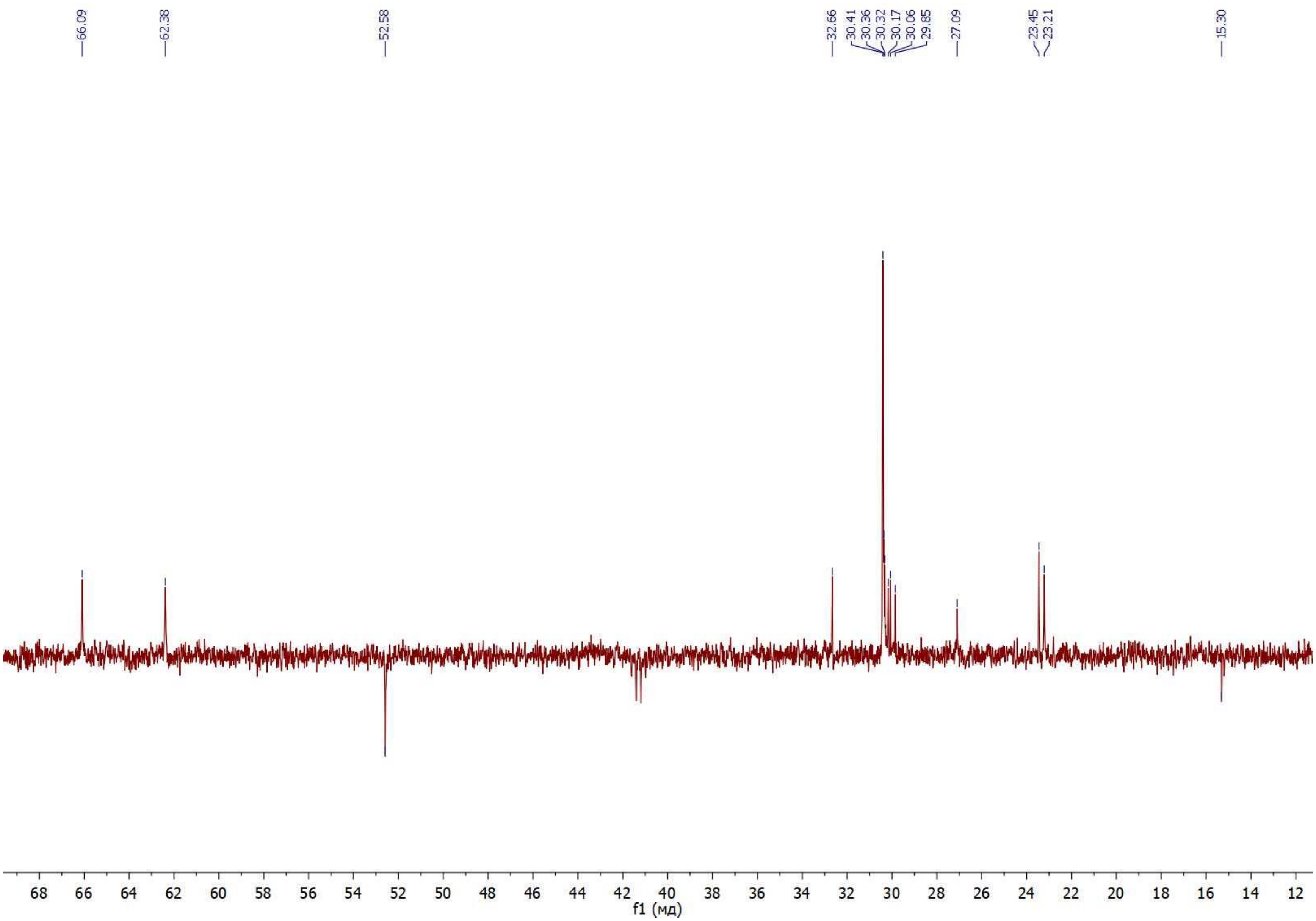

Figure S12. ^{13}C (dept) NMR spectrum of compound **1d** (101 MHz, DMSO-d_6)

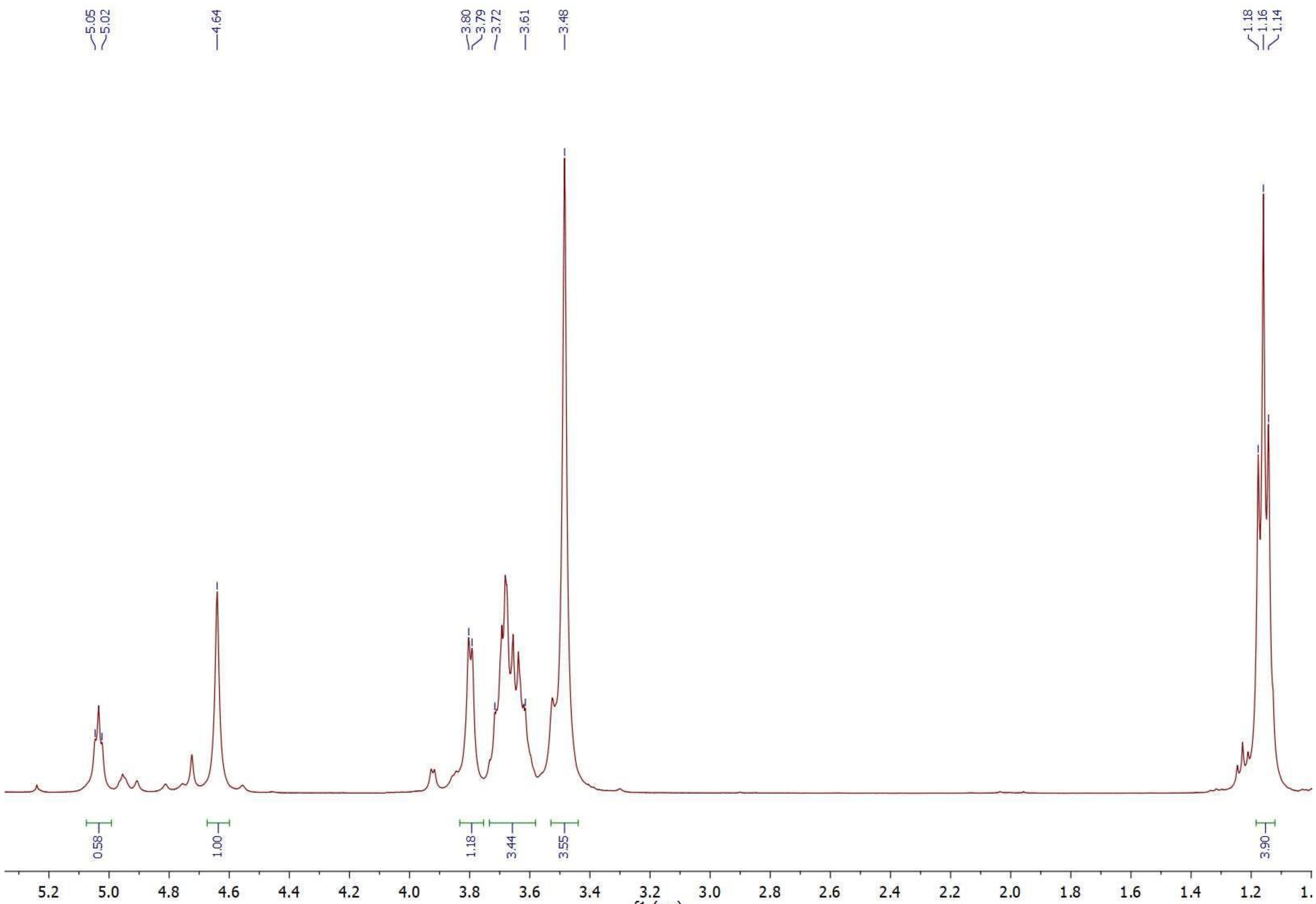

Figure S13. ^{1}H NMR spectrum of compound **1e** (400 MHz, $CDCl_3$)

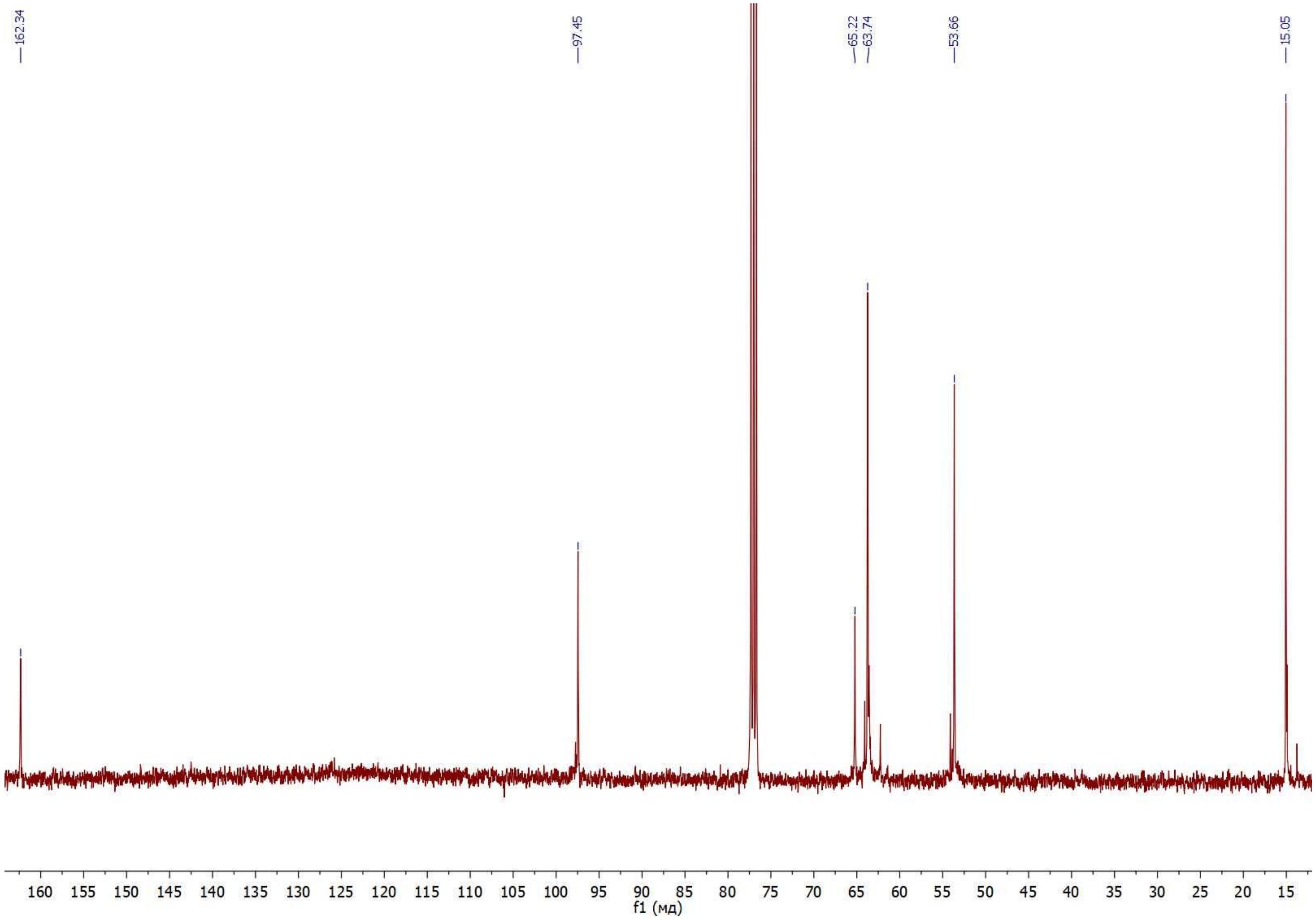

Figure S14. ^{13}C-{^{1}H} NMR spectrum of compound **1e** (101 MHz, $CDCl_3$)

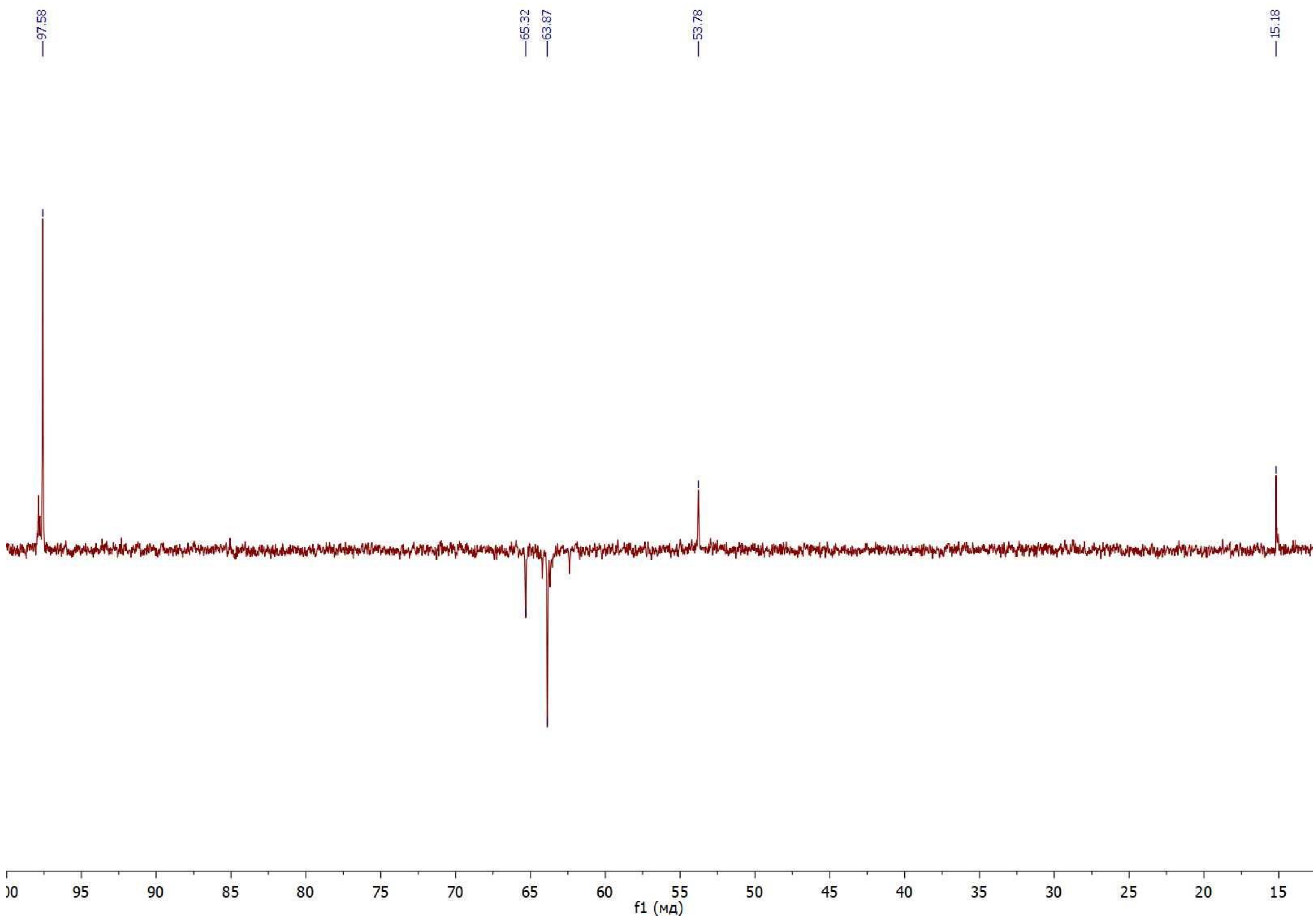

Figure S15. ^{13}C (dept) NMR spectrum of compound **1e** (101 MHz, $CDCl_3$)

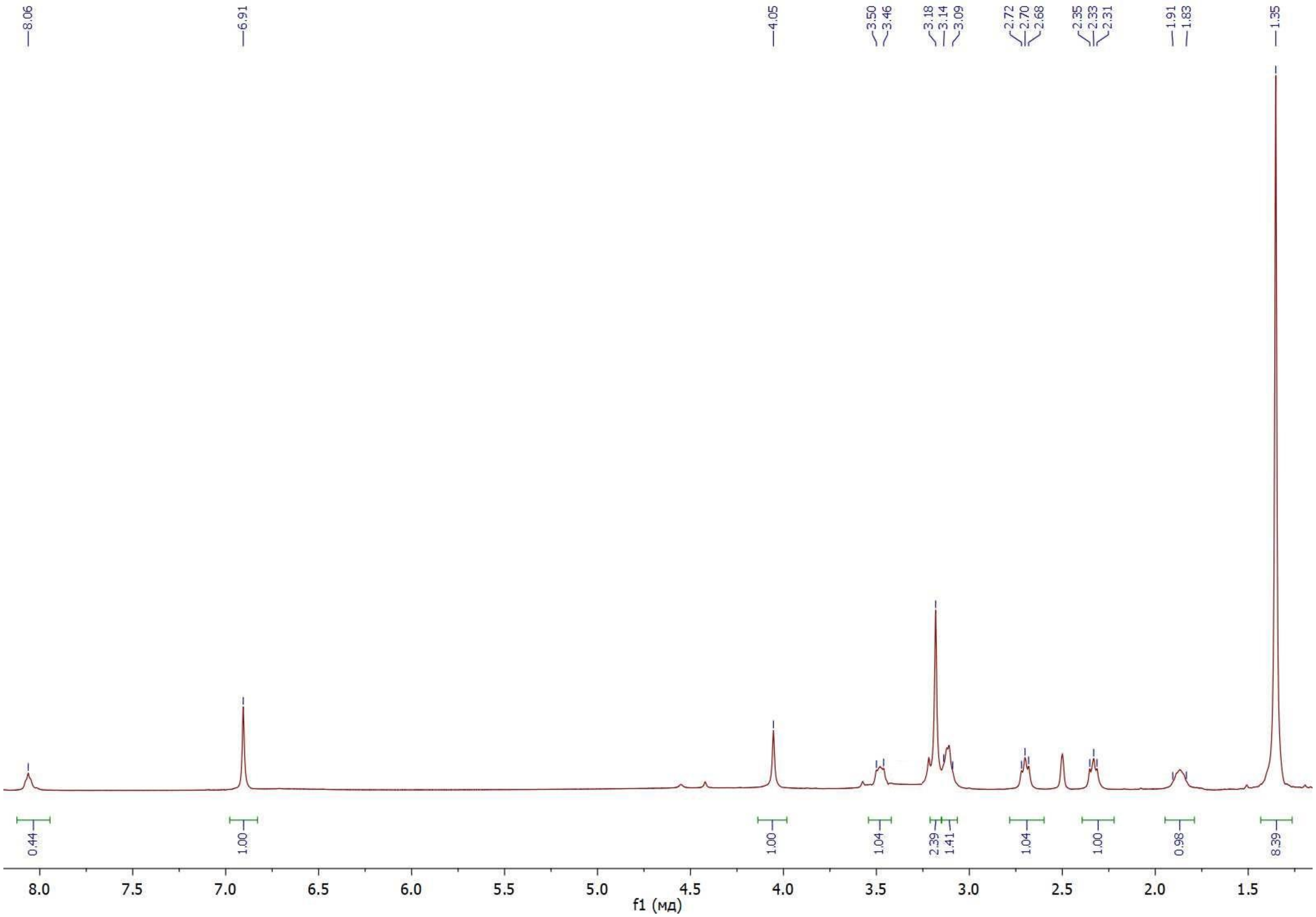

Figure S16. ^{1}H NMR spectrum of compound **1f** (400 MHz, DMSO-d_6)

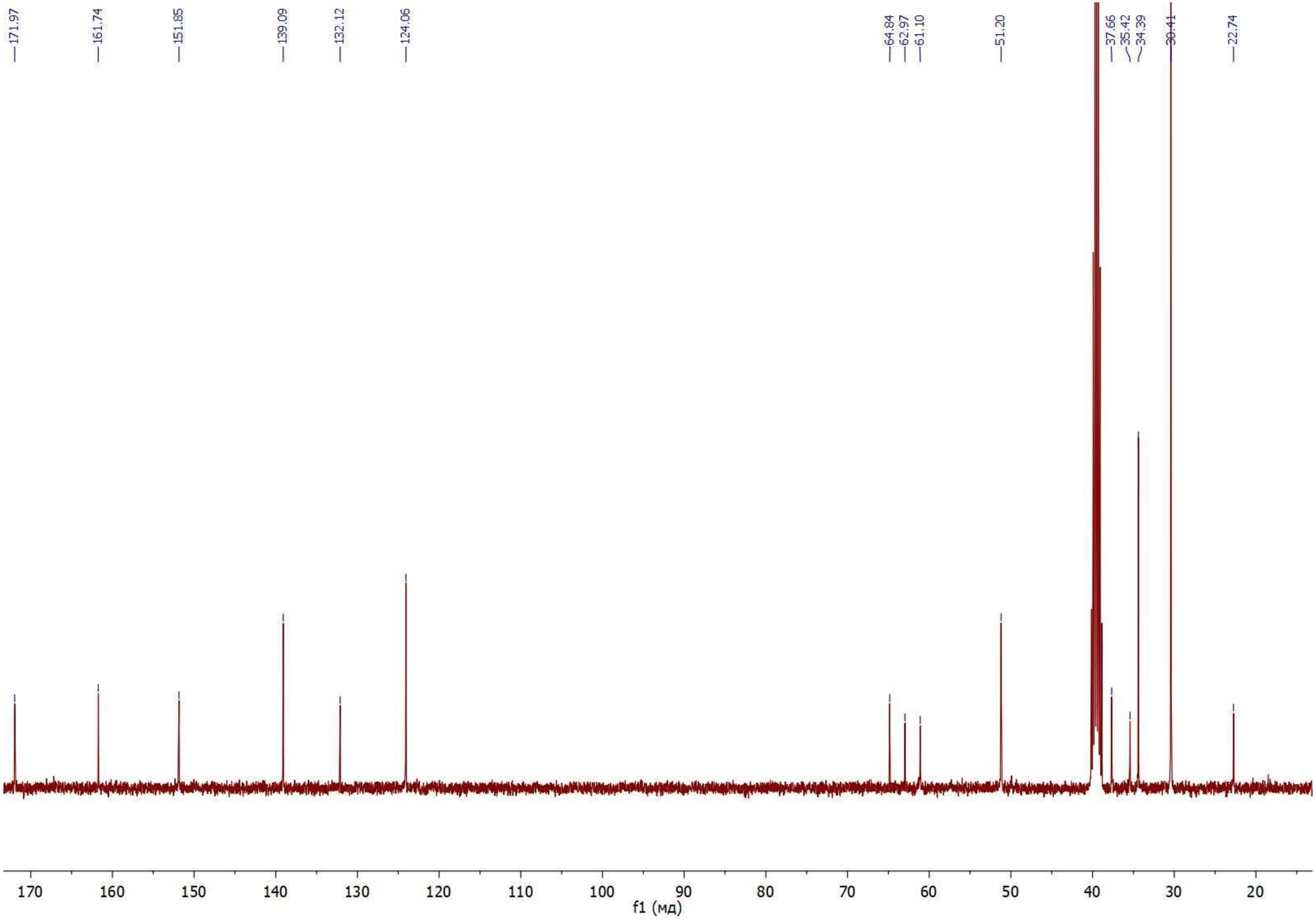

Figure S17. ^{13}C-{^{1}H} NMR spectrum of compound **1f** (101 MHz, DMSO-d_6)

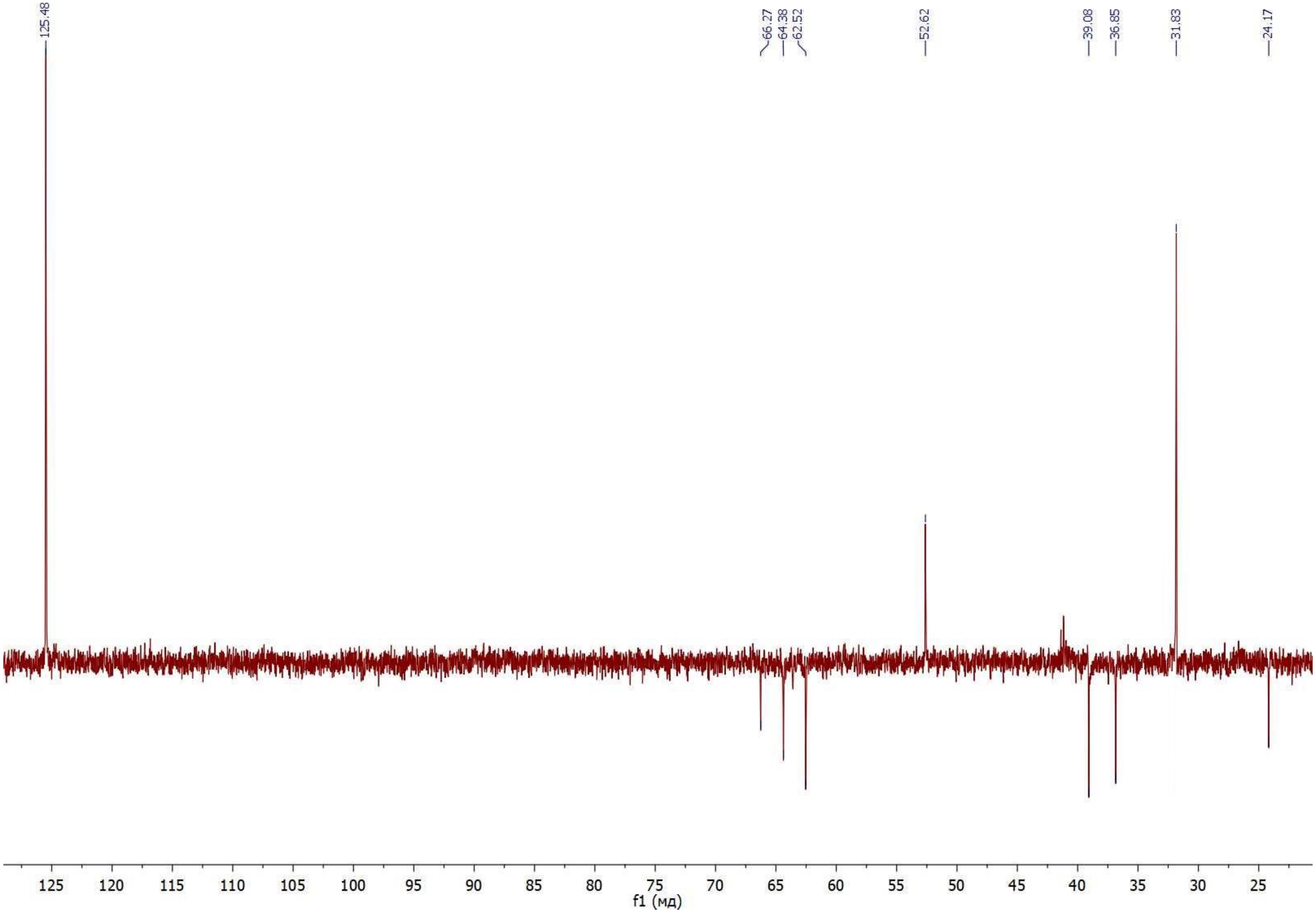

Figure S18. ^{13}C (dept) NMR spectrum of compound **1f** (101 MHz, DMSO-d_6)

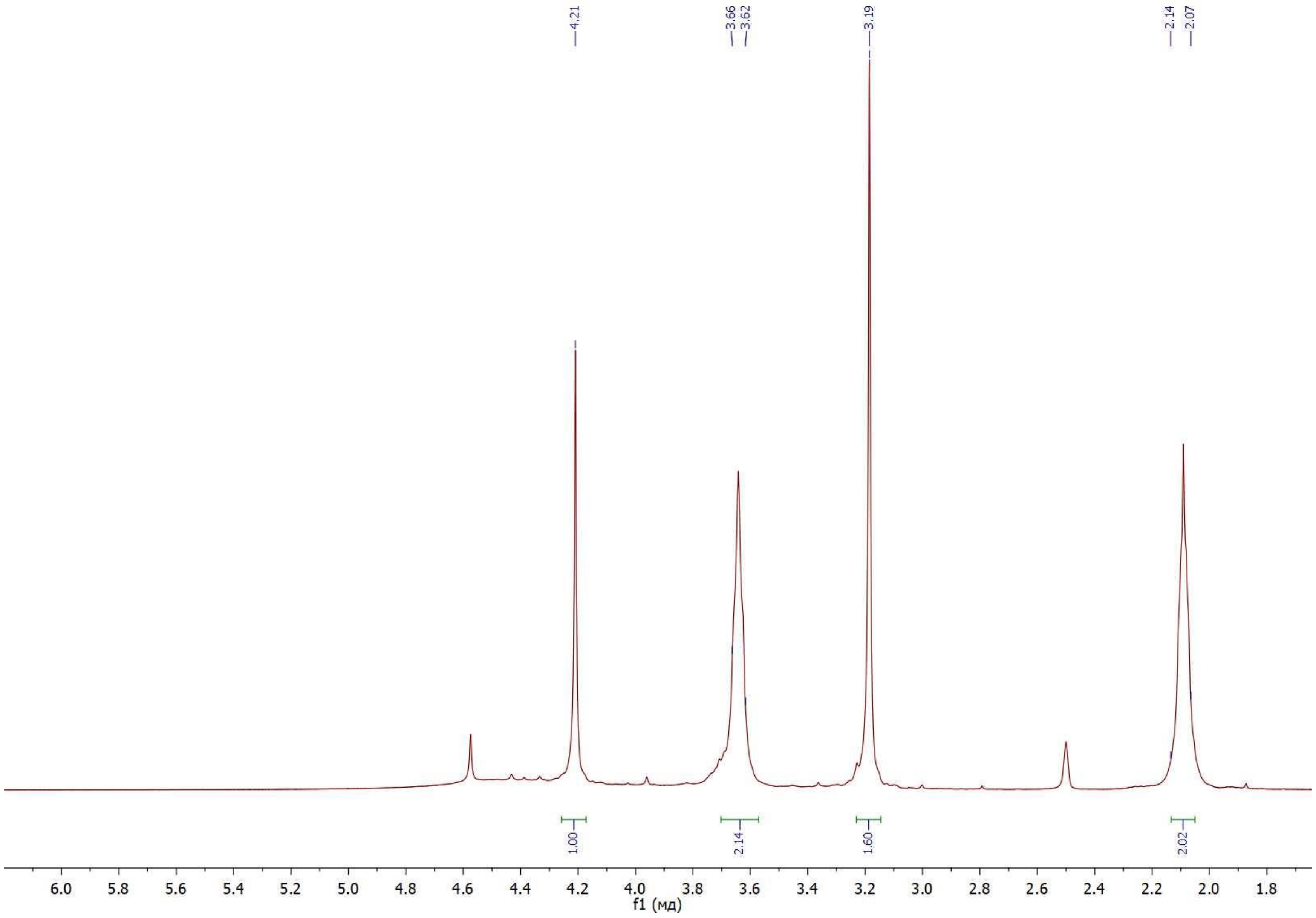

Figure S19. ^{1}H NMR spectrum of compound **1g** (400 MHz, DMSO-d_6)

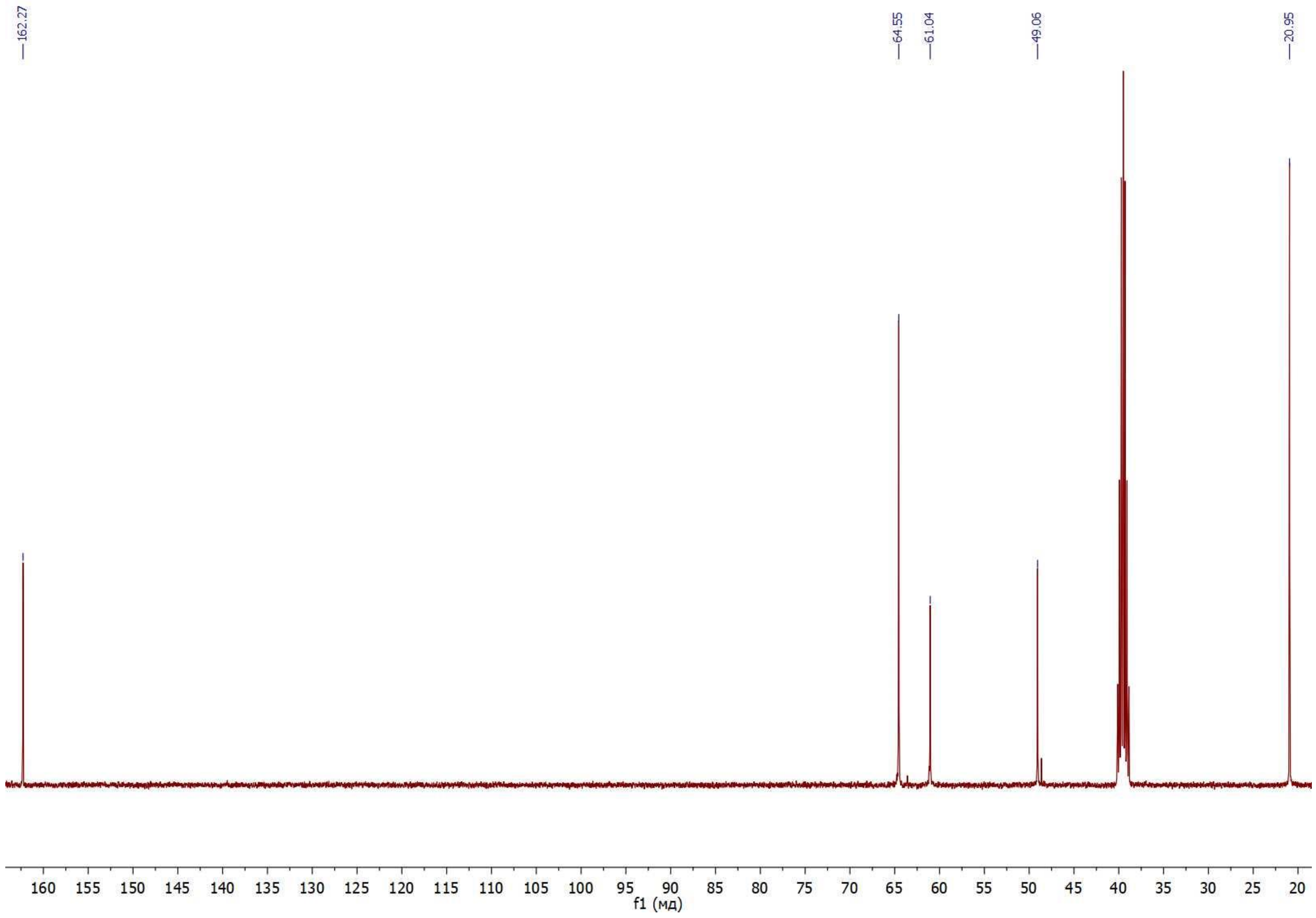

Figure S20. ^{13}C-{^{1}H} NMR spectrum of compound **1g** (101 MHz, DMSO-d_6)

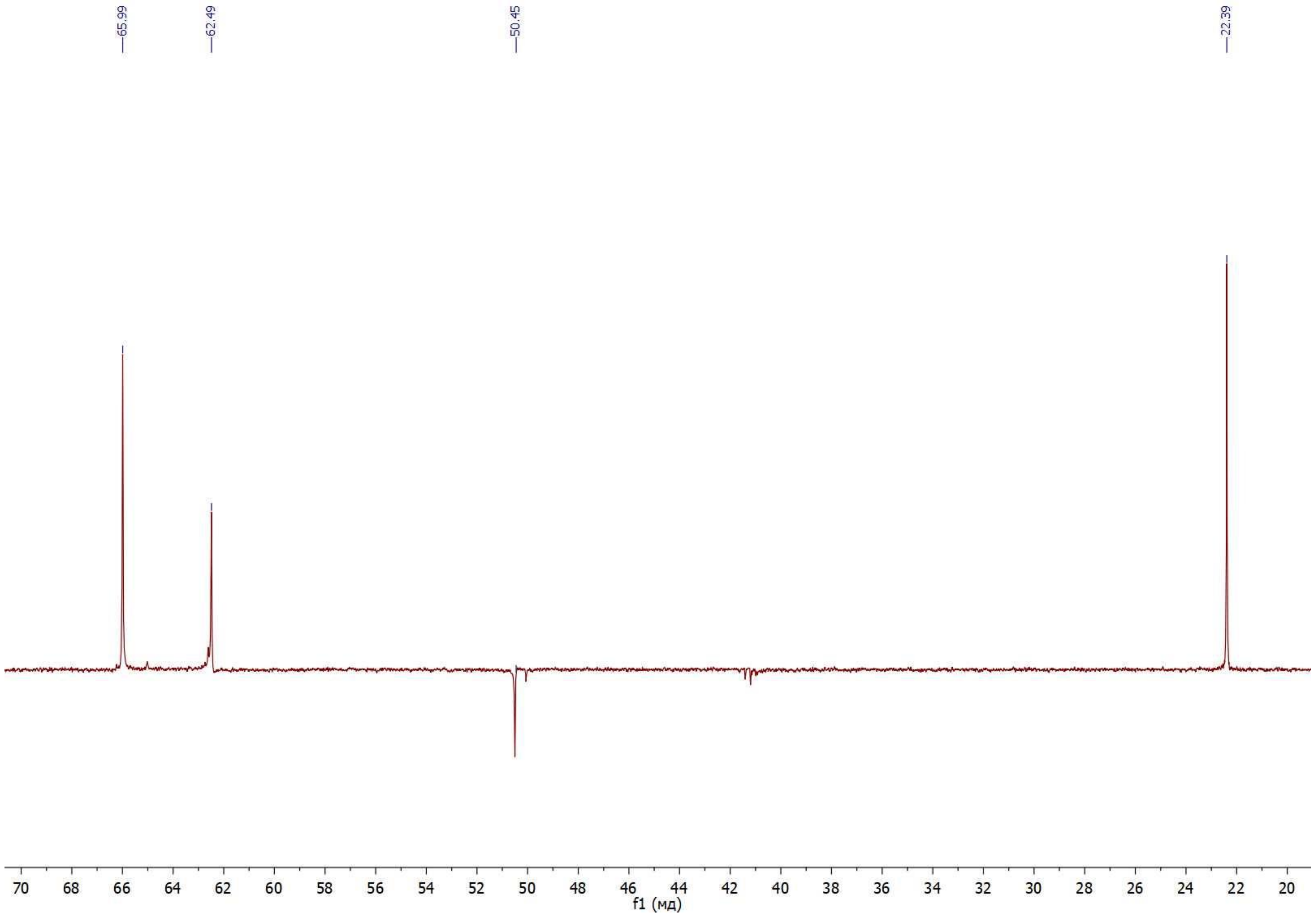

Figure S21. ^{13}C (dept) NMR spectrum of compound **1g** (101 MHz, DMSO-d_6)

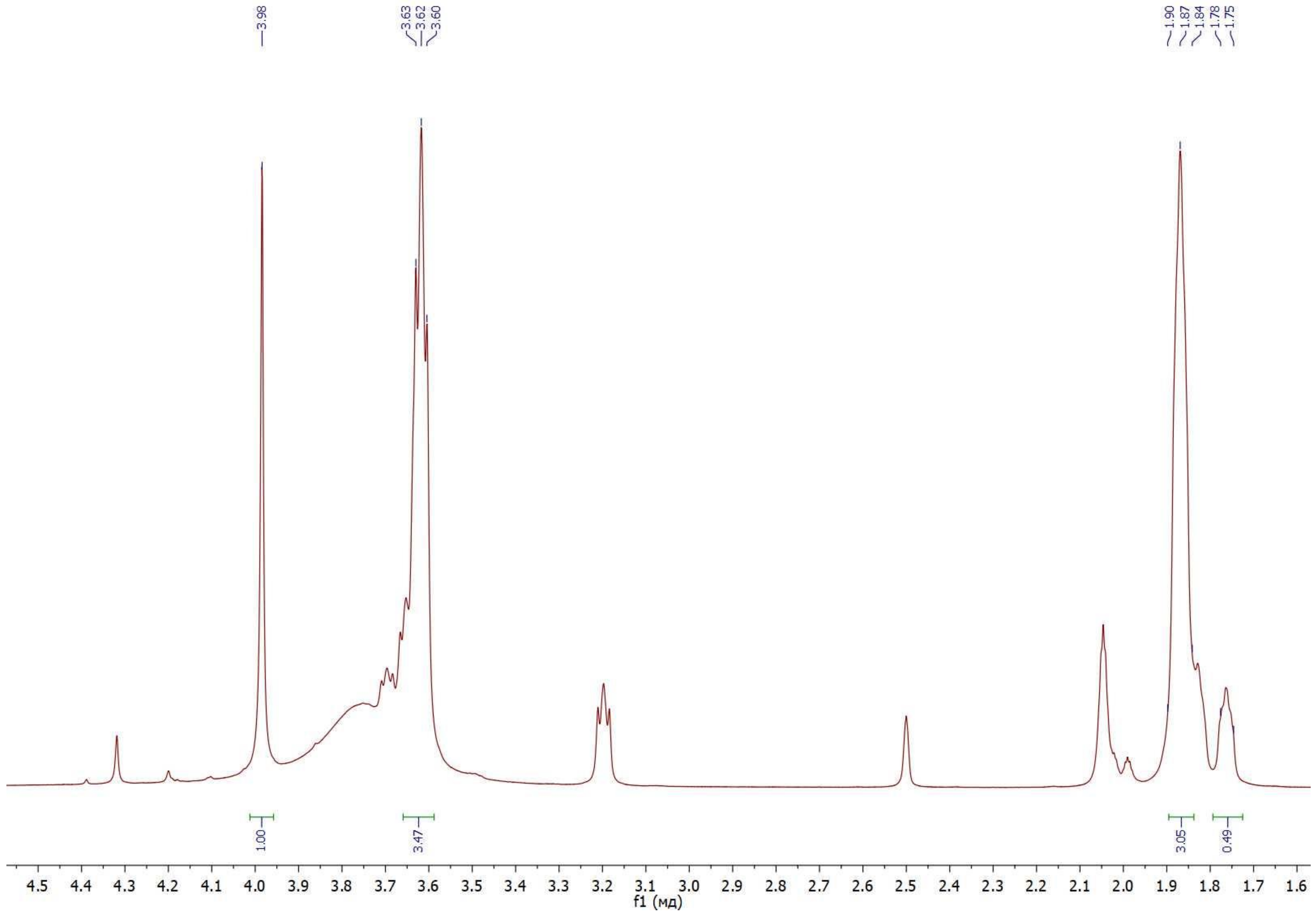

Figure S22. ^{1}H NMR spectrum of compound **1h** (600 MHz, DMSO-d_6)

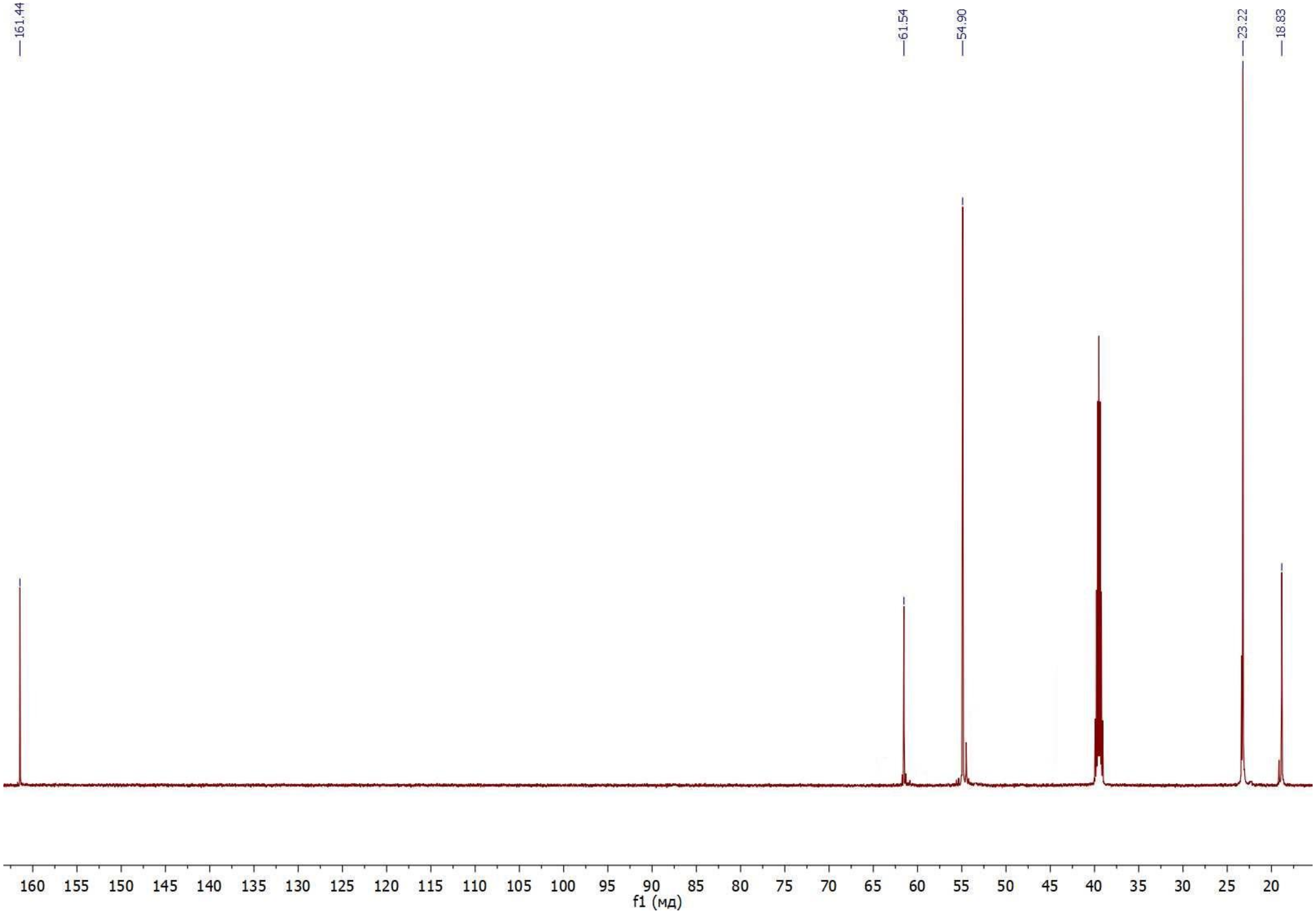

Figure S23. ^{13}C-{^1H} NMR spectrum of compound **1h** (101 MHz, DMSO-d_6)

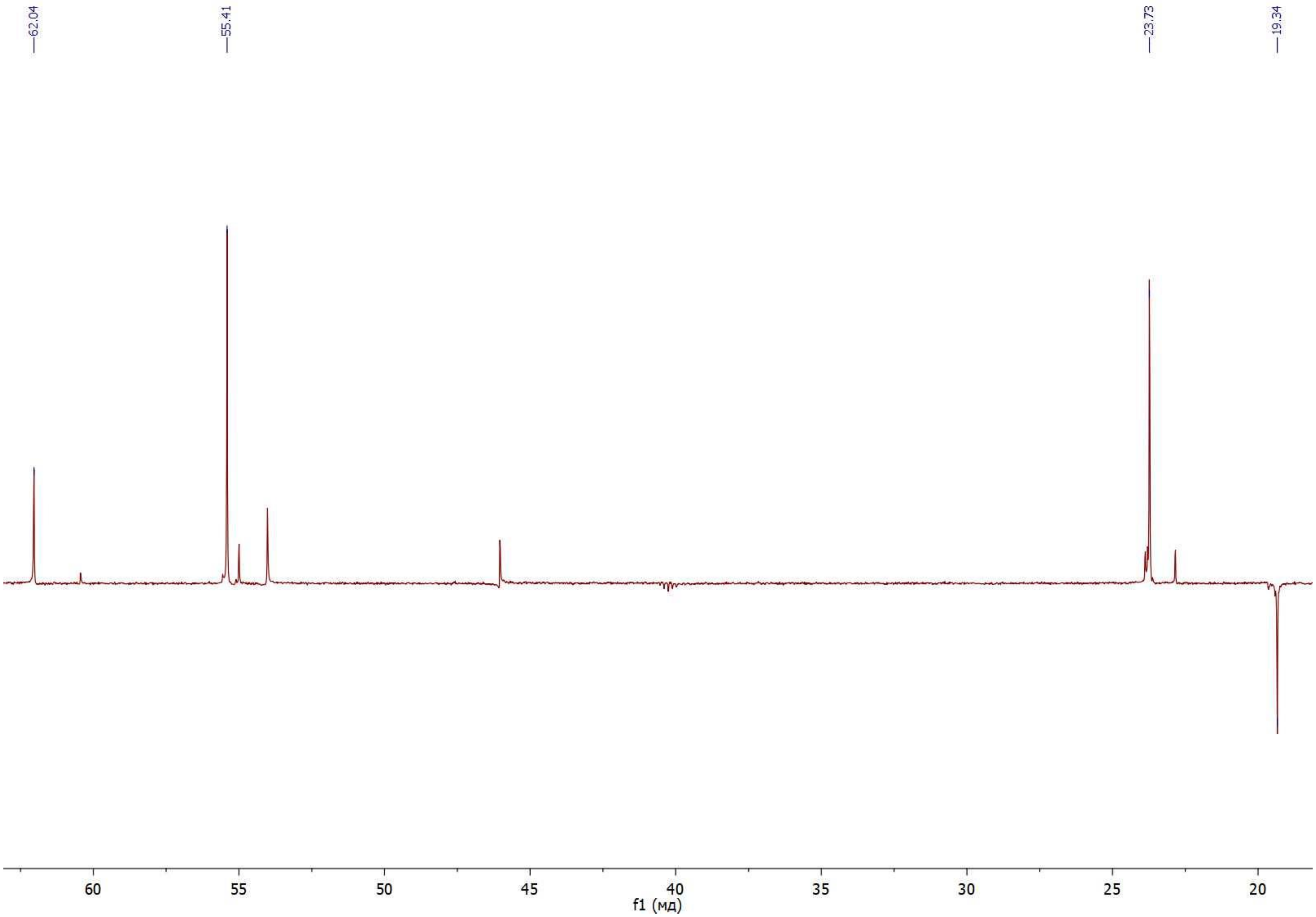

Figure S24. ^{13}C (dept) NMR spectrum of compound **1h** (101 MHz, DMSO-d_6)

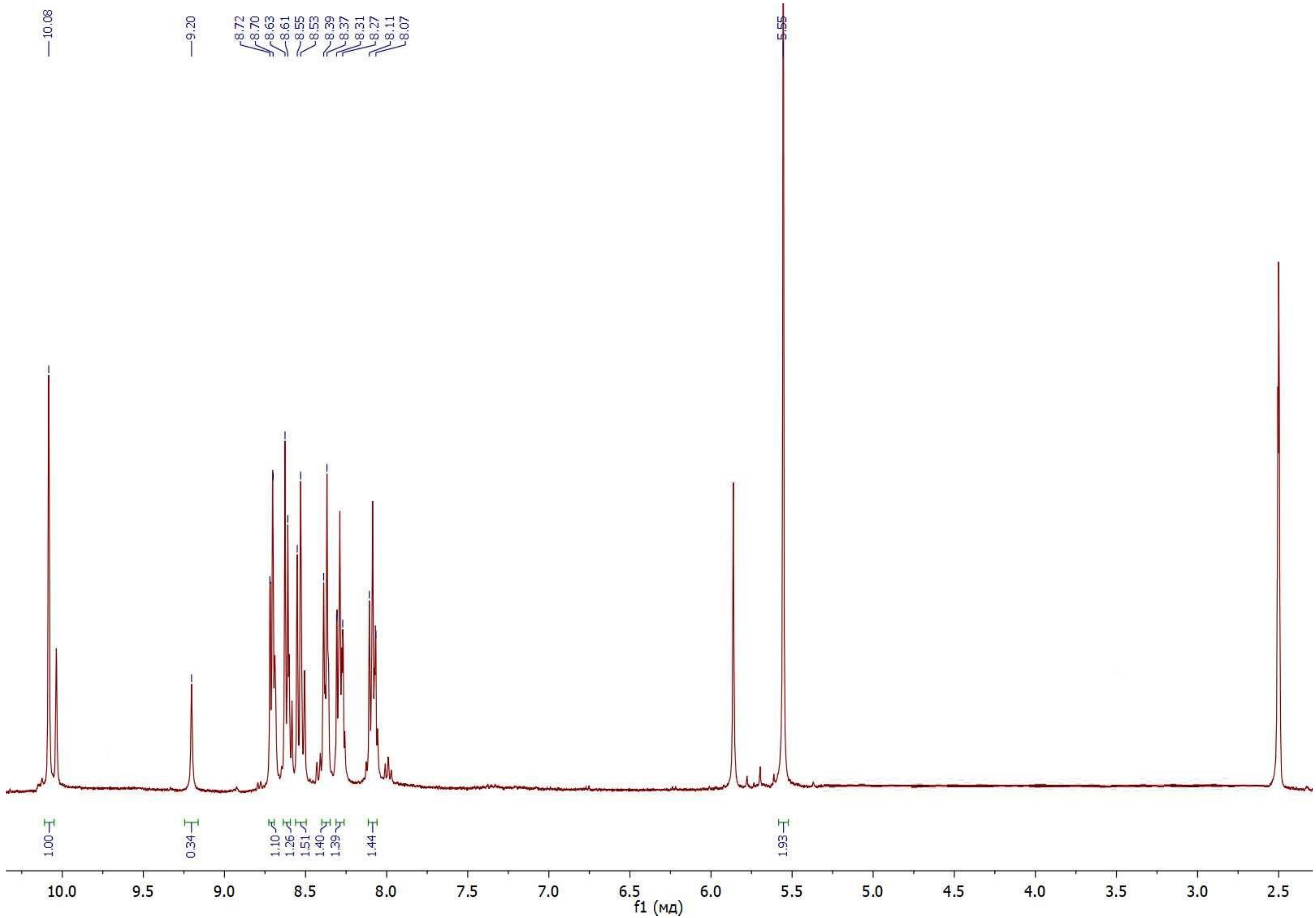

Figure S25. ^{1}H NMR spectrum of compound **1i** (400 MHz, DMSO-d_6)

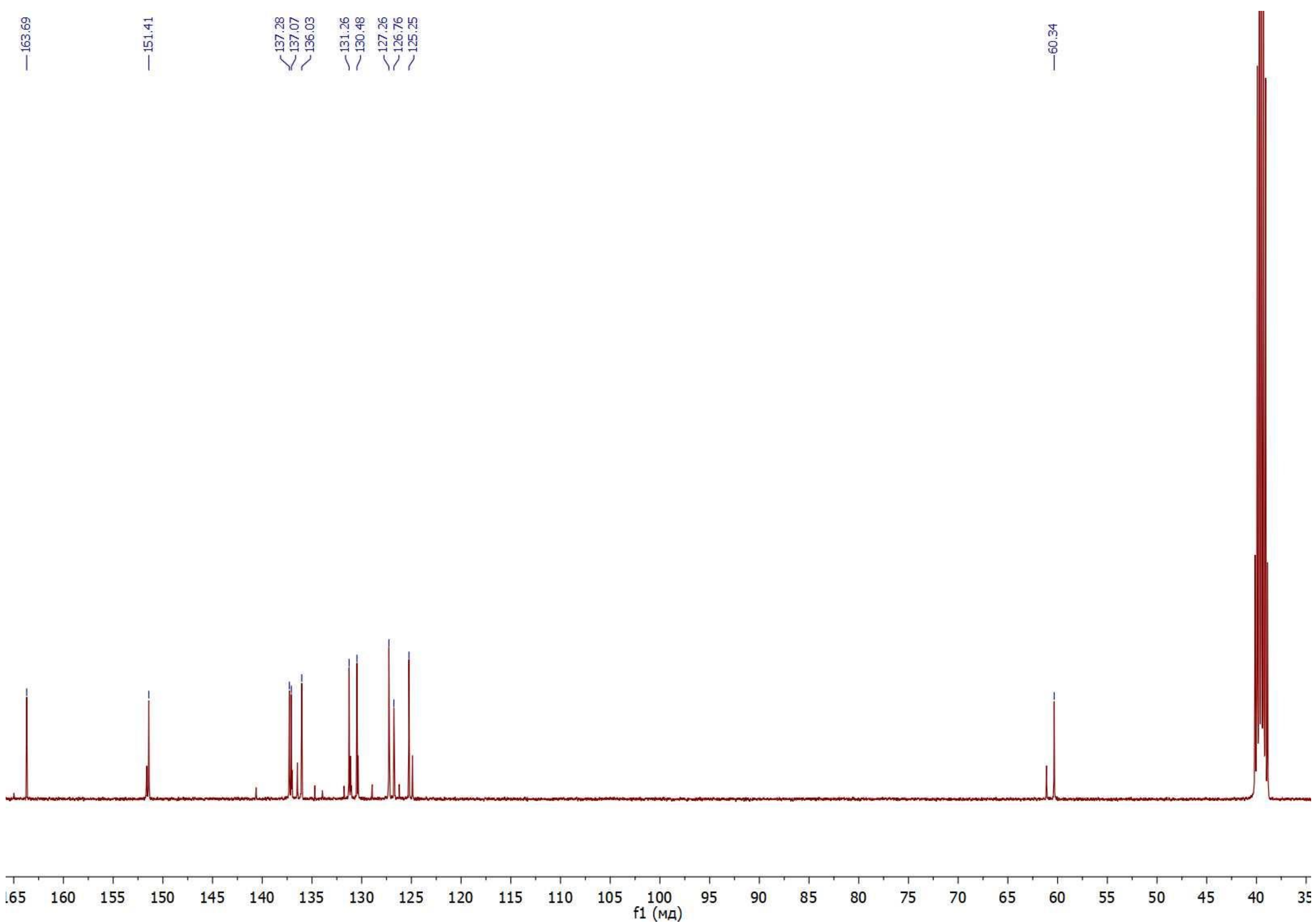

Figure S26. ^{13}C-{^{1}H} NMR spectrum of compound **1i** (101 MHz, DMSO-d_6)

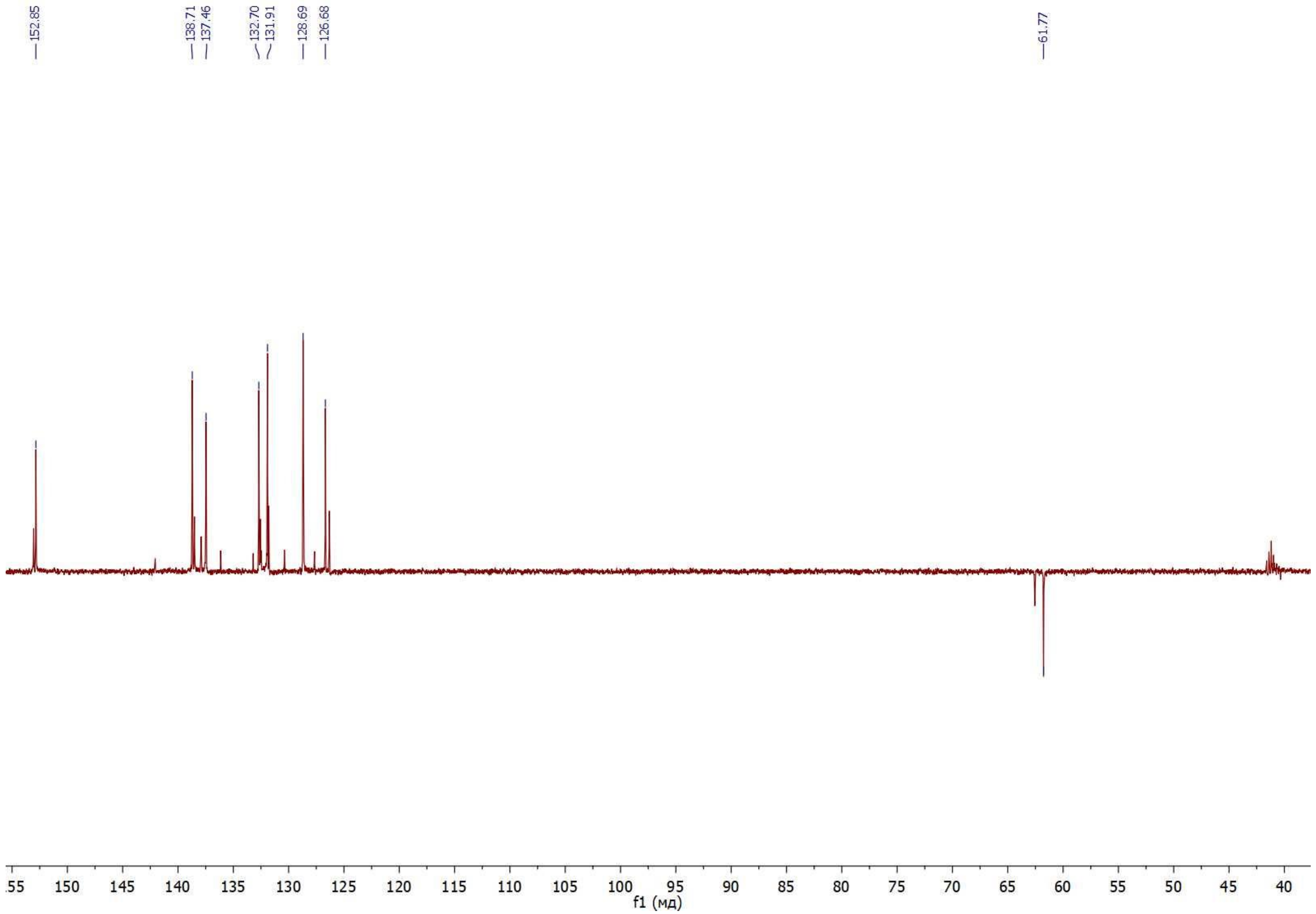

Figure S27. ^{13}C (dept) NMR spectrum of compound **1i** (101 MHz, DMSO-d_6)

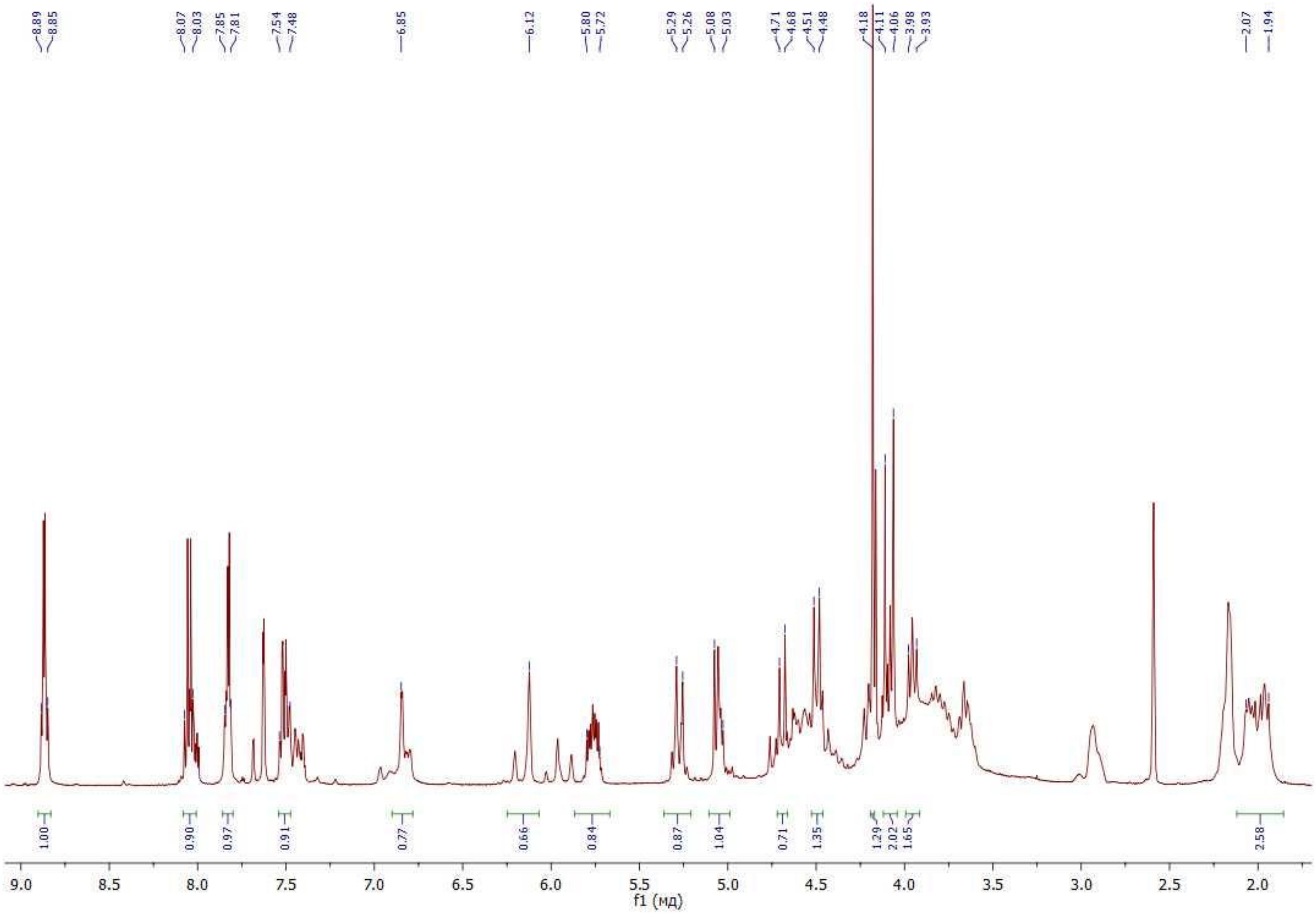

Figure S28. ^{1}H NMR spectrum of compound **1j** (500 MHz, DMSO-d_6)

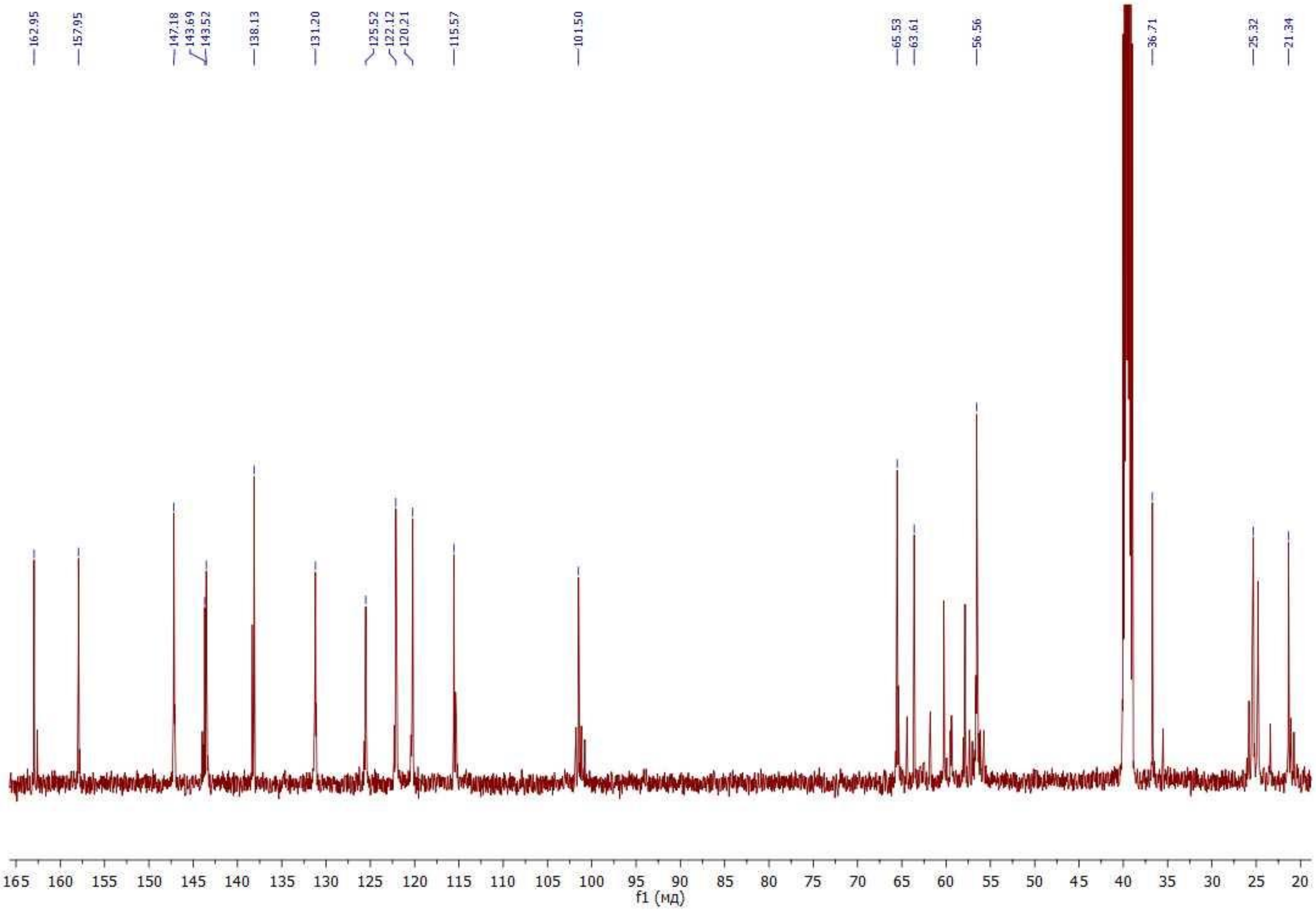

Figure S29. ^{13}C-{^{1}H} NMR spectrum of compound **1j** (126 MHz, DMSO-d_6)

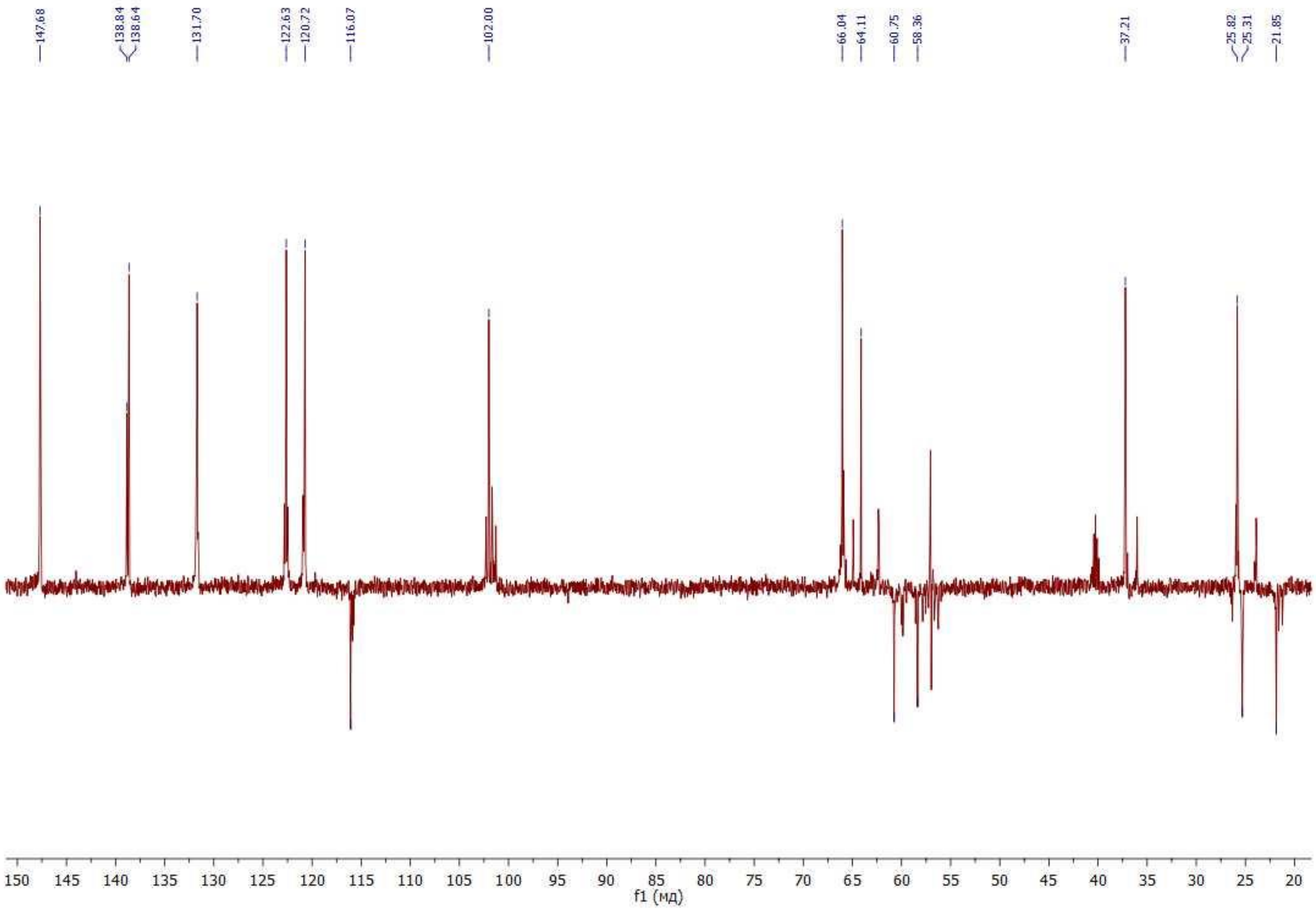

Figure S30. ^{13}C (dept) NMR spectrum of compound **1j** (126 MHz, DMSO-d_6)

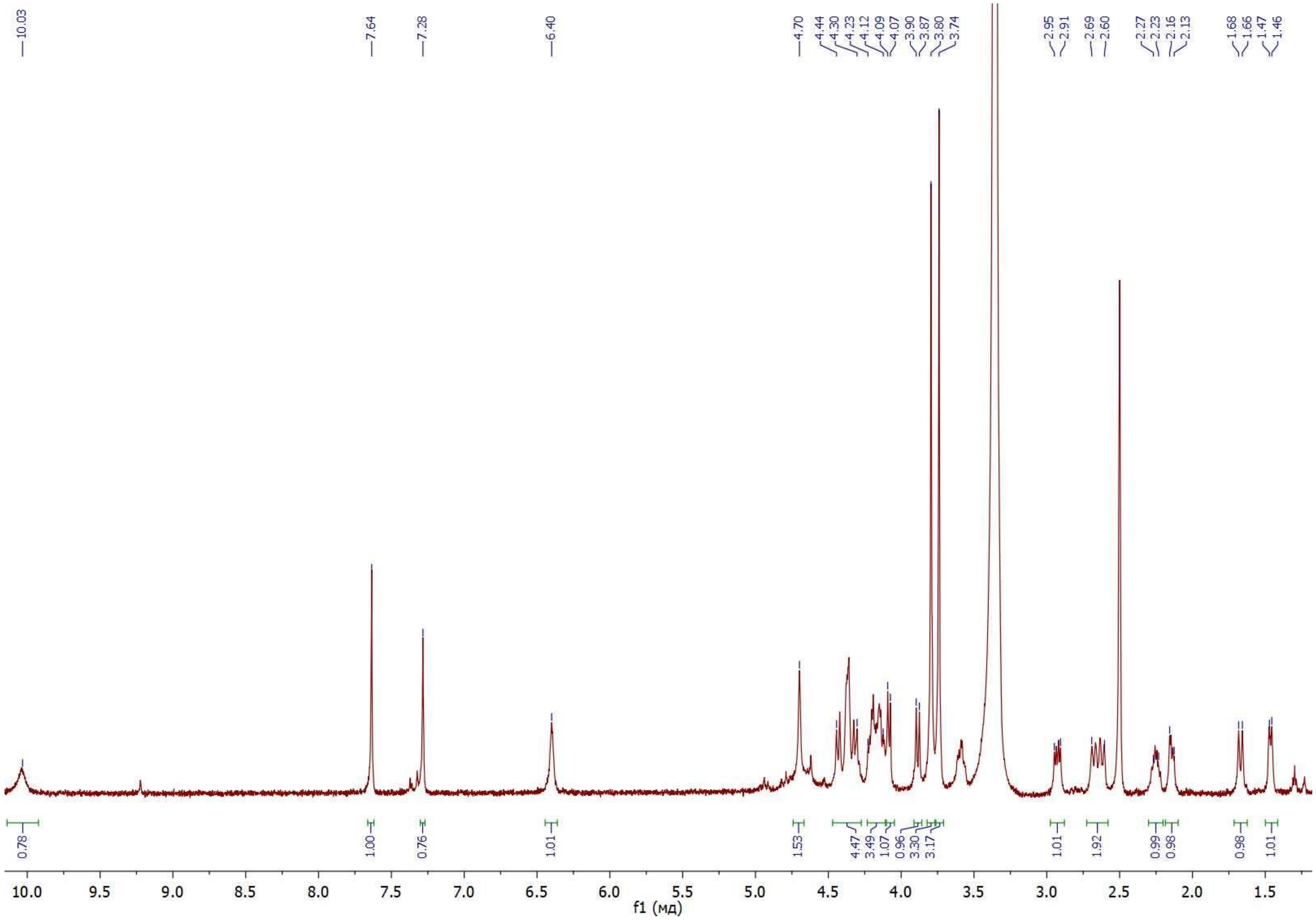

Figure S31. ^1H NMR spectrum of compound **1k** (400 MHz, DMSO-d_6)

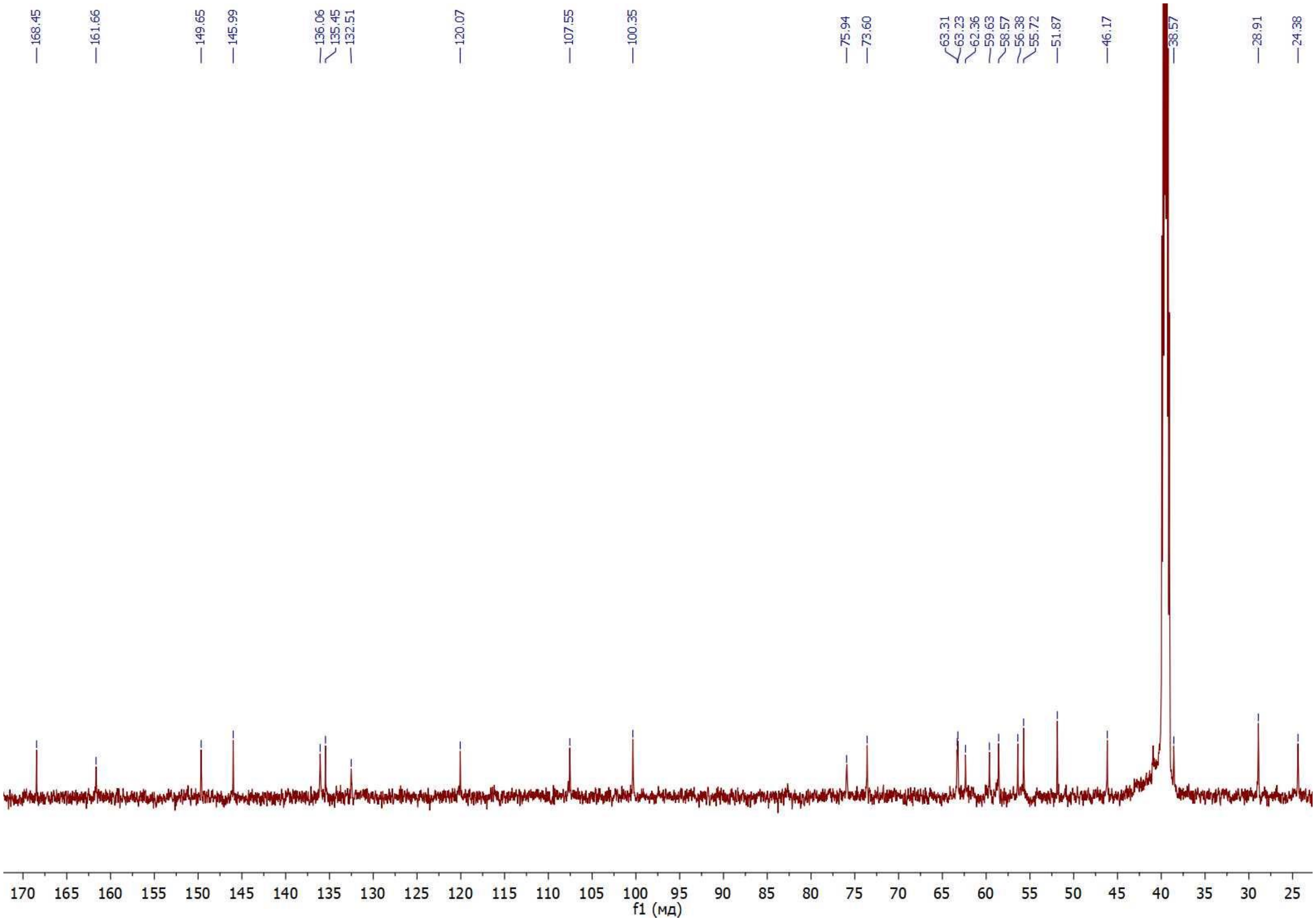

Figure S32. ^{13}C-{^{1}H} NMR spectrum of compound **1k** (101 MHz, DMSO-d_6)

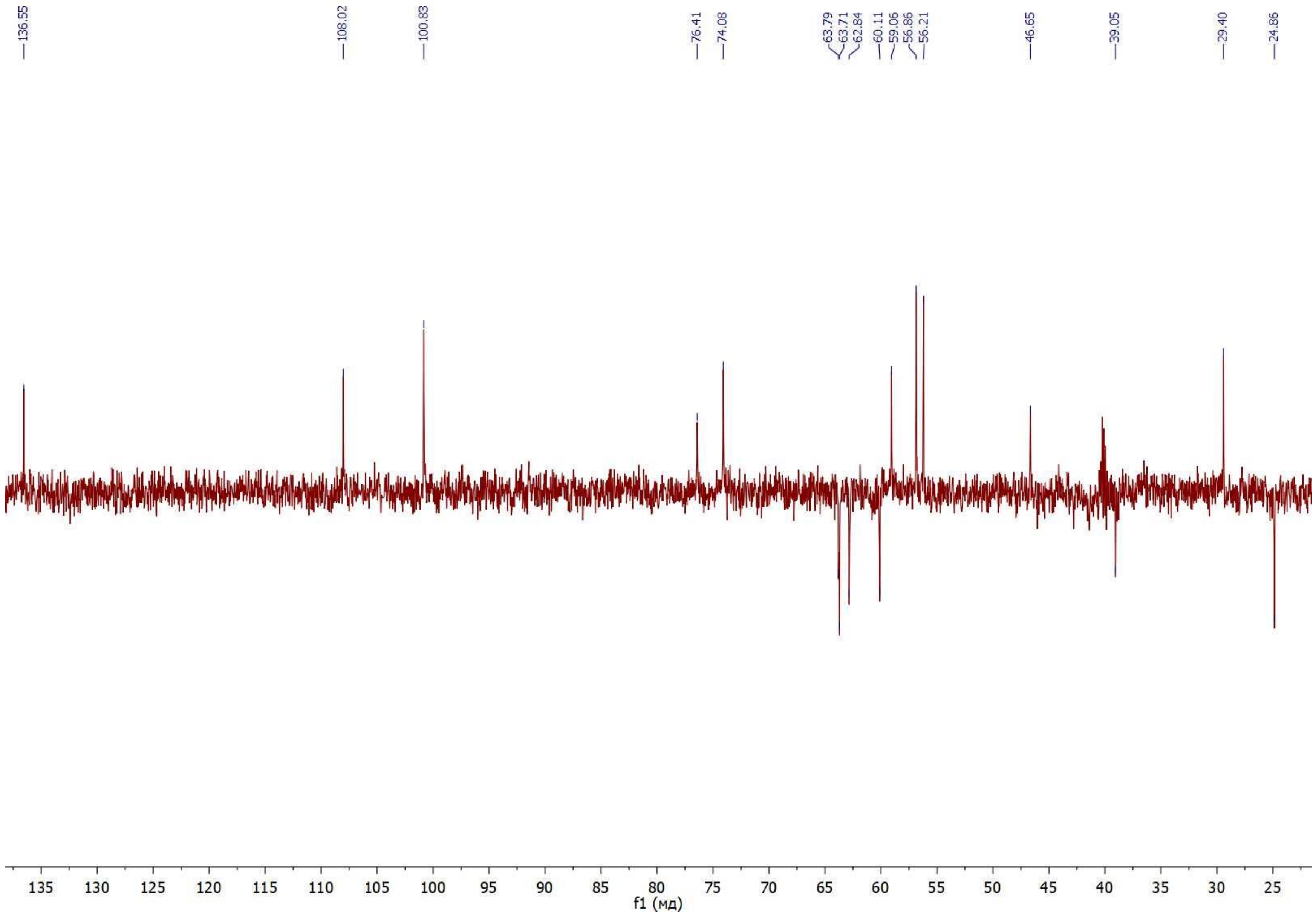

Figure S33. ^{13}C (dept) NMR spectrum of compound **1k** (101 MHz, DMSO-d_6)

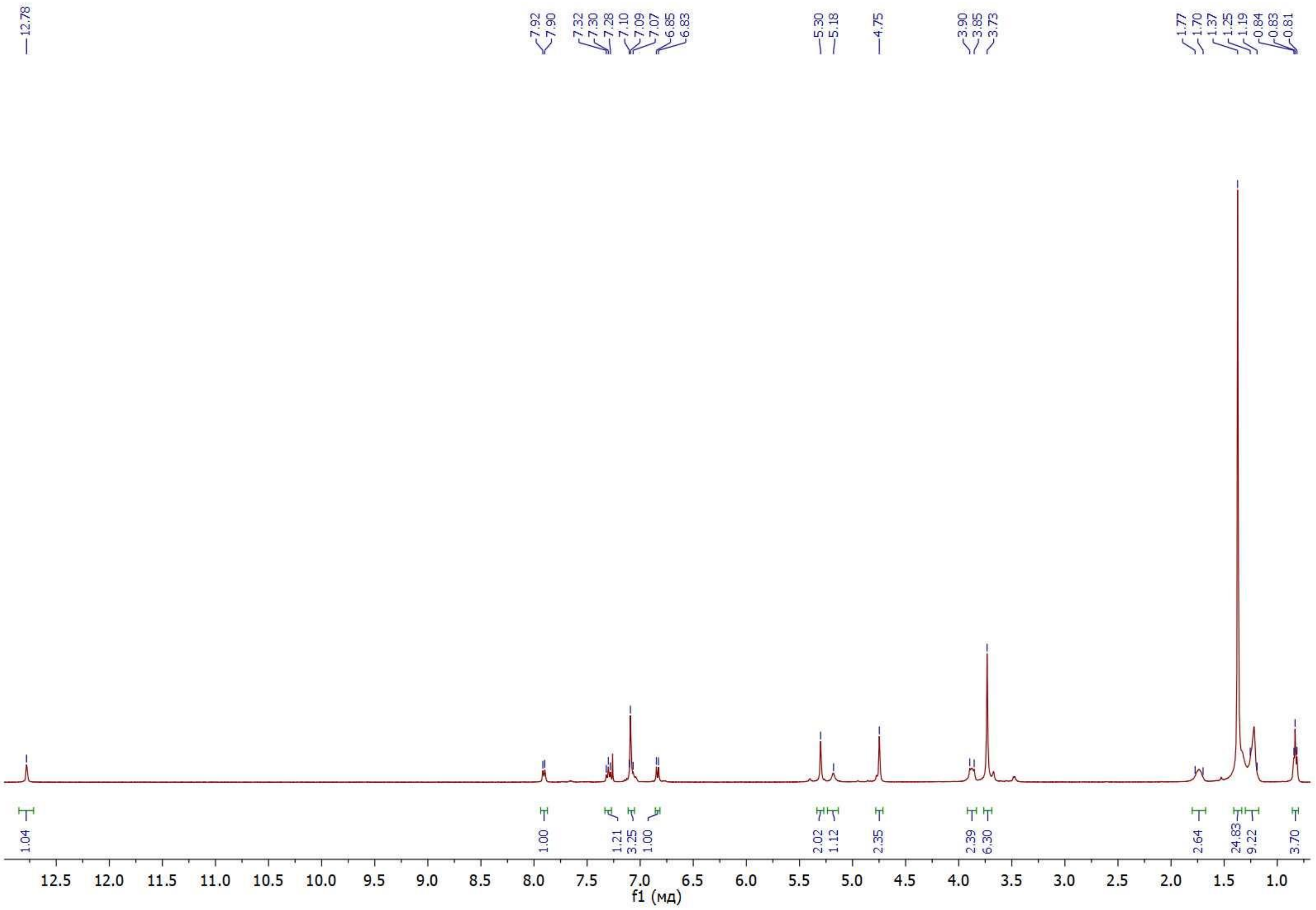

Figure S34. ^{1}H NMR spectrum of compound **3a** (600 MHz, $CDCl_3$)

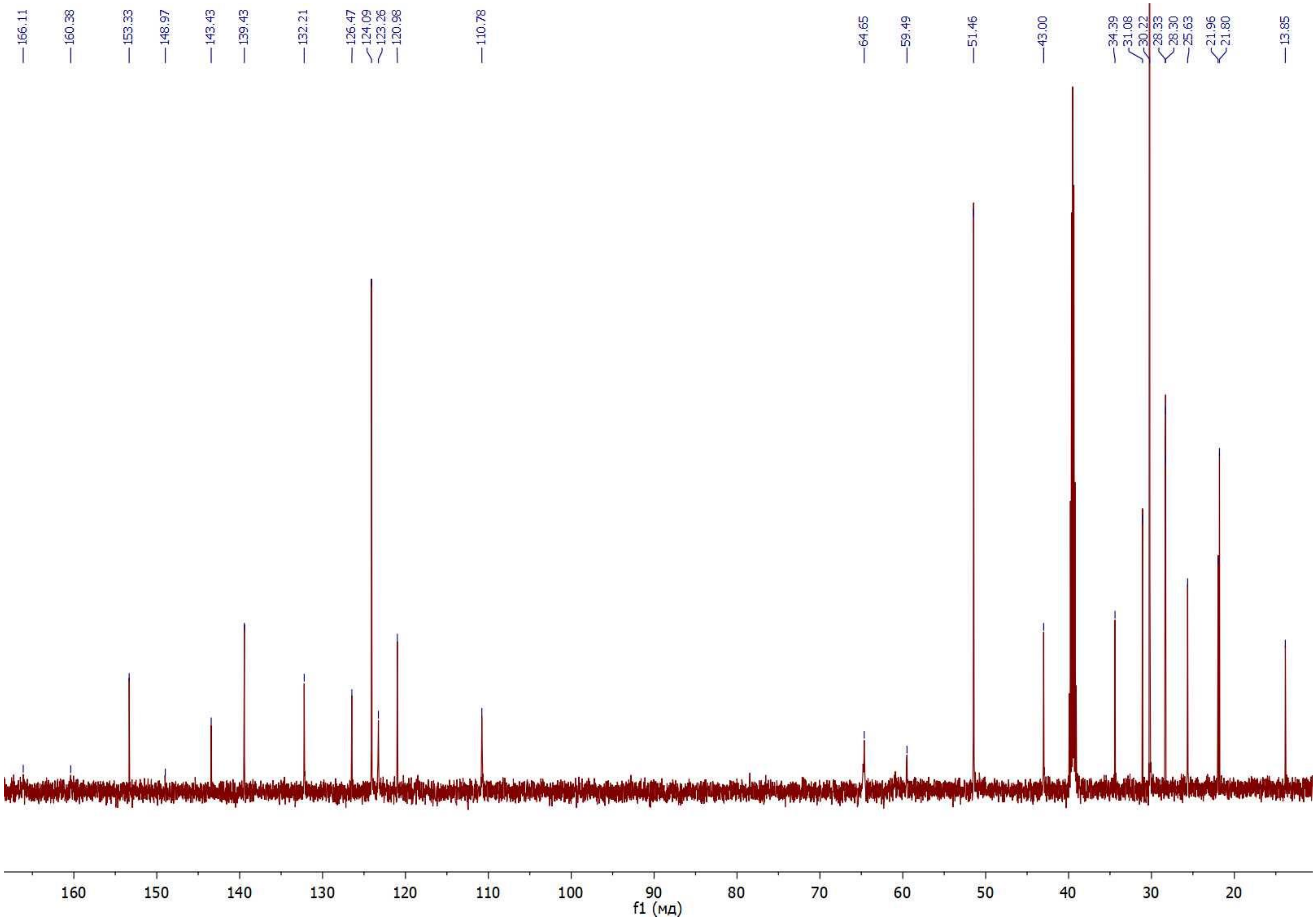

Figure S35. ^{13}C-{^{1}H} NMR spectrum of compound **3a** (151 MHz, DMSO-d_6)

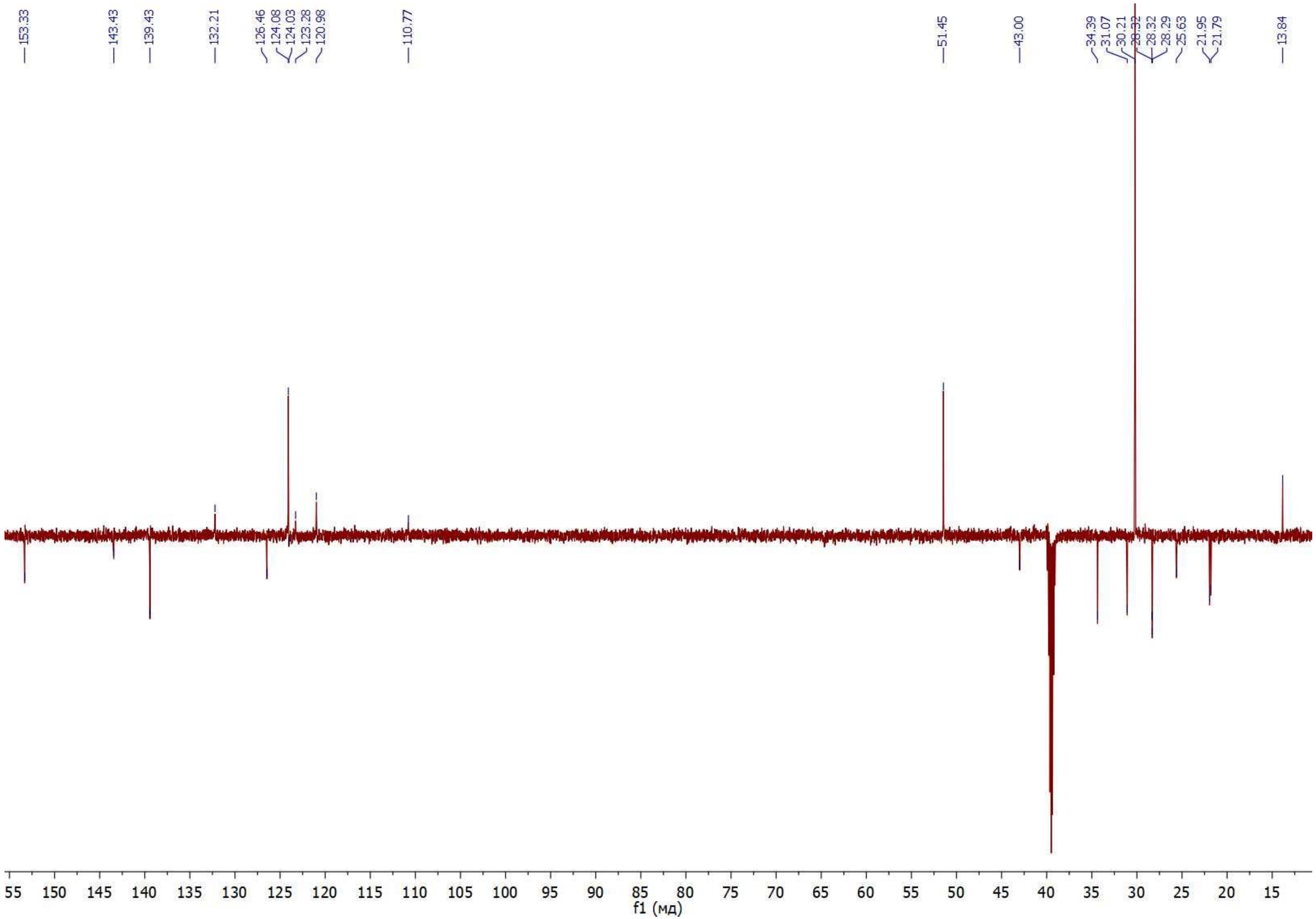

Figure S36. ^{13}C (JMOD) NMR spectrum of compound **3a** (151 MHz, DMSO-d_6)

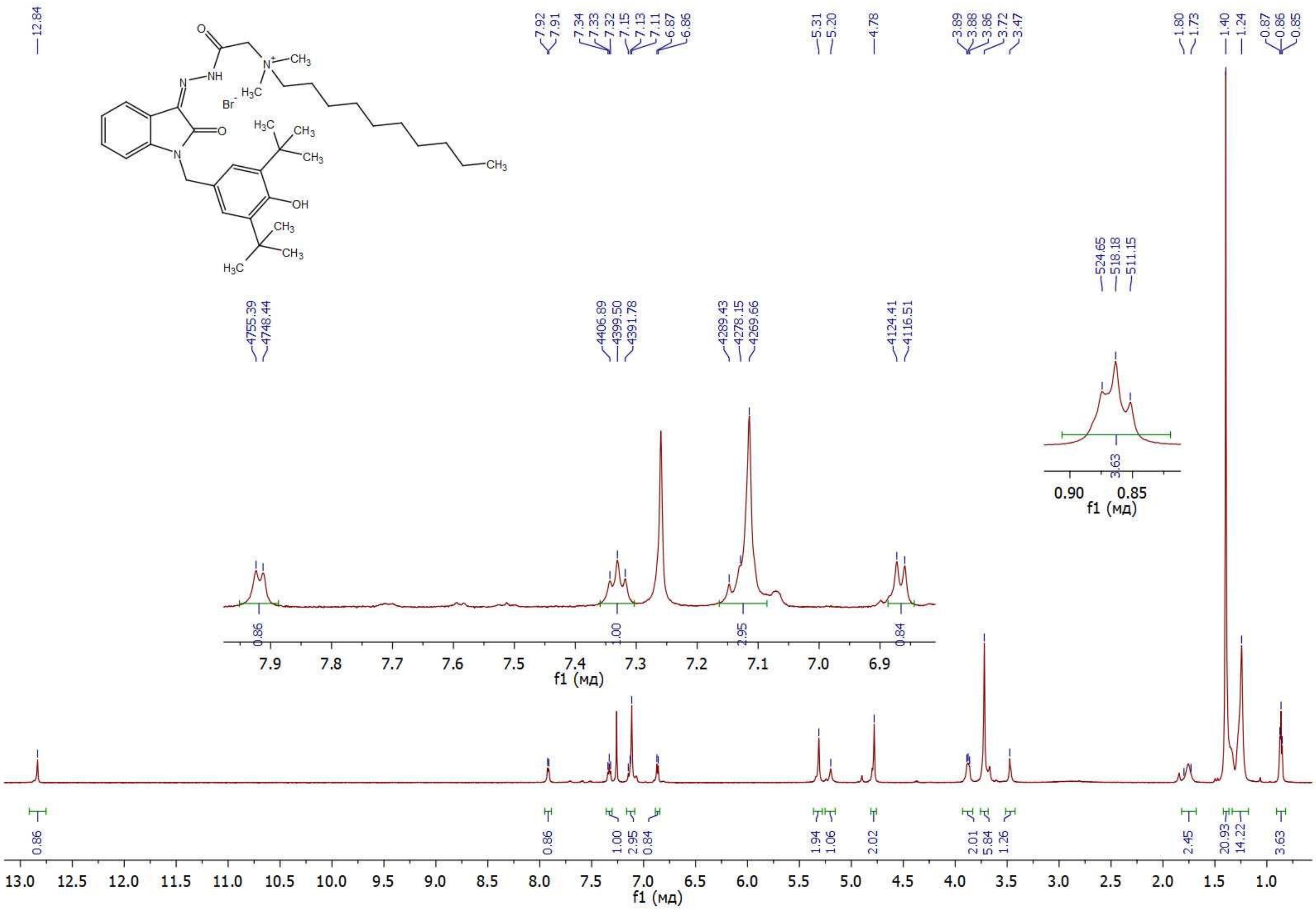

Figure S37. ^{1}H NMR spectrum of compound **3b** (600 MHz, $CDCl_3$)

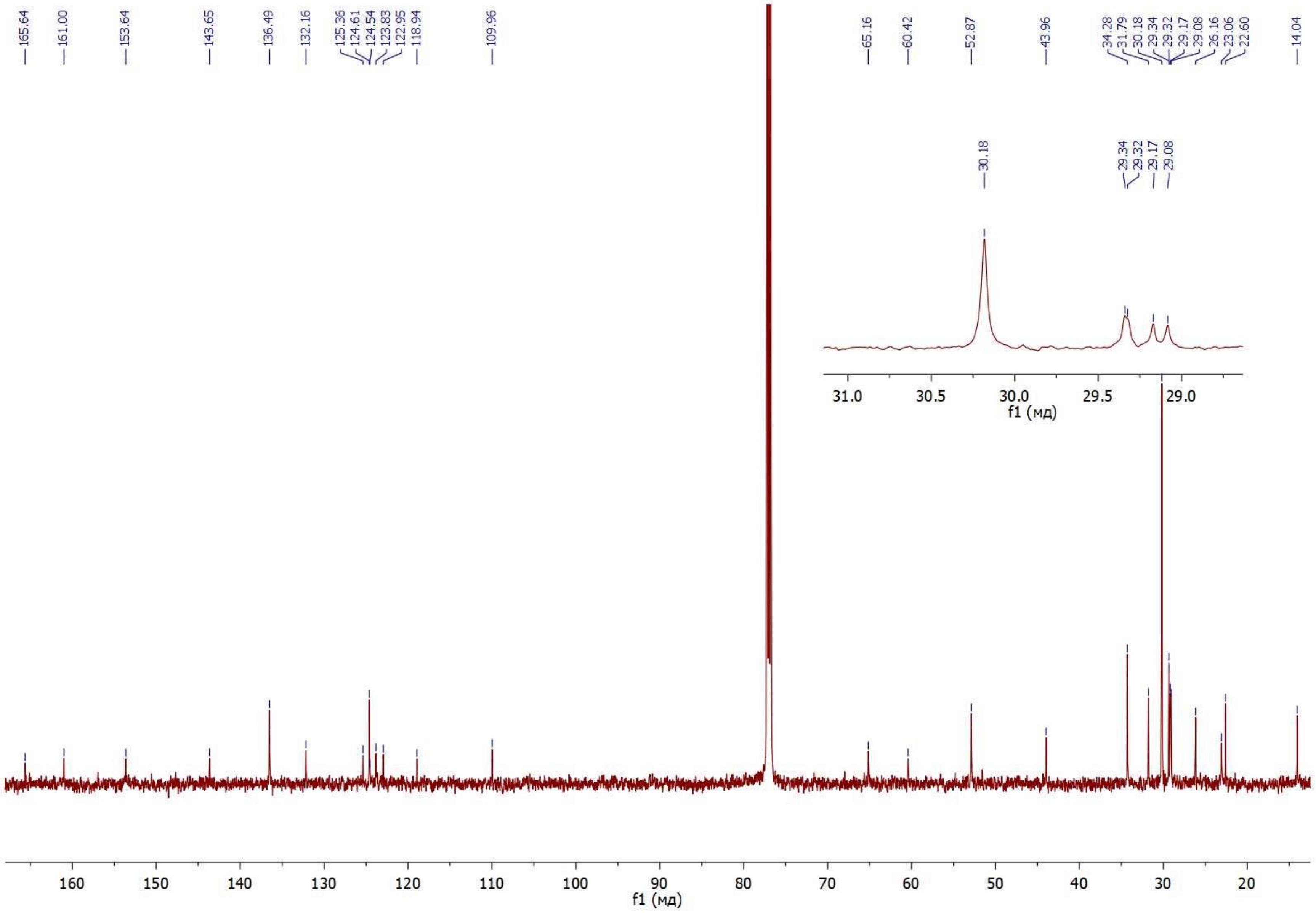

Figure S38. $^{13}C-\{^{1}H\}$ NMR spectrum of compound **3b** (151 MHz, $CDCl_3$)

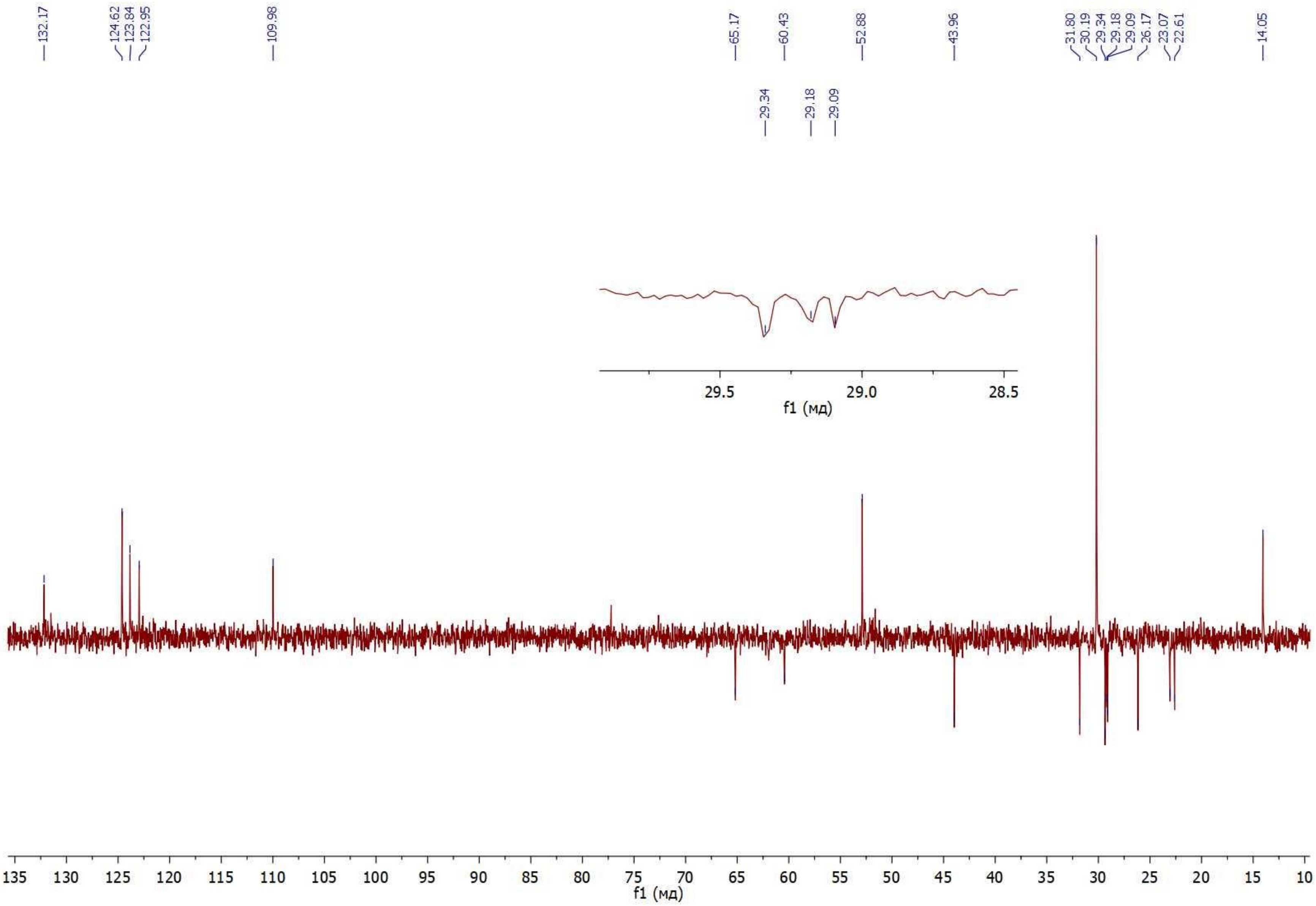

Figure S39. ^{13}C (dept) NMR spectrum of compound **3b** (151 MHz, $CDCl_3$)

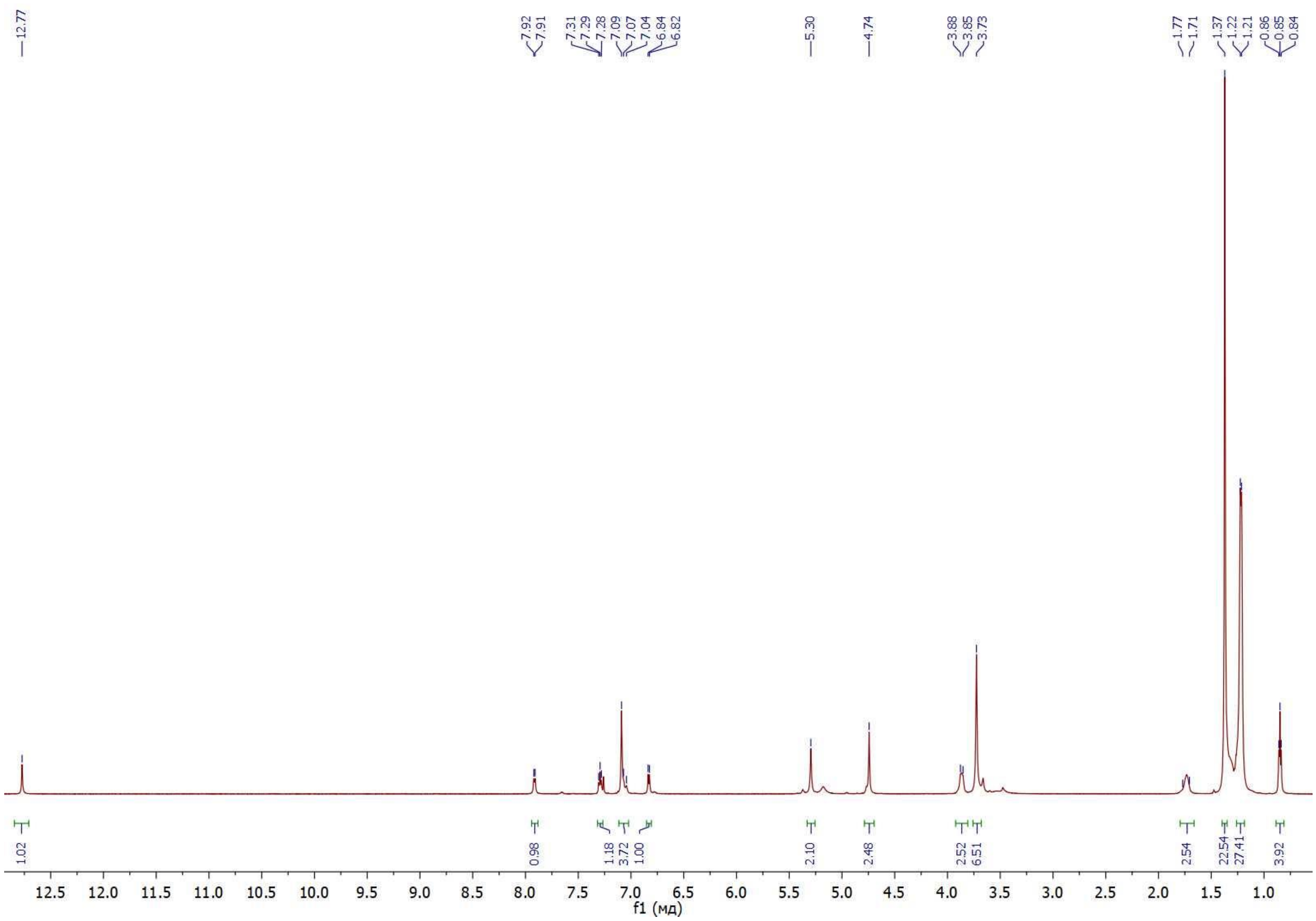

Figure S40. ^{1}H NMR spectrum of compound **3c** (400 MHz, $CDCl_3$)

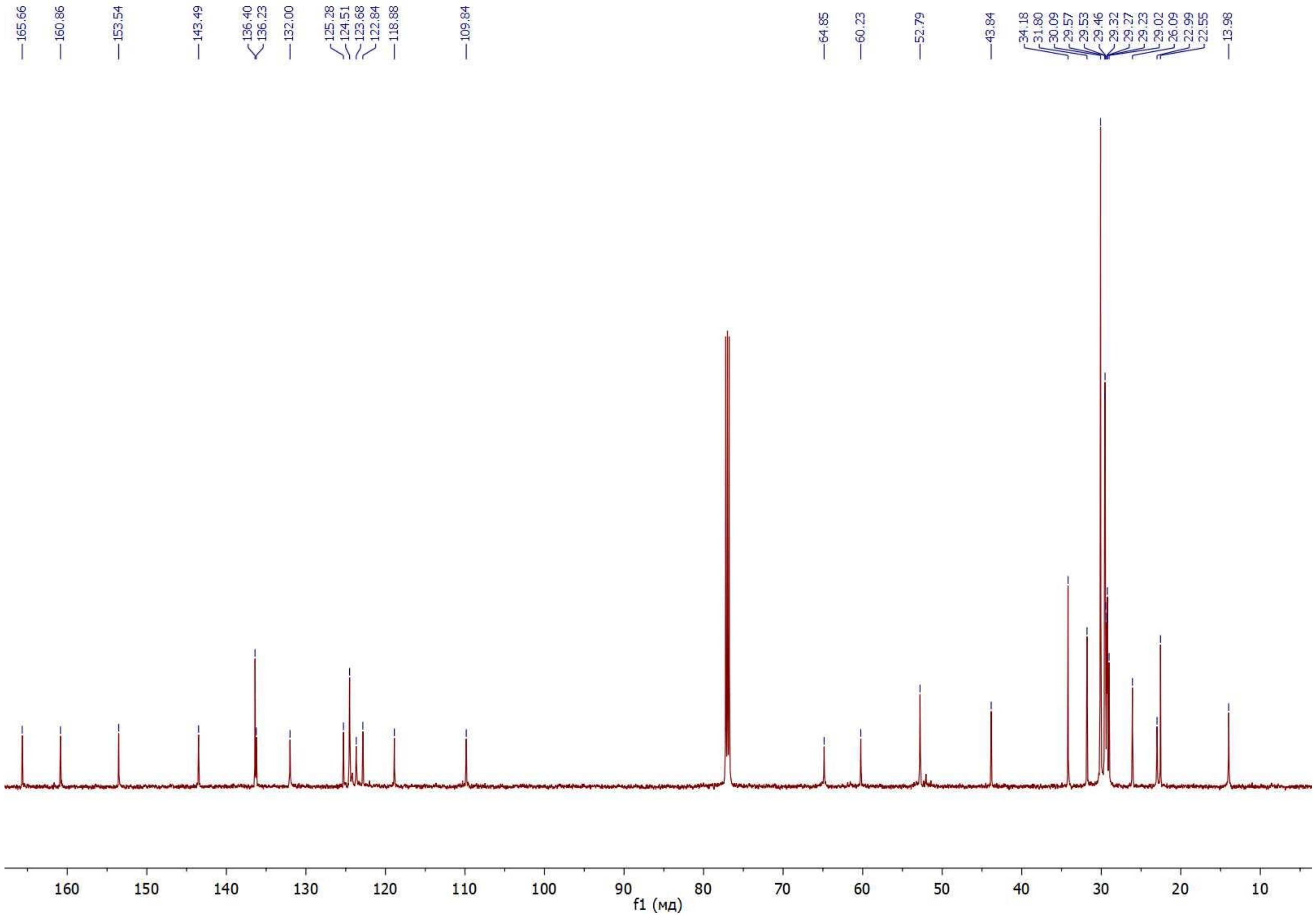

Figure S41. ^{13}C-{^{1}H} NMR spectrum of compound **3c** (101 MHz, $CDCl_3$)

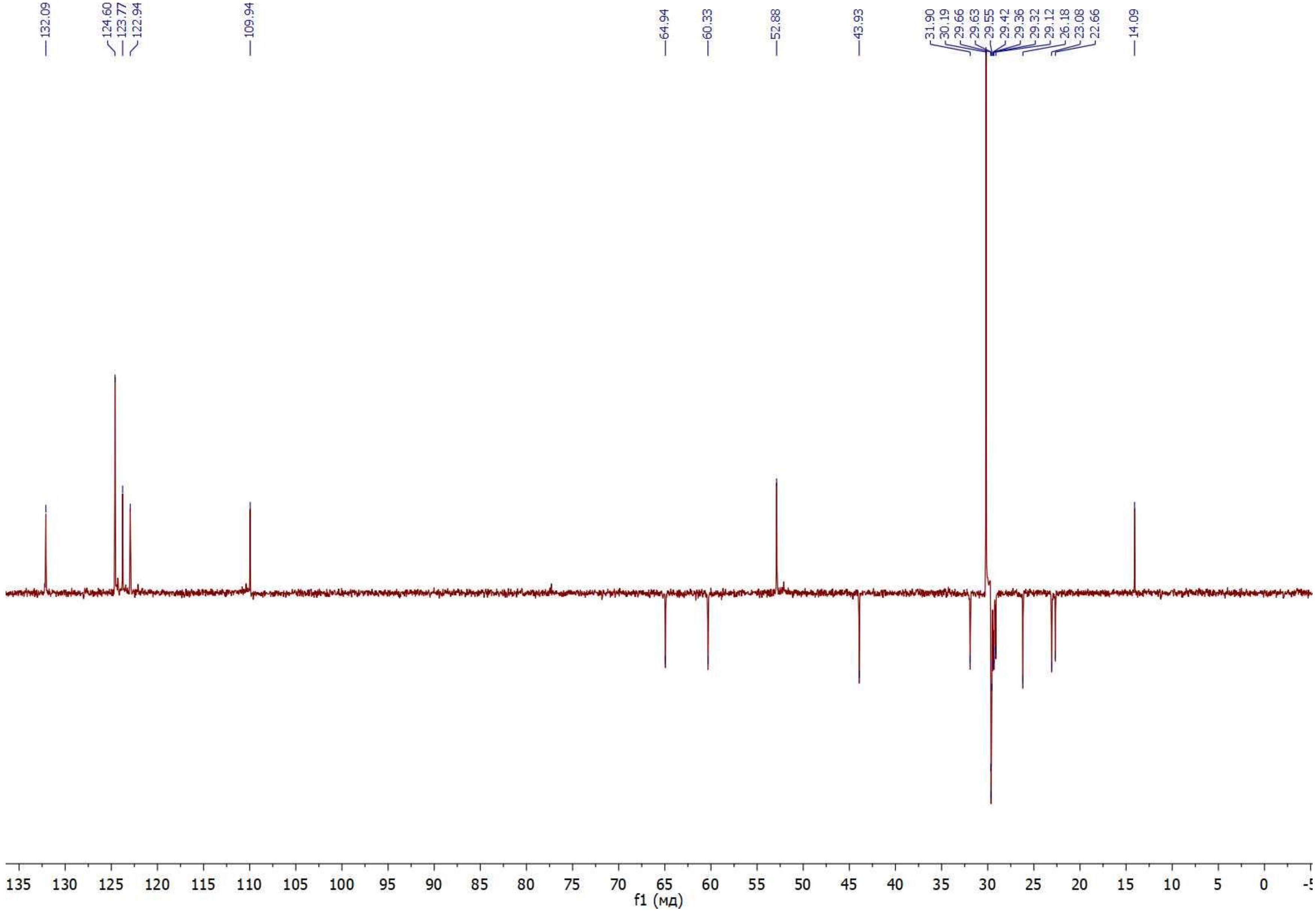

Figure S42. ^{13}C (dept) NMR spectrum of compound **3c** (101 MHz, $CDCl_3$)

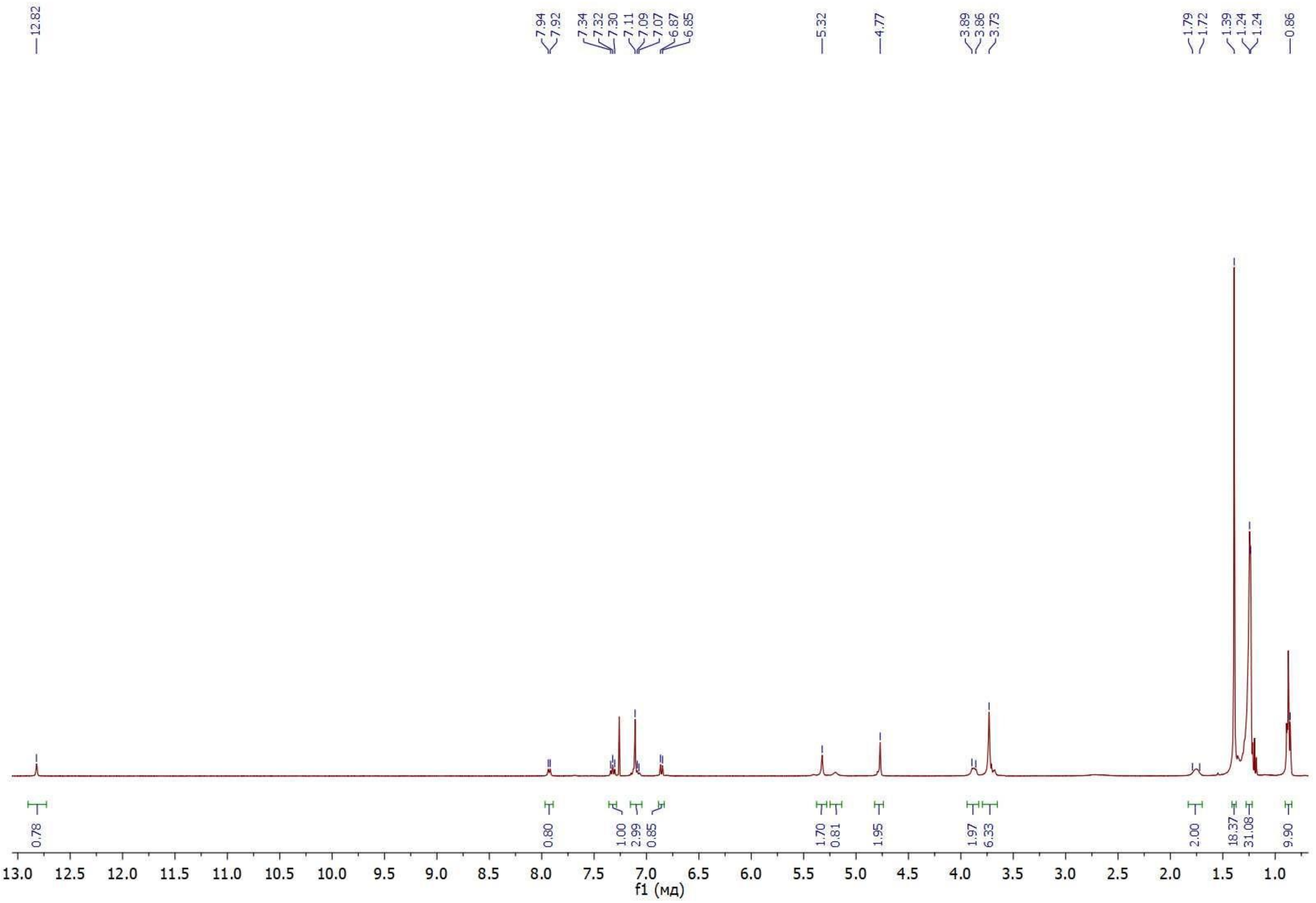

Figure S43. ^{1}H NMR spectrum of compound **3d** (400 MHz, $CDCl_3$)

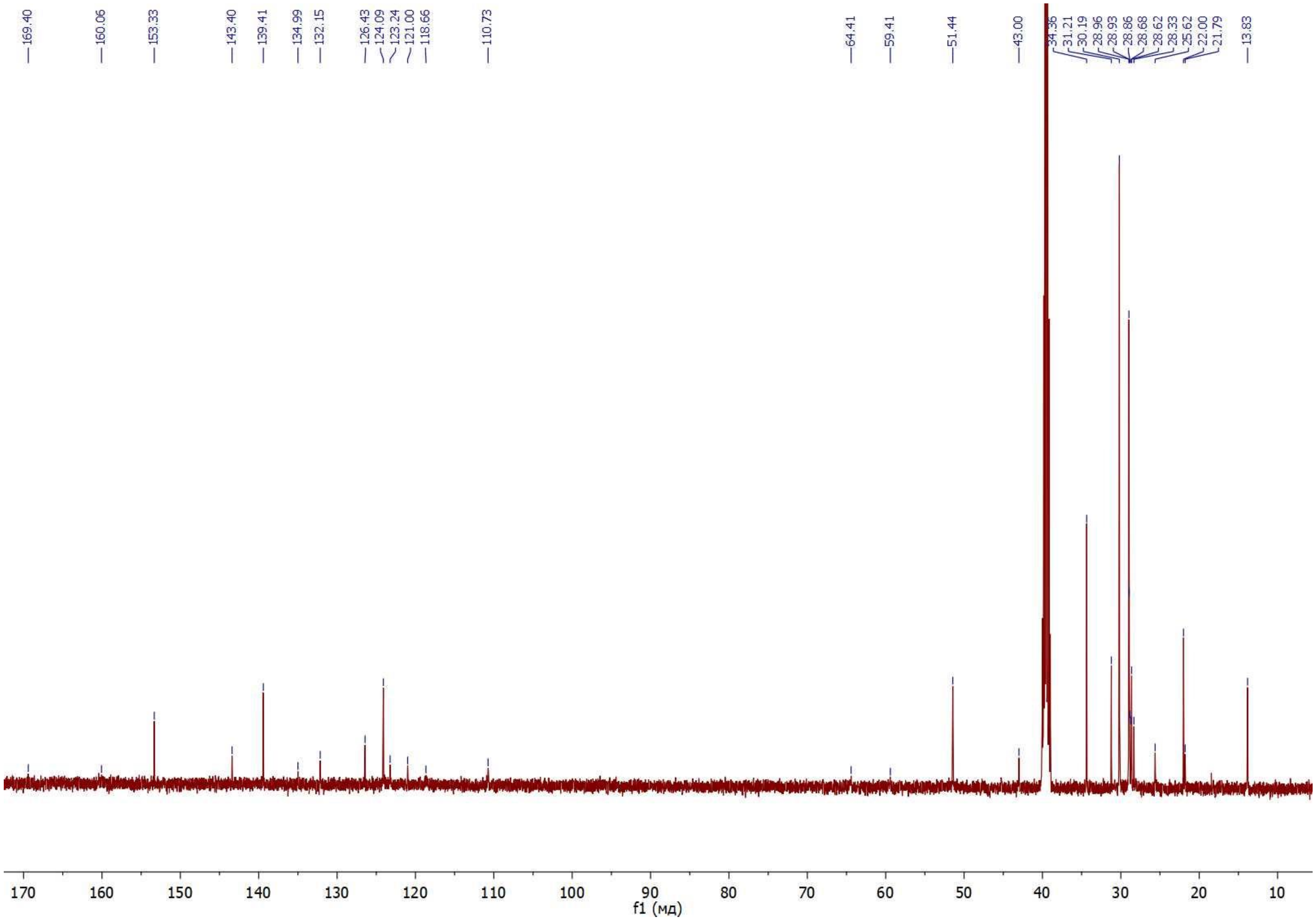

Figure S44. ^{13}C-{^{1}H} NMR spectrum of compound **3d** (101 MHz, DMSO-d_6)

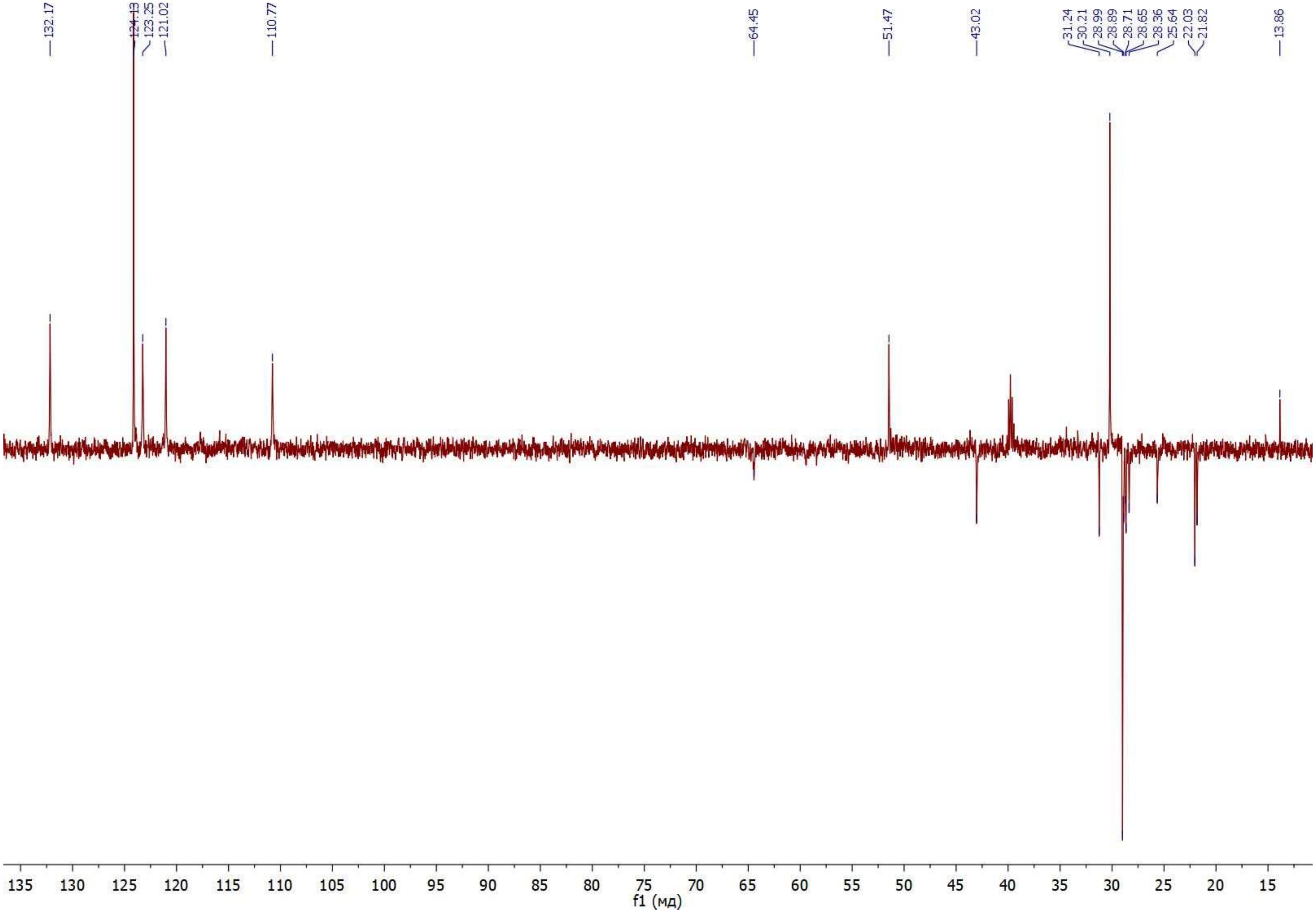

Figure S45. ^{13}C (dept) NMR spectrum of compound **3d** (101 MHz, DMSO-d_6)

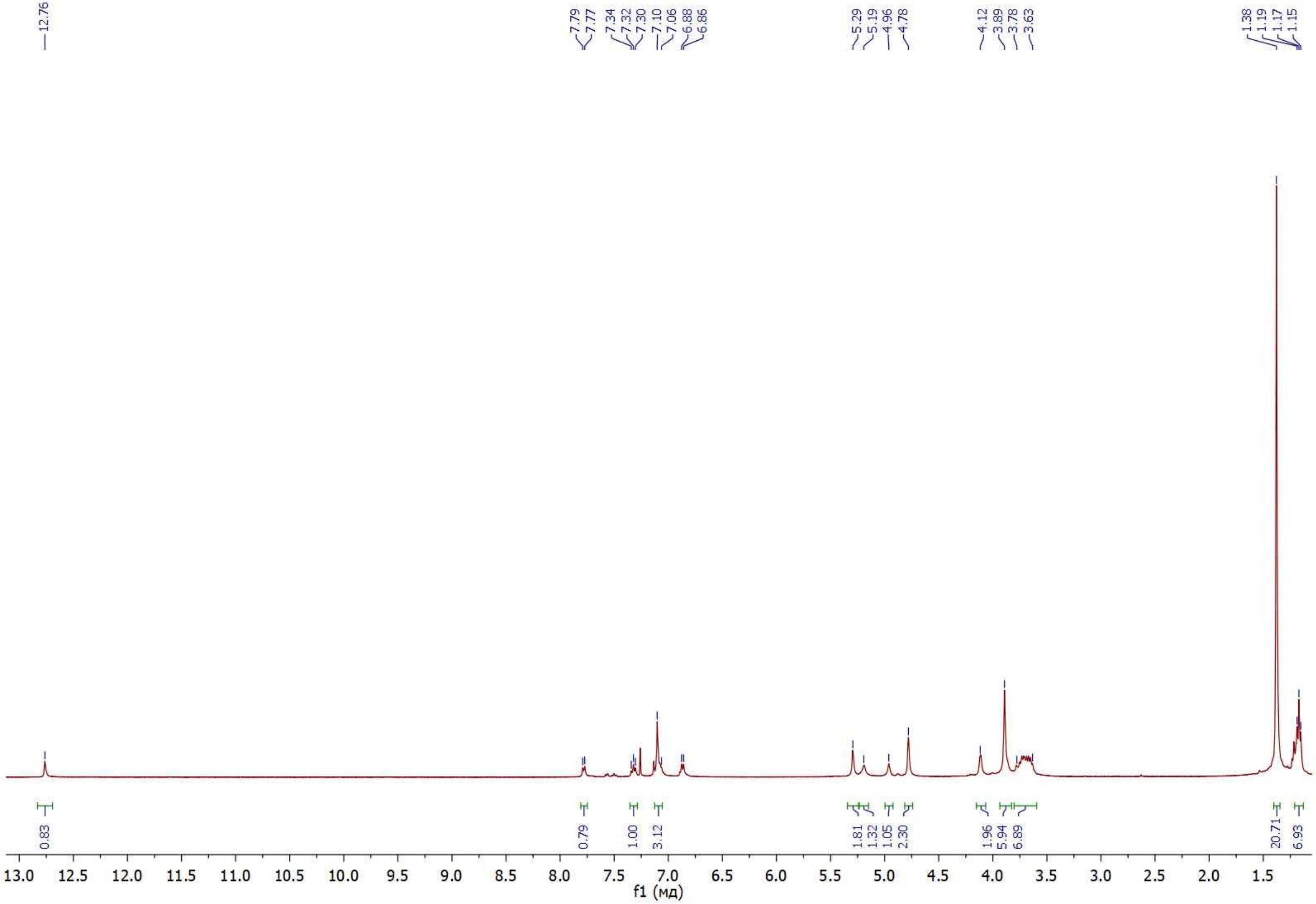

Figure S46. ^{1}H NMR spectrum of compound **3e** (400 MHz, $CDCl_3$)

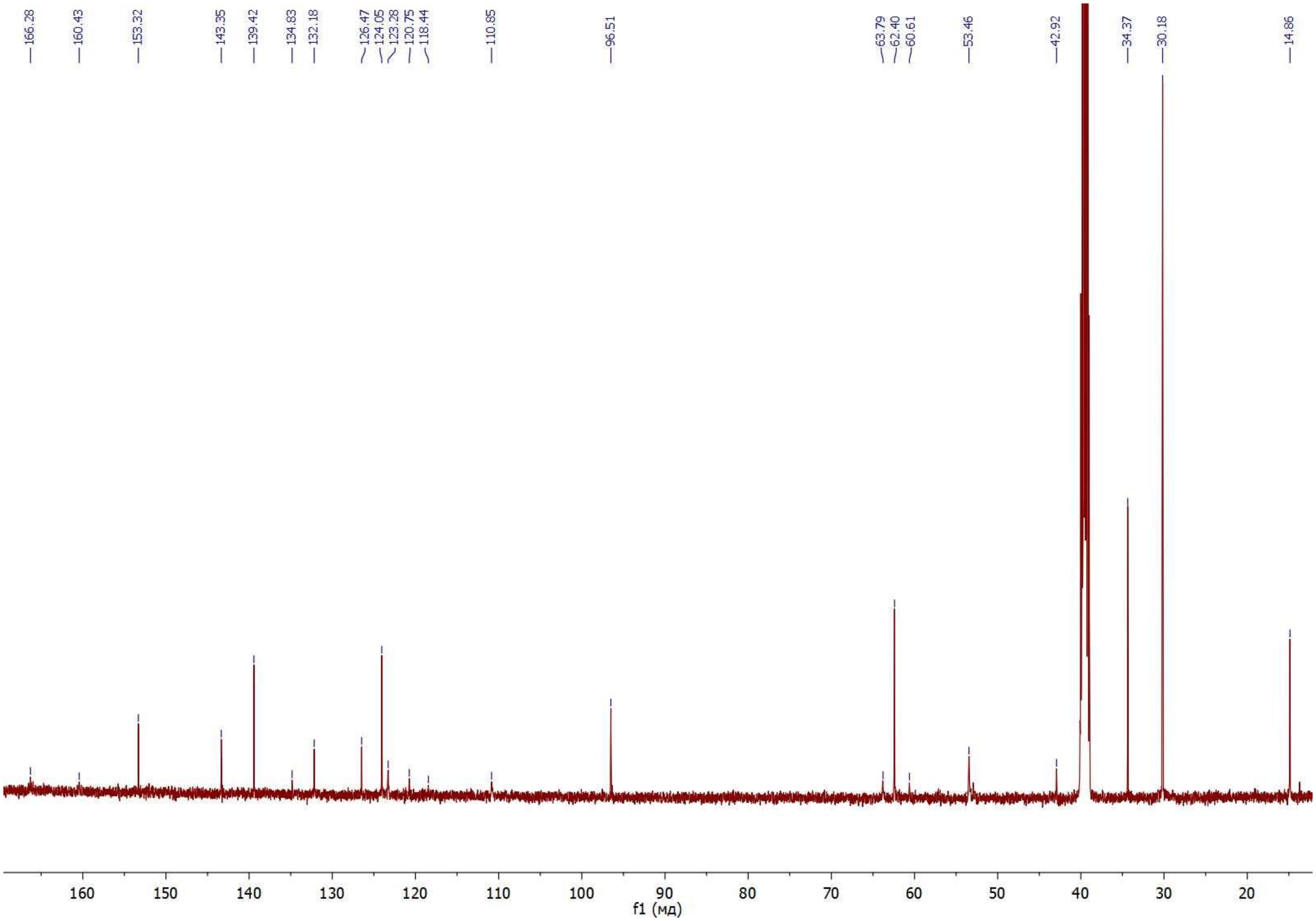

Figure S47. ^{13}C-{^{1}H} NMR spectrum of compound **3e** (101 MHz, DMSO-d_6)

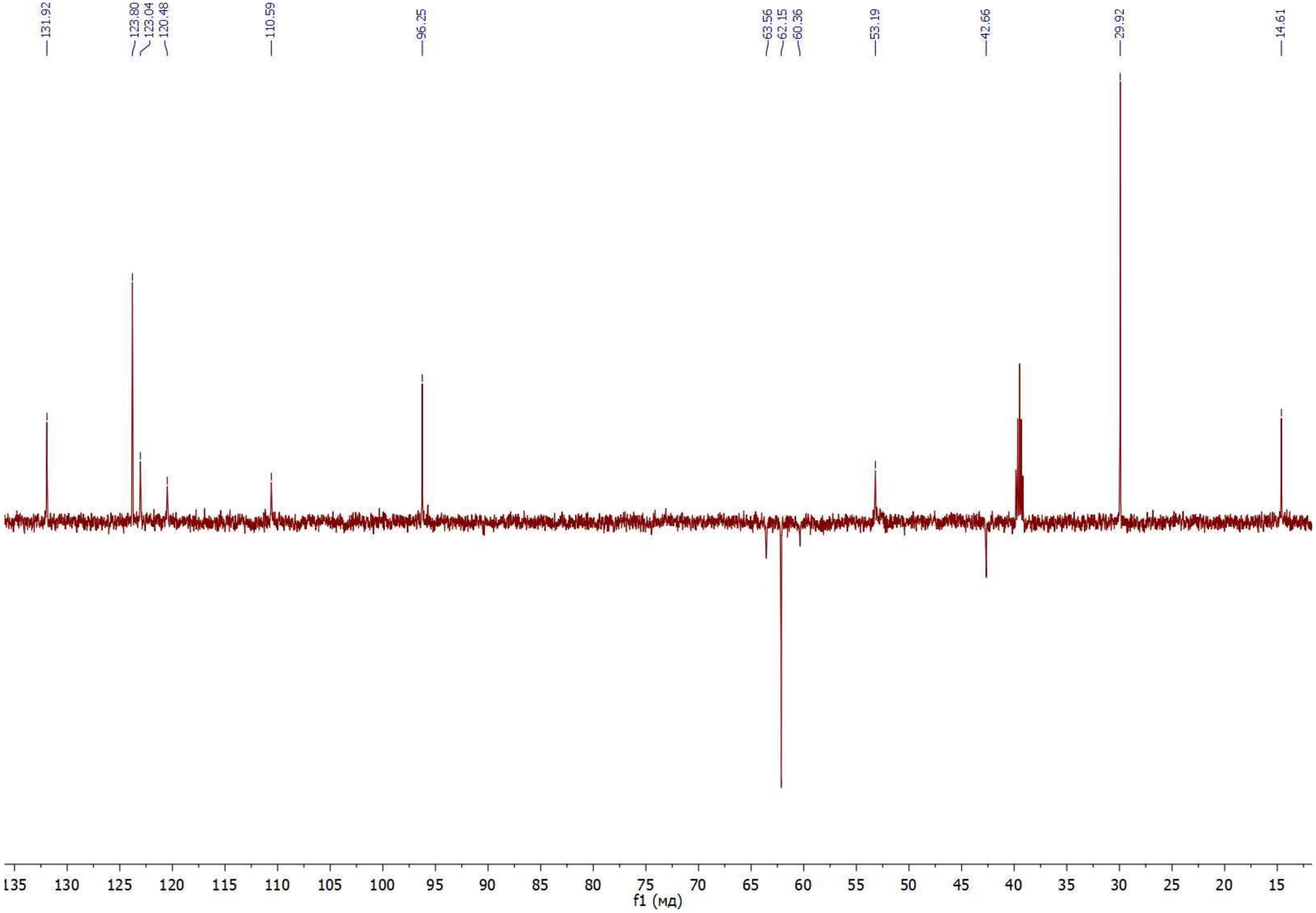

Figure S48. ^{13}C (dept) NMR spectrum of compound **3e** (101 MHz, DMSO-d_6)

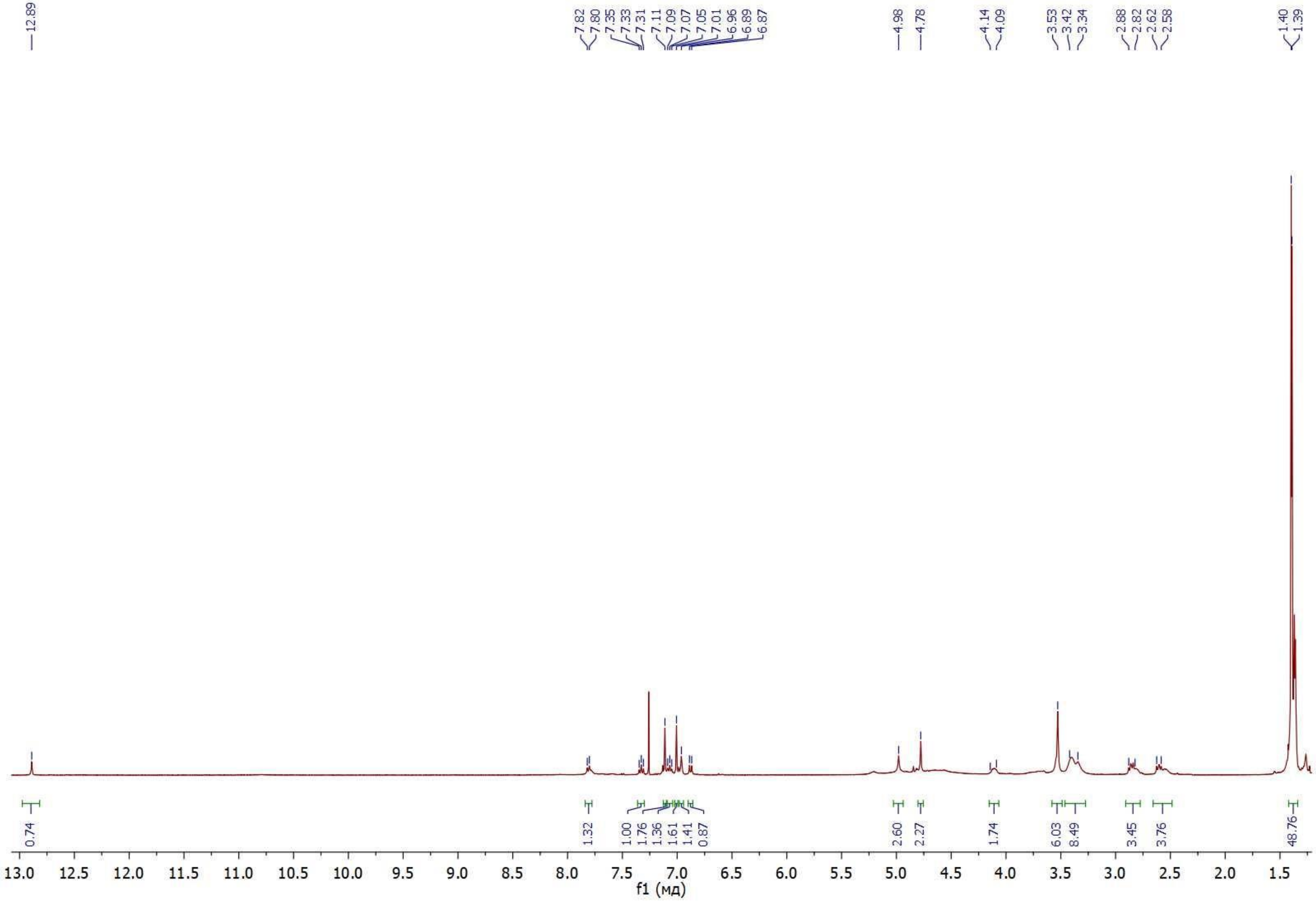

Figure S49. ^{1}H NMR spectrum of compound **3f** (400 MHz, $CDCl_3$)

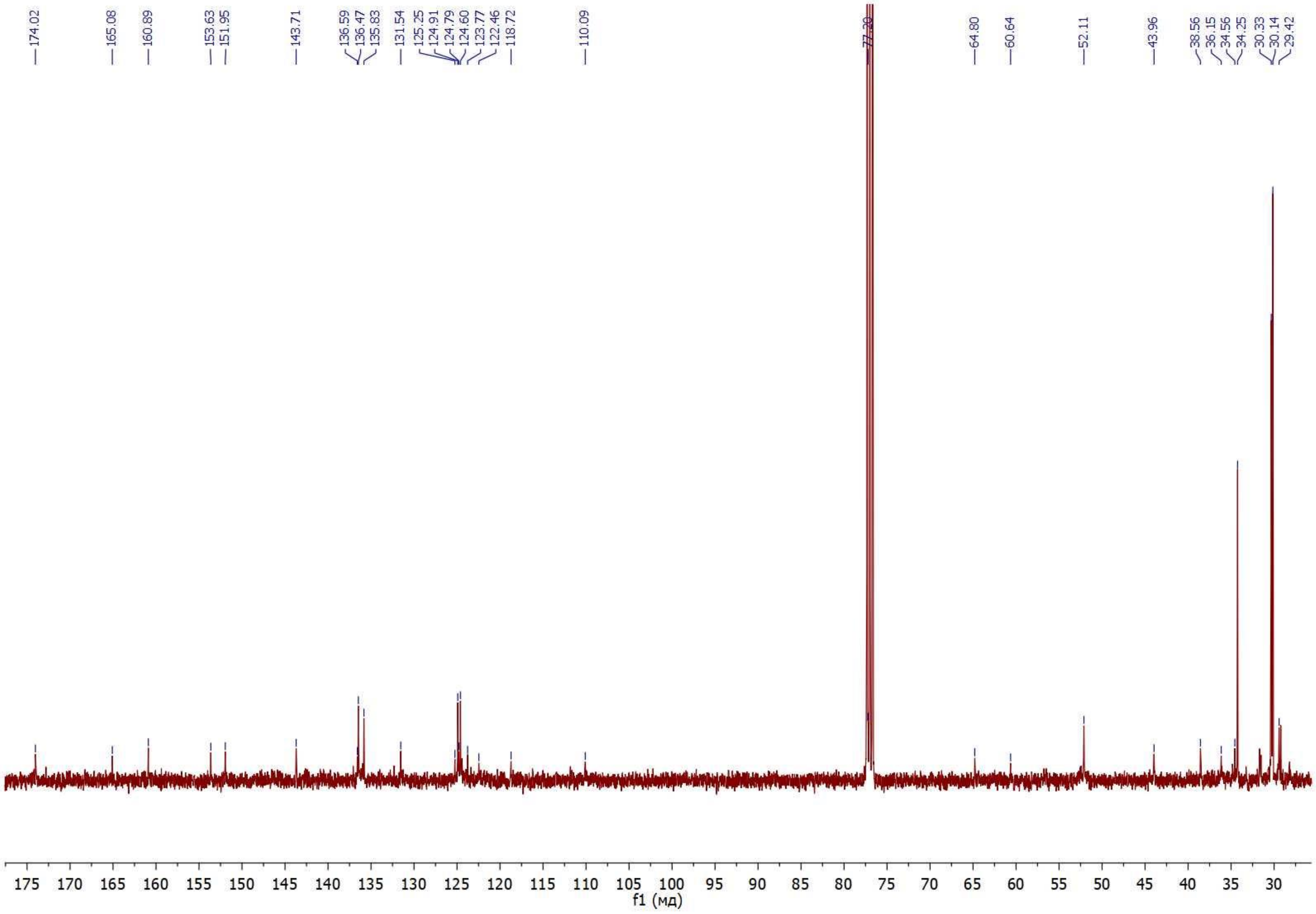

Figure S50. ^{13}C-{^{1}H} NMR spectrum of compound **3f** (101 MHz, $CDCl_3$)

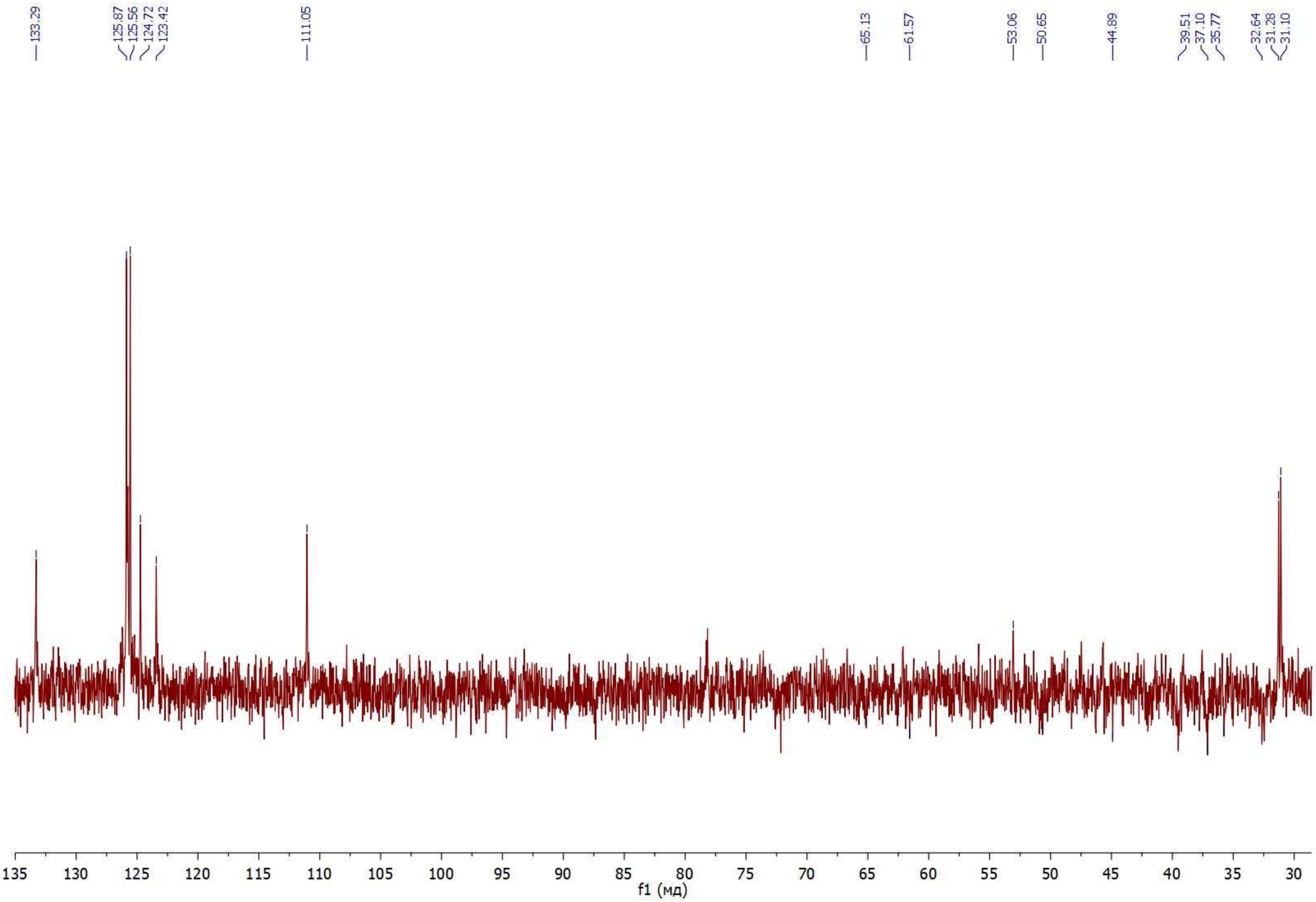

Figure S51. ^{13}C (dept) NMR spectrum of compound **3f** (101 MHz, $CDCl_3$)

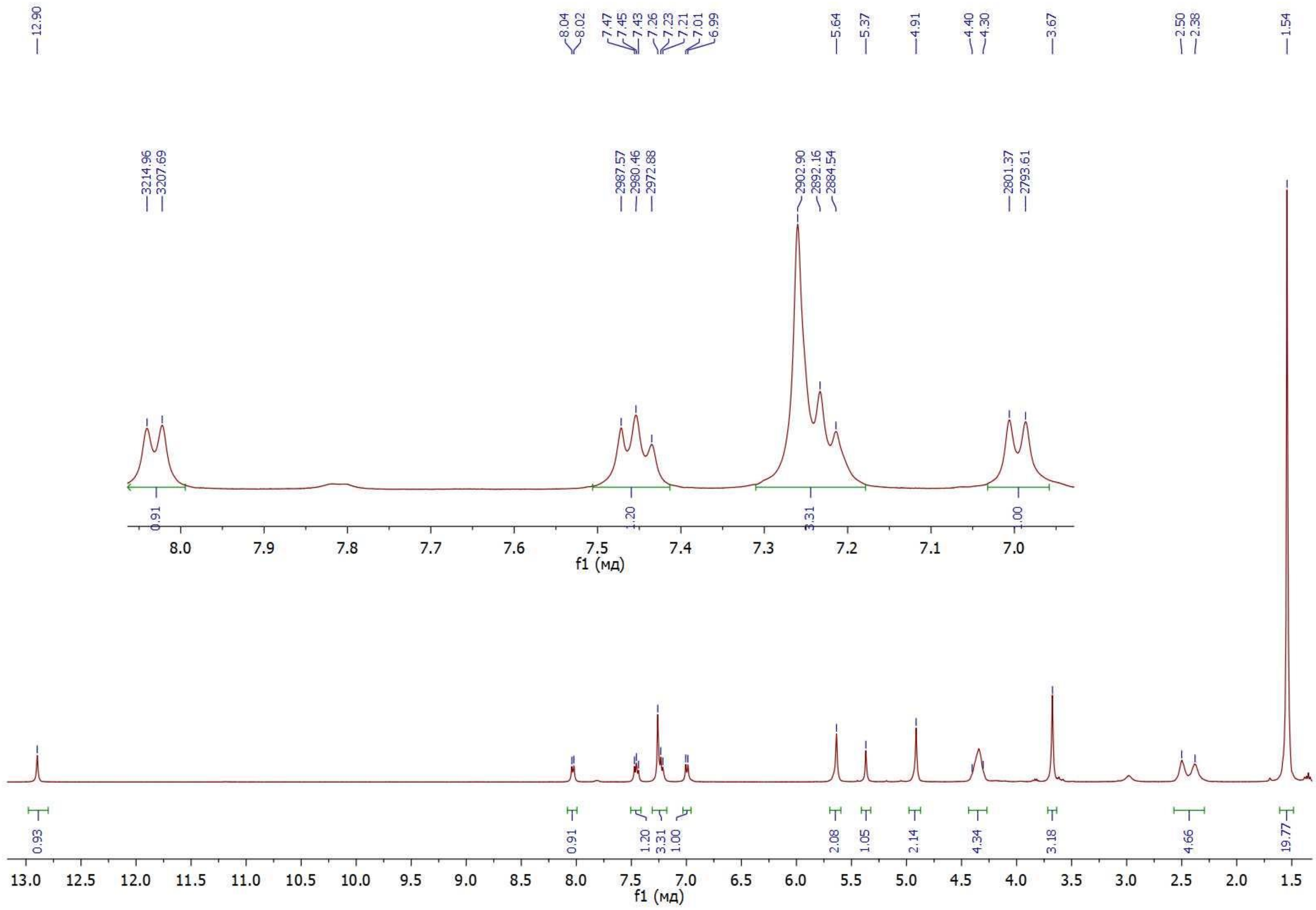

Figure S52. ^{1}H NMR spectrum of compound **3g** (400 MHz, $CDCl_3$)

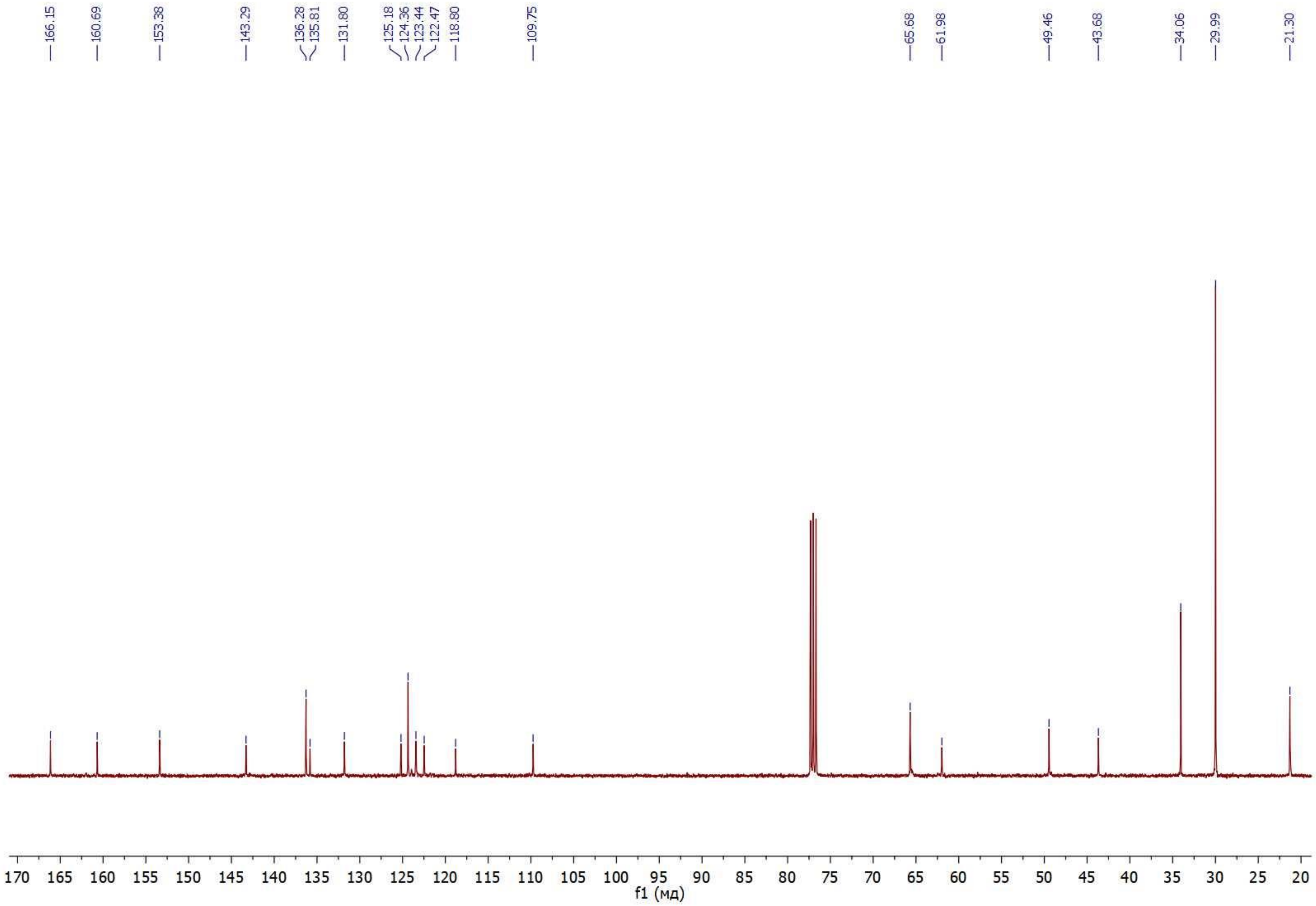

Figure S53. ^{13}C-{^{1}H} NMR spectrum of compound **3g** (101 MHz, $CDCl_3$)

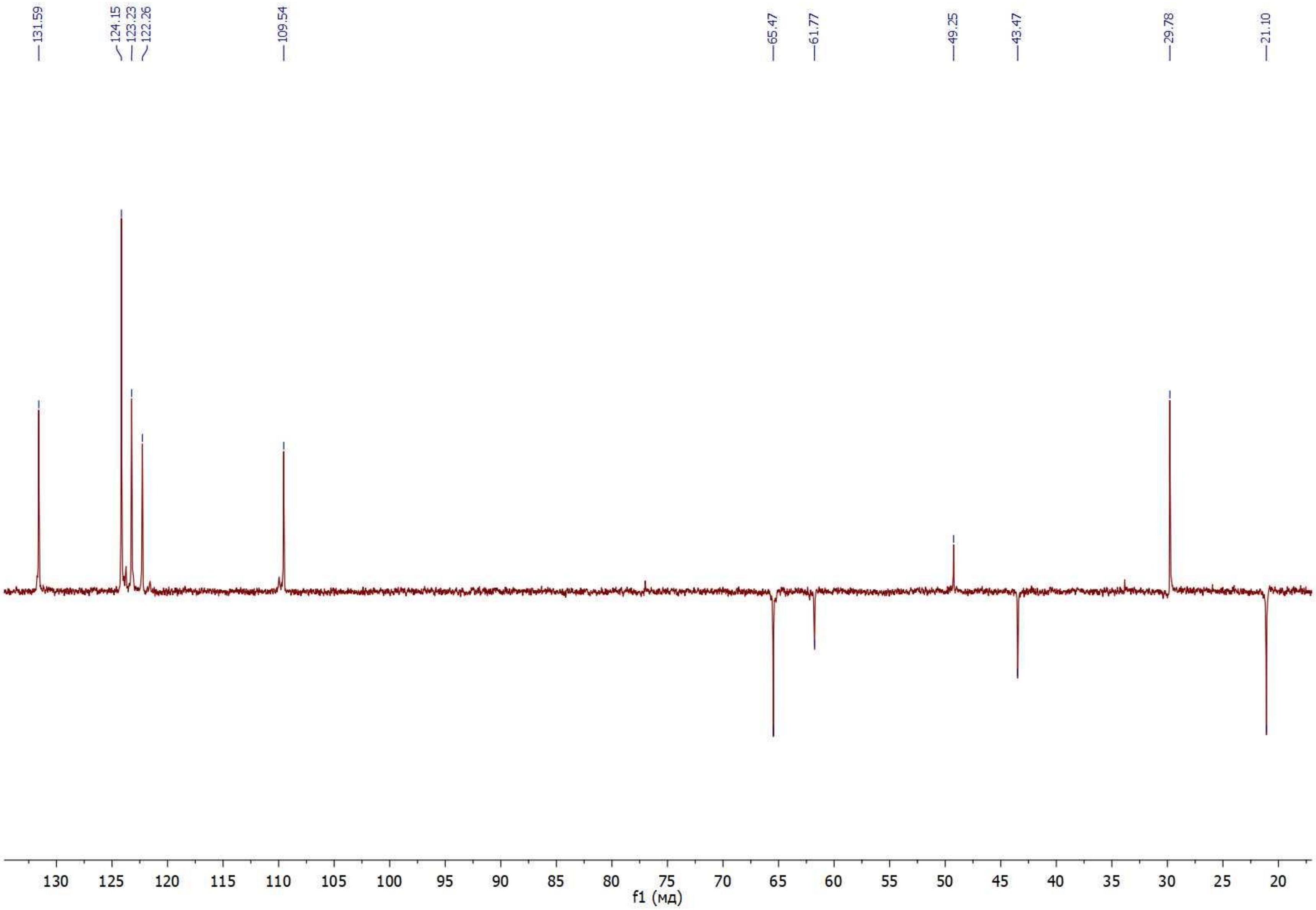

Figure S54. ^{13}C (dept) NMR spectrum of compound **3g** (101 MHz, $CDCl_3$)

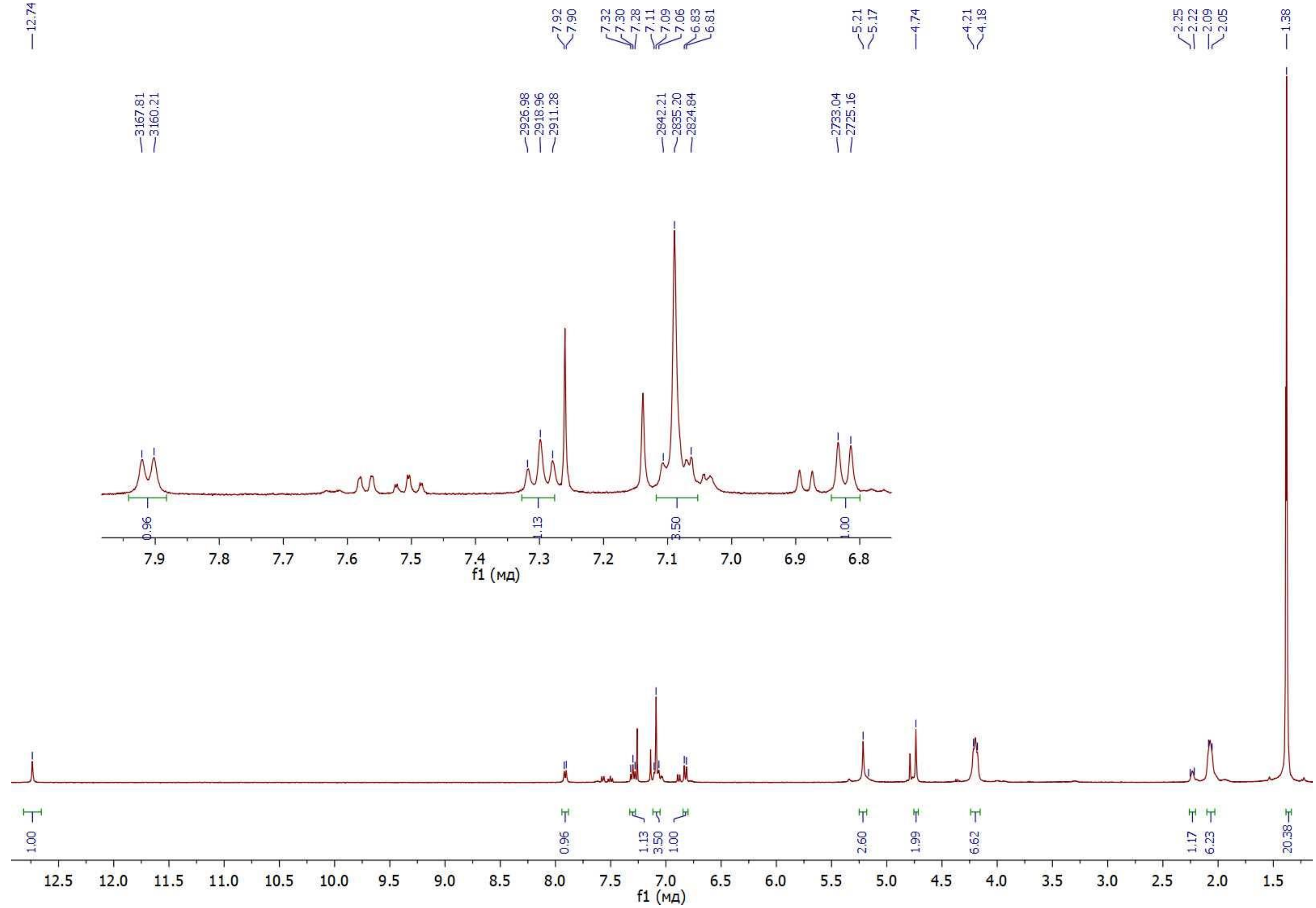

Figure S55. ^1H NMR spectrum of compound **3h** (400 MHz, $CDCl_3$)

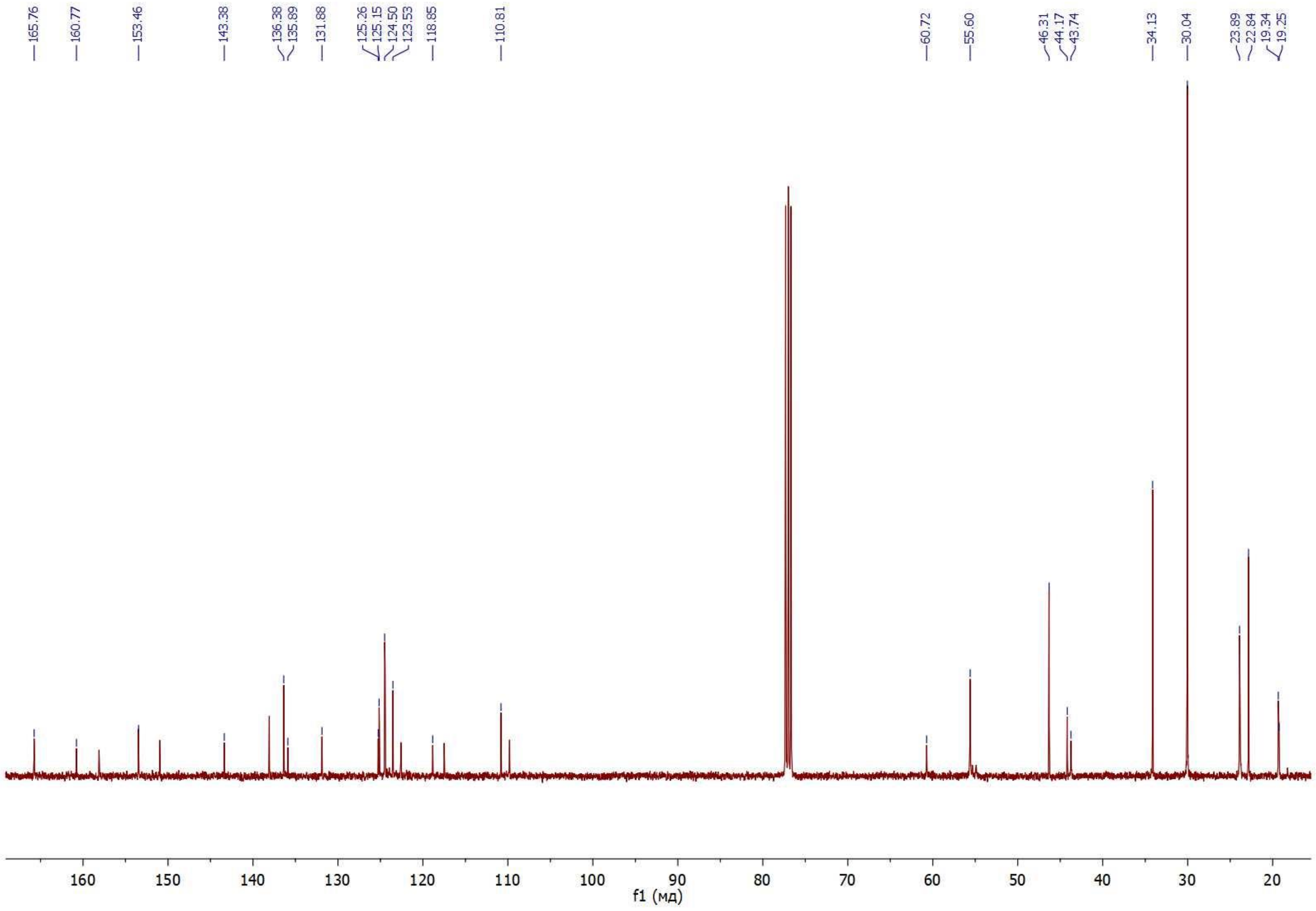

Figure S56. ^{13}C-{^{1}H} NMR spectrum of compound **3h** (101 MHz, $CDCl_3$)

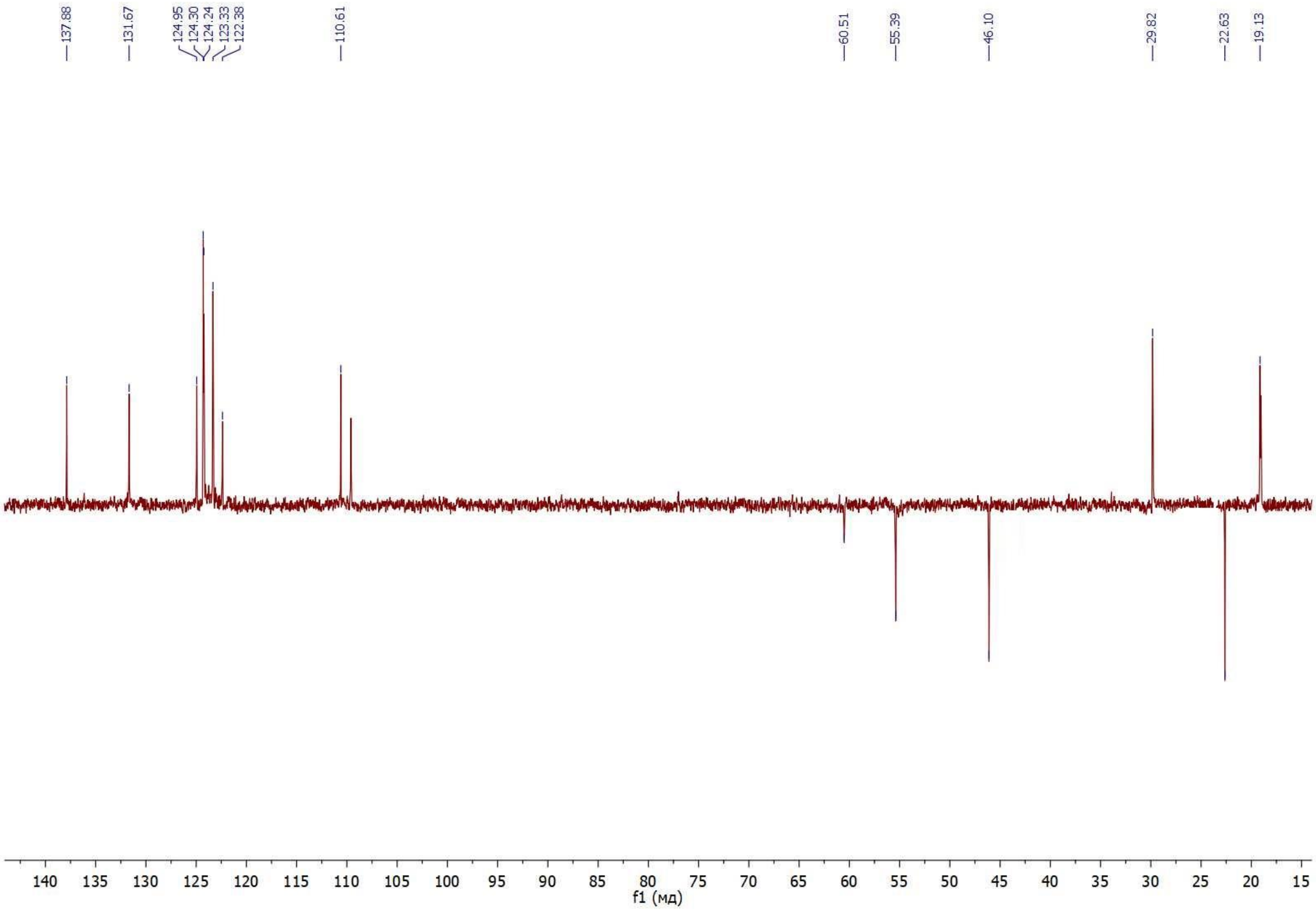

Figure S57. ^{13}C (dept) NMR spectrum of compound **3h** (101 MHz, $CDCl_3$)

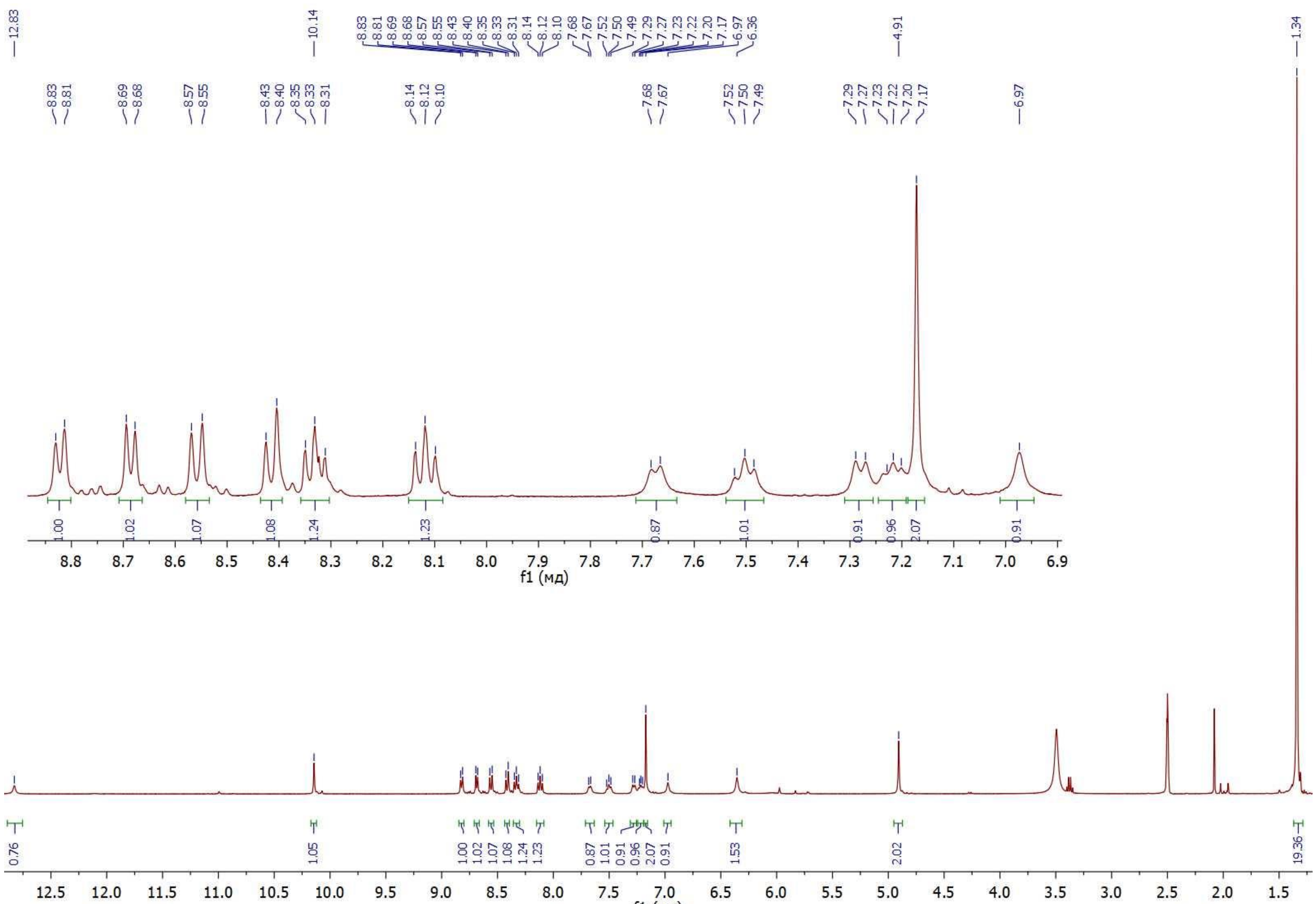

Figure S58. ^{1}H NMR spectrum of compound **3i** (400 MHz, DMSO-d_6)

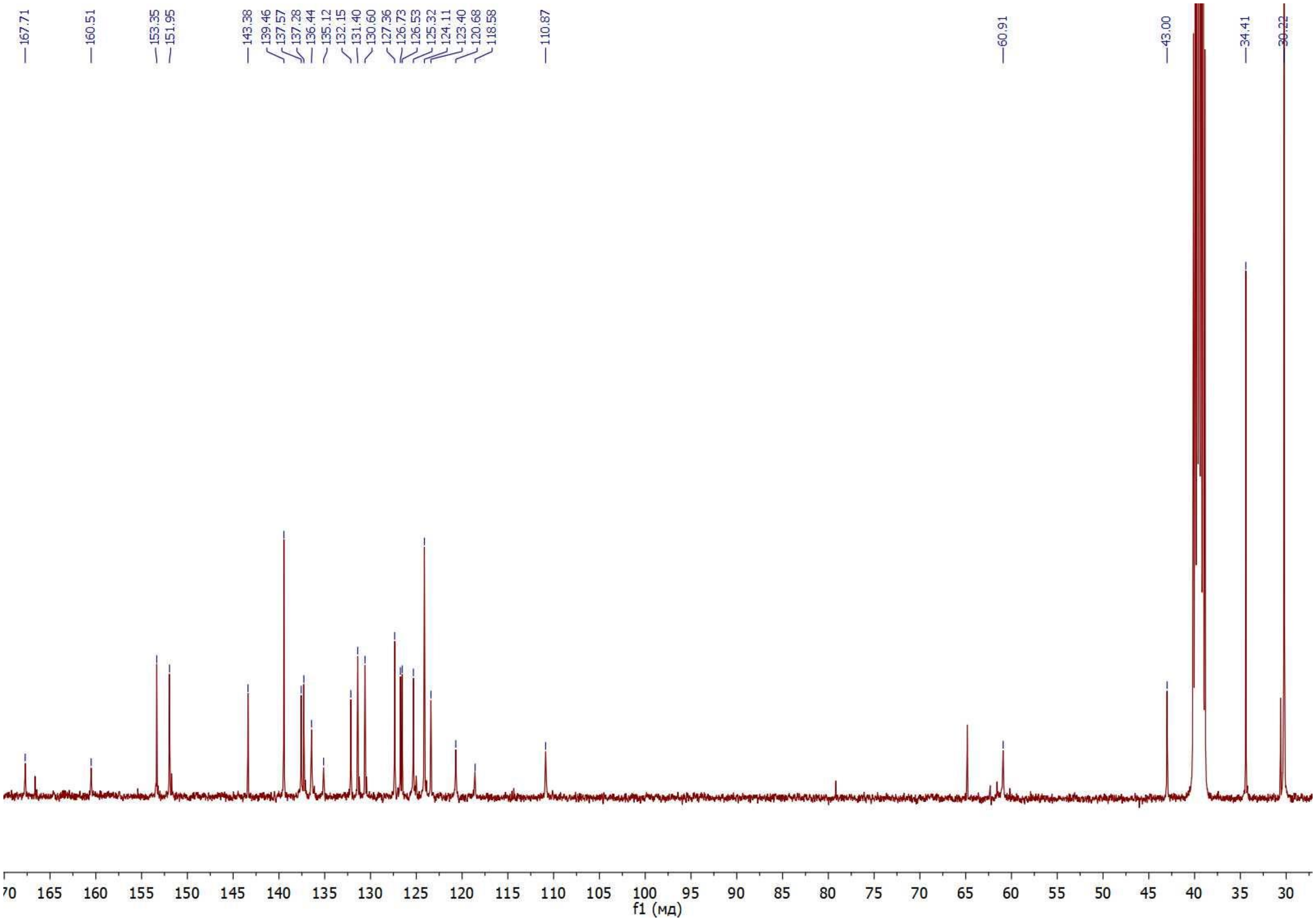

Figure S59. ^{13}C-{^{1}H} NMR spectrum of compound **3i** (101 MHz, DMSO-d_6)

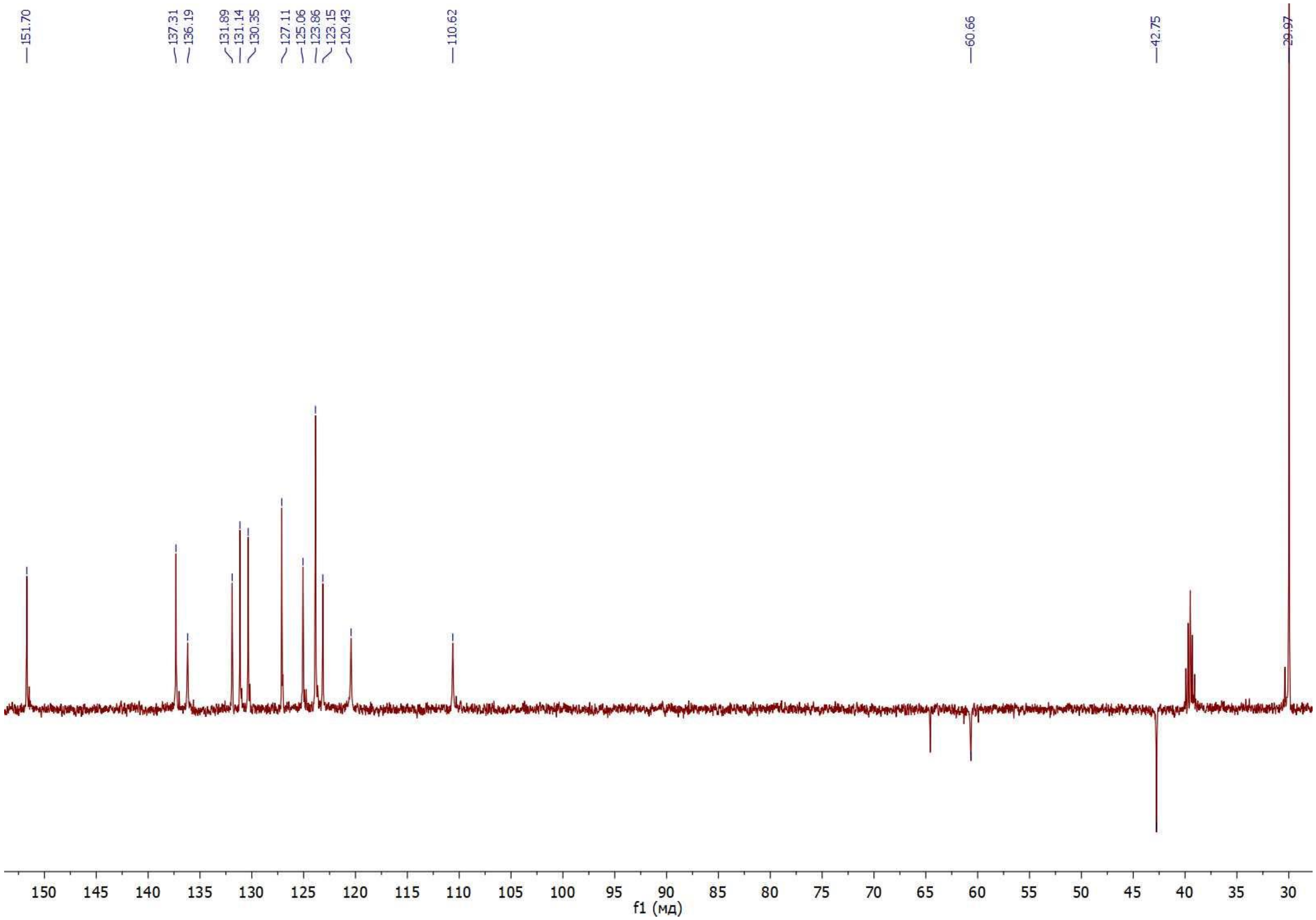

Figure S60. ^{13}C (dept) NMR spectrum of compound **3i** (101 MHz, DMSO-d_6)

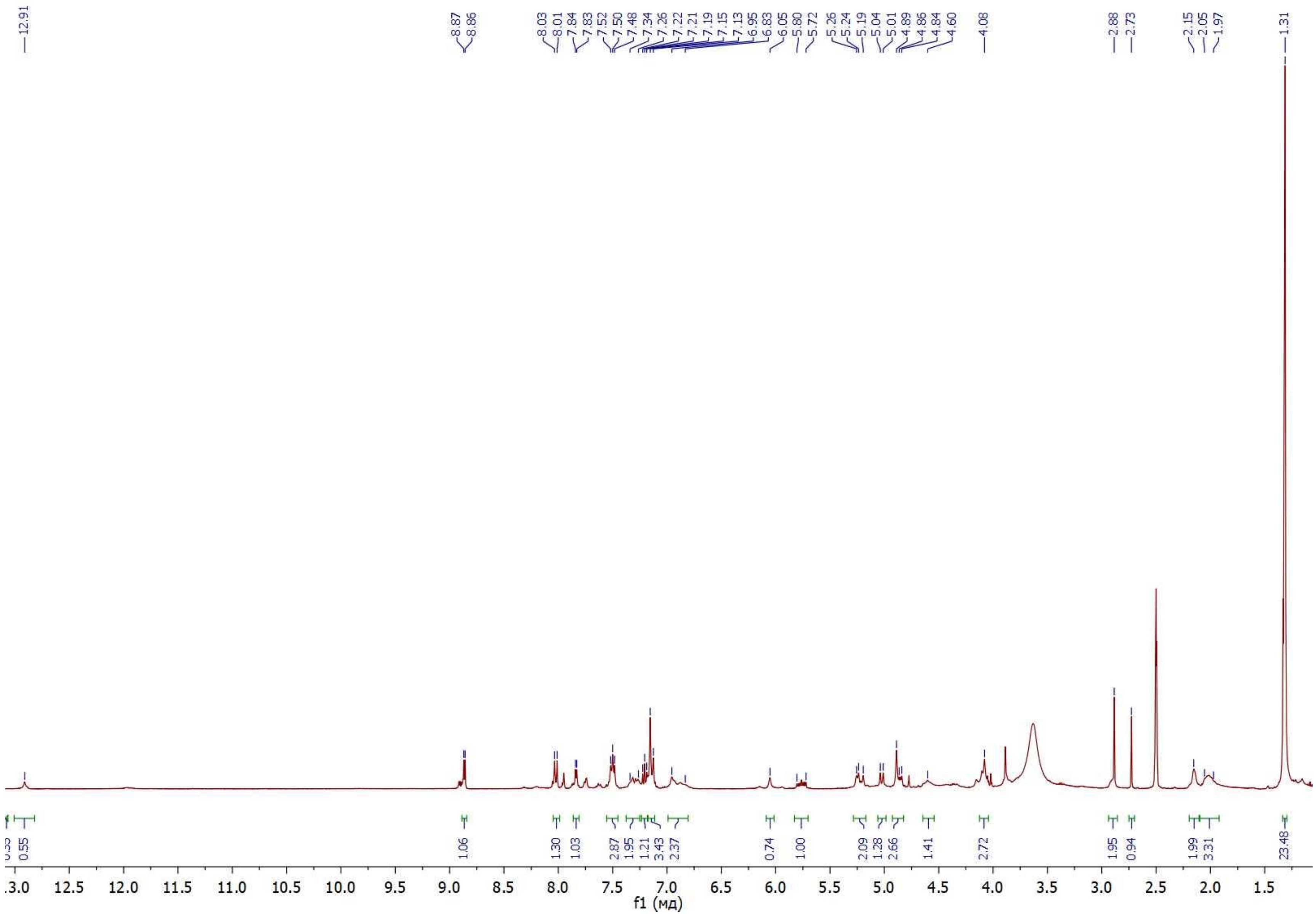

Figure S61. ^{1}H NMR spectrum of compound **3j** (400 MHz, DMSO-d_6)

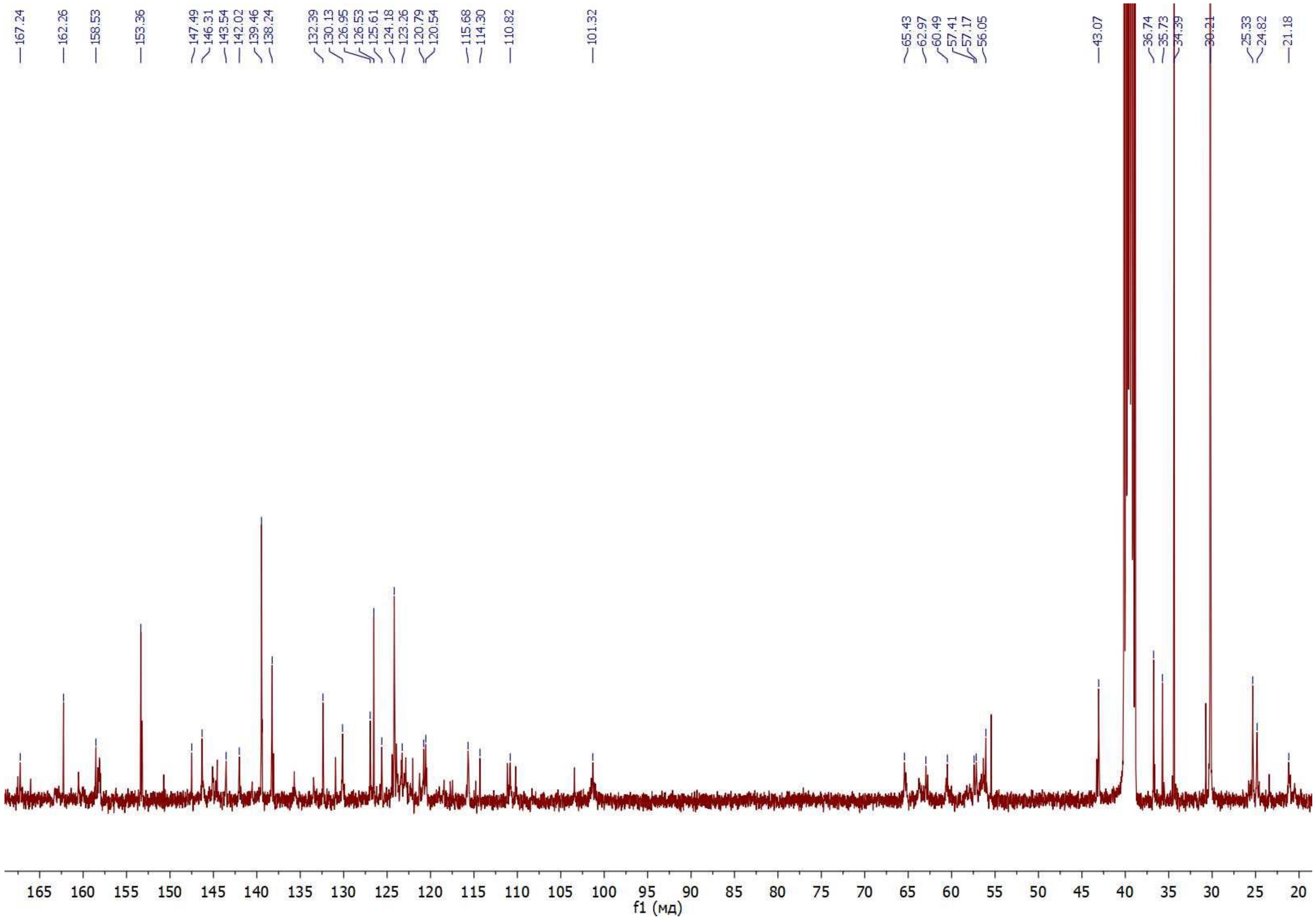

Figure S62. ^{13}C-{^{1}H} NMR spectrum of compound **3j** (101 MHz, DMSO-d_6)

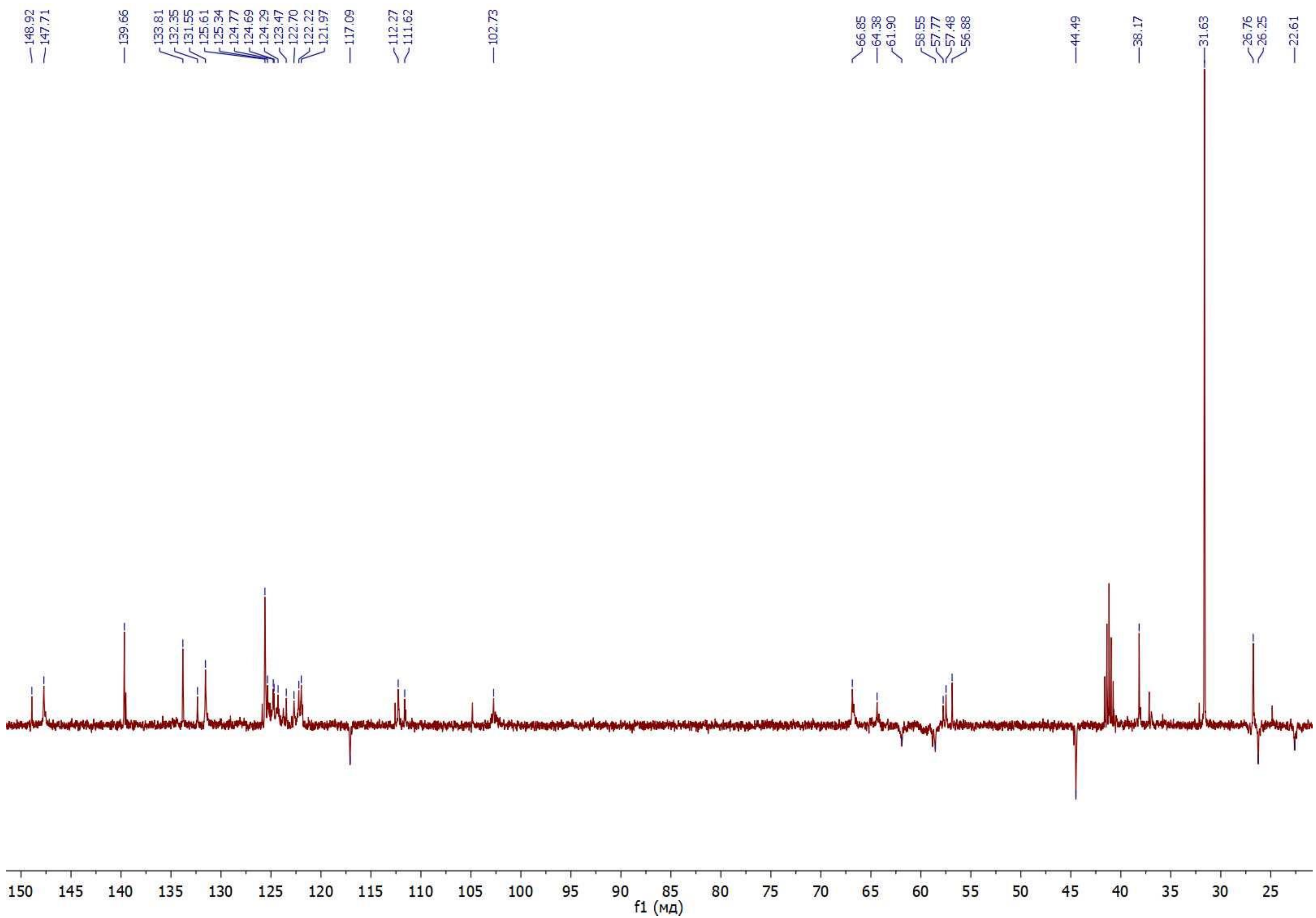

Figure S63. ^{13}C (dept) NMR spectrum of compound **3j** (101 MHz, DMSO-d_6)

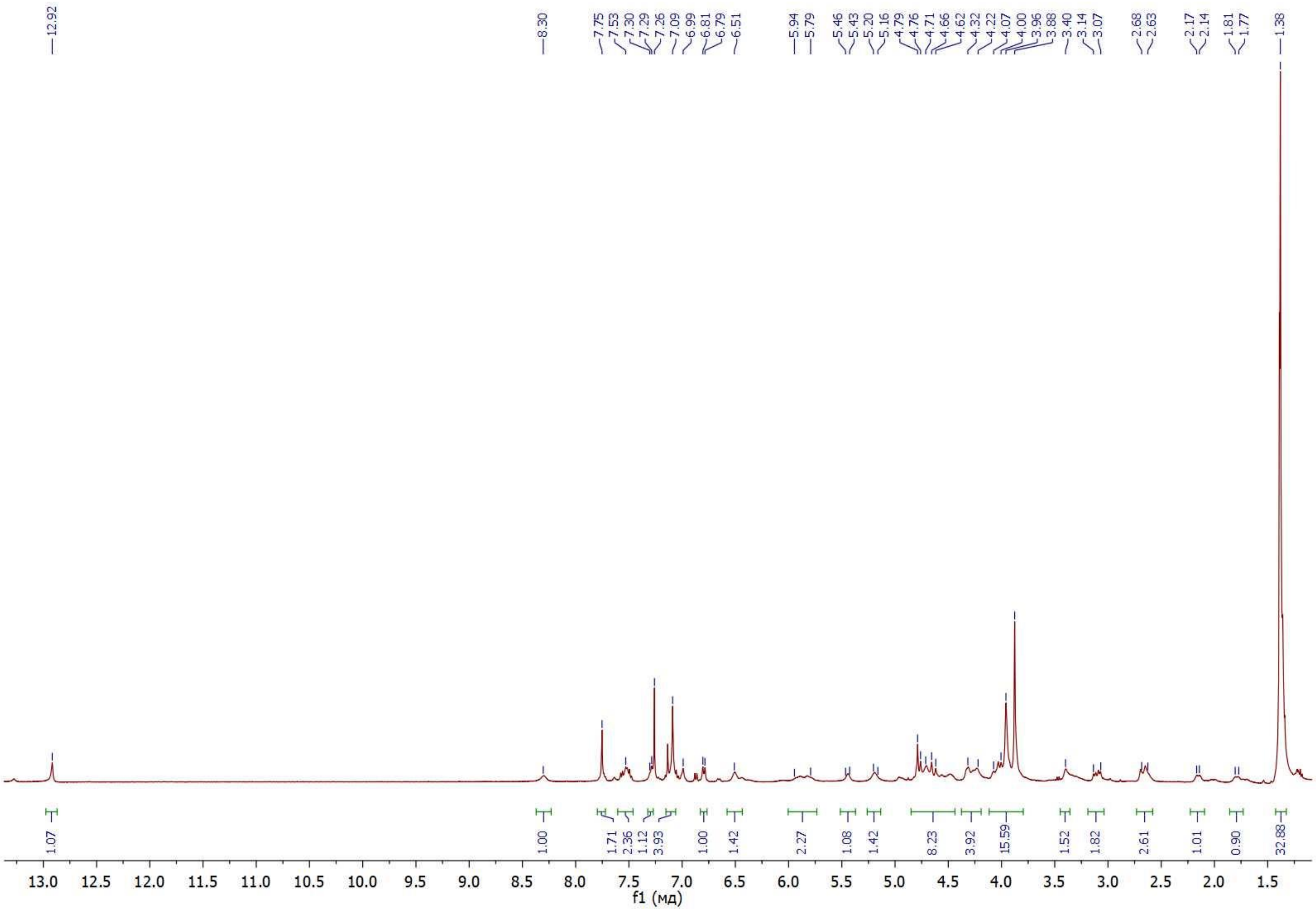

Figure S64. ^{1}H NMR spectrum of compound **3k** (400 MHz, $CDCl_3$)

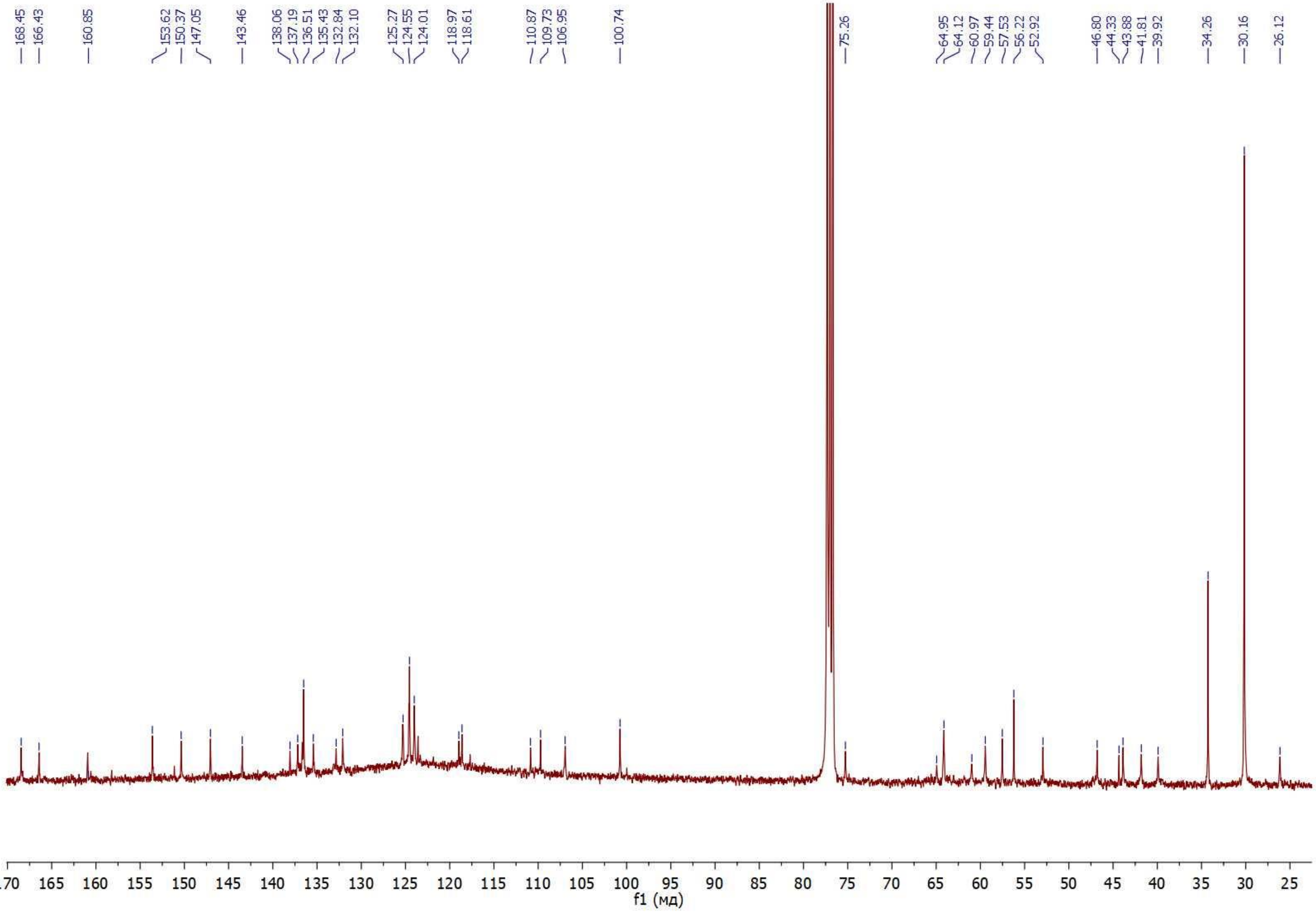

Figure S65. ^{13}C-{^{1}H} NMR spectrum of compound **3k** (101 MHz, $CDCl_3$)

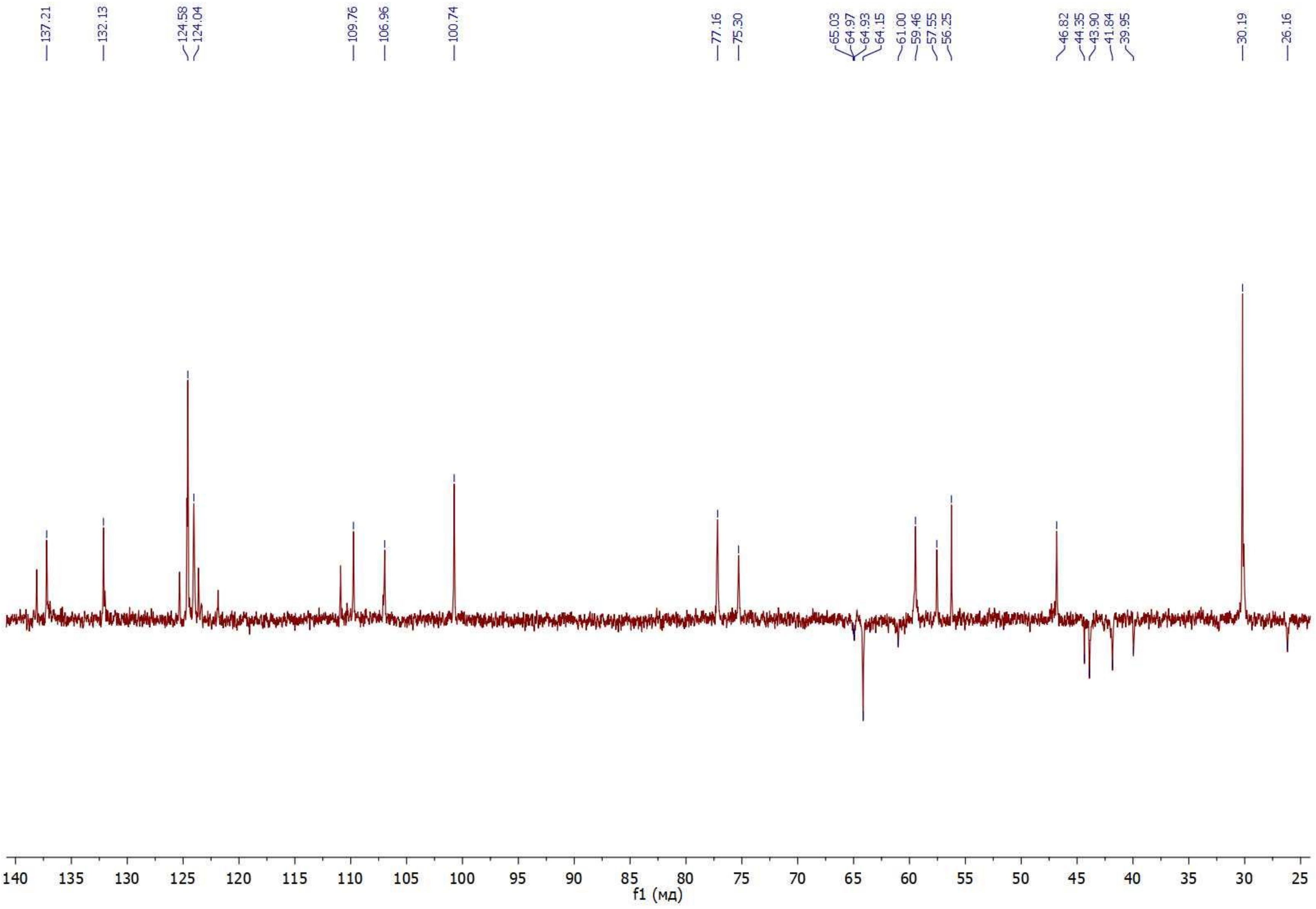

Figure S66. ^{13}C (dept) NMR spectrum of compound **3k** (101 MHz, $CDCl_3$)

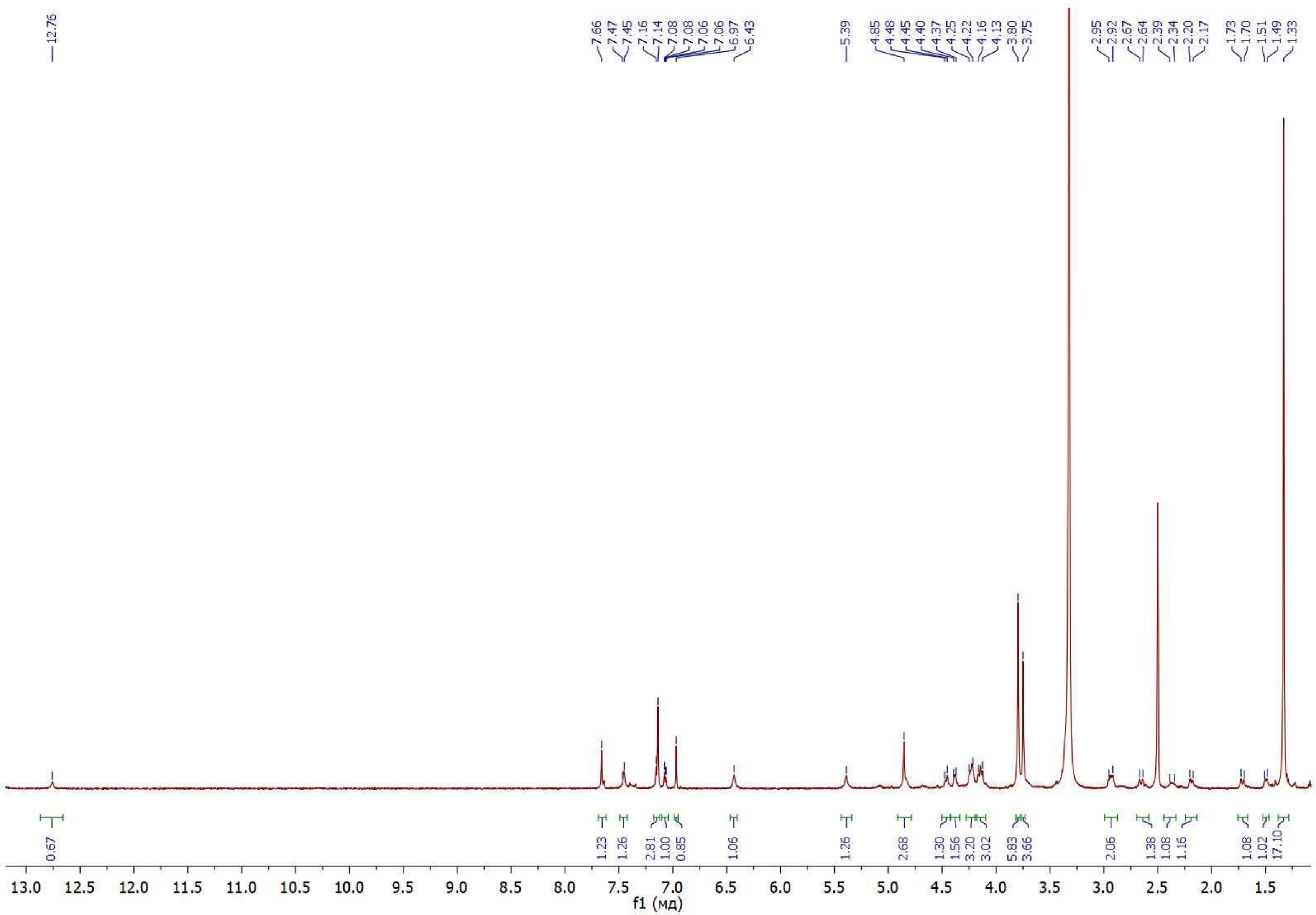

Figure S67. ^{1}H NMR spectrum of compound **3l** (600 MHz, DMSO-d_6)

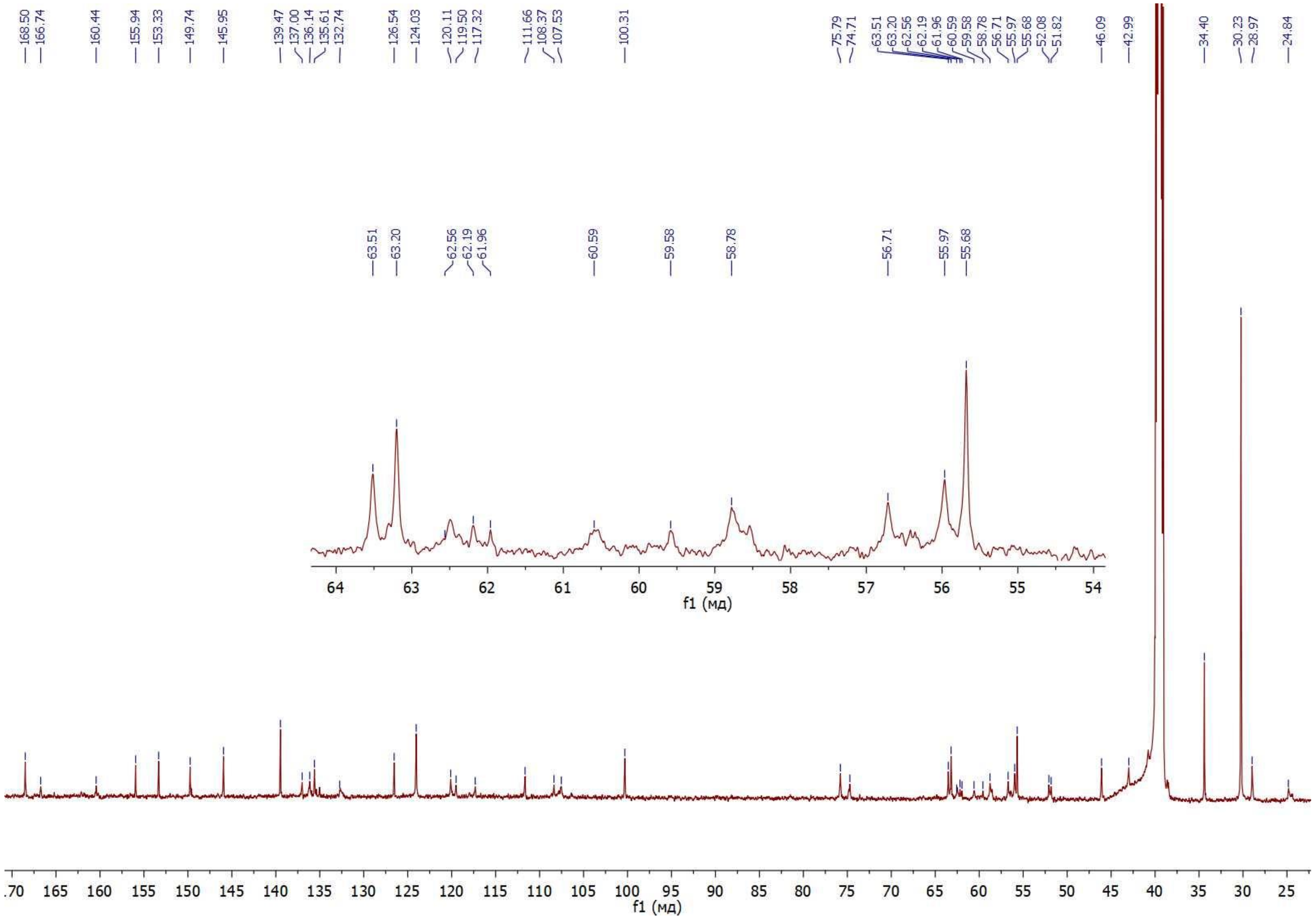

Figure S68. ^{13}C-{^{1}H} NMR spectrum of compound **3l** (151 MHz, DMSO-d_6)

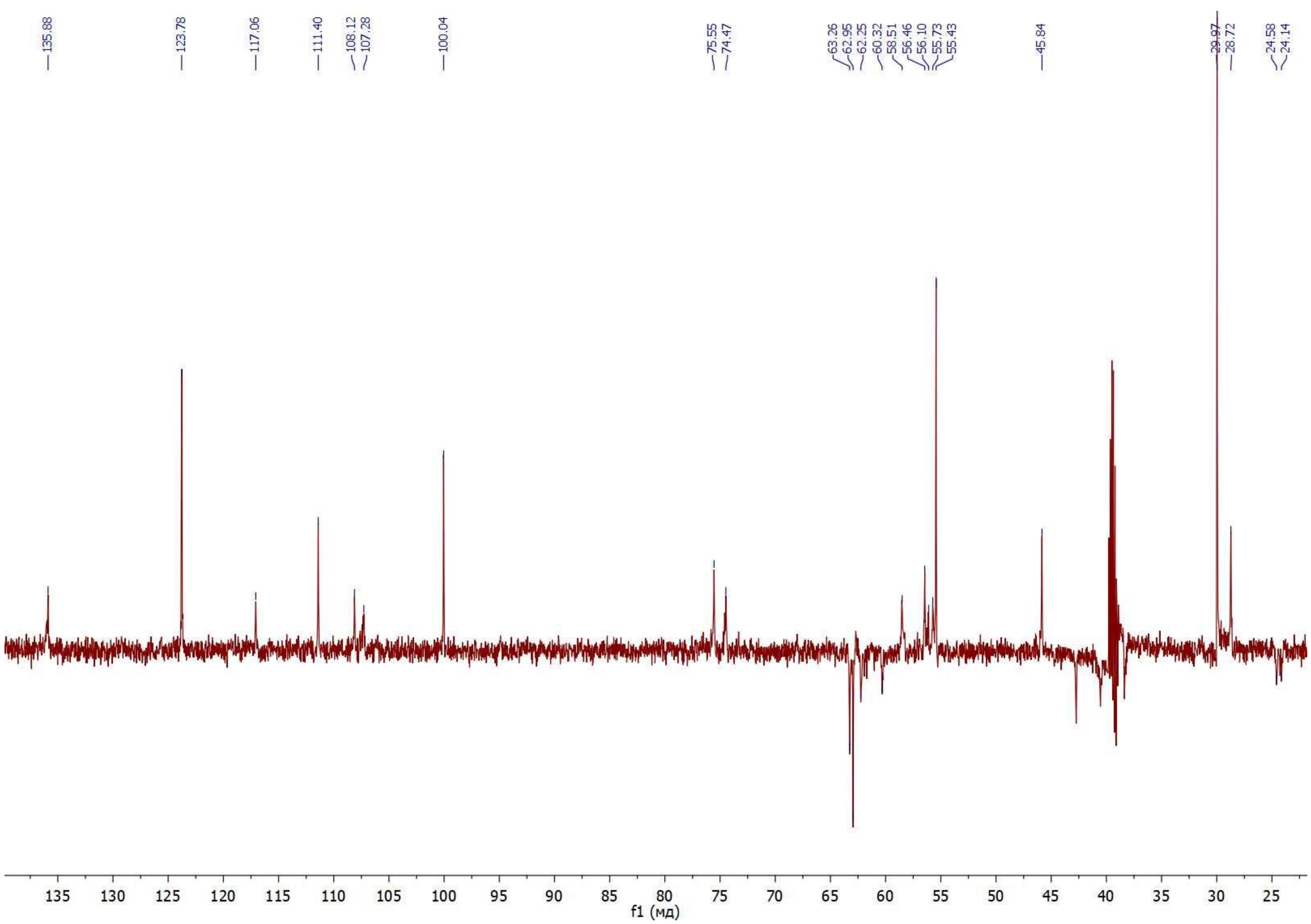

Figure S69. ^{13}C (dept) NMR spectrum of compound **3l** (151 MHz, DMSO-d_6)

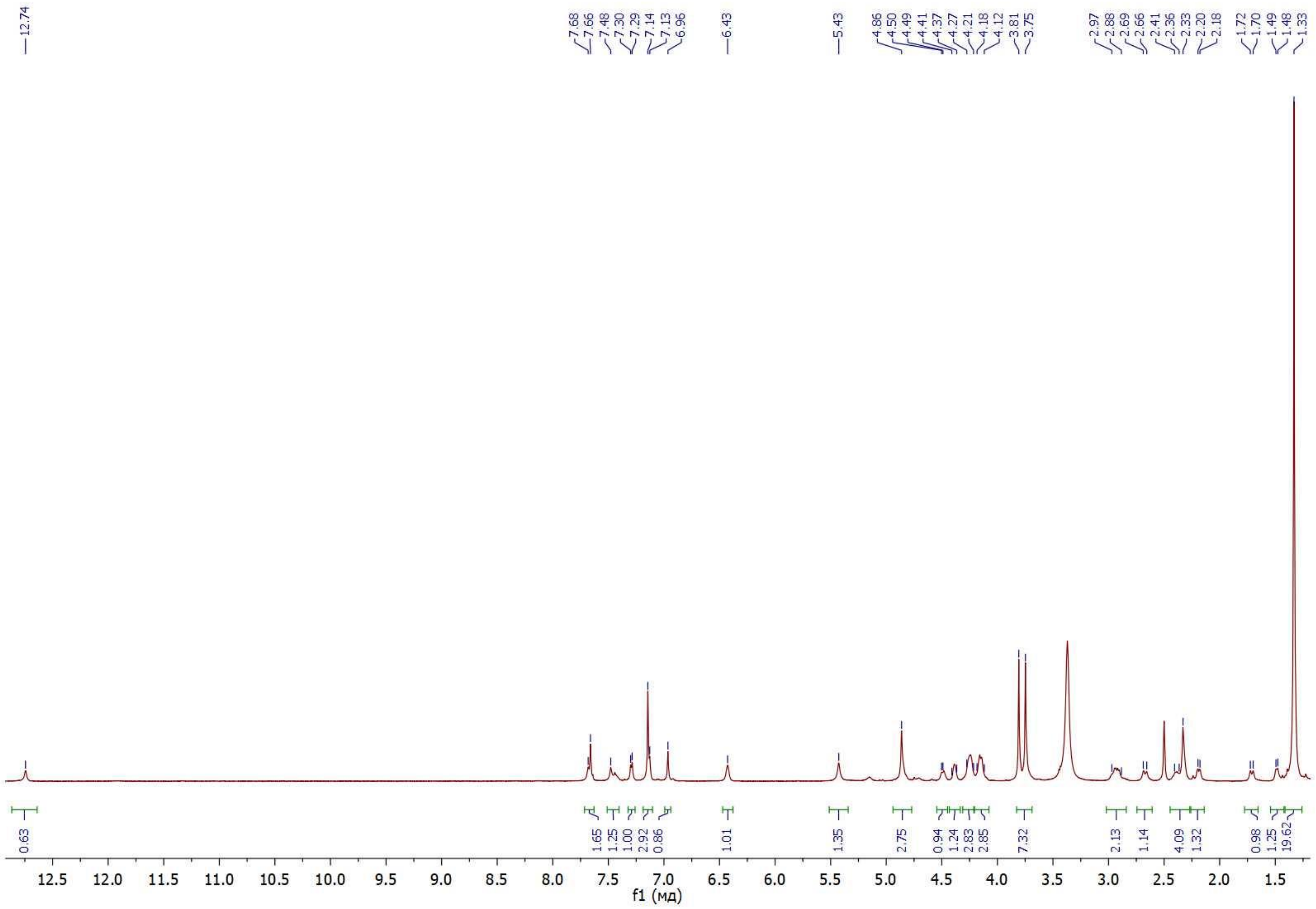

Figure S70. ^{1}H NMR spectrum of compound **3m** (600 MHz, DMSO-d_6)

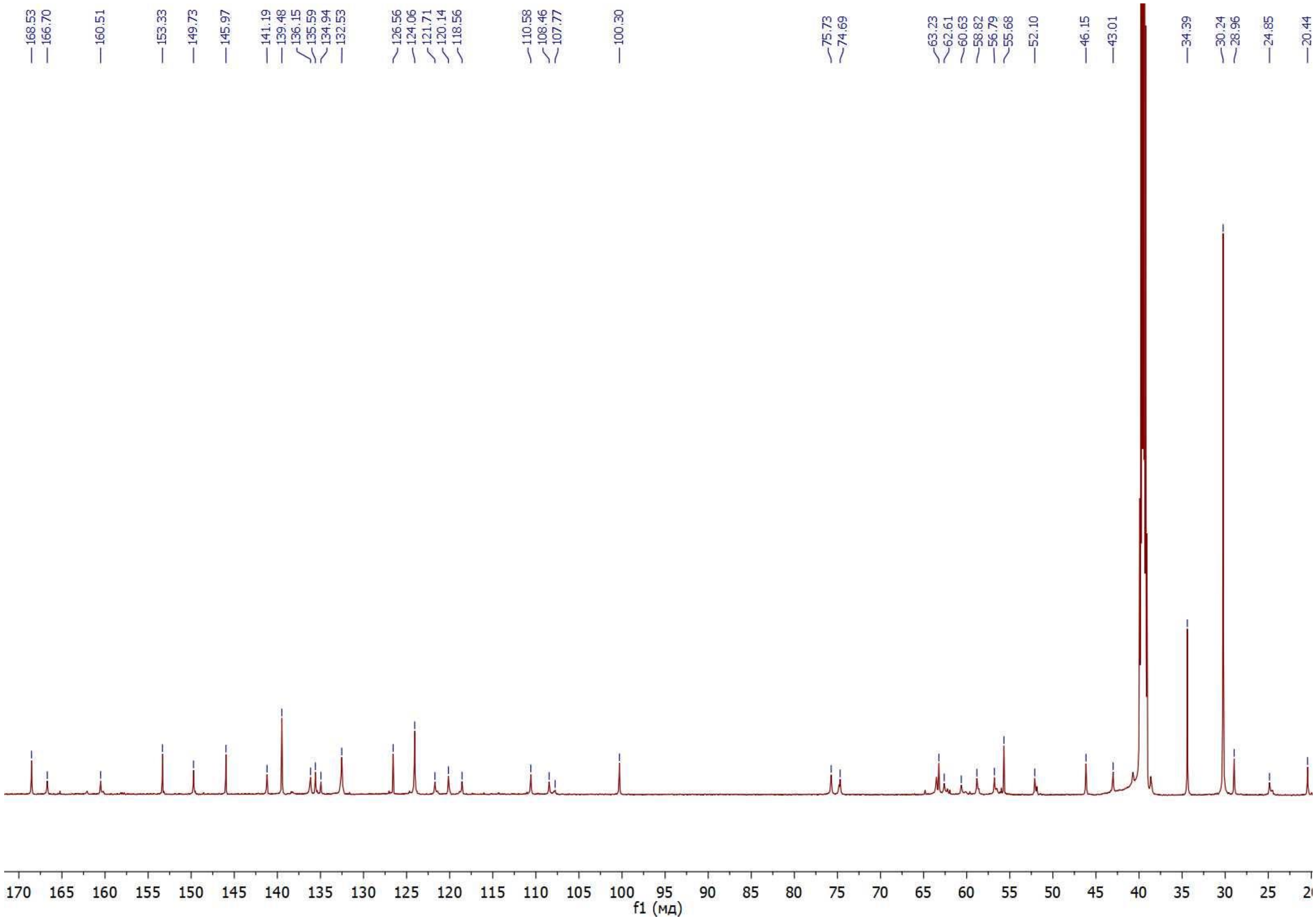

Figure S71. ^{13}C-{^{1}H} NMR spectrum of compound **3m** (151 MHz, DMSO-d_6)

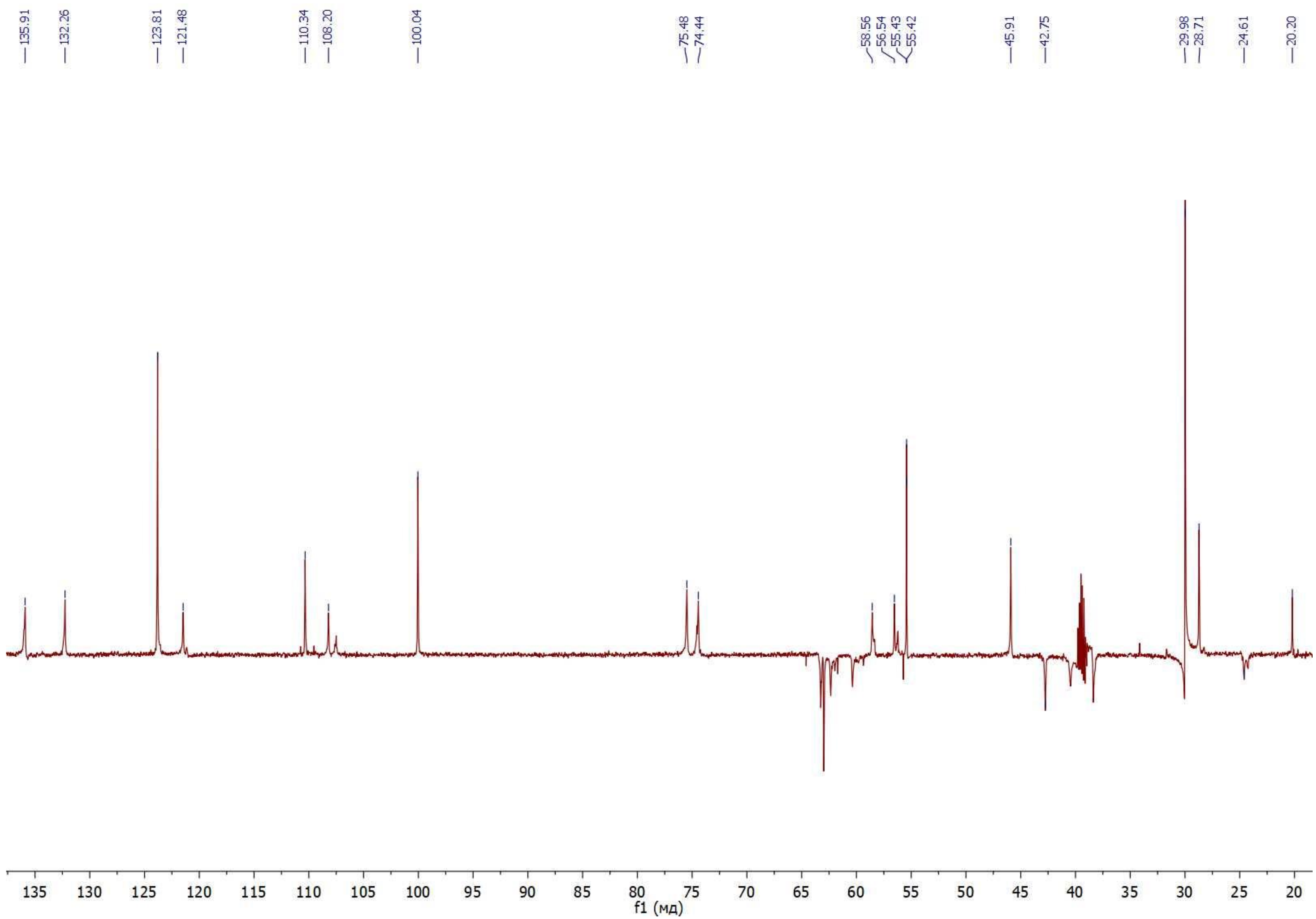

Figure S72. ^{13}C (dept) NMR spectrum of compound **3m** (151 MHz, DMSO-d_6)

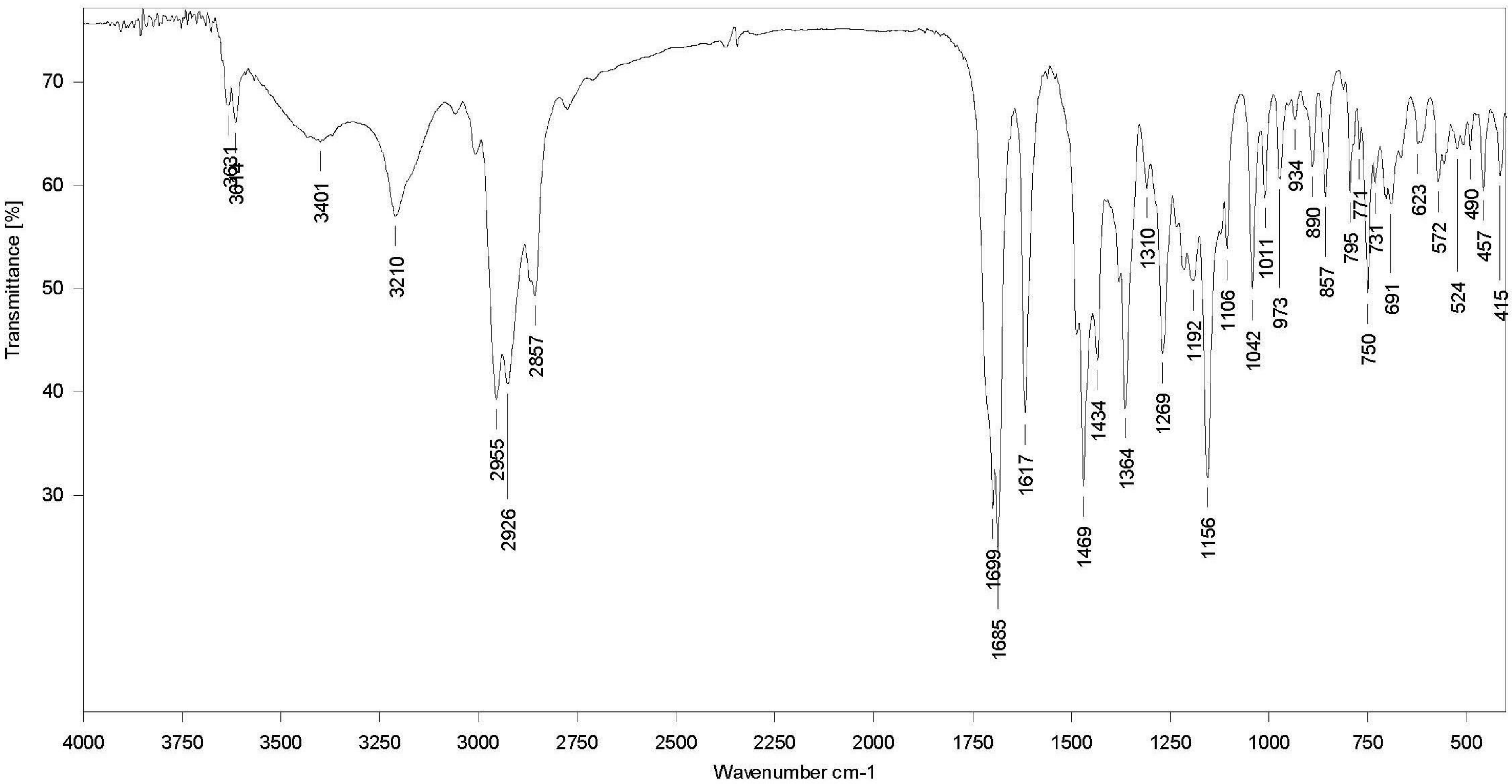

Figure S73. IR spectrum of compound **3a** (in KBr pellet)

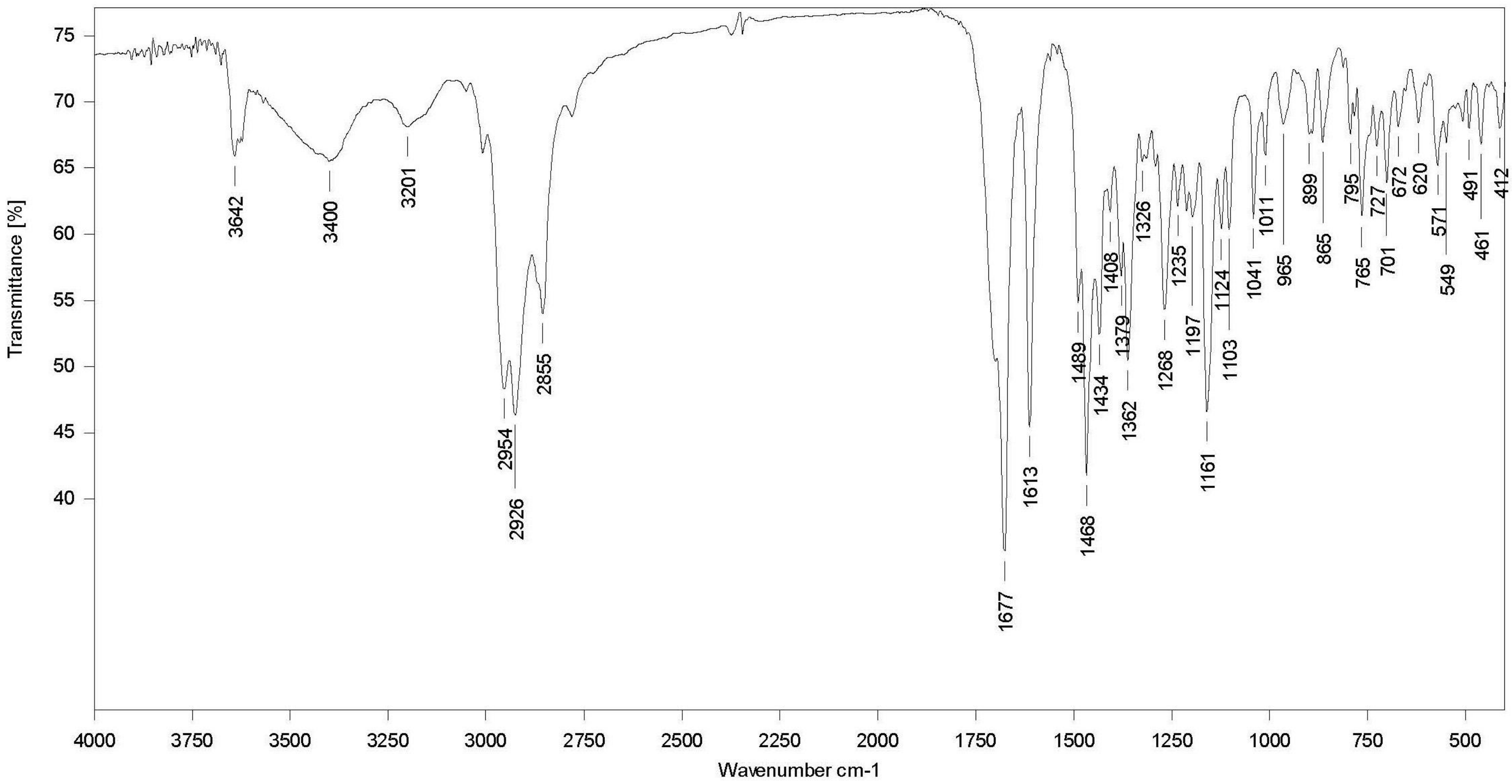

Figure S74. IR spectrum of compound **3b** (in KBr pellet)

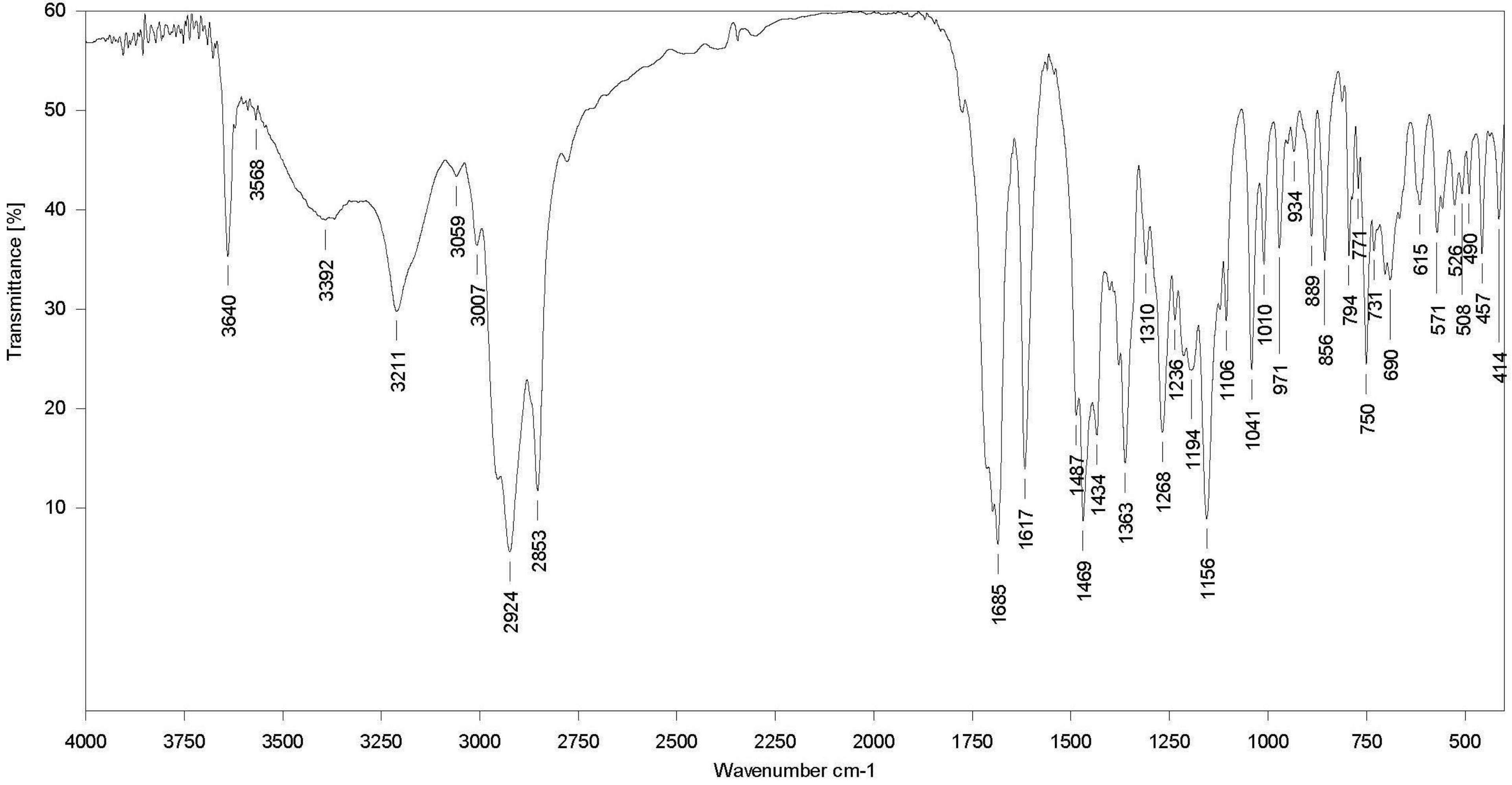

Figure S75. IR spectrum of compound **3c** (in KBr pellet)

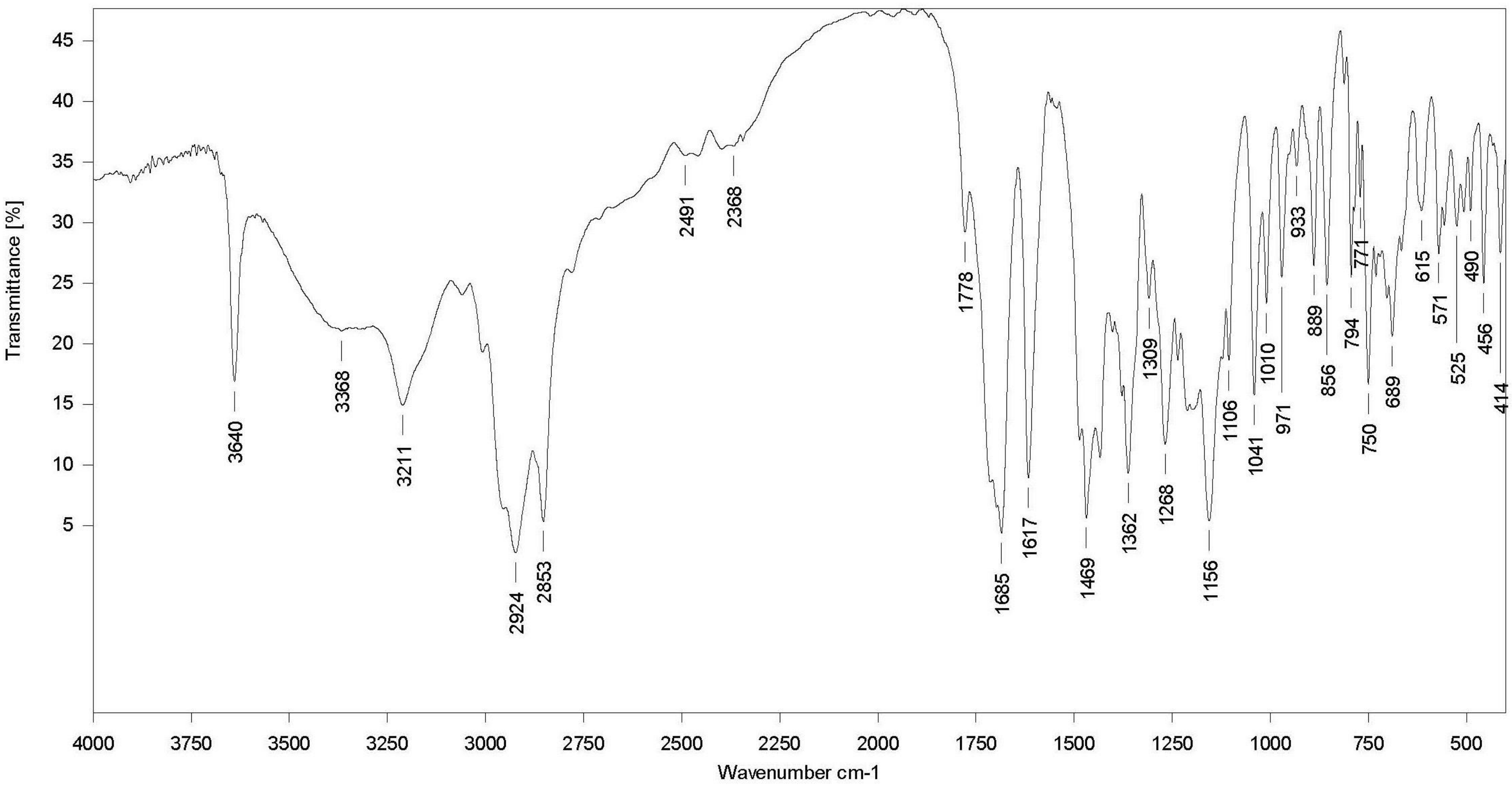

Figure S76. IR spectrum of compound **3d** (in KBr pellet)

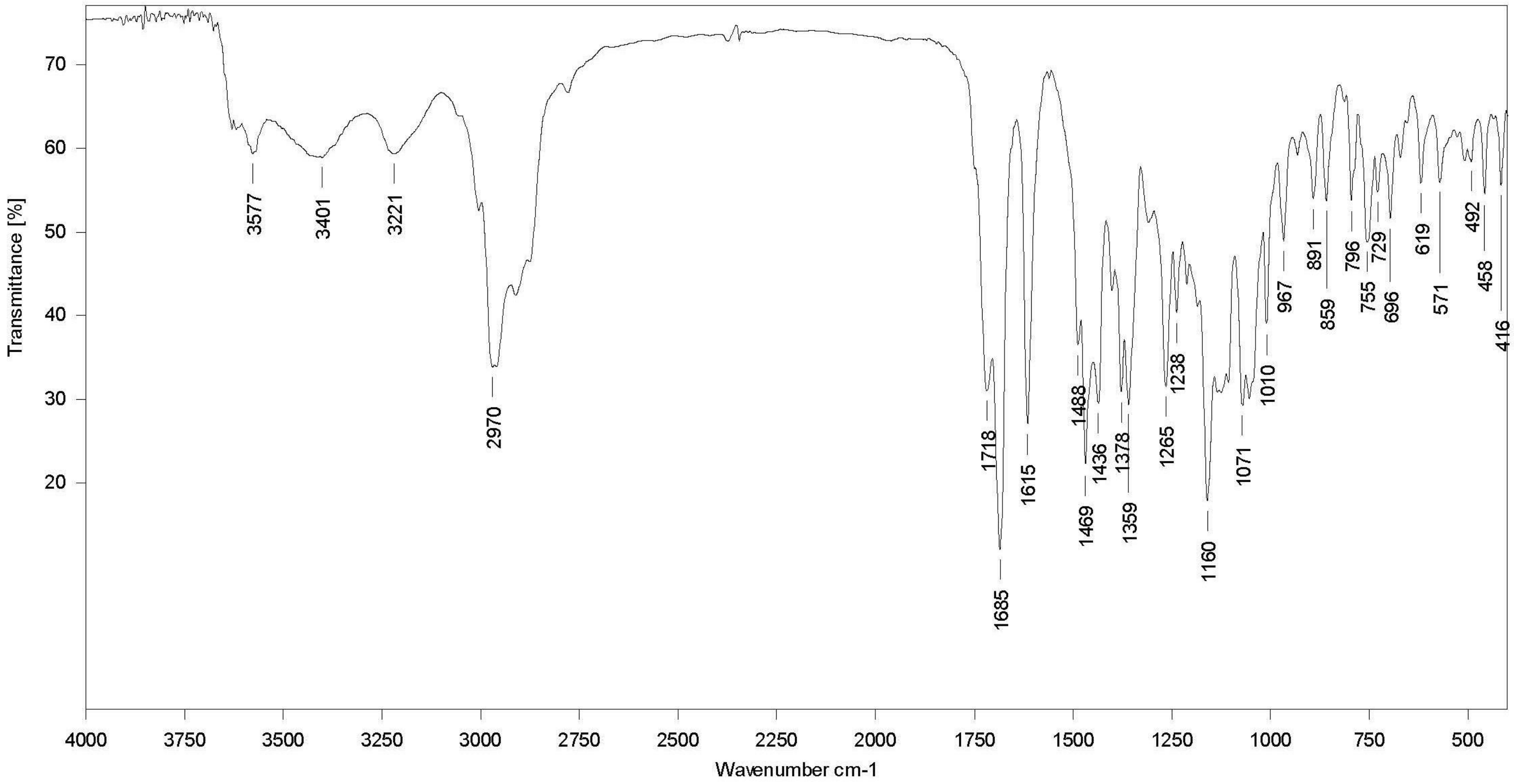

Figure S77. IR spectrum of compound **3e** (in KBr pellet)

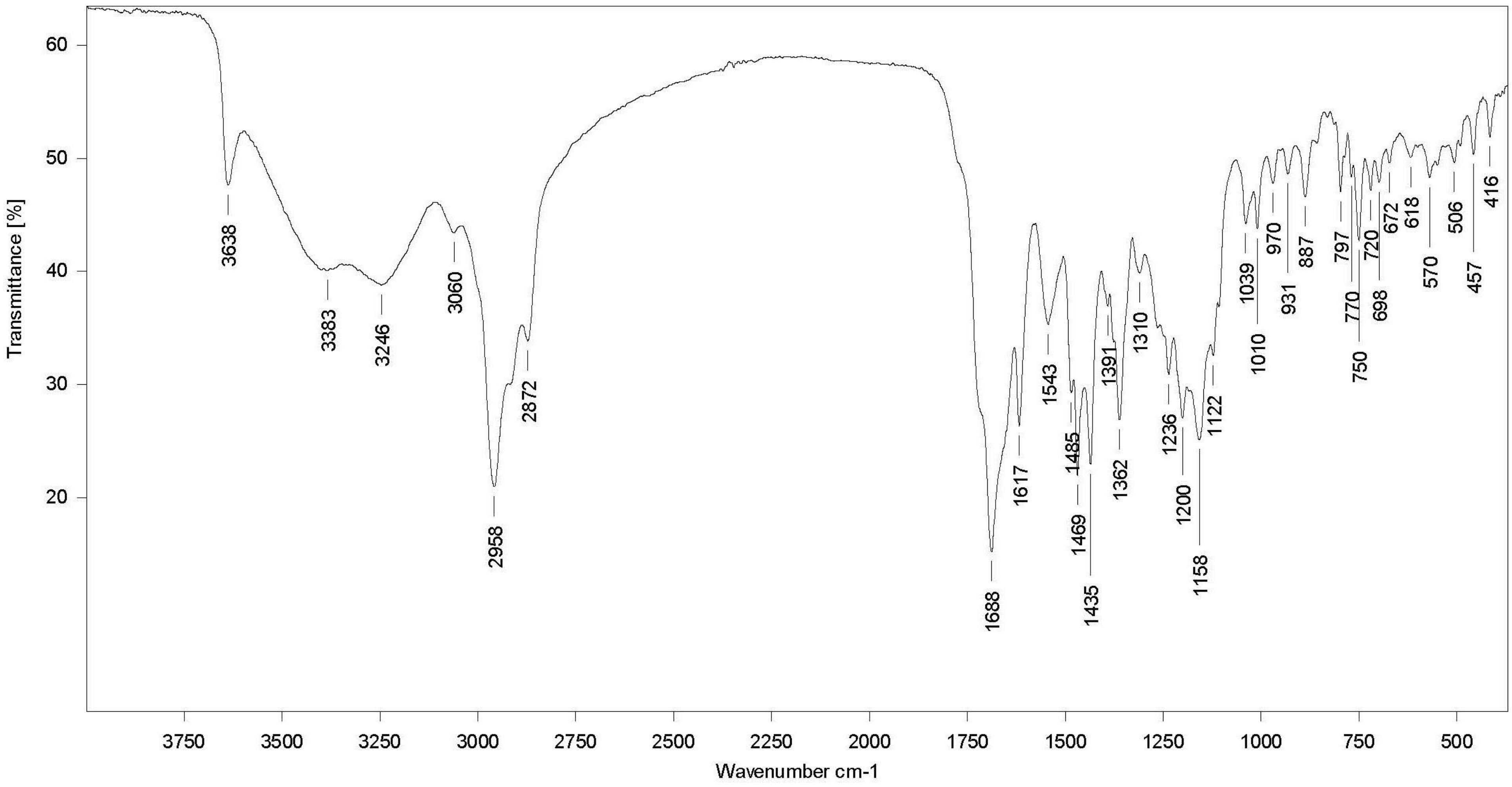

Figure S78. IR spectrum of compound **3f** (in KBr pellet)

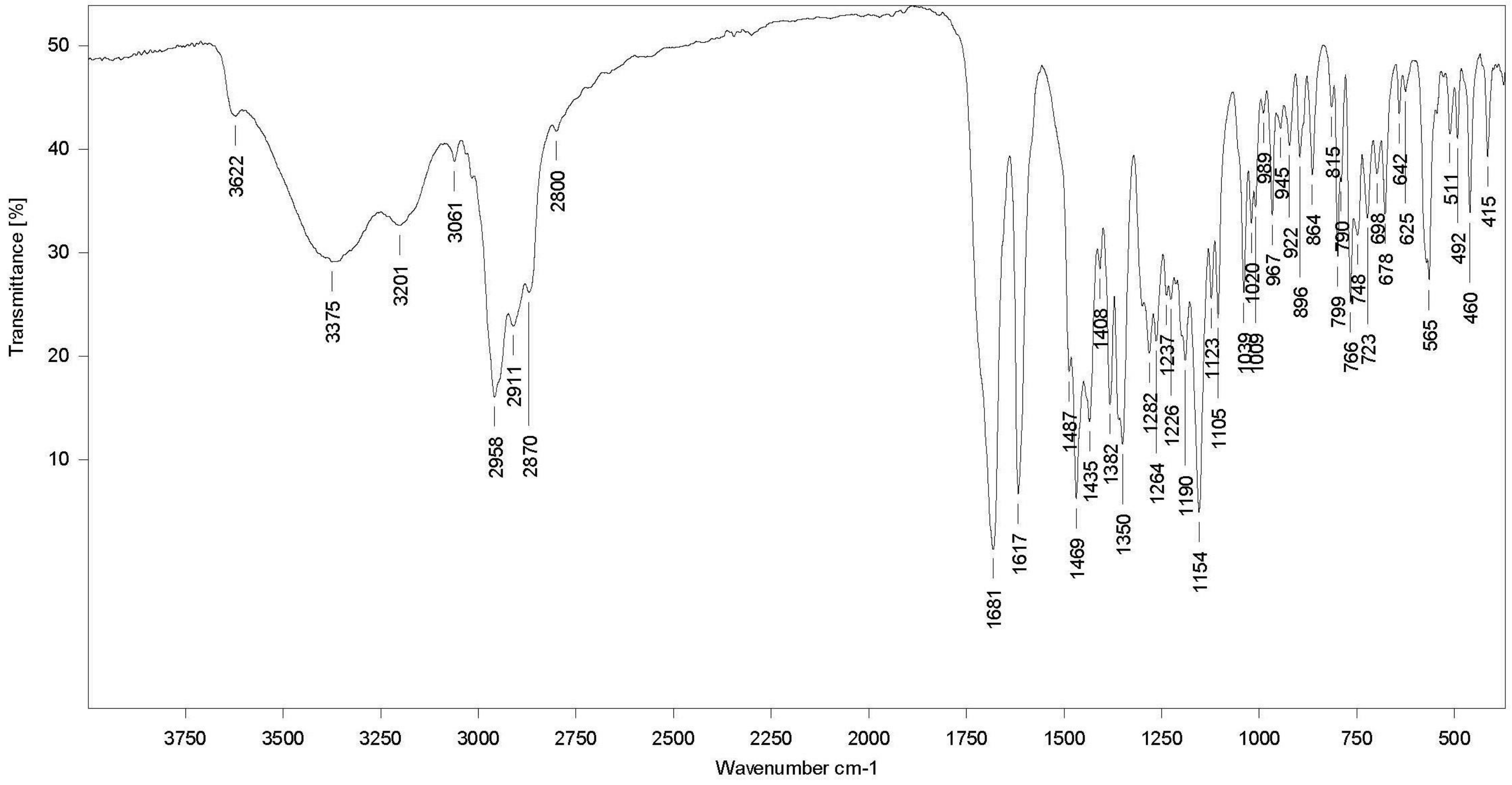

Figure S79. IR spectrum of compound **3g** (in KBr pellet)

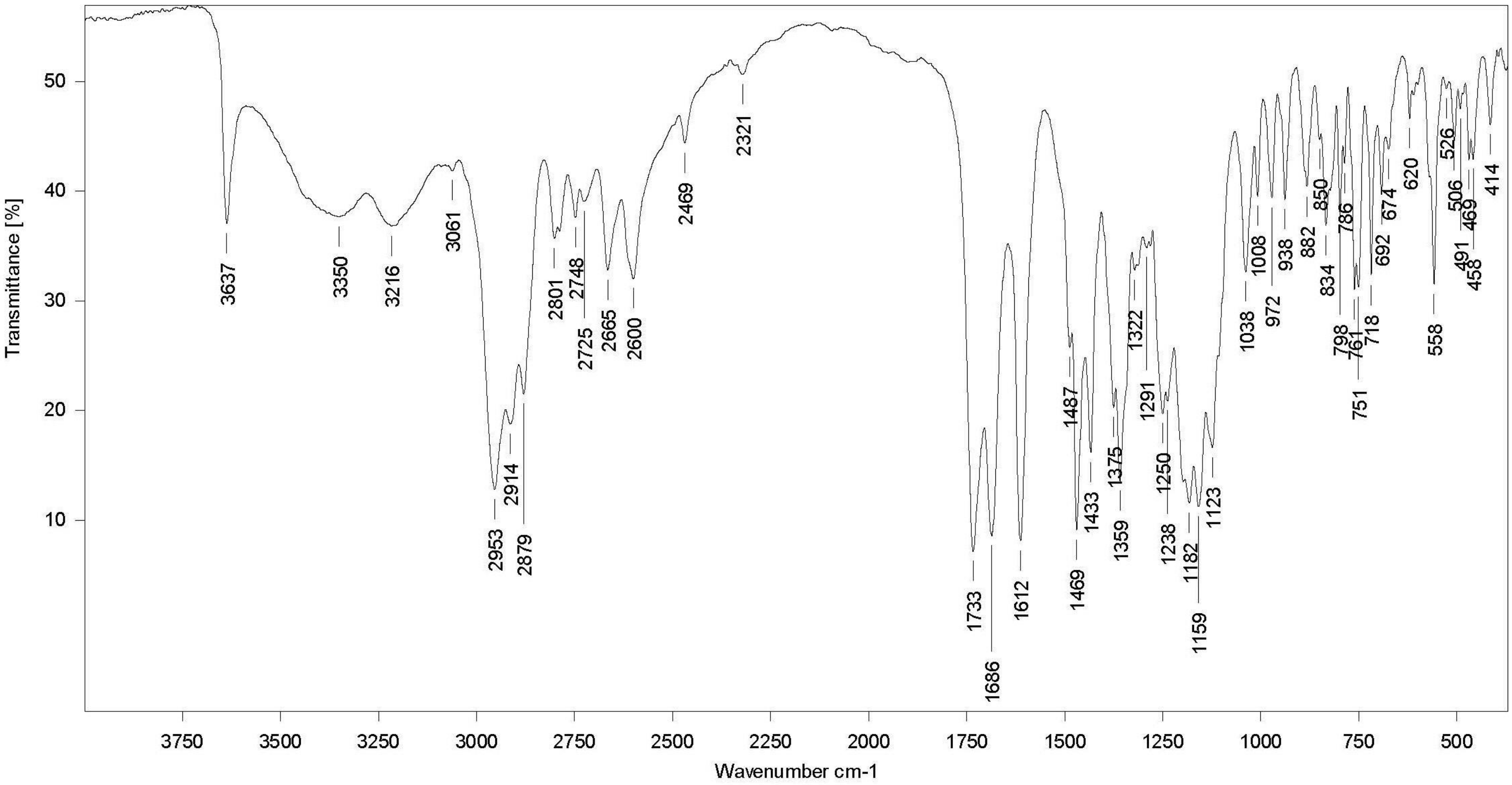

Figure S80. IR spectrum of compound **3h** (in KBr pellet)

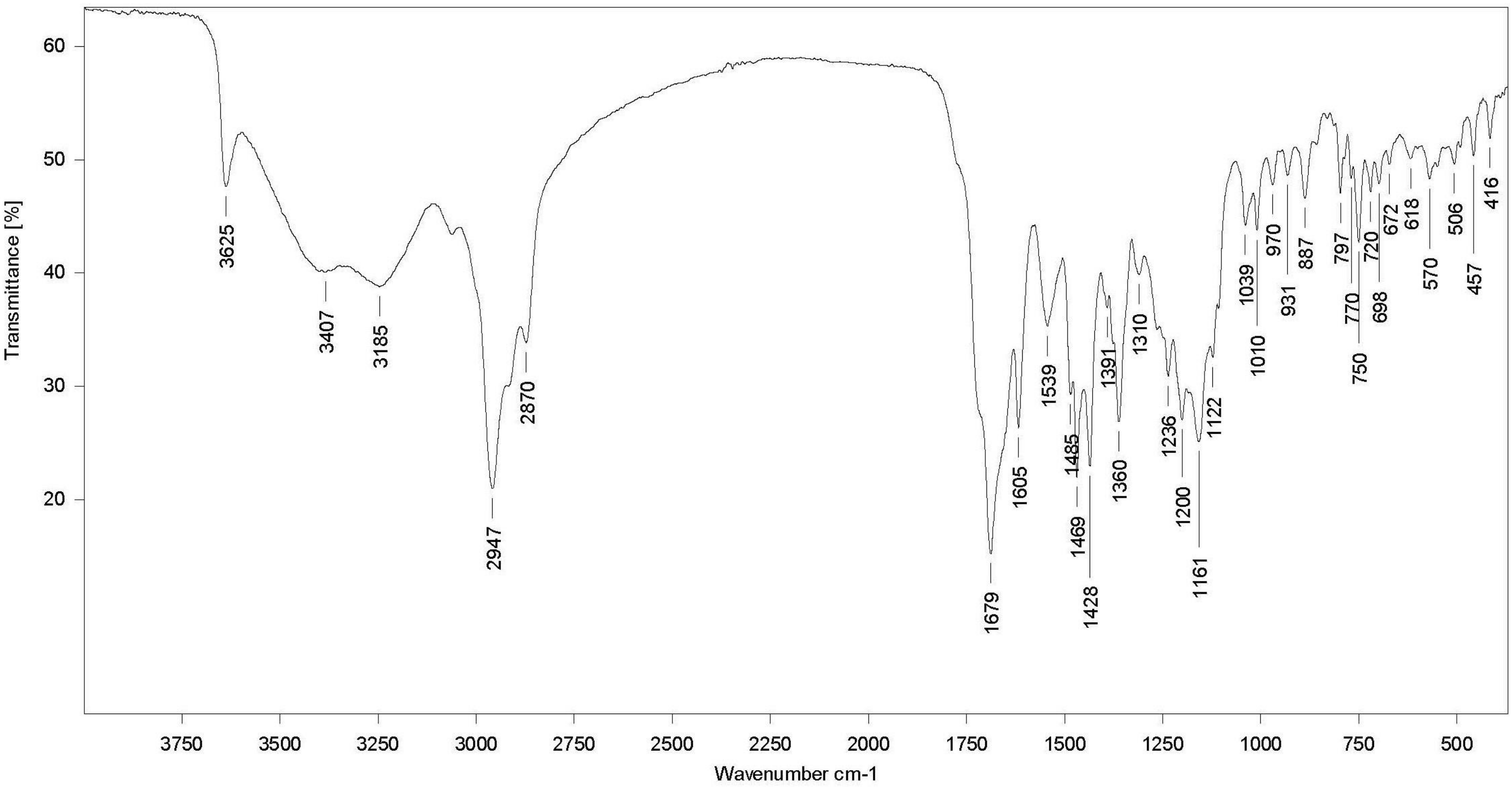

Figure S81. IR spectrum of compound **3i** (in KBr pellet)

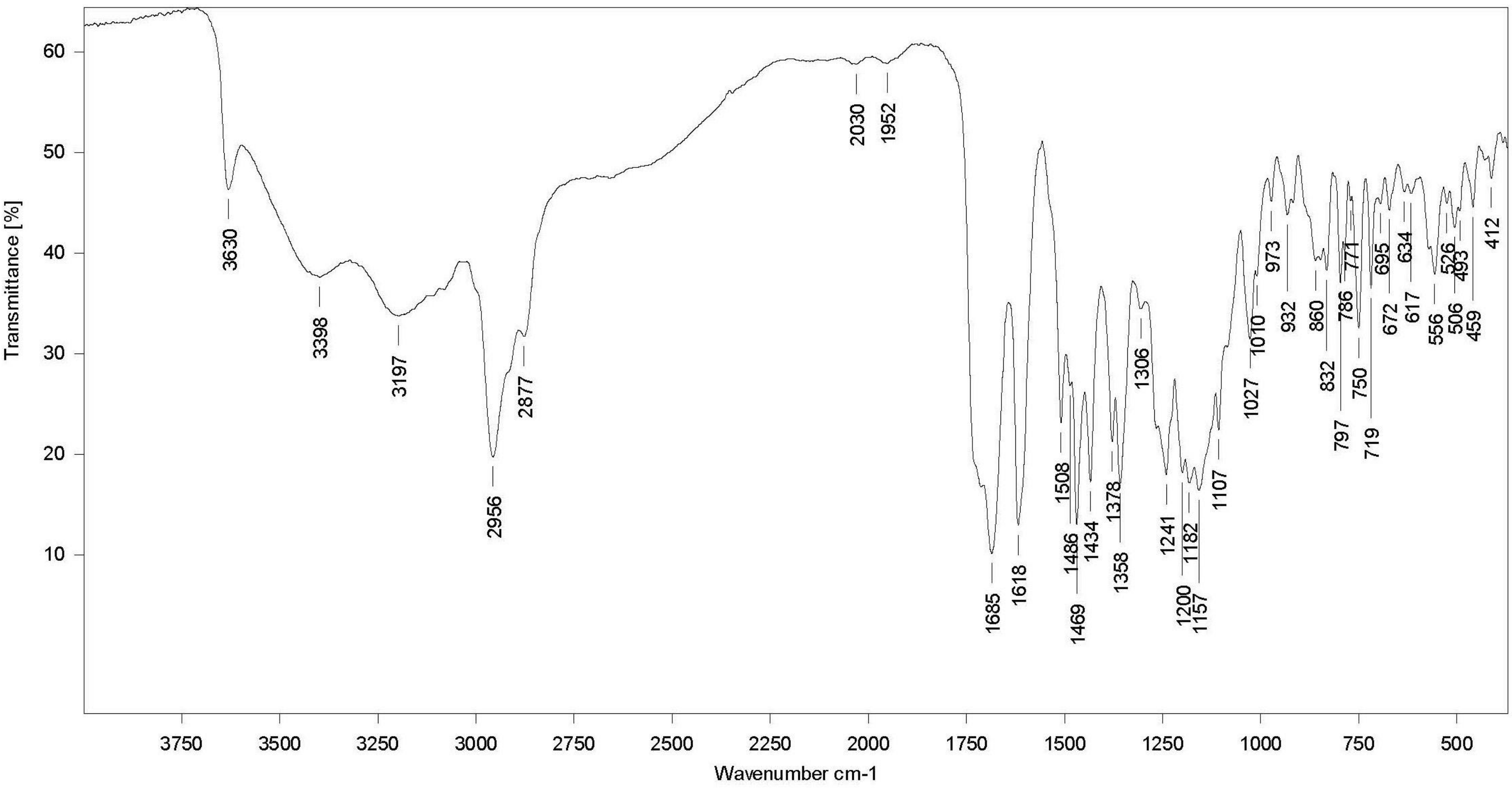

Figure S82. IR spectrum of compound **3j** (in KBr pellet)

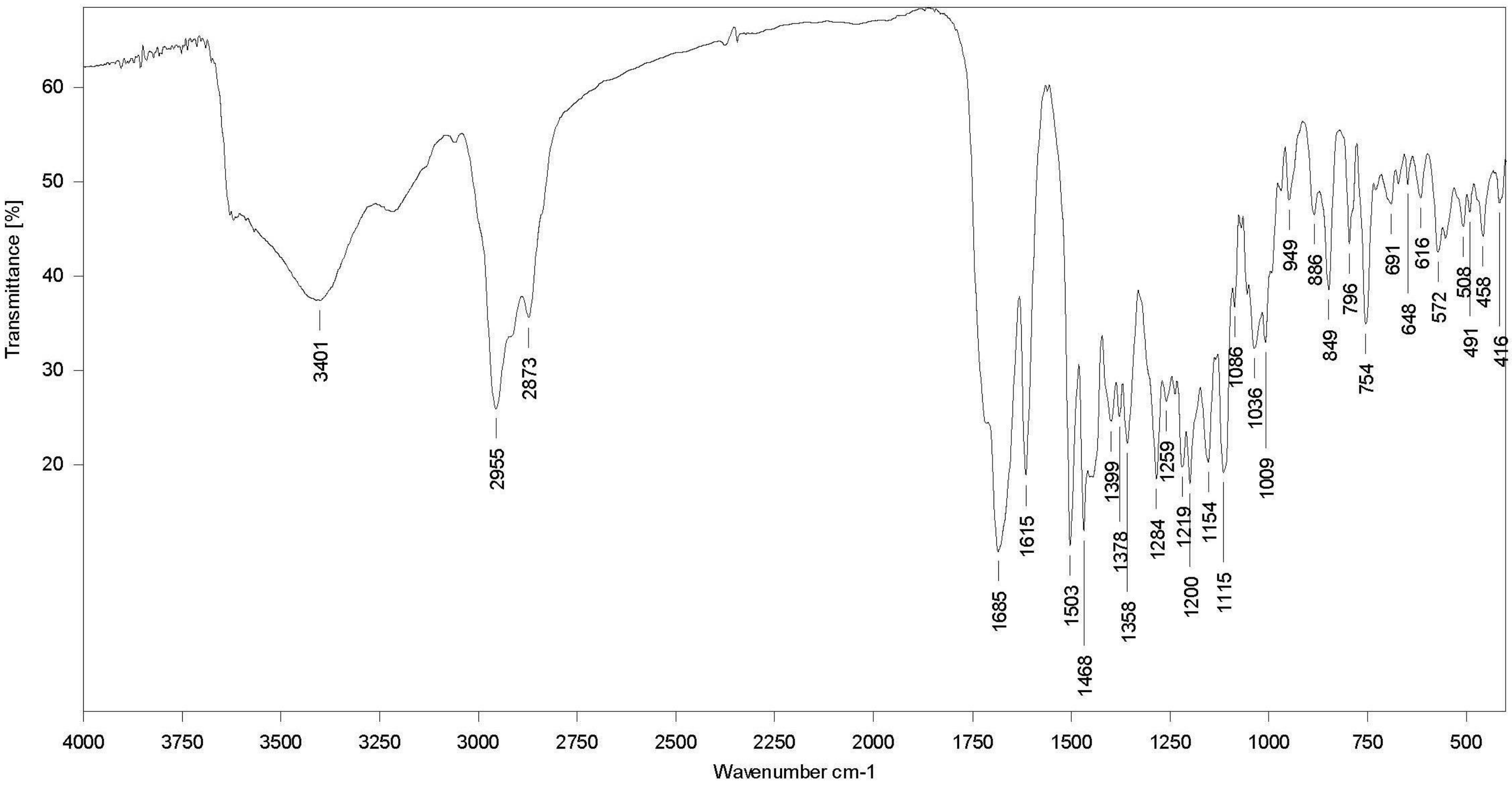

Figure S83. IR spectrum of compound **3k** (in KBr pellet)

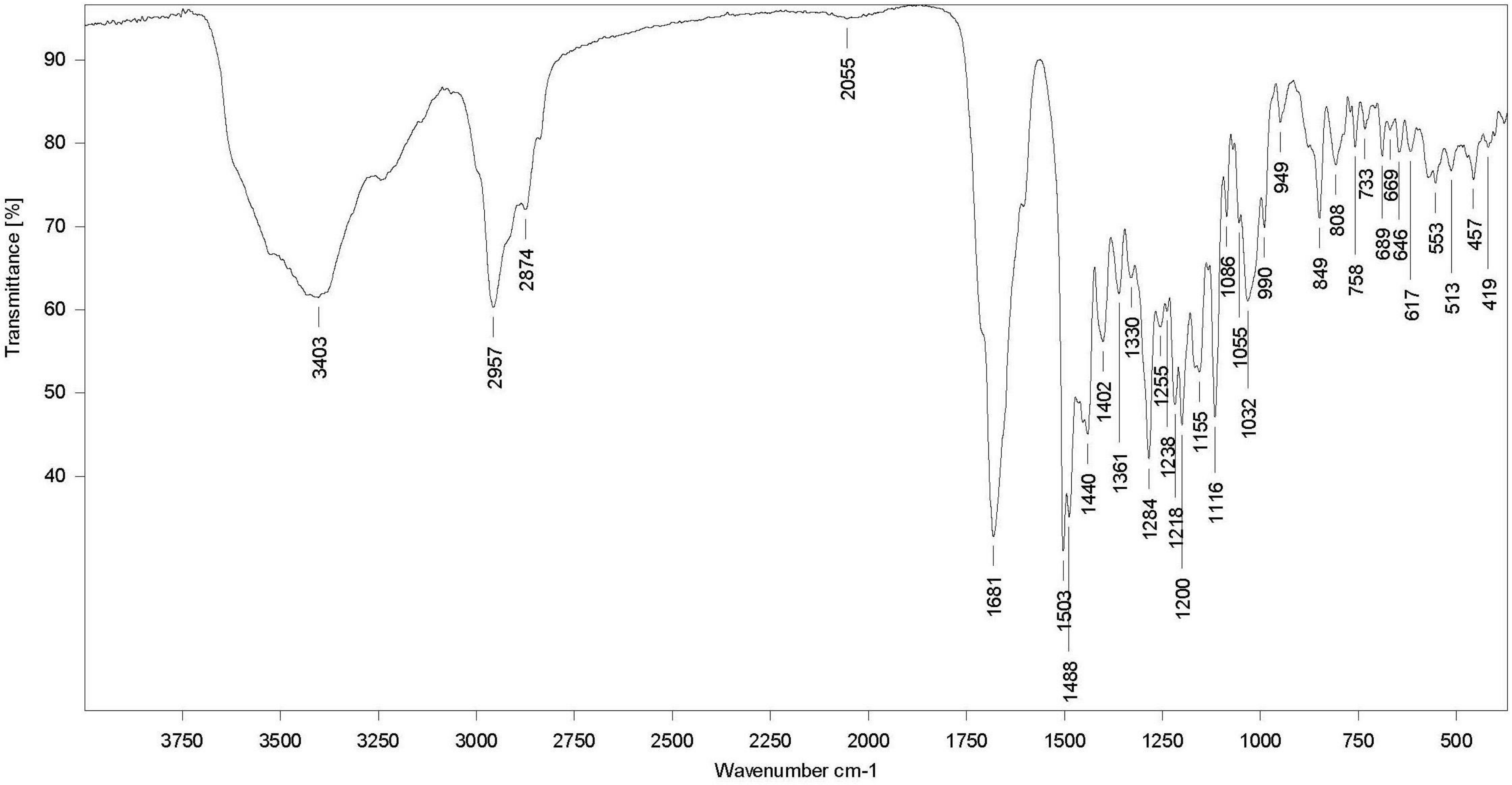

Figure S84. IR spectrum of compound **3l** (in KBr pellet)

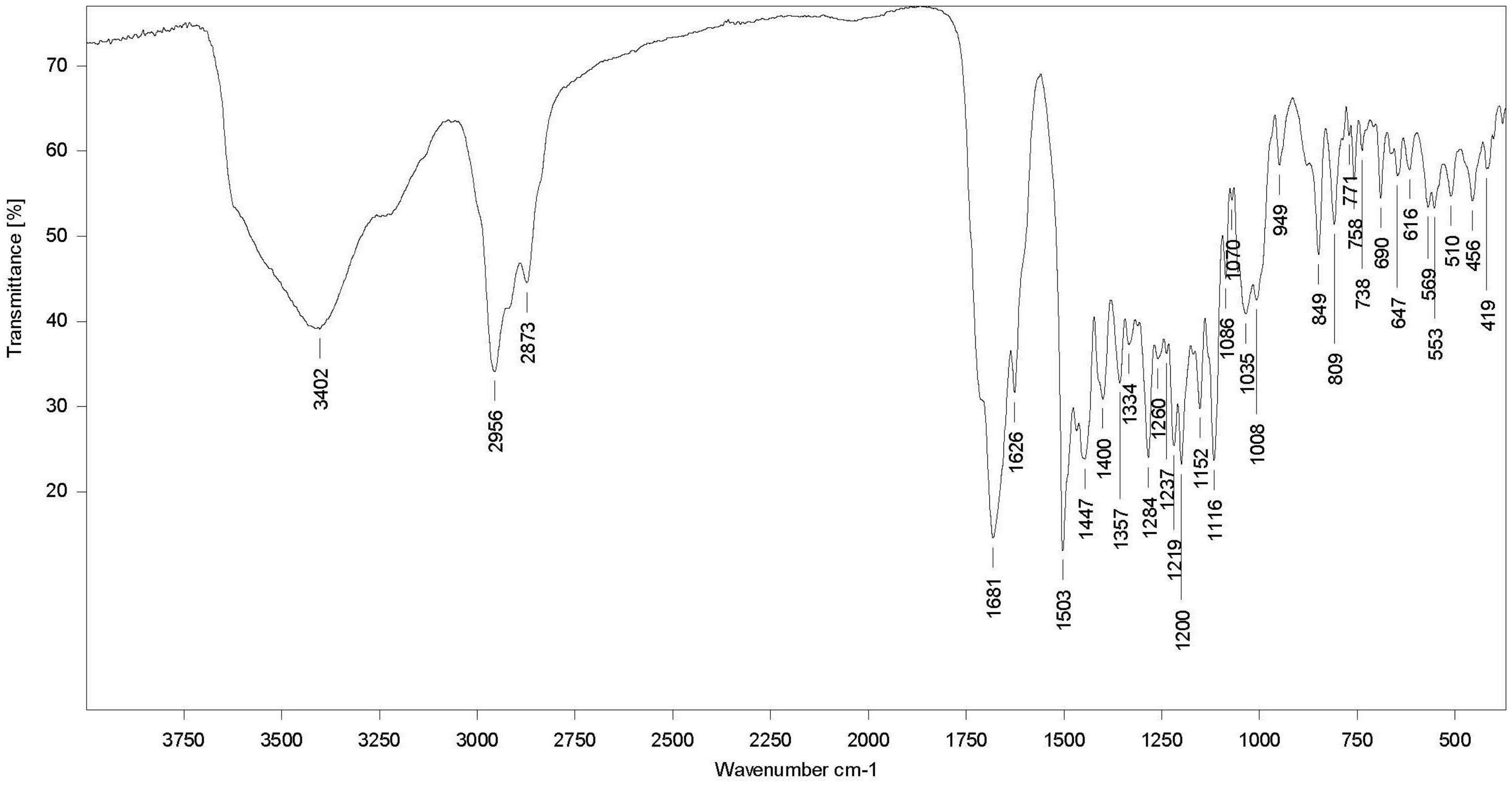

Figure S85. IR spectrum of compound **3m** (in KBr pellet)

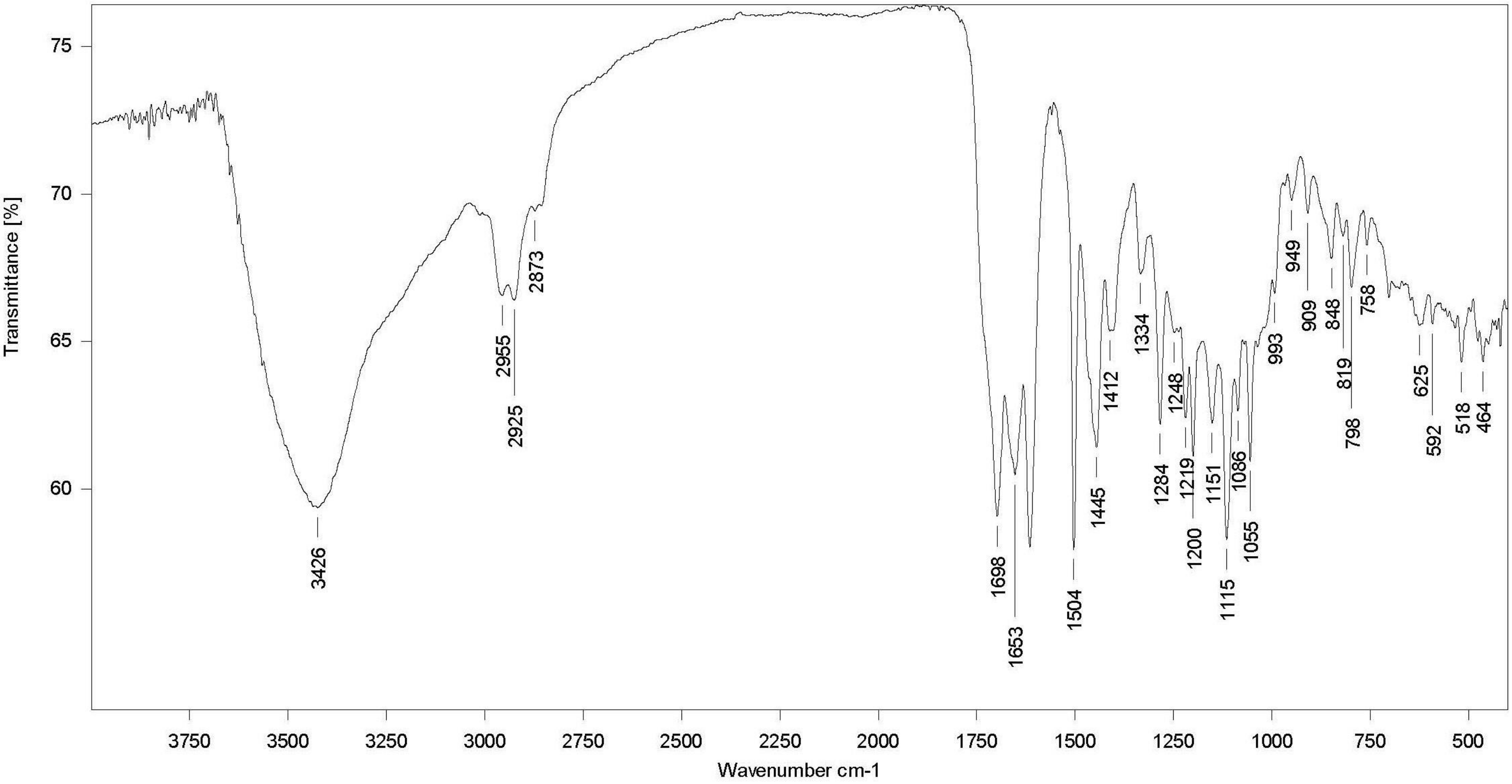

Figure S86. IR spectrum of compound **3n** (in KBr pellet)

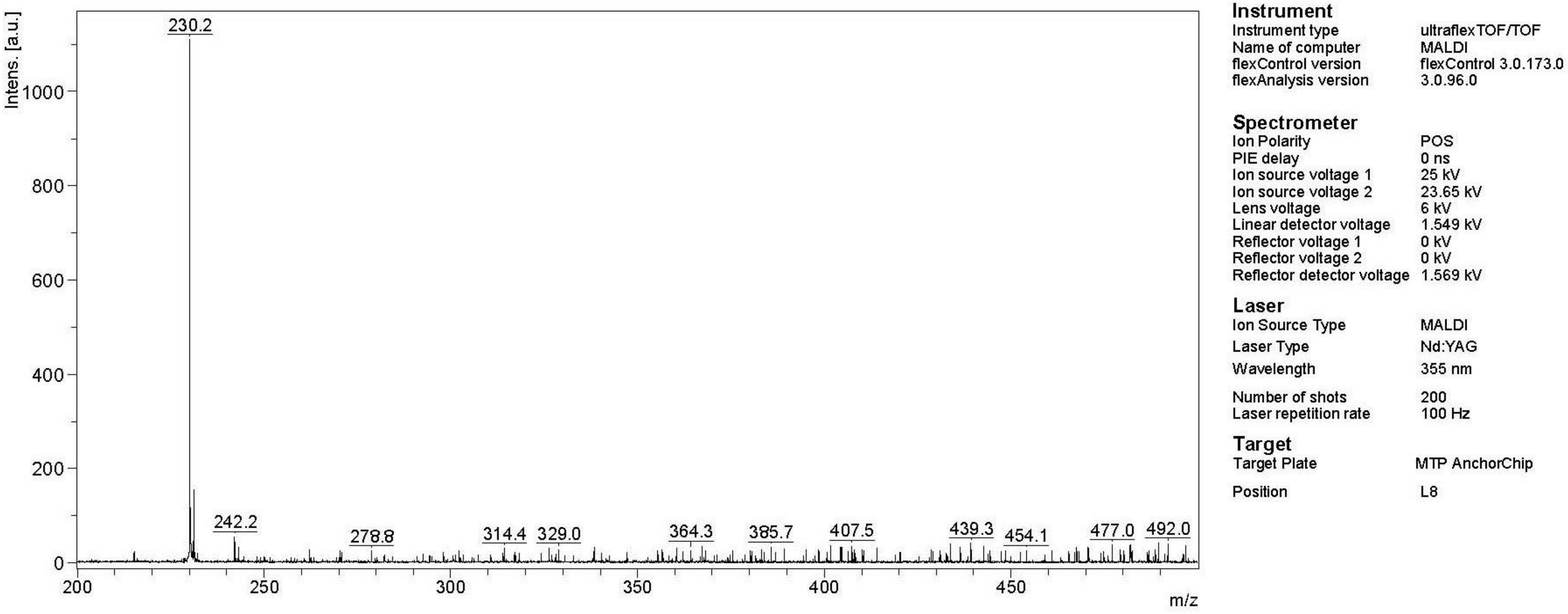

Figure S87. MALDI spectrum of compound **1a**

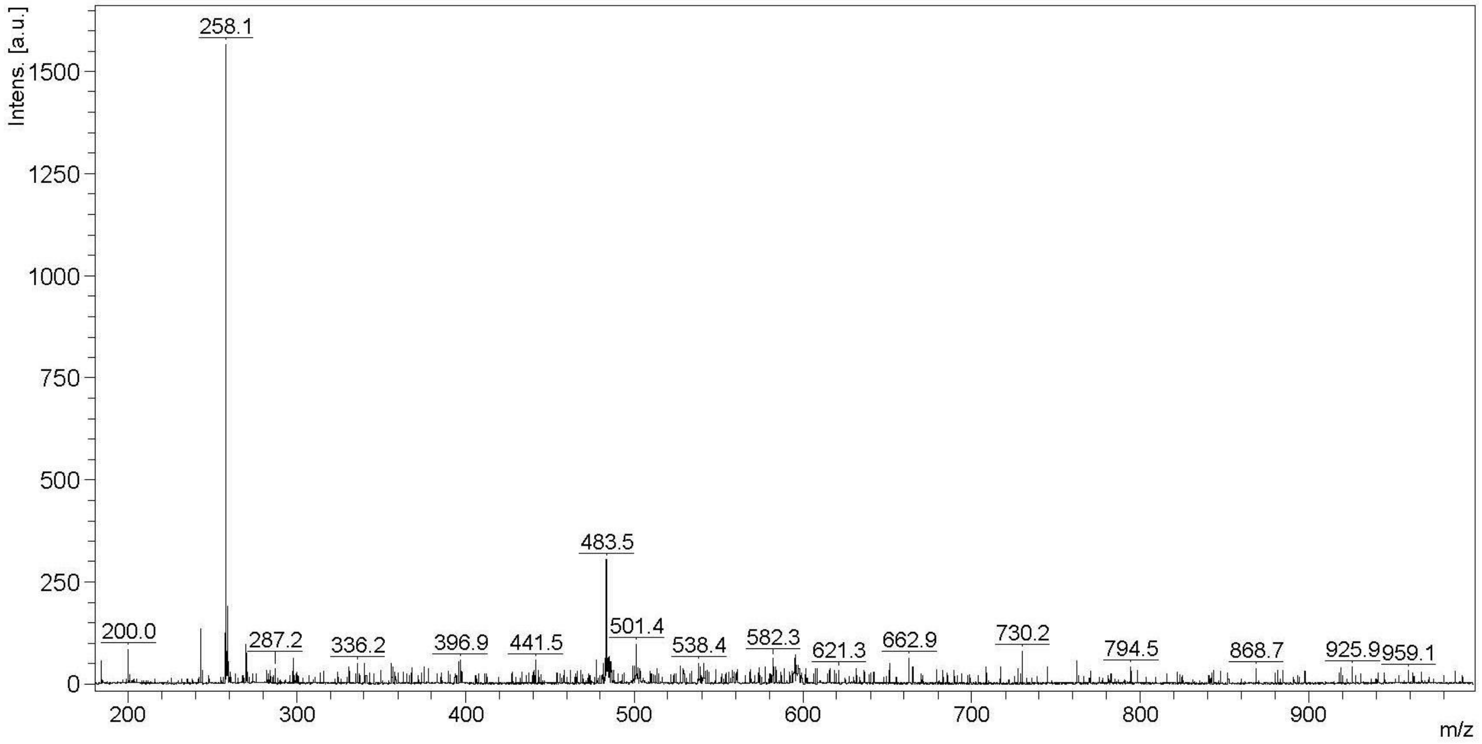

Instrument

Instrument type	ultraflexTOF/TOF
Name of computer	MALDI
flexControl version	flexControl 3.0.173.0
flexAnalysis version	3.0.96.0

Spectrometer

Ion Polarity	POS
PIE delay	0 ns
Ion source voltage 1	25 kV
Ion source voltage 2	23.65 kV
Lens voltage	6 kV
Linear detector voltage	1.549 kV
Reflector voltage 1	0 kV
Reflector voltage 2	0 kV
Reflector detector voltage	1.569 kV

Laser

Ion Source Type	MALDI
Laser Type	Nd:YAG
Wavelength	355 nm
Number of shots	200
Laser repetition rate	100 Hz

Target

Target Plate	MTP AnchorChip
Position	E2

Figure S88. MALDI spectrum of compound **1b**

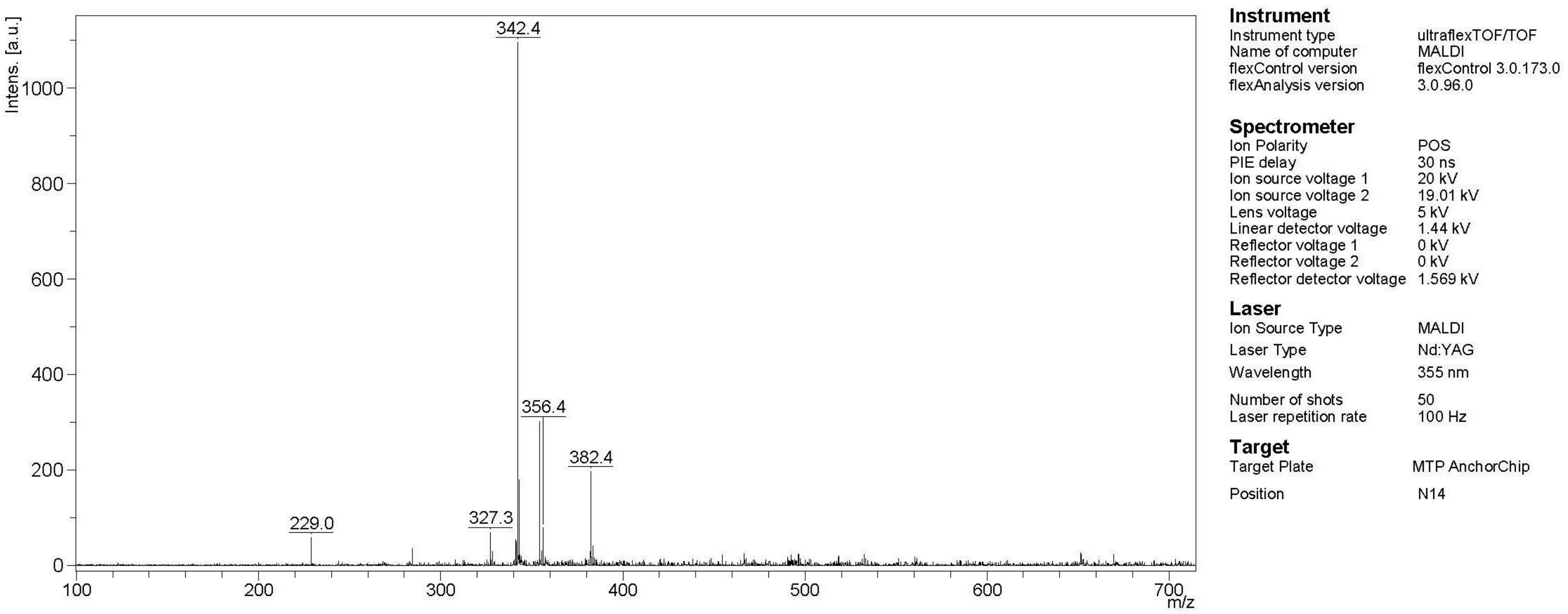

Figure S89. MALDI spectrum of compound **1c**

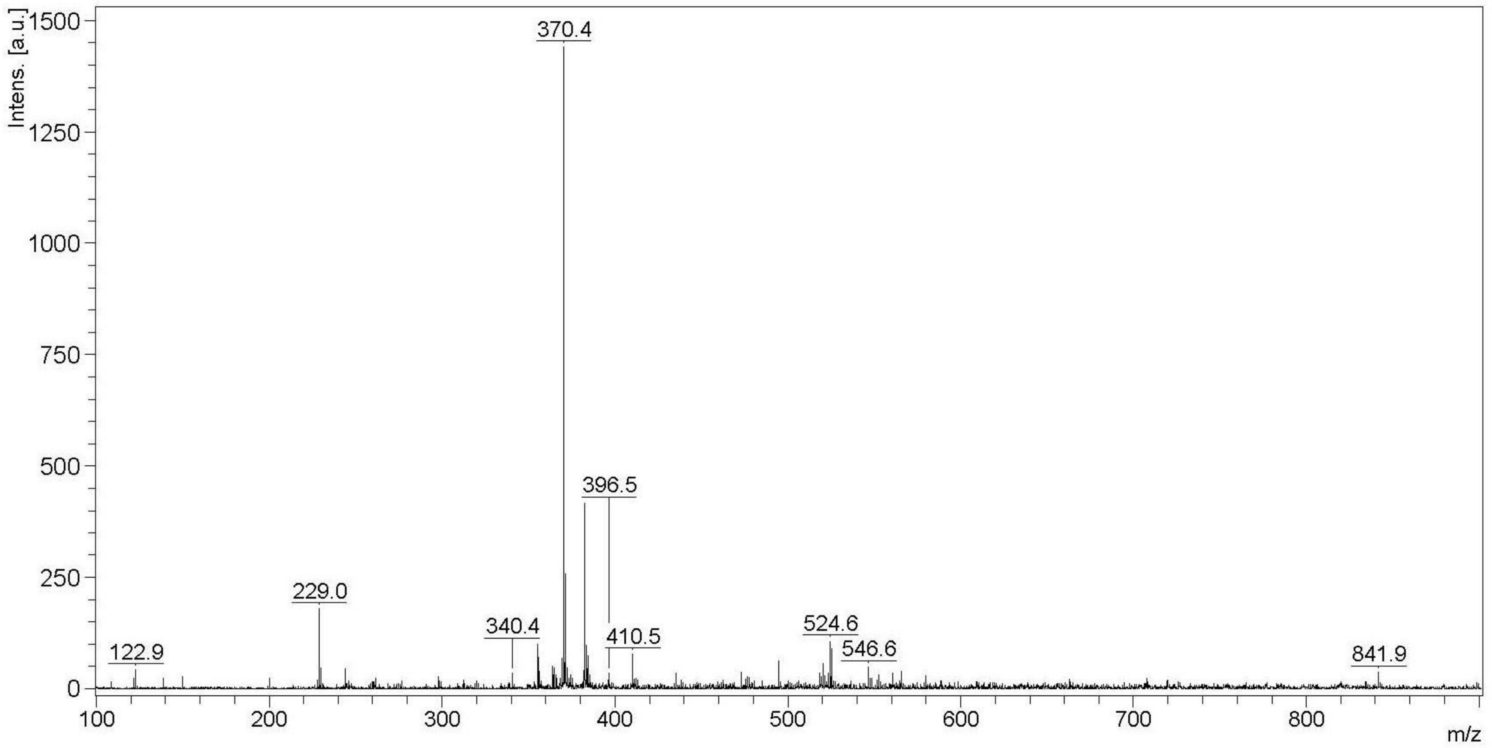

Instrument

Instrument type	ultraflexTOF/TOF
Name of computer	MALDI
flexControl version	flexControl 3.0.173.0
flexAnalysis version	3.0.96.0

Spectrometer

Ion Polarity	POS
PIE delay	30 ns
Ion source voltage 1	20 kV
Ion source voltage 2	19.01 kV
Lens voltage	5 kV
Linear detector voltage	1.44 kV
Reflector voltage 1	0 kV
Reflector voltage 2	0 kV
Reflector detector voltage	1.569 kV

Laser

Ion Source Type	MALDI
Laser Type	Nd:YAG
Wavelength	355 nm
Number of shots	150
Laser repetition rate	100 Hz

Target

Target Plate	MTP AnchorChip
Position	N15

Figure S90. MALDI spectrum of compound **1d**

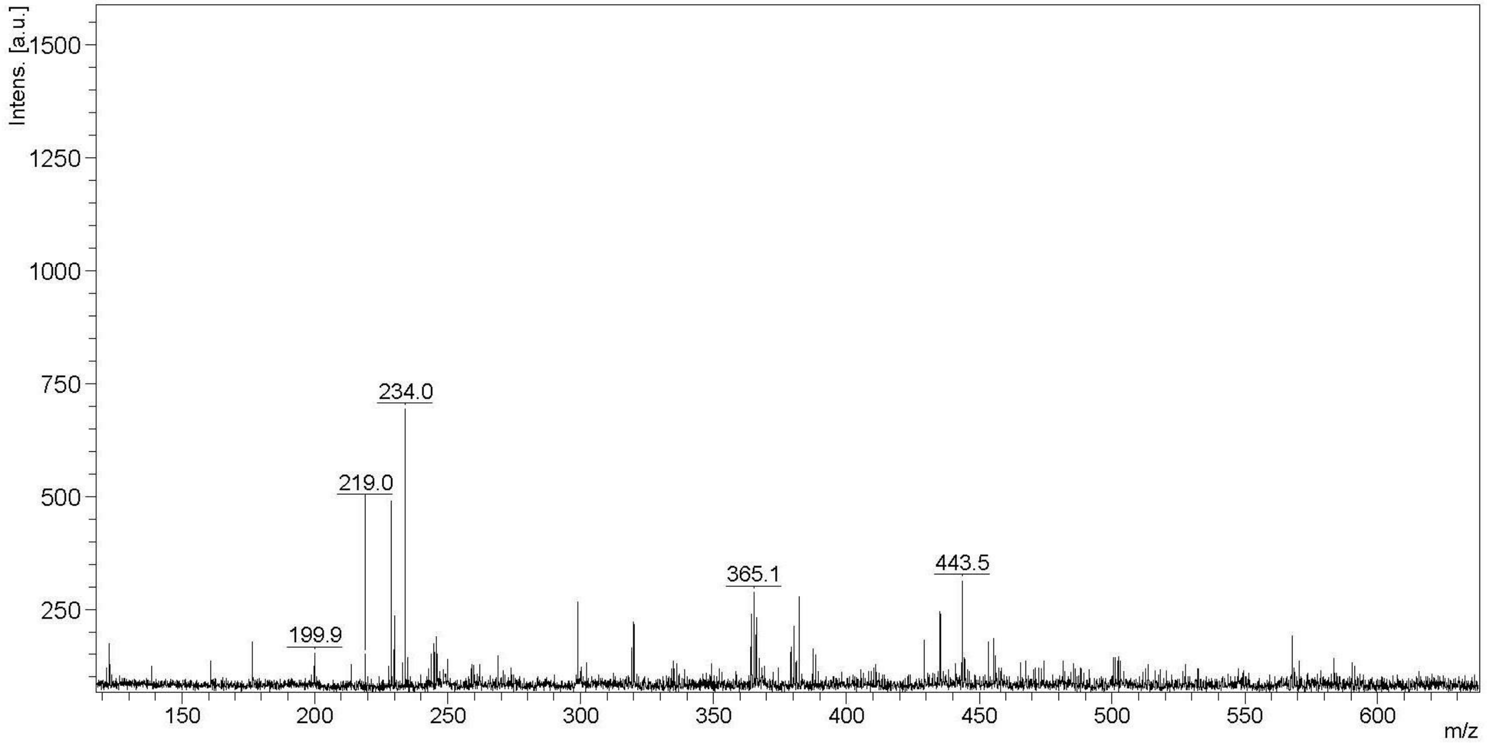

Instrument

Instrument type	ultraflexTOF/TOF
Name of computer	MALDI
flexControl version	flexControl 3.0.173.0
flexAnalysis version	3.0.96.0

Spectrometer

Ion Polarity	POS
PIE delay	50 ns
Ion source voltage 1	20 kV
Ion source voltage 2	19.01 kV
Lens voltage	6 kV
Linear detector voltage	1.498 kV
Reflector voltage 1	0 kV
Reflector voltage 2	0 kV
Reflector detector voltage	1.497 kV

Laser

Ion Source Type	MALDI
Laser Type	Nd:YAG
Wavelength	355 nm
Number of shots	100
Laser repetition rate	100 Hz

Target

Target Plate	MTP AnchorChip
Position	F11

Figure S91. MALDI spectrum of compound **1e**

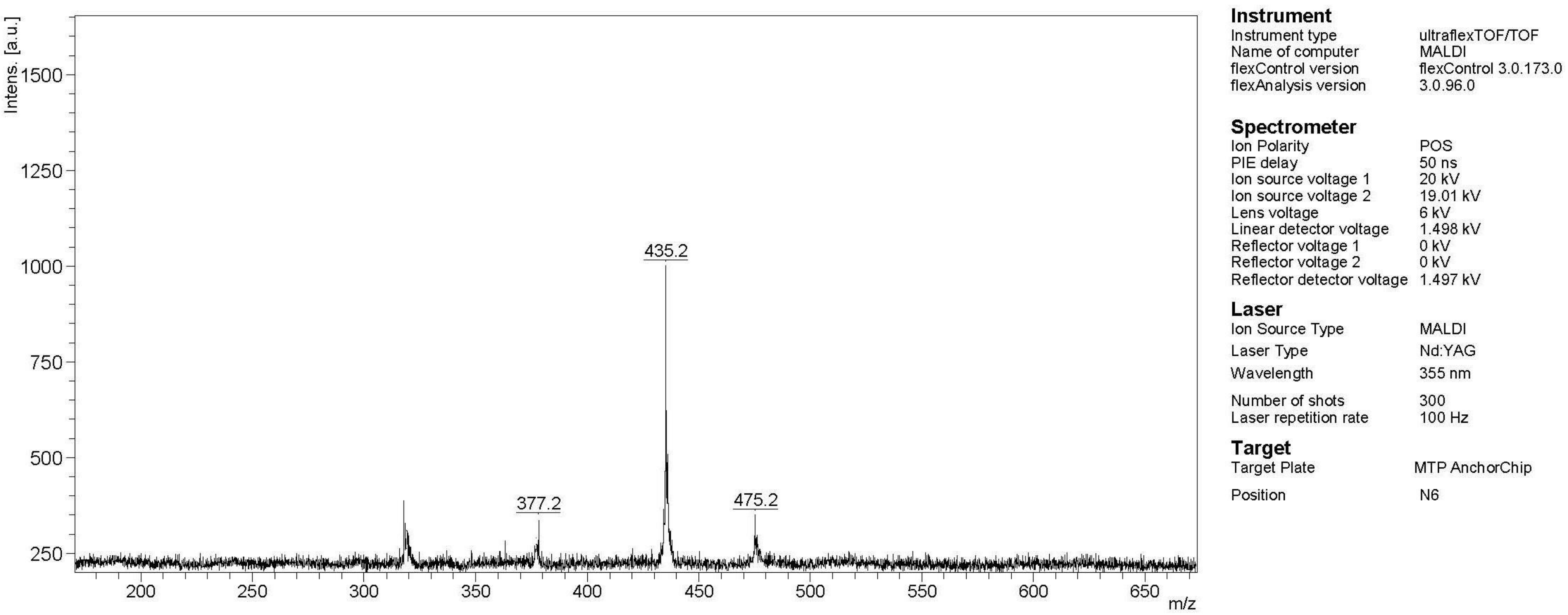

Figure S92. MALDI spectrum of compound **1f**

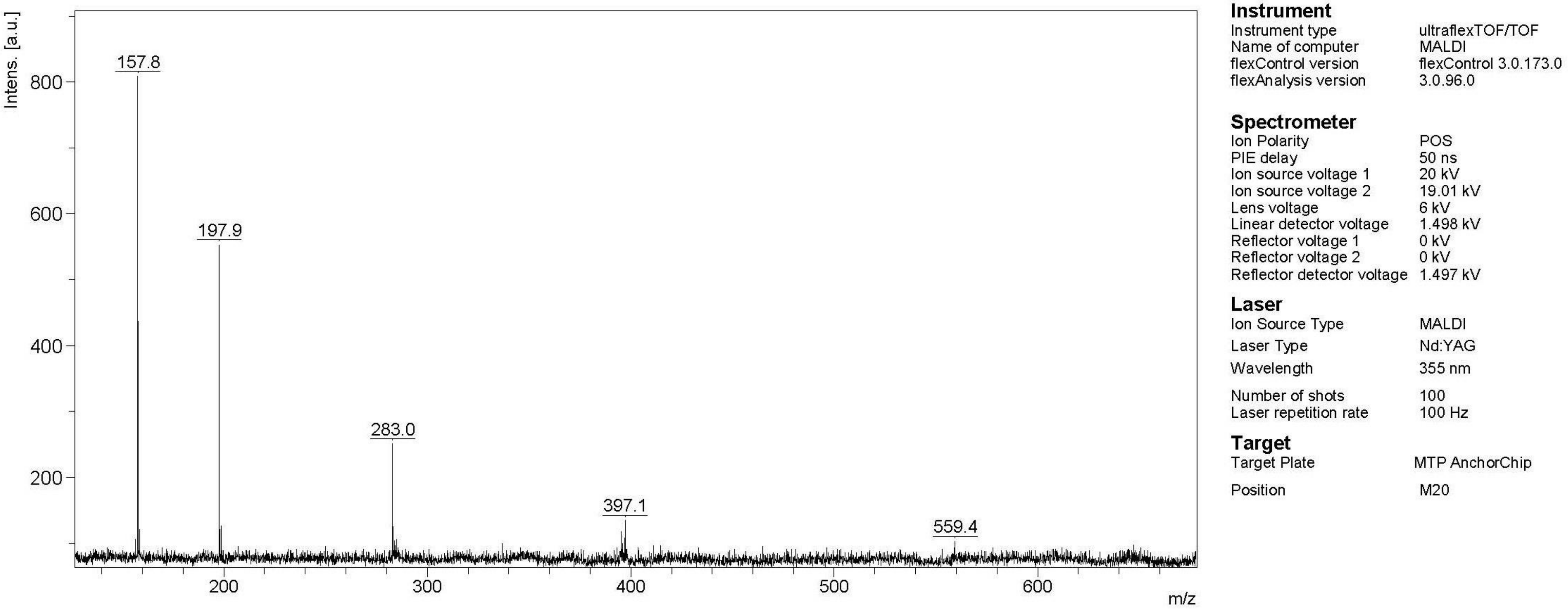

Figure S93. MALDI spectrum of compound **1g**

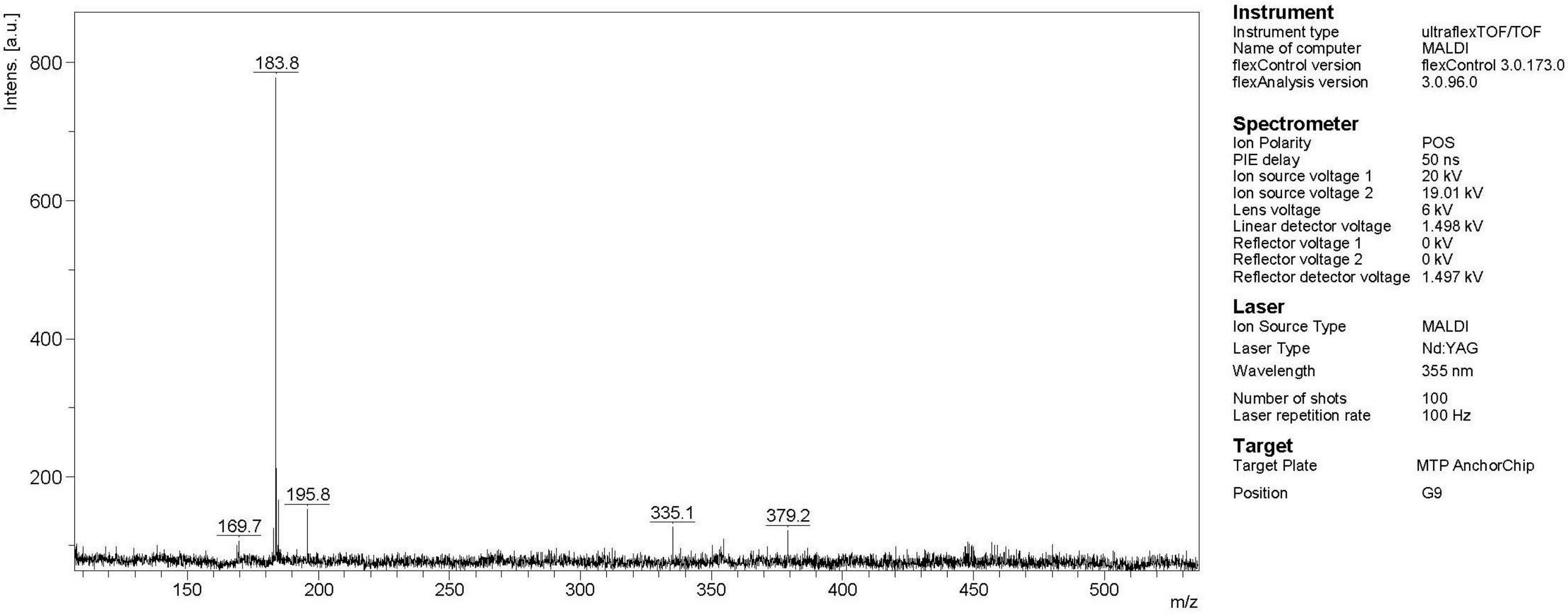

Figure S94. MALDI spectrum of compound **1h**

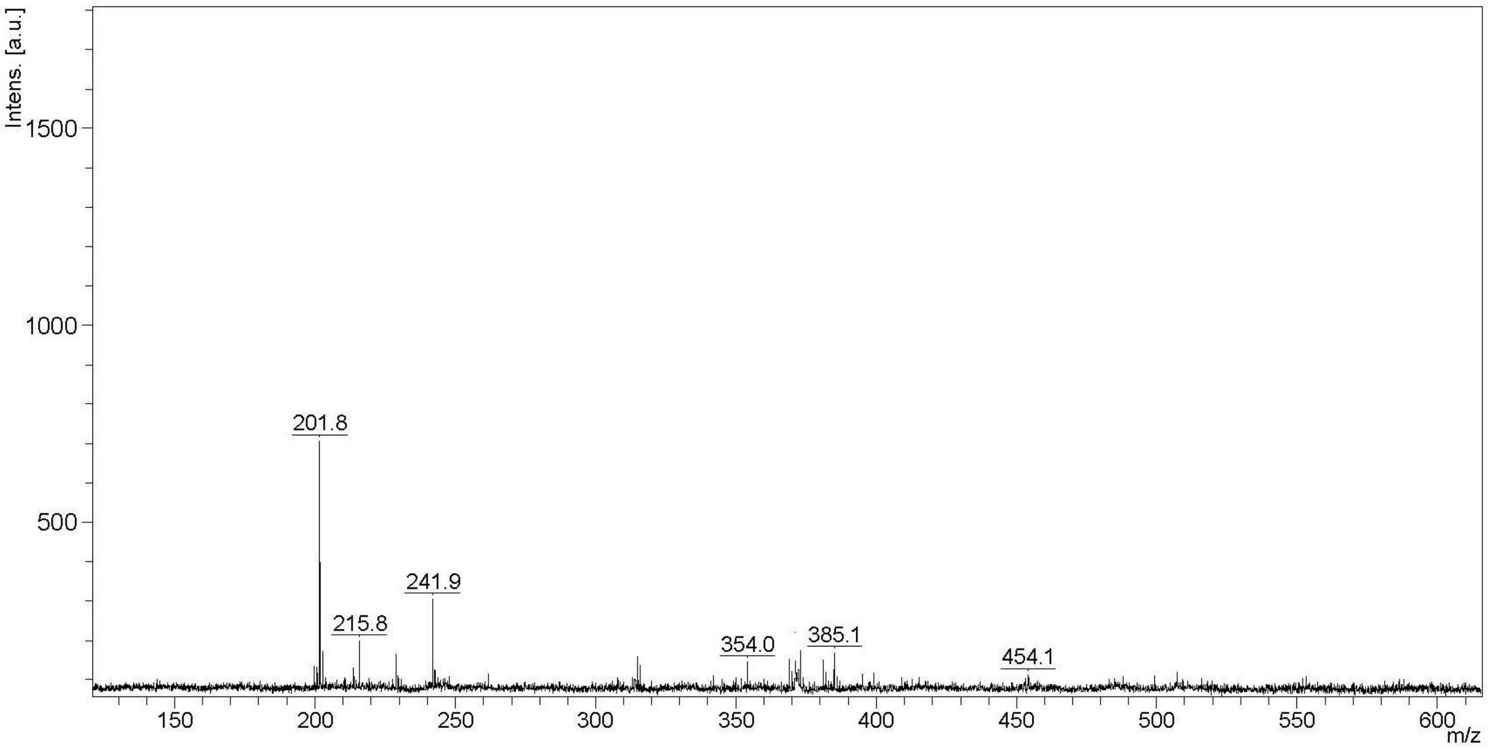

Instrument

Instrument type	ultraflexTOF/TOF
Name of computer	MALDI
flexControl version	flexControl 3.0.173.0
flexAnalysis version	3.0.96.0

Spectrometer

Ion Polarity	POS
PIE delay	50 ns
Ion source voltage 1	20 kV
Ion source voltage 2	19.01 kV
Lens voltage	6 kV
Linear detector voltage	1.498 kV
Reflector voltage 1	0 kV
Reflector voltage 2	0 kV
Reflector detector voltage	1.497 kV

Laser

Ion Source Type	MALDI
Laser Type	Nd:YAG
Wavelength	355 nm
Number of shots	100
Laser repetition rate	100 Hz

Target

Target Plate	MTP AnchorChip
Position	N8

Figure S95. MALDI spectrum of compound **1i**

Acquisition Parameter

Ion Source Type	ESI	Ion Polarity	Positive	Alternating Ion Polarity	off
Mass Range Mode	UltraScan	Scan Begin	70 m/z	Scan End	3000 m/z
Capillary Exit	140.0 V	n/a	n/a	Trap Drive	54.3
Accumulation Time	590 µs	Averages	5 Spectra	n/a	n/a

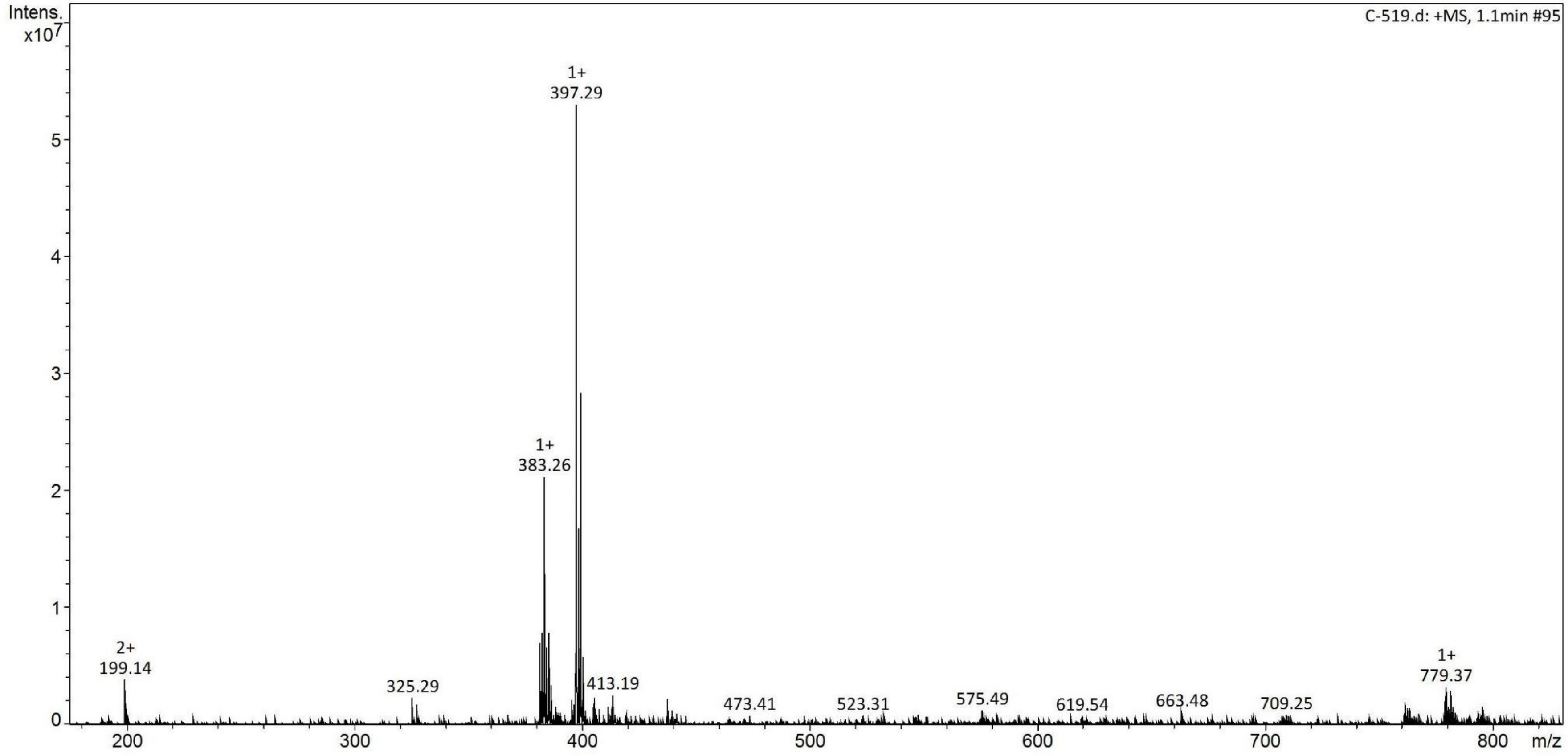

Figure S96. ESI spectrum of compound **1j**

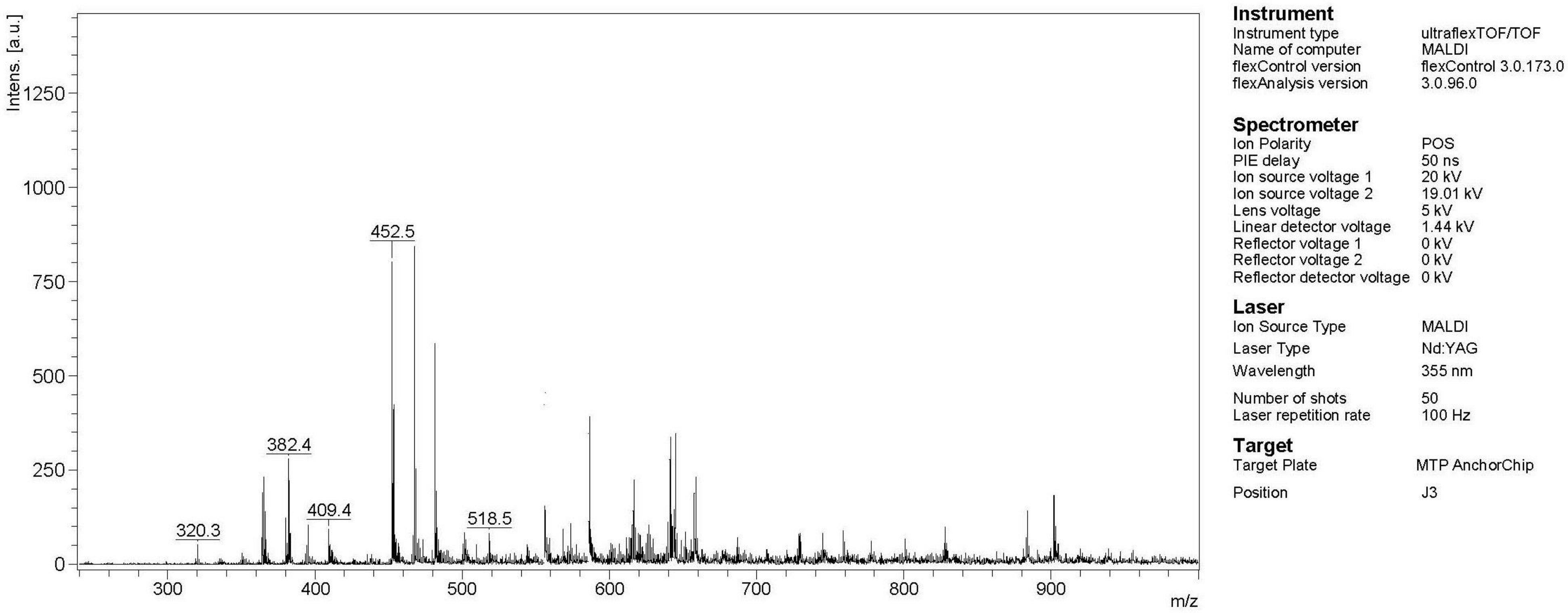

Figure S97. MALDI spectrum of compound **1k**

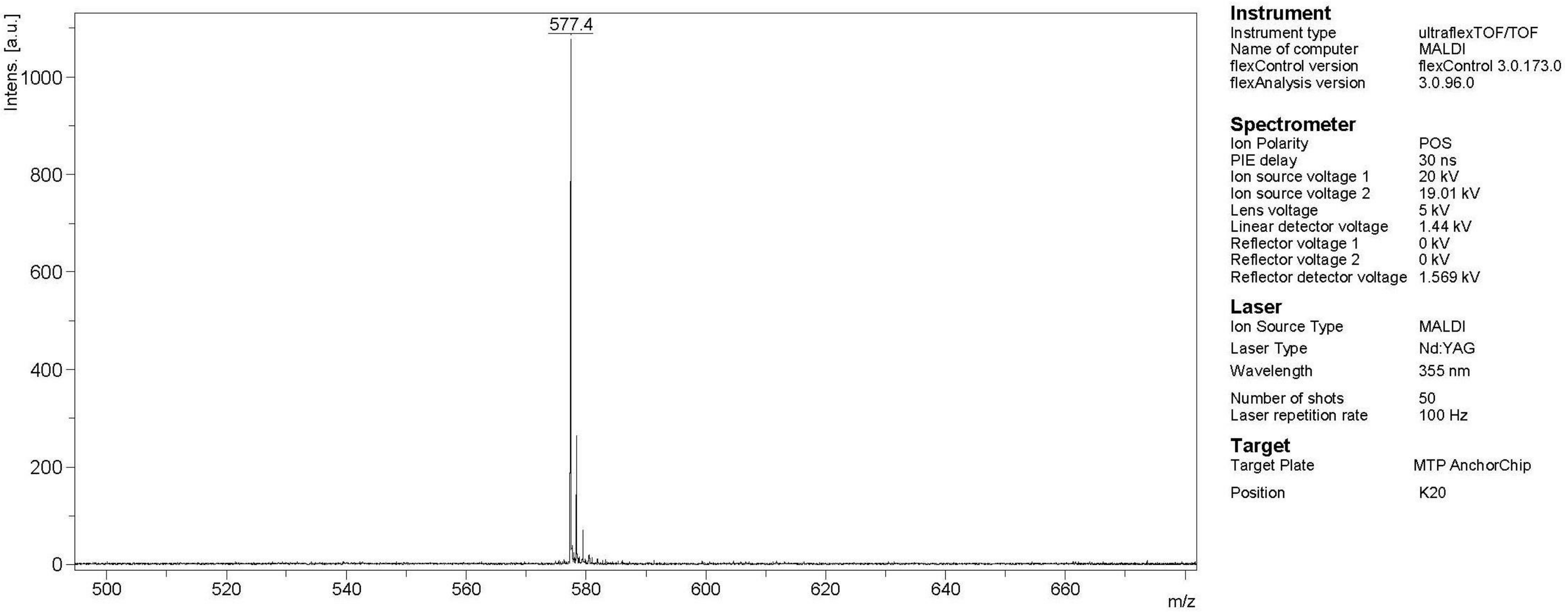

Figure S98. MALDI spectrum of compound **3a**

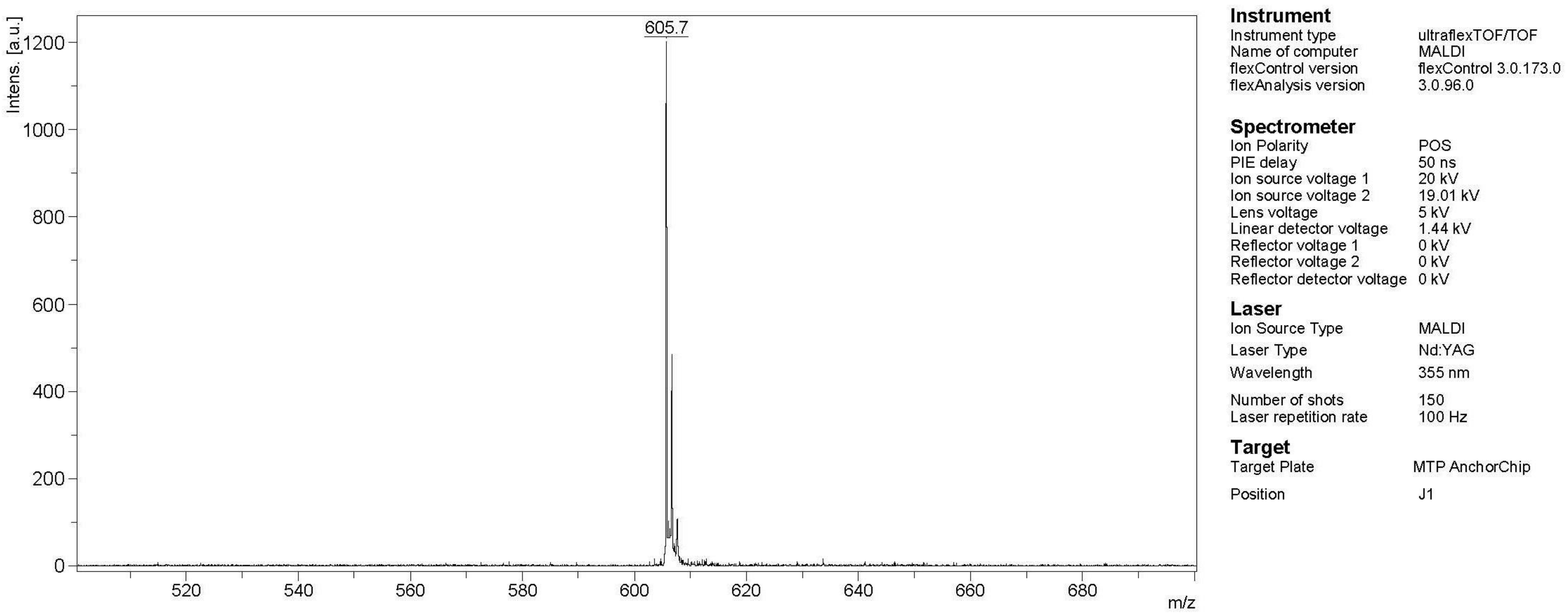

Figure S99. MALDI spectrum of compound **3b**

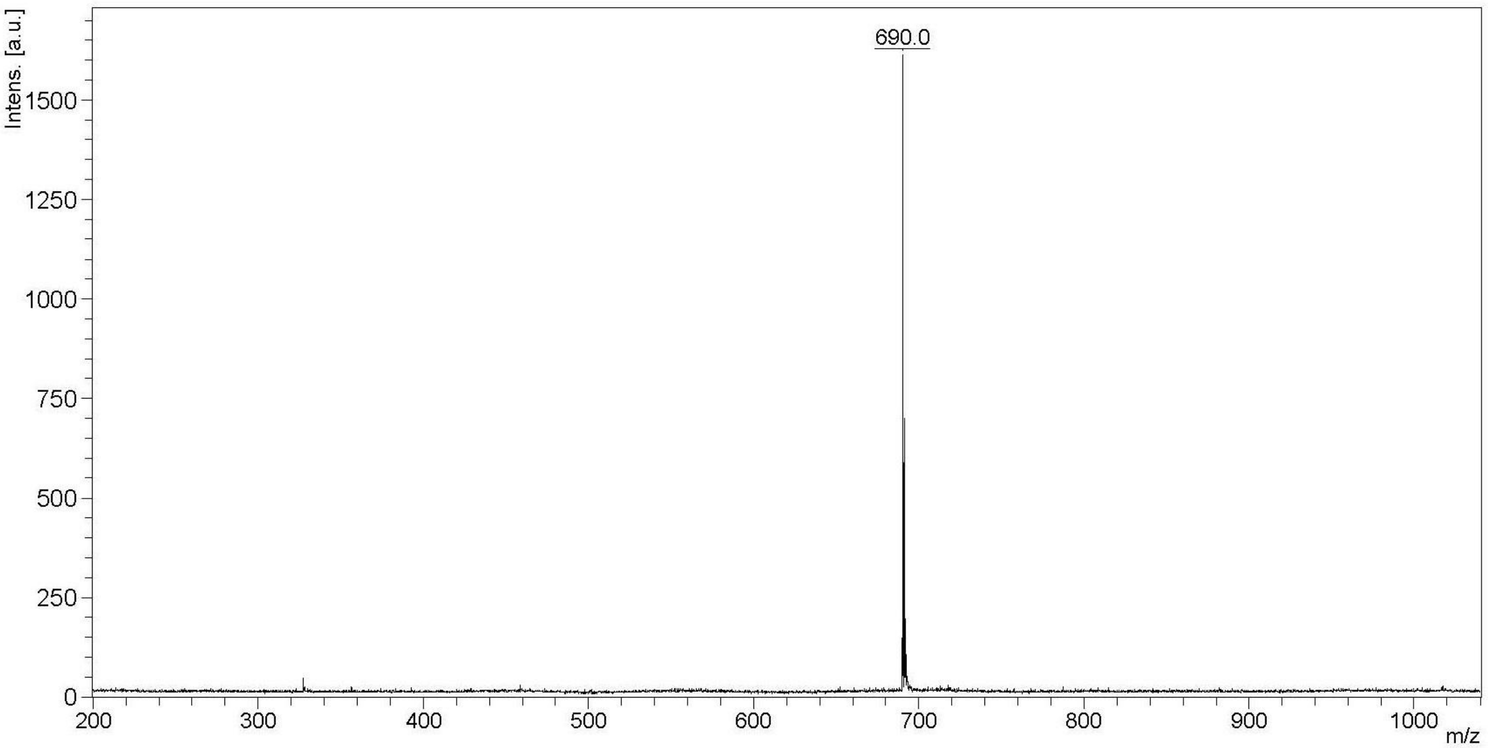

**Instrument**	
Instrument type	ultraflexTOF/TOF
Name of computer	MALDI
flexControl version	flexControl 3.0.173.0
flexAnalysis version	3.0.96.0
**Spectrometer**	
Ion Polarity	POS
PIE delay	30 ns
Ion source voltage 1	20 kV
Ion source voltage 2	19.01 kV
Lens voltage	5 kV
Linear detector voltage	1.44 kV
Reflector voltage 1	0 kV
Reflector voltage 2	0 kV
Reflector detector voltage	1.569 kV
**Laser**	
Ion Source Type	MALDI
Laser Type	Nd:YAG
Wavelength	355 nm
Number of shots	50
Laser repetition rate	100 Hz
**Target**	
Target Plate	MTP AnchorChip
Position	N19

Figure S100. MALDI spectrum of compound **3c**

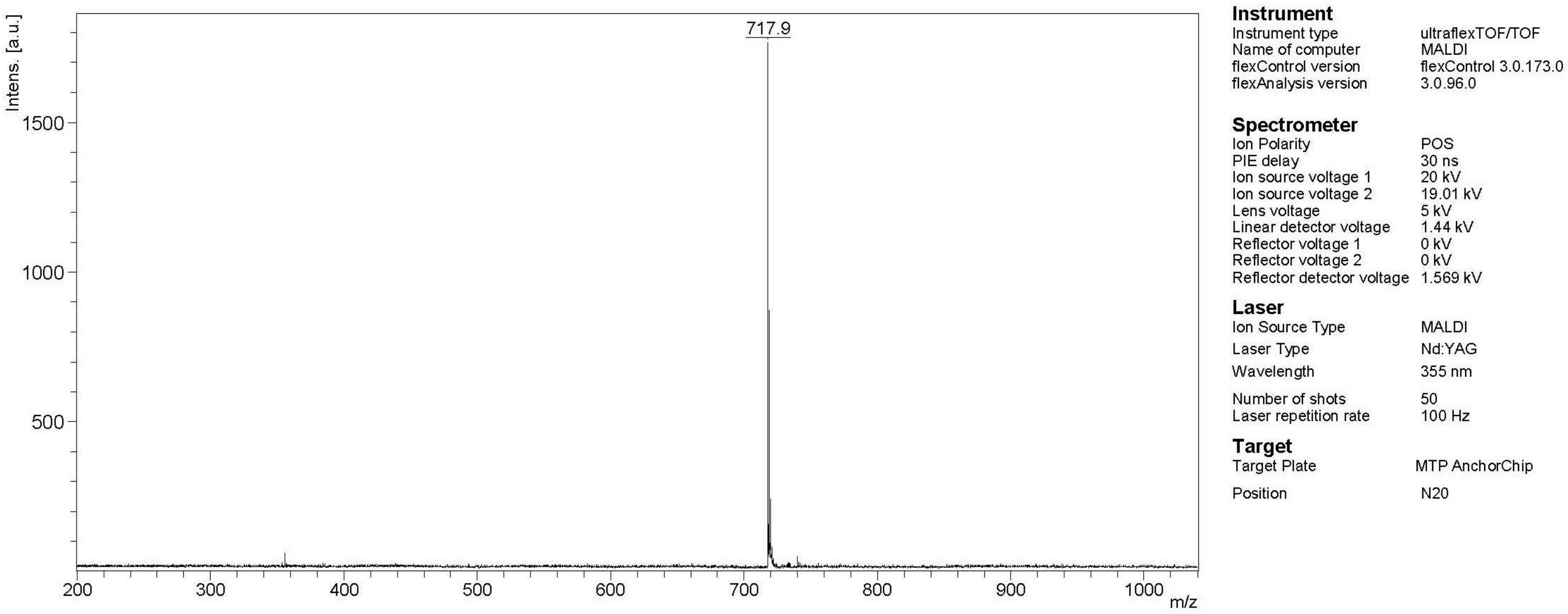

Figure S101. MALDI spectrum of compound **3d**

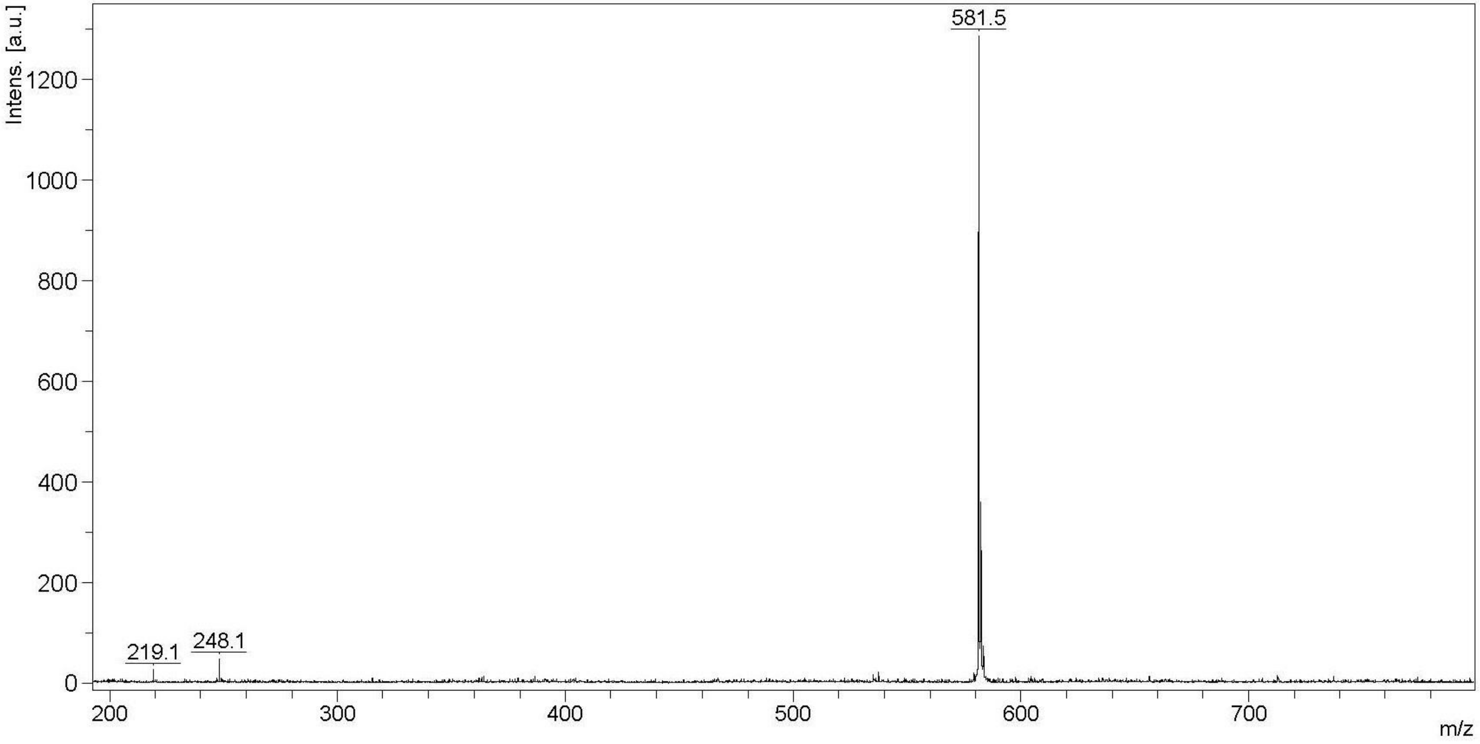

Instrument

Instrument type	ultraflexTOF/TOF
Name of computer	MALDI
flexControl version	flexControl 3.0.173.0
flexAnalysis version	3.0.96.0

Spectrometer

Ion Polarity	POS
PIE delay	30 ns
Ion source voltage 1	20 kV
Ion source voltage 2	19.01 kV
Lens voltage	5 kV
Linear detector voltage	1.44 kV
Reflector voltage 1	0 kV
Reflector voltage 2	0 kV
Reflector detector voltage	1.569 kV

Laser

Ion Source Type	MALDI
Laser Type	Nd:YAG
Wavelength	355 nm
Number of shots	50
Laser repetition rate	100 Hz

Target

Target Plate	MTP AnchorChip
Position	K24

Figure S102. MALDI spectrum of compound **3e**

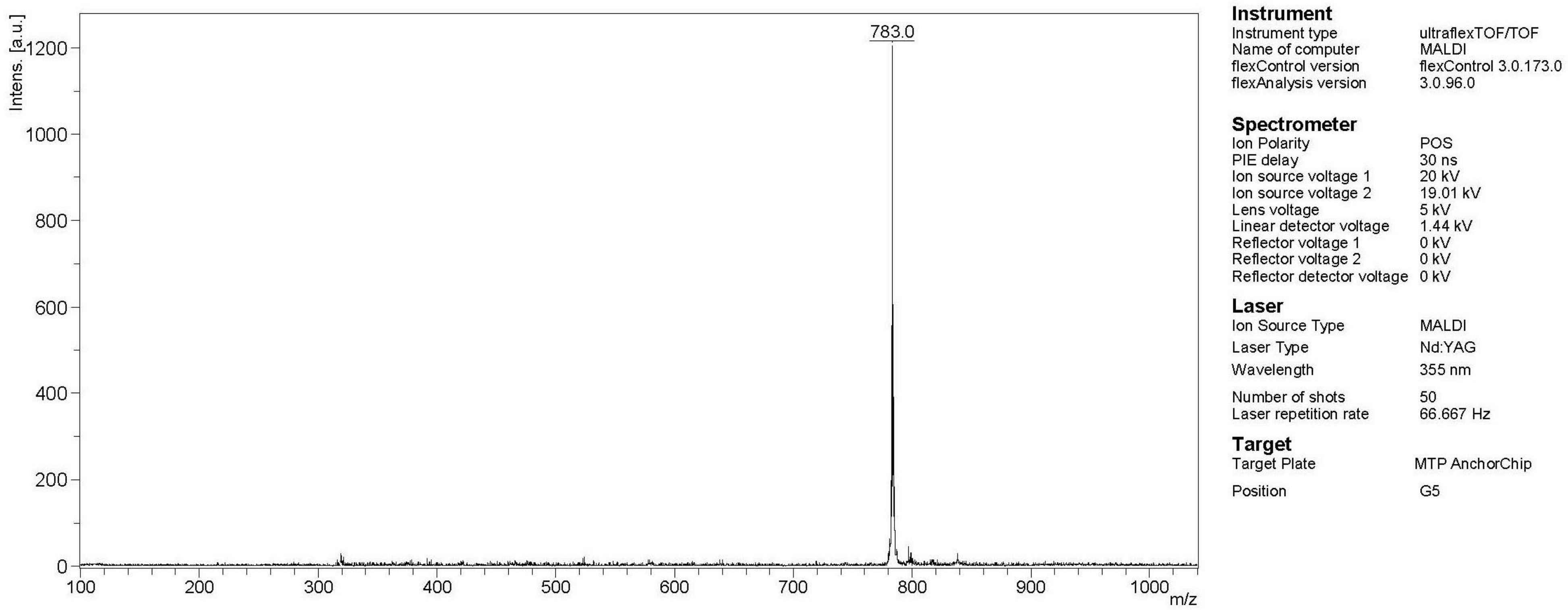

Figure S103. MALDI spectrum of compound **3f**

Acquisition Parameter

Ion Source Type	ESI	Ion Polarity	Positive	Alternating Ion Polarity	off
Mass Range Mode	UltraScan	Scan Begin	100 m/z	Scan End	2000 m/z
Capillary Exit	140.0 V	n/a	n/a	Trap Drive	58.1
Accumulation Time	831 µs	Averages	5 Spectra	n/a	n/a

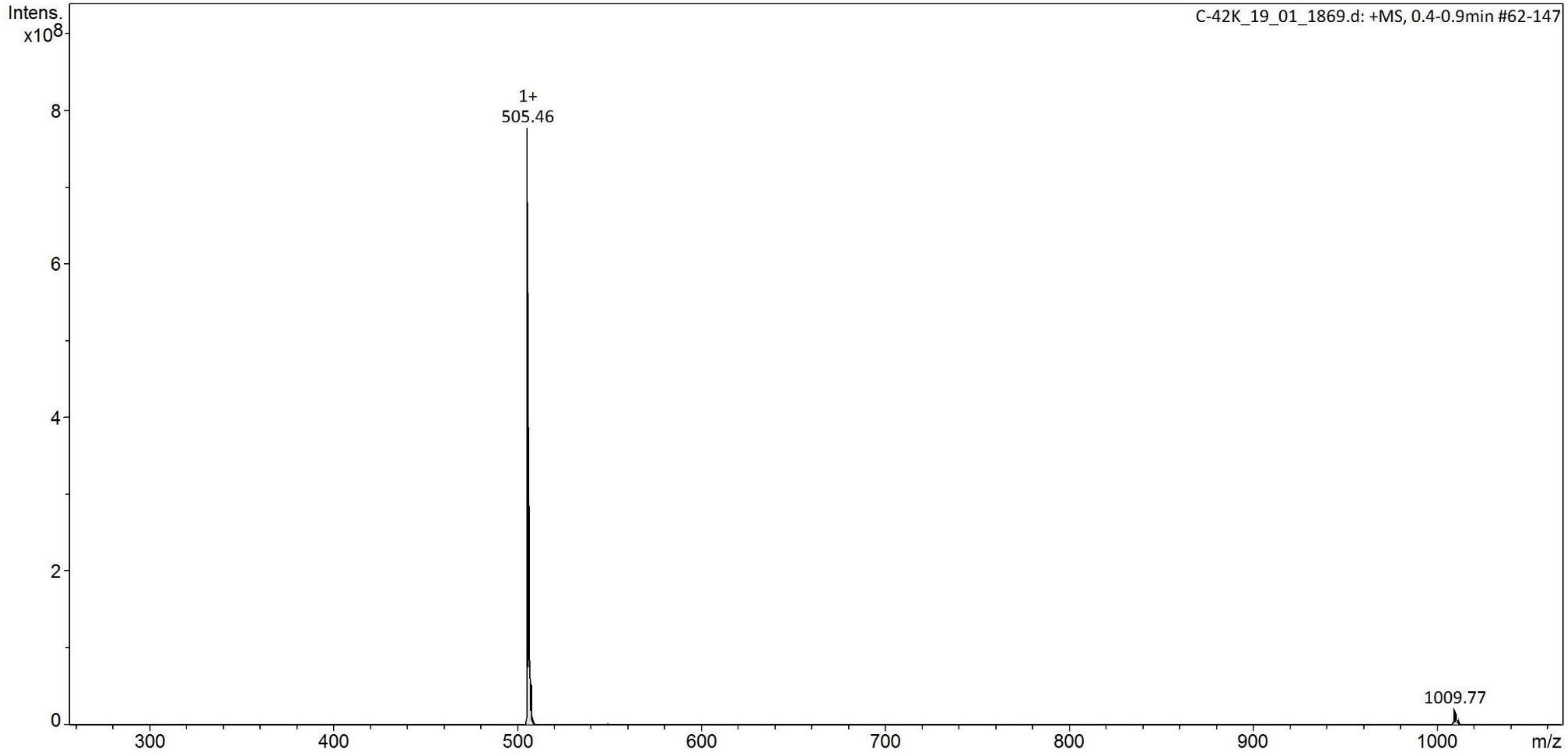

Figure S104. ESI spectrum of compound **3g**

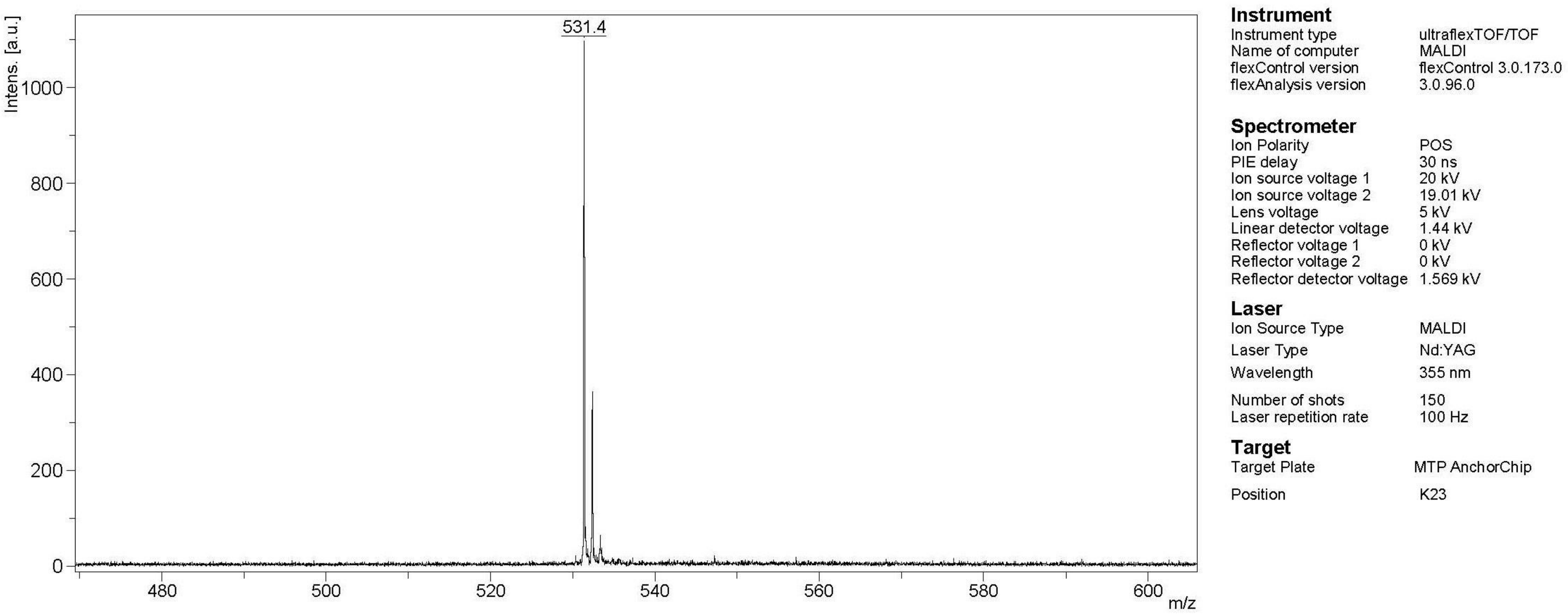

Figure S105. MALDI spectrum of compound **3h**

Acquisition Parameter

Ion Source Type	ESI	Ion Polarity	Positive	Alternating Ion Polarity	off
Mass Range Mode	UltraScan	Scan Begin	100 m/z	Scan End	2000 m/z
Capillary Exit	140.0 V	n/a	n/a	Trap Drive	58.1
Accumulation Time	1300 µs	Averages	5 Spectra	n/a	n/a

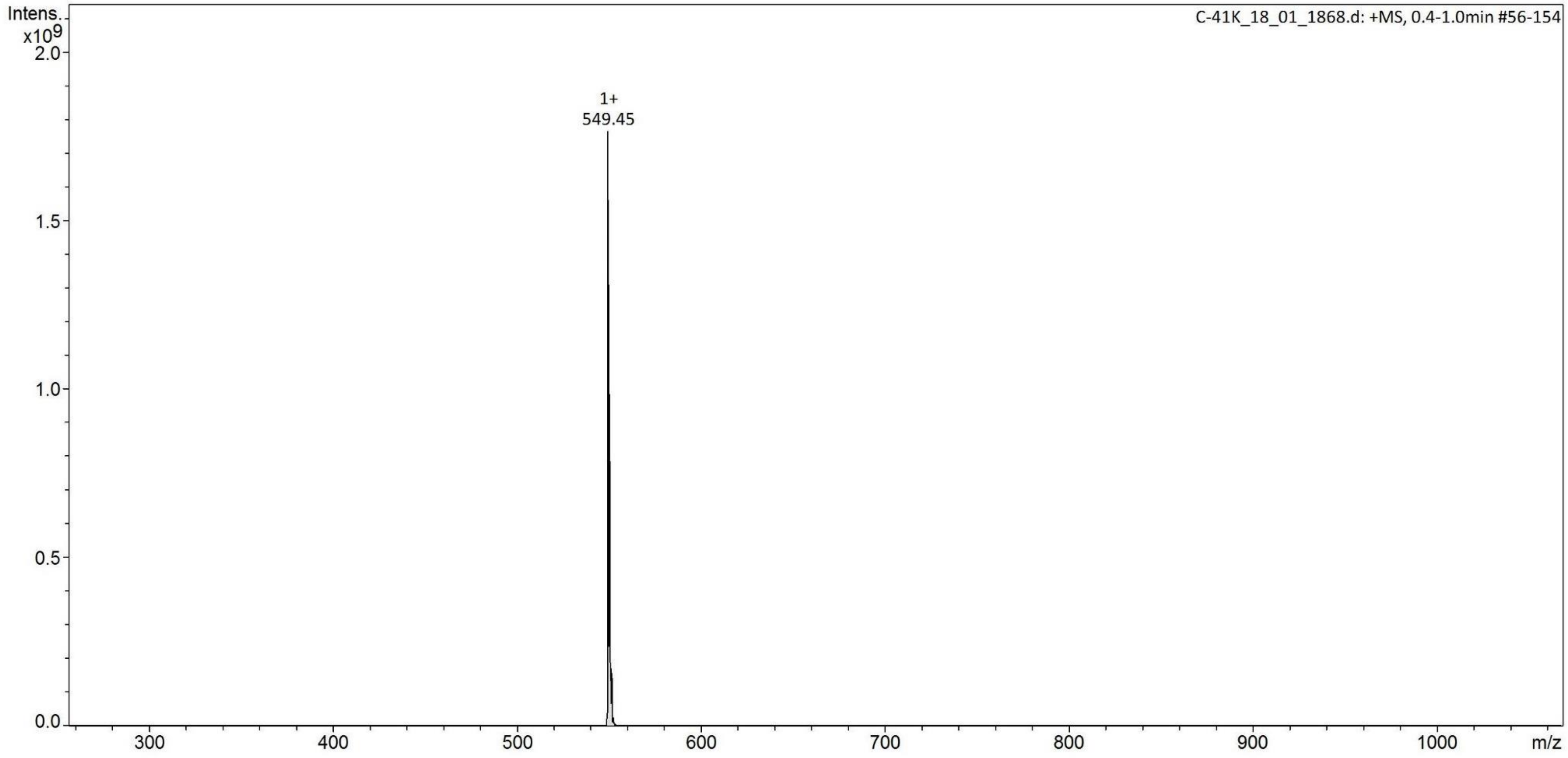

Figure S106. ESI spectrum of compound **3i**

Acquisition Parameter

Ion Source Type	ESI	Ion Polarity	Positive	Alternating Ion Polarity	off
Mass Range Mode	UltraScan	Scan Begin	200 m/z	Scan End	2800 m/z
Capillary Exit	140.0 V	n/a	n/a	Trap Drive	73.1
Accumulation Time	268 µs	Averages	5 Spectra	n/a	n/a

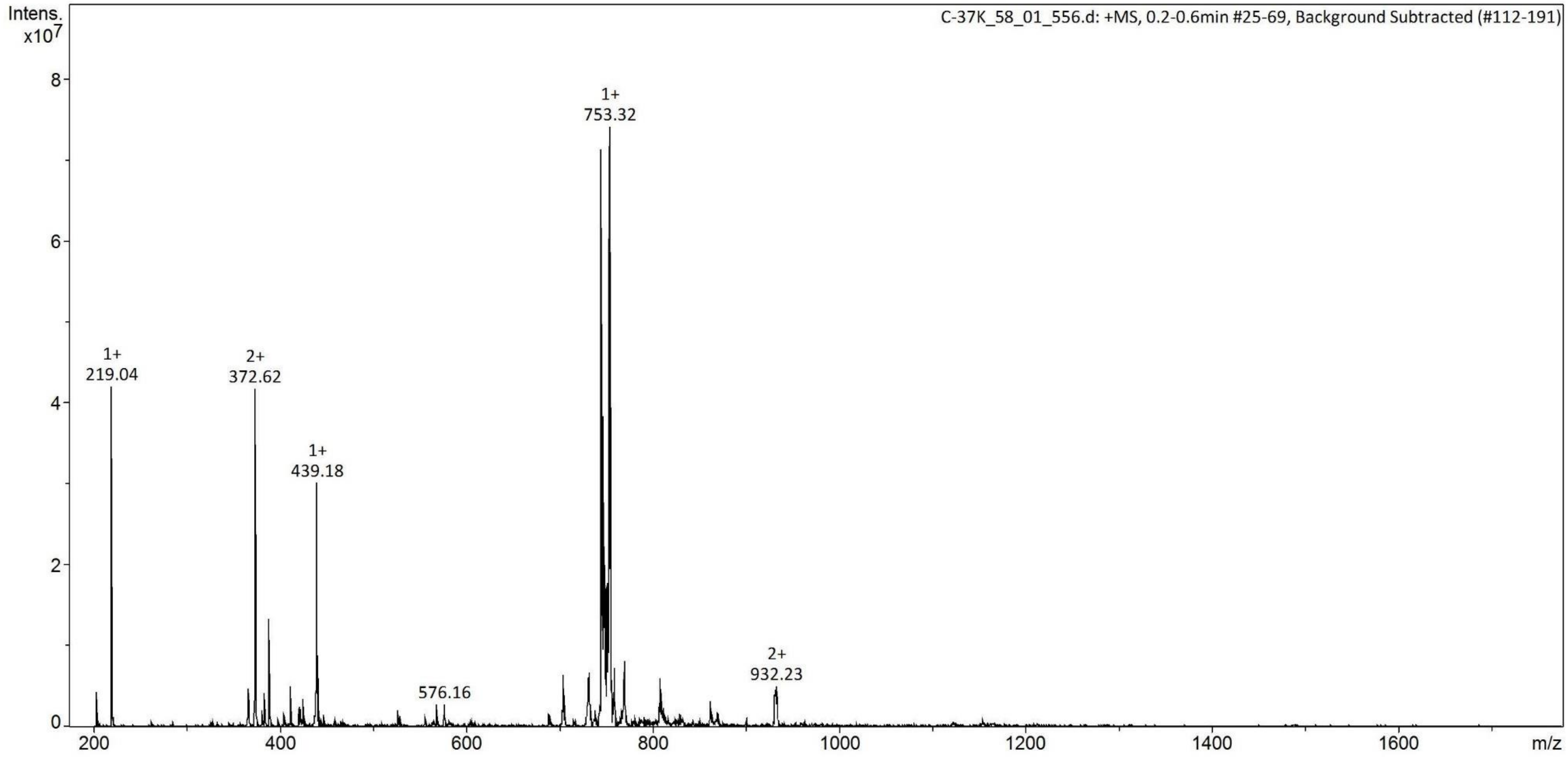

Figure S107. ESI spectrum of compound **3j**

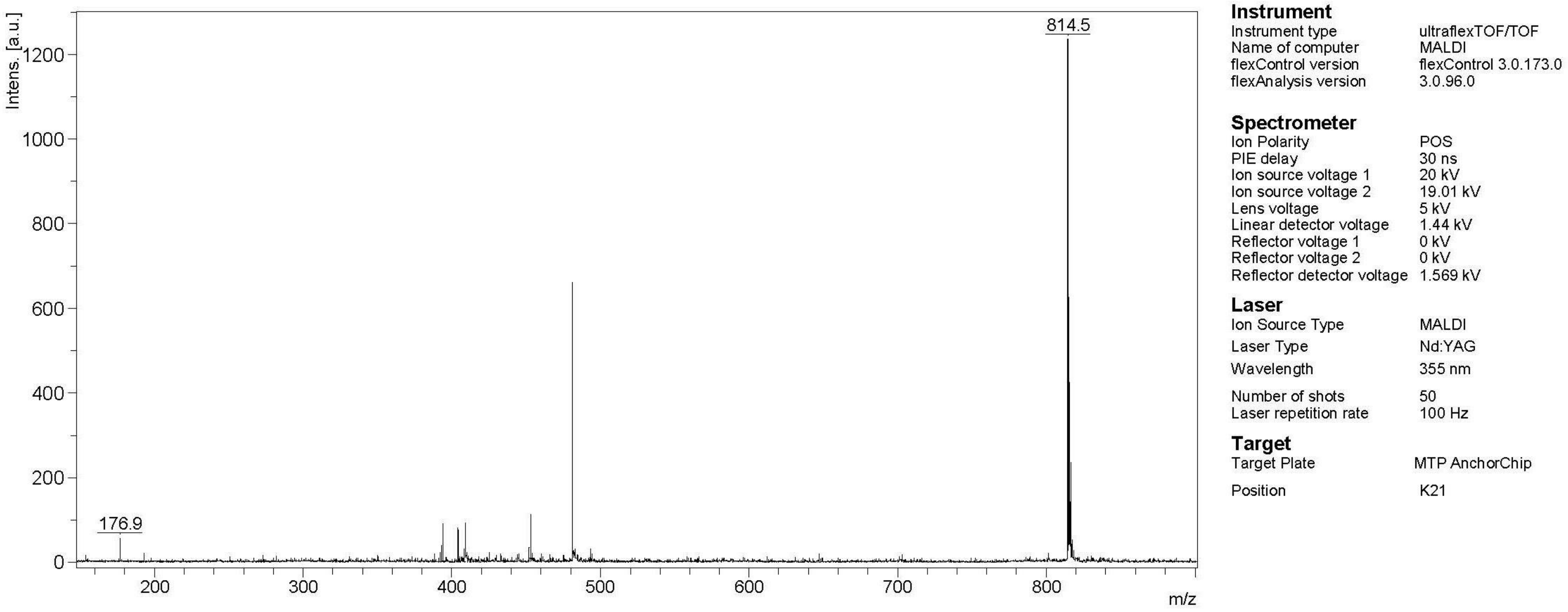

Figure S108. MALDI spectrum of compound **3k**

Acquisition Parameter

Ion Source Type	ESI	Ion Polarity	Positive	Alternating Ion Polarity	off
Mass Range Mode	UltraScan	Scan Begin	70 m/z	Scan End	2700 m/z
Capillary Exit	140.0 V	n/a	n/a	Trap Drive	73.1
Accumulation Time	556 µs	Averages	5 Spectra	Auto MS/MS	off

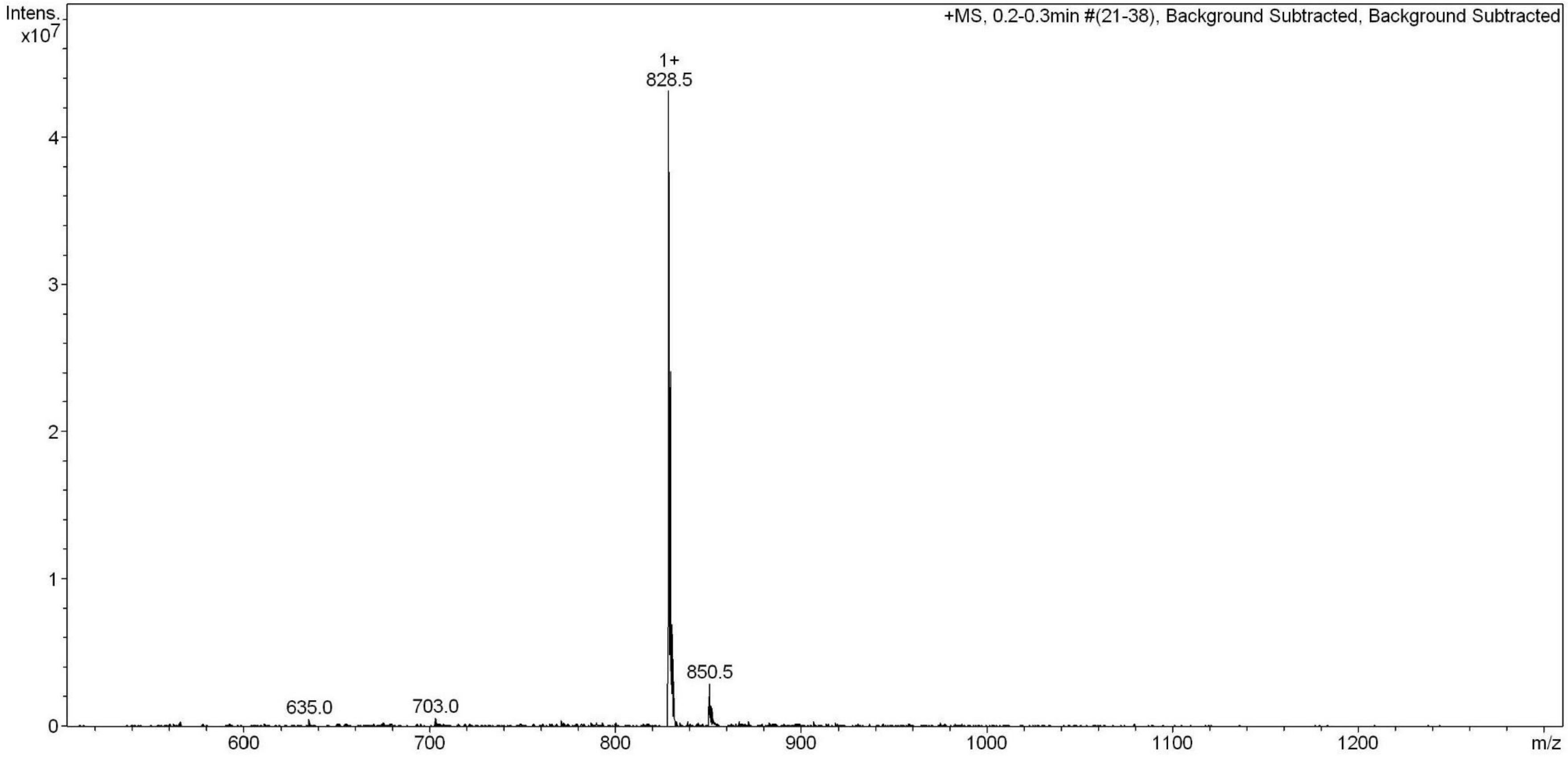

Figure S109. ESI spectrum of compound **3l**

Acquisition Parameter

Ion Source Type	ESI	Ion Polarity	Positive	Alternating Ion Polarity	off
Mass Range Mode	UltraScan	Scan Begin	70 m/z	Scan End	2700 m/z
Capillary Exit	140.0 V	n/a	n/a	Trap Drive	73.1
Accumulation Time	543 µs	Averages	5 Spectra	Auto MS/MS	off

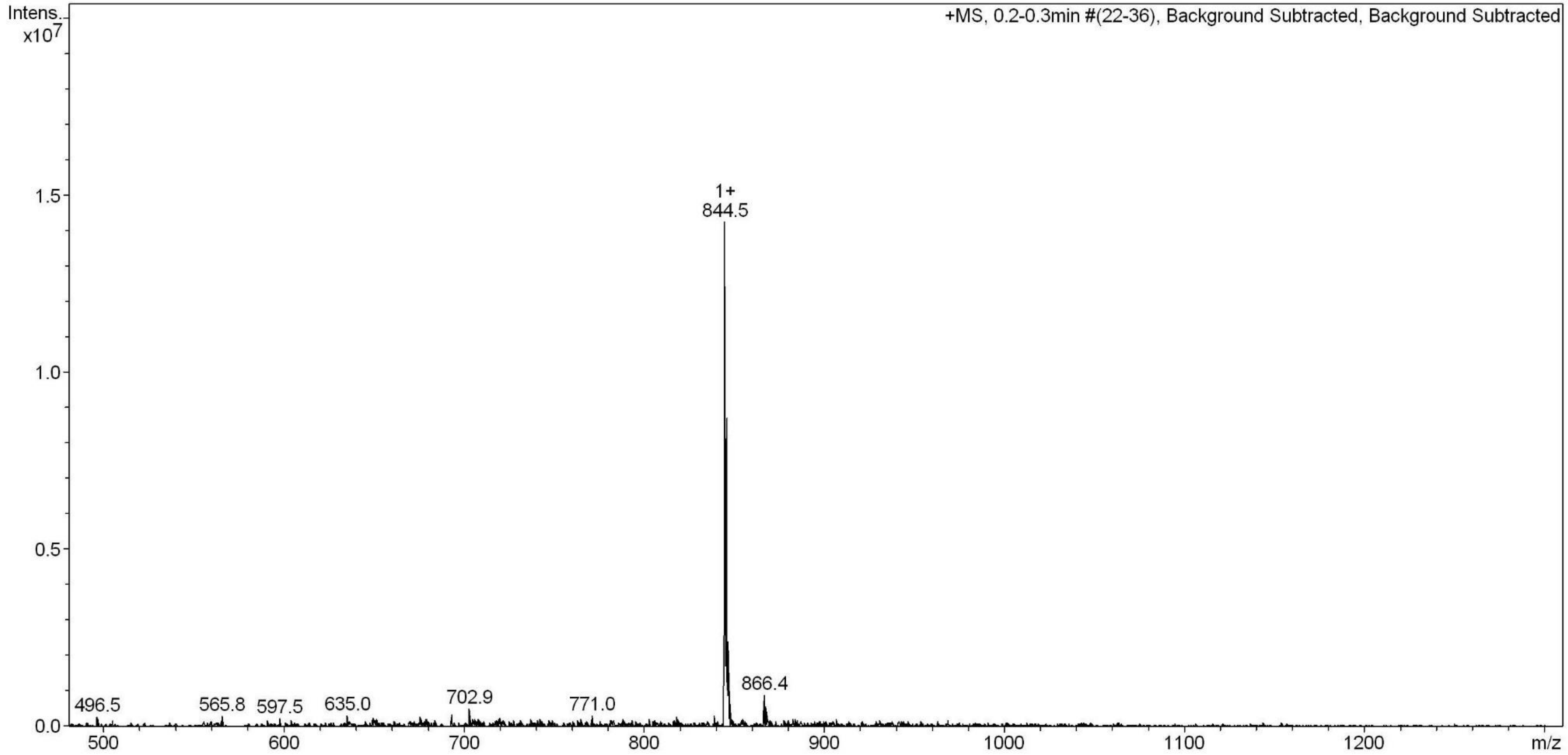

Figure S110. ESI spectrum of compound **3m**

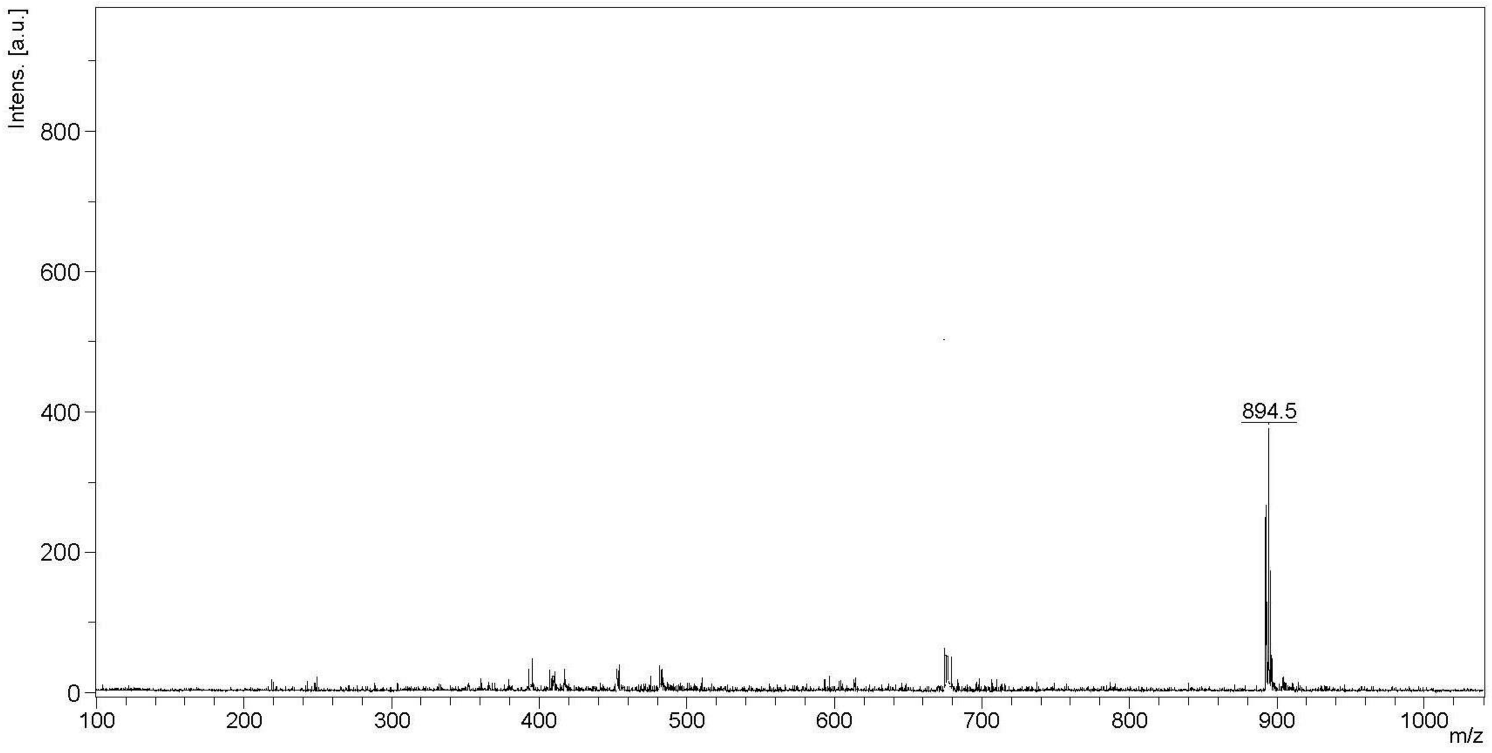

Instrument

Instrument type	ultraflexTOF/TOF
Name of computer	MALDI
flexControl version	flexControl 3.0.173.0
flexAnalysis version	3.0.96.0

Spectrometer

Ion Polarity	POS
PIE delay	30 ns
Ion source voltage 1	20 kV
Ion source voltage 2	19.01 kV
Lens voltage	5 kV
Linear detector voltage	1.44 kV
Reflector voltage 1	0 kV
Reflector voltage 2	0 kV
Reflector detector voltage	1.569 kV

Laser

Ion Source Type	MALDI
Laser Type	Nd:YAG
Wavelength	355 nm
Number of shots	100
Laser repetition rate	100 Hz

Target

Target Plate	MTP AnchorChip
Position	J18

Figure S111. MALDI spectrum of compound **3n**
